# *Mesostigma viride* Genome and Transcriptome Provide Insights into the Origin and Evolution of Streptophyta

**DOI:** 10.1002/advs.201901850

**Published:** 2019-10-24

**Authors:** Zhe Liang, Yuke Geng, Changmian Ji, Hai Du, Chui Eng Wong, Qian Zhang, Ye Zhang, Pingxian Zhang, Adeel Riaz, Sadaruddin Chachar, Yike Ding, Jing Wen, Yunwen Wu, Mingcheng Wang, Hongkun Zheng, Yanmin Wu, Viktor Demko, Lisha Shen, Xiao Han, Pengpeng Zhang, Xiaofeng Gu, Hao Yu

**Affiliations:** ^1^ Department of Biological Sciences National University of Singapore Singapore 117543 Singapore; ^2^ Biotechnology Research Institute Chinese Academy of Agricultural Sciences Beijing 100081 China; ^3^ Biomarker Technologies Beijing 101300 China; ^4^ Institute of Tropical Bioscience and Biotechnology Chinese Academy of Tropical Agricultural Sciences Haikou 571101 China; ^5^ College of Agronomy and Biotechnology Southwest University Chongqing 400715 China; ^6^ Department of Entomology University of California Riverside Riverside CA 92521 USA; ^7^ Department of Plant Physiology Faculty of Natural Sciences Comenius University in Bratislava Bratislava 84215 Slovakia; ^8^ Temasek Life Sciences Laboratory National University of Singapore Singapore 117604 Singapore; ^9^ College of Biological Science and Engineering Fuzhou University Fuzhou 350108 China

**Keywords:** evolution, green algae, *Mesostigma viride*, multicellularity, Streptophyta

## Abstract

The Streptophyta include unicellular and multicellular charophyte green algae and land plants. Colonization of the terrestrial habitat by land plants is a major evolutionary event that has transformed the planet. So far, lack of genome information on unicellular charophyte algae hinders the understanding of the origin and the evolution from unicellular to multicellular life in Streptophyta. This work reports the high‐quality reference genome and transcriptome of *Mesostigma viride*, a single‐celled charophyte alga with a position at the base of Streptophyta. There are abundant segmental duplications and transposable elements in *M. viride*, which contribute to a relatively large genome with high gene content compared to other algae and early diverging land plants. This work identifies the origin of genetic tools that multicellular Streptophyta have inherited and key genetic innovations required for the evolution of land plants from unicellular aquatic ancestors. The findings shed light on the age‐old questions of the evolution of multicellularity and the origin of land plants.

## Graphical abstract

*Mesostigma viride* is a single‐celled freshwater biflagellate charophyte green alga and represents the earliest diverging lineage of streptophytes. Herein, its high‐quality reference genome and transcriptome are presented, which reveal the origin and innovations of key genetic tools in Streptophyta, and shed light on the evolution of multicellularity and the origin of land plants.

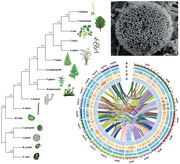

## 1 Introduction

One of the most important evolutionary innovations in the history of life is multicellularity, which contains simple (colonial, filamentous) and complex forms with elaborate cell–cell communication and network of genetic interactions for coordinated cell division and differentiation.^1^ Multicellularity has arisen multiple times independently in eukaryotes including animals, fungi, Amoebozoa, charophyte green algae, chlorophyte green algae, and red and brown algae.^2^ Comparisons of genomic and cellular traits of multicellular organisms with those of their unicellular relatives have gained important understanding of the evolution of multicellularity in several eukaryotic lineages, such as animals, fungi, and chlorophyte green algae.^3^ However, how the multicellularity of charophyte algae and land plants evolved from unicellular charophyte algae predecessors remains largely unknown.

All living green plants belong to one of the two major phyla: Streptophyta division containing the charophyte green algae in freshwater habitat and all land plants, and the Chlorophyta division with the other green algae.^4^ Charophyte algae are a morphologically diverse group encompassing unicellular and structurally complex multicellular forms with six distinct major lineages: Mesostigmatophyceae, Chlorokybophyceae, Klebsormidiophyceae, Zygnematophyceae, Charophyceae, and Coleochaetophyceae.^5^ Mesostigma (Mesostigmatophyceae) and Chlorokybus (Chlorokybophyceae) are representatives of the earliest diverging lineages of streptophytes.^6^ As the closest relatives of land plants, multicellular charophyte algae contain many important biological characters that were adopted by land plants.^7^

**Figure 1:**
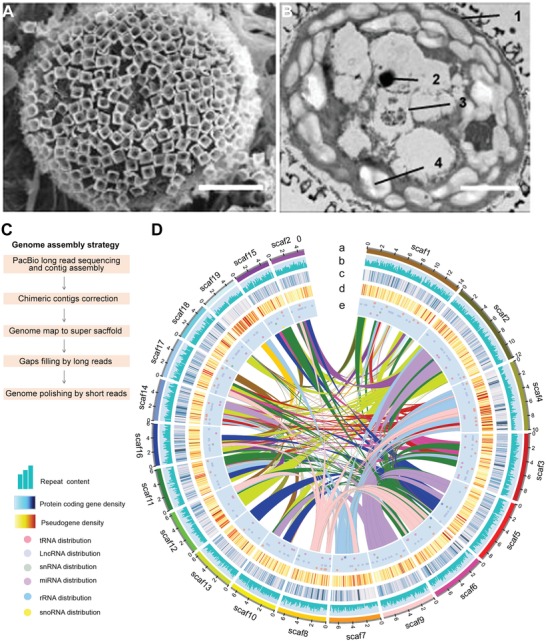
*M. viride* morphology and genome assembly. A) Scanning electron micrograph of *M. viride* cell surface shows its unified basket‐like scales. Scale bar, 2.5 µm. B) Ultrastructure of a *M. viride* cell observed under transmission electron microscope. 1, cytoderm; 2, pyrenoid; 3, eyespots; 4, starch granule. Scale bars, 2.5 µm. C) The assembly of the *M. viride* genome combines PacBio long reads, Illumina short reads, and optical map generated from Saphyr System. D) Circos plot depicting the genome content based on the 20 longest scaffolds in a 200 kb nonoverlapping window. Numbers on the circumference are at the megabase scale. “a” track represents the 20 longest scaffolds of *M. viride*, while the distribution of repeat b), gene c), pseudogene d), and ncRNA e), including tRNA, lncRNA, snRNA, miRNA, rRNA, and snoRNA, are indicated in the other tracks. Linked lines in the middle of the Circos plot connect syntenic blocks (minimum five gene pairs) from the most recent segmental duplication events. Different colors were used to distinguish different scaffolds a) or syntenic blocks (linked lines).

Currently, all sequenced plant genomes within the Streptophyta division are from multicellular charophyte algae and land plants, which make it difficult to investigate the origin and the genetic “toolkits” of plant multicellularity. *Mesostigma viride* is an extant unicellular biflagellate freshwater charophyte algae covered by an outer layer of basket‐like scales instead of cell wall (**Figure** **1**A,B; Figure S1, Supporting Information). It is the only known flagellate charophyte algae with a multilayered structure,^8^ and is one of the earliest diverging members of streptophytes.^6^, ^9^ This crucial phylogenetic position in the evolution of green plants makes *M. viride* an essential model for understanding the evolution of multicellularity and the origin of land plants.

Here we report the high‐quality reference genome of *M. viride* by combining single molecule real‐time sequencing, Illumina sequencing and optical mapping. Comparative analyses of its genome and transcriptome with those of other green algae and early diverging land plants allow us to identify the origin of key genetic tools that multicellular charophyte algae and land plants have either inherited or created during the evolution from unicellular to multicellular green plants for colonization of the terrestrial habitat in our planet.

## 2 Results and Discussion

### 2.1 *Mesostigma viride* Genome Assembly and Annotation

We assembled the reference genome of *Mesostigma viride* (strain NIES‐296) using a combination of the generated Illumina short reads (162 × coverage), Pacific Biosciences (PacBio) long reads (113 × coverage; N50 read length 11.2 kb), and optical mapping data (203.6 × coverage; Molecule N50 229 kb) (Figure 1C; Table S1A, Supporting Information). The final hybrid assembly yielded 2363 scaffolds (scaffold N50 = 2.6 Mb) covering 442 Mb (Figure 1D and **Table** **1**; Table S1B, Supporting Information), which is the second largest available genome of green algae after *Chara braunii*.[qv: 7a] Using the Benchmarking Universal Single‐Copy Orthologs (BUSCO) plant database,^10^ we detected 90.1% complete and 5.0% fragmented BUSCO genes (Table S1C, Supporting Information). Illumina short reads and single‐molecule real‐time (SMRT) subreads could also be remapped to the assembly results (Experimental Section), demonstrating the high quality of our genome sequence assembly. To facilitate genome annotation, we also performed RNA sequencing (RNA‐seq) on small RNAs, long noncoding RNAs (lncRNAs) and mRNAs isolated from *M. viride* using a combination of Illumina and PacBio sequencing technologies (Table S1D, Supporting Information). Our annotation revealed 24431 putative protein‐coding genes, among which more than 90% genes were supported by expression data. There were 2540 noncoding RNA genes supported by RNA‐seq data, including 652 tRNA, 73 rRNA, 116 miRNA, 11 snRNA, 5 snoRNA, and 1680 lncRNA genes (Figure 1D and Table 1; Table S1E–K, Supporting Information). We also annotated 7570 pseudogenes with frameshifts and/or premature stop codon mutations (Table S1L, Supporting Information).

**Table 1:** Statistics of *M. viride* genome assembly and annotation

Feature	*M. viride*
Genome size [bp]	441 847 188
Contig number	3074
Maximum contig length [bp]	2003 508
Contig N50 [bp]	319 906
Contig N90 [bp]	56 379
Scaffold N50 [bp]	2558 729
Scaffold N90 [bp]	58 377
Gap ratio [%]	0.04
Gene number	24 431
Average gene length [bp]	5940.83
CDS length [bp]	1585.60
Exons number per gene	4.81
Exon length [bp]	329.36
Exons number per gene	3.81
Intron length [bp]	1141.87

### 2.2 Gene and Genome Evolution

**Figure 2:**
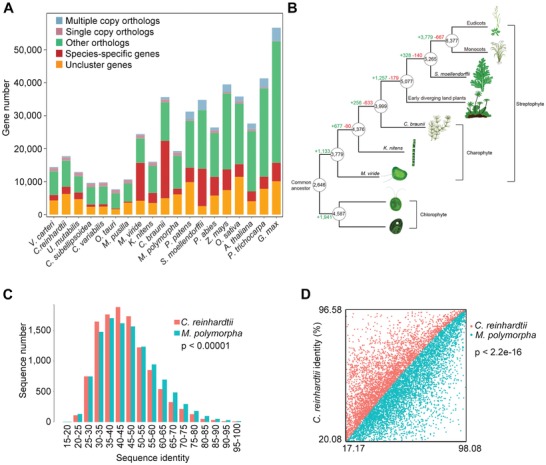
Evolutionary analysis of *M. viride* with other selected green plant species. A) Gene family clustering statistics. The *M. viride* genome contains a large portion of species‐specific genes, which represent those belonging to a gene family that only exists in a particular species. Multiple and single copy orthologs include the common orthologs with different copy numbers in the species studied. Other orthologs include unclassified orthologs, whereas unclustered genes include those that are not assigned into any gene families. B) Gene family gains (+) and losses (−) mapped onto the plant phylogenetic tree. The minimum numbers of gene families present in the ancestors of different plant lineages are circled. Branch lengths are arbitrary. The analysis includes all the species in (A), only the representative species for each lineage are shown in the schematic diagram. C,D) Frequency distribution with Chi‐square test (C) and scatter plot with two‐sample Kolmogorov–Smirnov test of protein sequence identity (D) between 11 239 homologous gene pairs of *M. viride* versus *C. reinhardtii* and *M. viride* versus *M. polymorpha*. Only 1:1:1 common orthologs of *M. viride*, *C. reinhardtii*, and *M. polymorpha* were considered. There is a significantly higher identity between homologous gene pairs in *M. viride* versus *M. Polymorpha*. Red or blue represents the sequence identity between *M. viride* and *C. reinhardtii* or between *M. viride* and *M. polymorpha*, respectively.

Comparative analysis of gene families across Viridiplantae (green plants) showed that the gene number of *M. viride* was lower to that of *C. braunii*,[qv: 7a] but higher than all the other known sequenced green algae (**Figure** **2**A). Interestingly, almost half of the putative protein coding genes (11507), which were designated as species‐specific genes, were unique to *M. viride* without any homolog detected among the 18 selected Chloroplastida groups (Figure 2A). We performed phylogenetic analyses of representative land plants and green algae species based on 117 single‐copy orthologs. The resulting topology revealed that *M. viride* was one of the earliest diverging green plant lineages as a basally branching member of the streptophytes (Figure S2A, Supporting Information), which is in agreement with previous studies using chloroplast DNA or transcriptome data.[qv: 9b,11] We subsequently used a homology‐based approach to distinguish gene family gains and losses among selected plant species and mapped these onto the phylogenetic tree (Figure 2B). In agreement with a previous study suggesting that the number of transcription factors increases with organismal complexity,^12^ the number of gene families seems to correlate with morphological complexity (Figure 2B). The chlorophyte algae gene set (4587 families) and *M. viride* gene set (3779 families) evolved from 2646 common gene families, which were present in all green lineages and defined the minimum set of genes that were likely to be present in the common ancestor of all green plants. There was a net increase with little loss of gene families in the evolution from single‐celled *M. viride* to filamentous multicellular *Klebsormidium nitens*,[qv: 7d] and from *C. braunii* to the nonvascular early diverging land plants, *Marchantia polymorpha* and *Physcomitrella patens*.^13^ Evolution from nonvascular to early diverging vascular plant *Selaginella moellendorffii* was associated with the gain of far fewer new gene families (328),^14^ whereas there was a substantial increase in gene families from early diverging vascular plants to flowering plants. Only the evolution from *K. nitens* to *C. braunii* was associated with more losses in gene families than gains (Figure 2B). Notably, the transport‐related genes, such as amino acid and P—P‐bond‐hydrolysis‐driven protein transmembrane transporter genes, were significantly gained in *K. nitens* compared to *M. viride* (Figure S2B, Supporting Information), coinciding with the novel cell–cell transport/communication systems that may contribute to multicellularity.[qv: 7d] In addition, the gene sequence identity between *M. viride* and early diverging land plants was significantly higher than that between *M. viride* and chlorophyte algae (Figure 2C,D). This corroborates the greater similarity of genetic characters between *M. viride* and land plants than that between the unicellular charophyte and chlorophyte algae, implying that *M. viride* could evolve with many genetic innovations relevant to land plants compared to chlorophyte algae after the early green plant split.

### 2.3 Duplication and Repetitive Sequences

**Figure 3:**
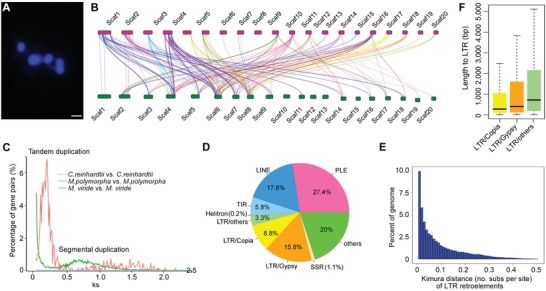
Duplication and repetitive sequences in *M. viride*. A) Fluorescent microscopy of chromosome number in *M. viride*. The sample was stained with DAPI. Scale bar, 1 µm. B) A schematic diagram showing segmental duplications in the 20 longest scaffolds. Colored lines connect syntenic blocks (minimum five gene pairs) from the most recent segmental duplication events. C) Frequency distribution of values of synonymous substitutions Ks (synonymous substitutions/synonymous site) between pairs of paralogs in *M. viride*, *C. reinhardtii* and *M. polymorpha*. The peak in *C. reinhardtii* represents 698 tandem duplicated genes at Ks = 0.14, whereas the peak in *M. viride* indicates a possible early whole genome duplication of *M. viride* at Ks = 0.7. The latter comparison consists of 56595 paralogous gene pairs. D) Pie chart illustrating major repeat classes in the *M. viride* genome. LTR, long terminal repeat; LINE, long interspersed nuclear element; PLE, Penelope‐like element; TIR, terminal inverted repeat. E) Box plots showing the length distribution of LTR families in the *M. viride* genome. Boxes indicate the first quartile, median and third quartile with whiskers extending up to 1.5 times the interquartile distance. F) Relative age (Kimura distance) computed for LTR retroelements suggests a prolonged transposition activity of the retroelements.

The haploid genome of *M. viride* was encoded on 5 chromosomes (**Figure** **3**A) with many segmental duplications (SDs) and a possible whole genome duplication (Figures 1D and 3B,C; Table S1M, Supporting Information). The relatively high Ks value (the number of synonymous substitutions per synonymous site) of *M. viride* compared to that of *Chlamydomonas reinhardtii* and *M. polymorpha* suggests that these SDs emerge within *M. viride*. This indicates that gene family expansion may occur at the basal lineage of Streptophyta. Repetitive elements represented 66.02% of the *M. viride* genome assembly (Table S1N, Supporting Information), a value that was similar to *C. braunii* (61%) but much higher than that of early diverging land plants, *M. polymorpha* (22%) and *P. patens* (48%), and other green algae (Figures S3A–D and Table S1N, Supporting Information). Long terminal repeat (LTR) retroelements (60339 in total) constituted the largest portion of the repetitive elements (27.9%; Figure 3D; Table S1N, Supporting Information), which is similar to that in angiosperms and gymnosperms.^15^ The LTR length in *M. viride* was considerably longer than those found in *K. nitens*, *C. reinhardtii*, *M. polymorpha*, and *P. patens* (Figure 3E; Figures S3A–D, Supporting Information), contributing to the relatively larger genome size of *M. viride* within green algae. Calculated Kimura distances for LTR retroelements indicate a long term and increasing transposon activity in *M. viride* (Figure 3F), which is different from an apparent transposition burst pattern in *M. polymorpha*, *P. patens*, and *K. nitens* (Figure S3B–D, Supporting Information). Repetitive elements in *M. viride* represented 30.1% of the intron space, leading to the second longest intron length (1141.9 bp) after *C. braunii* among the selected species examined, including *Arabidopsis thaliana* and *C. reinhardtii* (Figure S3E–G and Table S1O, Supporting Information).^16^ The long intron size increased the average length of protein coding genes to 5940 bp in *M. viride* (Table 1), which is larger than many land plants.

### 2.4 Evolutionary Novelties of Multicellularity and Land Plant Heritage Genes

We further performed comparative analyses to understand the differential genetic basis of unicellular charophyte and chlorophyte algae, and to identify novel or ancestral traits and their associated genes during the evolution from unicellular to multicellular charophyte algae and from charophyte algae to land plants.

#### 2.4.1 The Split of Charophyta and Chlorophyta

The early split of green plants gave rise to charophyte and chlorophyte algae. This split is associated with major differences in morphological, physiological, and molecular characteristics. The underlying genetic basis was explored by comparative analysis of the genomes of charophyte algae, including *M. viride*, *K. nitens*, and *C. braunii*,[qv: 7a,d] with chlorophyte algae, including *C. reinhardtii*,^16^ *Volvox carteri*,^17^ *Ulva mutabilis*,[qv: 3b] *Chlorella variabilis*,^18^ *Coccomyxa subellipsoidea*,^19^ *Micromonas pusilla*, and *Ostreococcus tauri* (Figure S2C, Supporting Information).^20^ This revealed specific gene ontology (GO) terms for Charophyta and Chlorophyta (Table S2A, Supporting Information). Notably, the charophyte genomes were enriched for many GO terms relevant to land plants, such as “positive regulation of seed germination,” “root development,” “inflorescence development” and “stomatal complex morphogenesis,” all of which were absent in the chlorophyte genomes (Table S2A, Supporting Information). This is consistent with the characteristics of the multicellular land plants evolved from charophytes but not chlorophytes, suggesting that many important genes for land plant development were already present in unicellular charophyte green algae *M. viride*. This supports the hypothesis of exaptations in the evolution of Streptophyta.^21^ In addition, analysis of our RNA‐seq data for *M. viride* (Table S1D, Supporting Information) showed that expression of the genes with GO terms relevant to land plant development in *M. viride* were expressed and dynamically changed under different environmental conditions (Figure S2D, Supporting Information), suggesting that these genes are quickly responsive to external conditions.

#### 2.4.2 Cell Division and Cell Wall Synthesis

From unicellular charophyte algae to land plants, the mechanism of cell division has undergone several adjustments, including the evolution of cytokinetic phragmoplast and the preprophase band (PPB) of microtubules, while cell division in *M. viride* is an old model of centripetal cleavage.^22^ We compared the genes involved in cell wall synthesis, cell division and cell–cell communication among *M. viride*, *K. nitens*, *C. braunii*, *C. reinhardtii*, and *A. thaliana* (Table S2B, Supporting Information). As expected, gene sequences in unicellular chlorophyte *C. reinhardtii* are more divergent than those in the streptophyte species. Notably, since the homologs of many important genes involved in the function of PPB, phragmoplast and cell–cell communication were also found in *M. viride* (Table S2B, Supporting Information), these pre‐existing genes in unicellular *M. viride* may become co‐opted for new functions during evolution. For example, *DEFECTIVE KERNEL 1* (*DEK1*) is required for cell wall placement and three‐dimensional growth in *A. thaliana* and *P. patens*.^23^ The function of its homologs is evolutionarily conserved in land plants, but not in *M. viride*,[qv: 7c] indicating that DEK1 may have acquired new functions during the evolution of multicellularity or the origin of land plants. In contrast, although we found one cellulose synthase (CesA) and one cellulose synthase‐like gene in *M. viride* (Table S2B, Supporting Information),^24^ many genes related to cell wall synthesis, including glycosyltransferase family 8 (GT8), GT34, and GT47,^25^ were not found in *M. viride*, but existed in multicellular charophytes and land plants (Table S2B, Supporting Information). Interestingly, we found that biosynthesis and transport genes for a 2‐keto sugar acid, 3‐deoxy‐d‐manno‐2‐octulosonic acid (Kdo) (Data S1A–D, Supporting Information),^26^ which is a major component of scales,^27^ are present in *M. viride*. This is consistent with the observation on scales rather than cell wall covering of *M. viride*.

#### 2.4.3 Transcriptional Regulation

**Figure 4:**
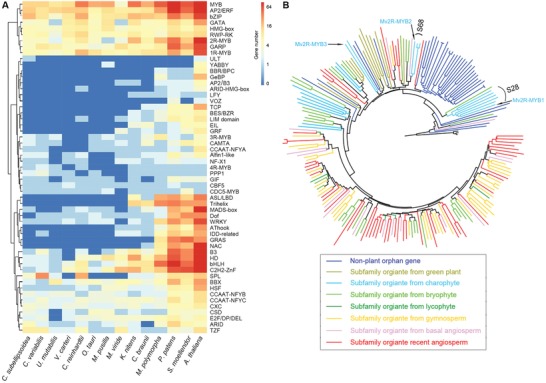
Transcription factors in *M. viride*. A) Heat map comparing the numbers of transcription factor genes in *M. viride* with those of representative land plants and green algae. The detailed information is shown in Table S2C in the Supporting Information. B) The R2R3‐MYB neighbor‐joining (NJ) phylogenetic tree includes representative sequences from previously identified 73 subfamilies and 95 nonplant orphan genes based on 50 eukaryotes,^30^ and all R2R3‐MYB proteins from *K. nitens*, *M. polymorpha*, and *M. viride* (Mv2R‐MYB1‐3).

Transcriptional regulation in plants has been extensively investigated in recent years.[qv: 7a,11b,28] We identified 123 putative transcription factors (TFs) encoded by the *M. viride* genome through blast and phylogenetic analyses. These TFs were classified into 31 families (**Figure** **4**A; Table S2C,D and Data S1E–M, Supporting Information). While most of these TFs (≈80%) were likely present in the last common ancestor of Viridiplantae, three TF families (AP2/B3, AThook, and GRF) could be specific to Streptophyta as they were absent in the chlorophytes (Table S2C, Supporting Information). Except for bZIP, C2H2‐ZnF, and GARP, all the other TF families in *M. viride* contained less than ten members. The number per TF family in *M. viride* was the smallest among all known genomes within Streptophyta (Table S2C, Supporting Information), possibly coinciding with its simplest morphological organization. TFs in *M. viride* accounted for 0.5% of the protein coding genes, the lowest percentage among all known species within Streptophyta, substantiating the observation that the TF number increases with organismal complexity.^12^, ^29^ Notably, TF datasets in *M. viride* allowed us to reveal their ancestral forms of land plant heritage TF genes in the evolutionary history (Figure 4B; Data S1E–M, Supporting Information). For example, R2R3‐MYB TFs represent one major family of regulatory factors in plants. Among three R2R3‐MYBs found in *M. viride*, two of them were classified as the members of the S28 and S68 subfamilies,^30^ respectively, while the third one did not belong to any existing subfamily (Figure 4B; Data S1E, Supporting Information). These findings suggest that both S28 and S68 subfamilies at least exist in the single‐celled charophyte alga, which sheds new light on the origin of S68 that was previously suggested to evolve from early diverging land plants.^30^

#### 2.4.4 Phytohormones

Phytohormones are signal molecules regulating cellular processes and are key hallmarks of multicellular plants. Several phytohormones, including auxin, abscisic acid (ABA), cytokinin, and jasmonic acid, have been detected in *K. nitens*, suggesting their early origins in charophyte algae.[qv: 7a,11b,31] However, because of low resolution of the previous transcriptome of *M. viride*,[qv: 31b] the genes coding for phytohormone biosynthesis and signaling pathways in *M. viride* were not unambiguously detected. Here, we found that there were almost no orthologs involved in biosynthesis of these phytohormones in the *M. viride* genome except for one ortholog of the ABA biosynthetic gene, *ABA1*, which encodes a zeaxanthin epoxidase that might be involved in the xanthophyll cycle (Table S2E and Data S1N–W, Supporting Information).^32^ Furthermore, we found a paucity of phytohormone‐related genes orthologous to those relevant to phytohormone transport, perception and signaling (Table S2E, Supporting Information). These observations argue against the presence of these phytohormones in *M. viride*. It is conceivable that those few hormone‐related genes identified in *M. viride* may serve different functions than their counterparts in multicellular plants, and were likely co‐opted for mediating phytohormone signaling during evolution.

#### 2.4.5 Epigenetic Regulation

**Figure 5:**
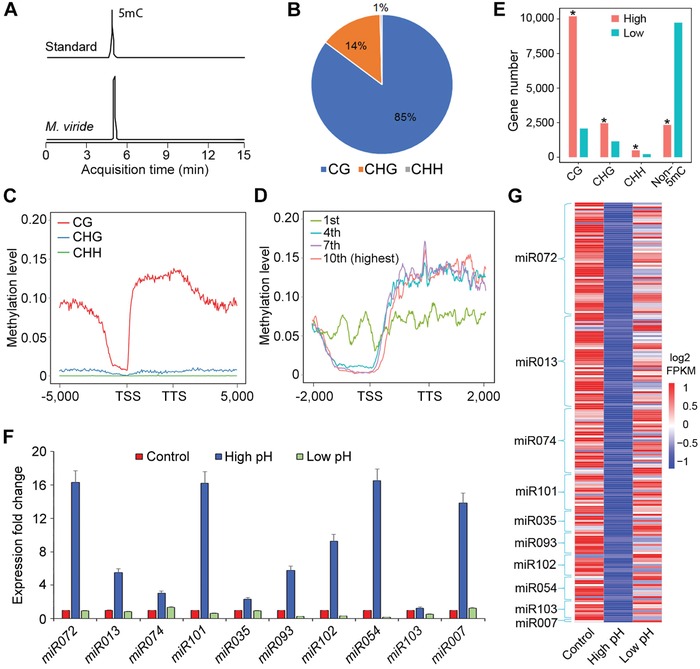
Epigenetic and miRNA regulation in *M. viride*. A) Ion chromatograms for 5mC nucleoside standard and 5mC nucleosides in genomic DNA purified from *M. viride*. B) Pie chart showing the composition of 5mC methylation motifs with CG as the major methylation site in *M. viride*. C) The average methylation levels of genes (including 5000 bp upstream of TSS and 5000 bp downstream of TTS) for each 100 bp interval plotted. D) Methylation levels of genes grouped into deciles based on expression levels (fragments per kilobase of transcript per million mapped reads, FPKM). The levels for four deciles (from the lowest first to the highest tenth) are shown. E) List of the numbers of 5mC‐methylated genes with high (FPKM ≥ 1) and low (FPKM < 1) expression levels. Asterisks indicate statistically significant differences in the numbers of methylated genes between highly and lowly expressed genes (Chi‐square test, *p* < 10^−5^). F) qPCR analysis of ten randomly selected pri‐miRNA in samples cultured under different pH conditions. Gene expression levels in the control are set as 1. Error bars, mean ± SD; *n* = 3 biological replicates. G) Heat map showing the expression of miRNA target genes extracted from the RNA‐seq data. Their expression negatively correlates with the expression of their corresponding pre‐miRNAs (F) under different pH conditions.

*M. viride* possessed homologs of many genes related to epigenetic processes so far identified in other eukaryotes (Table S2F and Data S1X–BA, Supporting Information). The transcription of some of these genes, such as those involved in DNA methylation, was dynamically altered in *M. viride* under different growth conditions (Table S3A–F, Supporting Information), implicating that epigenetic regulation of green plant responses to environmental changes is at least present in charophytes. DNA methylation is a reversible and dynamic epigenetic modification that regulates gene expression in eukaryotes. *M. viride* contains the orthologs of chromomethylase/DNA methyltransferase (CMT/DMT) genes (Data S1AP, Supporting Information).^33^ In agreement with this, our liquid chromatography‐tandem mass spectrometry (LC‐MS/MS) analysis detected the presence of 5‐methylcytosine (5mC) in the *M. viride* genome (**Figure** **5**A). To profile the genome‐wide 5mC sites, we applied bisulfite sequencing and found that 5mC was widely distributed in the *M. viride* genome (Figure S4, Supporting Information). CG was the most abundant site (Figure 5B) with the lowest and highest methylation levels detected at transcription start sites (TSSs) and transcription termination sites (TTSs), respectively (Figure 5C). Gene expression varied inversely with the promoter methylation, but correlated positively with the gene body methylation especially for genes with moderate to high expression levels (Figures 5D,E; Figure S4C, Supporting Information). This effect is partially consistent with that in land plants.^33^, ^34^

#### 2.4.6 Small RNA

The *M. viride* genome encodes several orthologs of Dicer‐like (DCL) and Argonaute (AGO) proteins (Data S1AQ,AU, Supporting Information) that are possibly required for miRNA processing.^35^ We also identified 116 pre‐miRNAs from small RNA sequencing data (Table S1J, Supporting Information). Their predicted target genes were associated with multiple biological processes according to GO analysis (Figure S5A, Supporting Information). Subsequent quantitative PCR analysis (qPCR) on ten randomly selected pre‐miRNAs and their target genes revealed that miRNAs were partly responsible for regulating transcript levels of their target genes in response to changes in growth conditions, such as pH, light intensity, and temperature (Figure 5F,G; Figure S5B,C, Supporting Information). Such a regulatory function of miRNAs for modulating gene transcription is likely an ancestral feature of streptophytes.

#### 2.4.7 RNA Methylation

Methylation of the N^6^ position of adenosine (m^6^A) is one of the most prevalent modifications on eukaryotic mRNA and plays a key regulatory role in development across several kingdoms of life. Strikingly, we did not identify any orthologs for components of the known m^6^A methylation machinery,^36^ which is in agreement with undetectable m^6^A signal in *M. viride* mRNA by LC‐MS/MS analysis (Figure S5D, Supporting Information). These results indicate that m^6^A modification at mRNA is dispensable for this unicellular taxon. It remains to be determined whether such a post‐transcriptional RNA modification is also absent from other charophyte algae and early diverging land plants, and whether acquisition of this additional layer of gene regulation was instrumental to colonization of the land by streptophytes.

#### 2.4.8 Sexual Reproduction

In eukaryotes, sexual reproduction is believed to be an ancient feature accomplished by meiosis.^37^ Whether sexual reproduction present in *M. viride* is unknown. Thus, we searched the “meiosis detection toolkit,” including eight meiosis‐specific proteins SPO11, HOP1, HOP2, MND1, REC8, DMC1, MSH4, and MSH5, which represent the best markers for the presence of meiosis,^37^ and found that all of them are present in *M. viride* genome (Data S1BB–BH, Supporting Information). This indicates that sexual reproduction may exist at the earliest diverging lineage of Streptophyta.

### 2.5 Stress Response to Environmental Conditions

**Figure 6:**
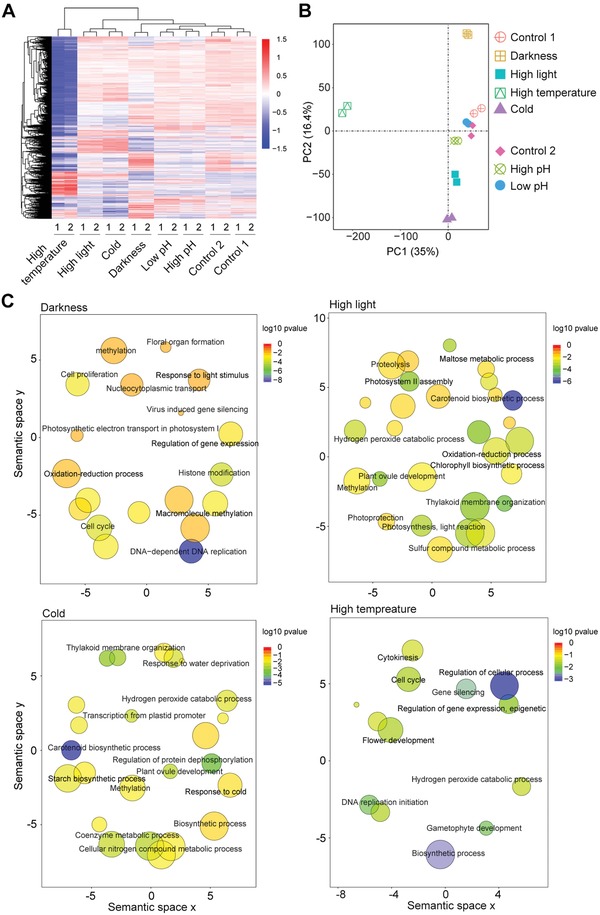
Transcriptome profiles of *M. viride* cultured under different environmental conditions. A) Heat map showing differentially expressed genes (*p* < 0.01, fold change > 2) under different light intensity, temperature and pH compared to optimal growth conditions as indicated in Methods. Two biological replicates were included for each treatment. Samples cultured under different light intensity and temperature conditions were compared with Control 1, while those cultured under different pH conditions were compared with Control 2. B) Principal component analysis of RNA‐seq data derived from samples cultured under different conditions. Axis percentages indicate variance contribution. C) Scatter plots of significant biological processes as determined by GO enrichment analysis of differentially expressed genes (DEGs) under different light intensity and temperature. The size of the circle is proportional to the number of DEGs.

We further performed transcriptome profiling of *M. viride* cultured under different environmental conditions to examine changes in transcripts in response to various stresses, including high temperature, cold, high light, darkness, high pH, and low pH (**Figure** **6**; Figure S6 and Tables S1D and S3A–F, Supporting Information). Notably, the greatest perturbation in transcriptome was observed under high temperature as 30% of the *M. viride* transcripts were differentially expressed (Table S3D, Supporting Information). The differentially expressed genes included those homologous to genes involved in redox regulation, protein chaperoning and repair, DNA damage sensing and repair, and metabolisms of maltose, sulfur and coenzyme (Figure 6; Table S3A–F, Supporting Information). Thus, *M. viride* exhibits typical cellular stress responses that are conserved in all organisms.^38^ Some of the hallmark genes for stress responses in land plants, including early light induced proteins, late embryogenesis proteins and ABA receptor proteins, have been reported to be upregulated in *K. nitens* and higher branching charophyte algae under stress.^39^ However, none of these homologs was found in *M. viride*, implying that some typical stress responses known to be present in land plants and some charophytes (e.g., *K. nitens*) may not occur in *M. viride*.

As expected, some of the differentially expressed genes under different light conditions belong to functional groups that are potentially involved in photosynthesis, Photosystem II assembly, chlorophyll biosynthetic process and thylakoid membrane organization (Figure 6C). The genes, which are essential for photosynthesis in land plants, are present in the *M. viride* genome (Table S2G and Data S1BI–BV, Supporting Information). We could also detect the expression of major proteins involved in the light‐dependent photosynthetic activity of *M. viride* (Figure S7A, Supporting Information). These observations, together with analysis of photosynthesis in *M. viride* (Figures S7B,C, Supporting Information), infer that the common photosynthesis systems in land plants were established at the base of Streptophyta. We further compared the photosynthesis systems between the unicellular chlorophyte algae *C. reinhardtii* and *M. viride*, and found that the photosynthesis activities of PSII and PSI were much lower in *M. viride* than in *C. reinhardtii* (Figures S7B,C, Supporting Information). *C. reinhardtii* exhibits an efficient photosynthetic capacity through carbon concentrating mechanisms (CCMs), which requires the activity of carbonic anhydrases (CAs) and StArch Granules Abnormal 1 (SAGA1).^40^ Notably, although we found 14 CA genes in *M. viride*, *SAGA1* was absent, implying that CCM may not be functional in *M. viride*. This may partly explain the low photosynthetic efficiency in *M. viride* versus *C. reinhardtii*.

## 3 Conclusion

In this study, we report the high‐quality genome of *M. viride*, which is the first sequenced unicellular genome in the Streptophyta division, which include land plants that colonized and transformed the terrestrial habitat of our planet. Comparative analysis of charophyte and chlorophyte genomes sheds light on the genetic variations underlying the early split of green plants, and indicates that evolution of Charophyta is associated with some genetic innovations relevant to multicellular land plant development.

Systematic comparisons of the genome and transcriptome of *M. viride* with those of other multicellular charophyte algae and land plants within Streptophyta enable us to investigate the hitherto unknown genetic basis of multicellularity in Streptophyta and the origin of land plants. On the one hand, we have identified the common genetic tools in *M. viride*, which are inherited by multicellular charophyte algae and land plants, such as those associated with cell division, cell–cell communication, DNA methylation, small RNA, transcriptional regulation of gene expression, sexual reproduction, and photosynthesis. On the other hand, our analysis has also revealed genetic innovations that are relevant to the evolution of multicellularity and land plants from unicellular charophyte algae, such as cell wall synthesis, phytohormones, RNA methylation, and stress response to environmental conditions. Taken together, our findings are essential to clear understanding of the genetic characteristics of the earliest diverging lineage of Streptophyta, and provide novel insights into the evolution of multicellularity and the origin of land plants.

## 4 Experimental Section

*Plant Materials: M. viride* strain NIES‐296 was obtained from the Microbial Culture Collection at the National Institute for Environmental Studies (NIES Collection, Japan). The cells were cultivated under optimal growth conditions in medium C (pH 7.5) in 250 mL Erlenmeyer flasks with gentle agitation at 23 °C under the light‐dark cycle of 10/14 h with light intensity of 50 µmol photons m^−2^ s^−1^.^41^ Prior to each experiment, the cultivated cells were checked under microscope to exclude potential external bacterial contamination. For RNA‐seq experiments, 24 day old cultured cells were subjected to different environmental conditions. The untreated cells were used as Control 1. To test the effects of light intensity, *M. viride* cells were cultured in darkness and under light intensity of 400 µmol photons m^−2^ s^−1^ for 24 h, respectively. To test the effects of different temperature conditions, *M. viride* cells were grown at 12 and 32 °C for 24 h, respectively. To test the effects of different pH conditions, *M. viride* cells were grown at pH 9.0 (adjusted with NaOH) and pH 6.0 (adjusted with Tris‐HCl) for 24 h, respectively. The untreated cells with the same volume of medium C (pH 7.5) were used as Control 2.

*Scanning Electron Microscopy (SEM): M. viride* cells were fixed for 1 h at room temperature in 0.1 m PBS (pH 7.2) containing 2% (v/v) glutaraldehyde and 1% (w/v) formaldehyde followed by gentle washing in 0.1 m PBS (pH 7.2) for 3–4 times. Cells were then dehydrated in a graded series of ethanol followed by incubation in isopropanol for 15–20 min and subsequently dried by the critical point method.^42^ All centrifugation steps for pelleting the cells were done at room temperature and at 7500 rpm for 7 min. Cells were finally coated with gold:palladium alloy (60:40) before being observed under a scanning electron microscope (Quanta 200, FEI Company, USA) at accelerating voltages of 6−10 kV.

*Transmission Electron Microscopy (TEM): M. viride* cells were fixed for 1 h at room temperature in 0.1 m PBS (pH 7.2) containing 2% (v/v) glutaraldehyde and 1% (w/v) formaldehyde followed by gentle washing in 0.1 m PBS (pH 7.2) for 3–4 times. Cells were postfixed with 1% osmium for 2 h followed by two washes in 0.1 m PBS (pH 7.2) and then dehydrated in a graded series of ethanol. The cells were then infiltrated in 50%, 66.7% and 75% embedding medium in acetone (1 h each at room temperature) and left in 75% embedding medium overnight at 4 °C. The sample was transferred into pure embedding medium on the next day and left overnight at 4 °C followed by curing at 37, 45, and 60 °C for 24 h each. The embedded sample was then sectioned into ultrathin sections of 70 nm thickness and stained following the double contrast method before being observed under a transmission electron microscope (JEM‐1400, EDL Company, Japan).

*Flow Cytometry: M. viride* cells were fixed for 1 h at room temperature in 4% (v/v) formaldehyde buffer followed by two washes in PBS (pH 7.2). The cells were incubated in 5% (w/v) EDTA for 3 h and then washed twice in PBS. The nuclei were stained using 10 µg mL^−1^ DAPI (4′,6‐diamidino‐2‐phenylindole) in PBS under dark conditions at 4 °C for 15 min before the cells were resuspended in 300 µl PBS for flow cytometry analysis (LSR Fortessa, BD Company, USA). The data were processed using FlowJo Version 7.0.

*Chromosome Number Analysis: M. viride* cells were treated with 0.02% colchicine and fixed with Farmer's fixative [anhydrous ethanol:glacial acetic acid = 3:1 (v/v)] for 24 h. The cells were then pelleted and dissociated by 1 m hydrochloric acid (HCl) for 7 min at 60 °C. The sample was stained with 5 µg mL^−1^ DAPI solution for 5 s and observed under a fluorescence microscope (AXIO IMAGER Z2).

*Library Preparation and Genome Sequencing: M. viride* cells were lysed in lysis buffer (50 × 10^−3^ m, Tris‐HCl pH 8.0, 200 × 10^−3^ m NaCl, 20 × 10^−3^ m EDTA, 2% SDS, 1% PVP4000, 1 mg mL^−1^ proteinase K). Genomic DNA for library construction was extracted using DNeasy Plant Mini Kit (QIAGEN). DNA concentrations and quality were measured using NanoDrop 2000 (Thermo) and Qbit Fluorometer (Thermo Fisher), respectively. Library preparation and quality assessment for Illumina X Ten PCR‐free paired‐end genome sequencing were performed according to the manufacturer's protocol (Illumina, USA). Genomic DNA was fragmented and size‐selected through agarose gel electrophoresis. The ends of selected DNA fragments were blunted with an A‐base overhang and ligated to sequencing adapters. After quality control by Agilent 2100 Bioanalyzer and qPCR, all PCR‐free libraries were sequenced on an Illumina X Ten platform with 150 bp paired‐end sequencing strategy. A total of 70.04 Gb paired end reads were obtained for genome survey and PacBio SMRT genome polishing. The 20 kb libraries for SMRT genome sequencing were constructed according to the protocol of the SMRT RSII platform (Pacific Biosciences). Sequencing was performed on 65 PacBio RSII cells with P6/C4 chemistry. The minimum subread length (50 bp) and RQ value (0.75) were adopted for data quality control. A total of 48.6 Gb PacBio high‐quality subreads, accounting for 113‐fold genome coverage, were obtained for the genome assembly. The subread length N50 of final clean data is 11.2 kb.

*Analysis of Bacterial Contamination*: To assess the potential contamination, 40 000 randomly selected paired‐end sequences of each short‐read library were mapped to NT database with bwa‐mem of BWA v0.7.10. More than 90% of short reads were supported by *M. viride* sequences in NT database, suggesting that the samples in this study were reliable. Short reads and translated protein sequences were used to map bacteria or virus NT/NR database. The mapped (*E* value < 10e−1) bacteria or virus were considered as potential contamination sources. Scaffolds/contigs having more than 1 kb contiguous matches with >85% sequence identity to contamination sources were probably contaminated and were filtered out for further analysis.

*Preliminary Contig Assembly of PacBio SMRT Reads*: After quality control, self‐correction of subreads was achieved using error correction model of Falcon package v1.8.7.^43^ Canu (v1.5) assembler was used for the de novo assembly of the PacBio single molecule sequencing data.^44^ Canu was selected because of its best capacity to perform error correction for PacBio sequences. To polish the assembly results, Pilon (v1.2) with default parameters (http://www.broadinstitute.org/software/pilon/) was used with 70.04 Gb Illumina short reads.^45^ Pilon corrects single nucleotide differences, small insertion/deletion events, misassemblies and gaps.

*Construction of BioNano Optical Map*: To develop a robust physical map for *M. viride* that could be helpful to place sequence contigs and determine the physical length of gaps between them,^46^ BioNano optical genome map libraries were constructed. Based on the enzyme density and distribution assessment of genome sequences by Label Density Calculator v1.3.0 (BioNano Genomics), Nt.BspQI nickase was used for the optical map library construction. The basic process of acquiring BioNano raw data was done using IrysView v2.5.1 package (BioNano Genomics). Molecules with the length more than 150 kb (with the label SNR >3.0 and the average molecule intensity <0.6) were retained for further construction of the genome map. 87.9 Gb high‐quality optical molecules were obtained, accounting for ≈203.6‐fold genome coverage. The N50 of the molecules is 229 kb. Based on the labeled positions on single DNA molecules, de novo assembly was performed by a pairwise comparison of all single molecules and overlap‐layout‐consensus path building, as adopted by IrysView v2.5.1 assembler (https://bionanogenomics.com/support/software-downloads/). Only the molecules containing more than eight nicking enzyme sites and longer than 150 kb for assembly were considered. A *p* value threshold of 1e−8 was used during the pairwise assembly, and 1e−9 for extension and refinement steps and 1e−11 for merging contigs. The high‐quality optical map facilitated the subsequent genome curation and hybrid assembly.

*Hybrid Assembly for Building Superscaffolds*: The assembly results from SMRT reads may introduce chimeric errors from homologous and/or large repeat regions of *M. viride*. The BioNano optical map of single molecules could assemble large‐sized homologous and repeat regions, taking advantage of its superlong reads. Thus, it is necessary and feasible to detect conflicts between contigs and the genome map, and to correct potential errors. To ensure the quality of assembly results, an in silico map of merged results was generated by the Knickers v1.5.5.0 program (https://bionanogenomics.com/support/software-downloads/) with Nt.BspQI nickase. The conflicts were identified in the comparison between the contigs and genome map by RefAligner v5122 (https://bionanogenomics.com/support/software-downloads/), and resolved using next generation mapping (NGM‐HS) by breaking the conflict points of assembly. Briefly, conflicts were identified based on a chimeric score of a conflict junction and the SMRT molecule alignment result, which is near the conflict junction on the optical genome map. The chimeric score of the conflict junction is defined as the percentage of BioNano molecules that were fully aligned to the 50 kb flanking regions of the optical map. If the chimeric score of the conflict junction was ≥30 with more than two fully aligned optical molecules located across the conflict junction of the genome map, a candidate chimerical error was assigned in the contig sequence. The alignment results of conflict regions were visualized in IrysView for manual inspection. Knickers, RefAligner, and IrysView were obtained from BioNano Genomics. The consistent soft‐clip sites of SMRT molecules on the reference sequence as an accurate break point was considered. All proposed cuts were manually evaluated using BioNano molecule‐to‐genome map alignments, and SMRT molecule‐to‐sequence contig alignments based on the integrated graphic platform. After chimeric correction, the hybrid assembly of PacBio contigs and the BioNano optical map was carried out using BioNano IrysSolve module “HybridScaffold.” The corrected BioNano map was aligned again to the contig map, and superscaffolds were built according to the syntenic relationship of optical labels between PacBio contigs and the BioNano genome map.

*Gap Filling and SMRT‐Error Correction*: SMRT sequencing data and Illumina data were also combined to fill gaps so as to improve the contiguity of the assembly results. PBJelly v14.9.9 was used to fill gaps in error‐corrected SMRT sequencing data from the initial contig assembly step.^47^ The remaining gaps were subsequently filled using Illumina paired‐end sequencing data (162‐fold coverage) with Gapcloser v1.12 in SOAPDenovo packages_015026.^48^ The consensus sequences for superscaffolds were further polished based on Illumina paired‐end reads using Pilon.^45^

*Genome Completeness Assessment*: Genome completeness was assessed using Benchmarking Universal Single‐Copy Orthologs (BUSCO) plant database with *E* value < 1e−5.^10^ It detected 90.1% complete and 5.0% fragmented BUSCO gene models in the assembly. The RNA‐seq data of transcriptomes and PacBio subreads were also remapped to the assembly results. For RNA‐seq data, paired‐end reads were aligned by bwa‐mem of BWA v0.7.10,^49^ and it was found that most of the transcriptome data could be correctly remapped to the consensus sequences. The error‐corrected PacBio data were also successfully remapped to the assembly results by blastr with default parameters.^50^

*Repeat Sequence Annotation*: Both homolog‐based and de novo strategies were applied to identify repetitive sequences of the *M. viride* genome. Five de novo prediction software, including RepeatScout,^51^ LTR‐FINDER,^52^ MITE‐Hunter,^53^ PILER‐DF, and RepeatModeler,^54^ were adopted for ab initio prediction. RepeatScout identified all repeat classes, while LTR‐FINDER predicted the location and structure of full‐length LTR retrotransposons. MITE‐Hunter discovered miniature inverted‐repeat transposable elements (MITEs) from genomic sequence, while PILER‐DF found repeated elements, such as satellites and transposons. Results from ab initio prediction were combined to construct a library of repetitive sequences. This library was then merged with Repbase,^55^ and classified into different categories by the PASTEClassifier.py script of REPET.^56^ The repetitive sequences of the *M. viride* genome were then identified by homolog searching against this newly created database through RepeatMasker.^57^

*LncRNA Sequencing and Analysis*: Total RNA was extracted using RNeasy Plus Mini Kit (Life Technologies). The sample was subjected to poly(A) purification using oligo‐dT beads (Life Technologies) followed by rRNA removal using Ribo‐Zero Kit (Epicenter). RNA integrity was measured by 2100 RNA Nano 6000 Assay Kit (Agilent Technologies). The resulting RNA sample was then used for library construction using the dUTP method as described.^58^ The library was sequenced on Illumina HiSeqX Ten system, producing 150 bp paired‐end reads. The transcriptome was assembled using StringTie v1.34d by mapping the reads to the reference genome using HiSAT2.^59^ The assembled transcripts of three biological replicates were merged by StringTie merge command, and compared to the gff format file of annotation results to identify any novel lncRNA candidates using gffcompare v0.10.4 program (http://ccb.jhu.edu/software/stringtie/gff.shtml). Unknown transcripts were screened for putative lncRNAs. Class code attributions of “u,” “I,” and “x” represented candidate lncRNA, intronic lncRNA and anti‐sense lncRNA, respectively. Transcripts with length more than 200 bp and containing more than two exons were considered as lncRNA candidates. Four computational approaches, including CPC v1,^60^ CNCI v2,^61^ Pfam, and CPAT v1.2,^62^ were combined to sort nonprotein‐coding RNA candidates into the above unknown transcripts. The transcripts with potential protein coding capability as identified by any one of the above approaches were discarded.

*Small RNA Sequencing and Analysis*: Small RNA was extracted from three independent biological replicates using mirPremier microRNA Isolation Kit (Sigma‐Aldrich). Subsequent small‐RNA libraries were constructed using NEBnext small‐RNA Library Kit (NEB). Raw reads (50 bp single‐end read) from Illumina HiSeqX Ten system were trimmed to remove 3′‐adapters and filtered for quality using cutadapt v1.9.1. This gave rise to small RNAs with trimmed length of ≥16 nucleotides. Any sequences less than 18 nts or longer than 30 nts were filtered out. The clean reads were mapped to several databases (Silva, GtRNAdb database, Rfam and Repbase) to remove rRNAs, tRNAs, small nuclear RNAs (snRNAs), small nucleolar RNAs (snoRNAs), and other ncRNAs and repeats. The remaining reads were aligned to miRBase using “blastall‐p blastn” against reference miRNAs in database to annotate miRNAs.^63^ These reads were subsequently aligned with zero mismatch using bowtie v1.1.2 (setting “‐v 0”) to the *M. viride* genome.^64^ The miRNA target genes were identified using targetfinder v1.6.package with parameter of “‐c 5.”

*Full‐Length Isoform Sequencing and Analysis*: To eliminate residual genomic DNA contamination, RNA samples were treated with Turbo DNase and cleaned up using RNeasy MinElute Cleanup Kit. PacBio SMRT libraries were prepared from integrated and normalized cDNAs, and sequenced on PacBio RSII system, which generated an average of 4–6 kb read length with 3–20 kb library preparations. cDNAs were amplified using five cycles of PCR, and four size fractions (<1, 1–2, 2–3, and >3 kb) were excised from a 0.8% agarose gel. These fractions were purified using Zymoclean Large Fragment DNA Recovery Kit (Zymo). To minimize the over‐representation of abundant transcripts and reduce the required number of SMRT Cells for unique transcript identification, cDNAs were normalized using the Trimmer‐2 cDNA normalization kit (Evrogen JSC). To reduce short‐read bias, cDNAs from ten cycles of PCR were loaded onto a 0.75% cassette (Sage) and cDNAs <1 kb were selected on a Sage Science Electrophoretic Lateral Fractionator (ELF). cDNA fractions >3 kb were collected for additional ten PCR cycles for enrichment of long reads. These cDNA fractions were then treated with the DNA damage repair mix followed by end repair and ligation of SMRT adapters using the PacBio SMRTbell Template Prep Kit to create PacBio libraries, which were sequenced on the PacBio RSII platform. A total of 10.5 million reads (25 Gb) were obtained for gene prediction and lncRNA identification.

Raw reads were processed into error‐corrected reads of insert (ROIs) using Iso‐seq pipeline with minFullPass = 0 and minPredictedAccuracy = 0.80. The ROIs were further classified into circular consensus sequences (CCS) and non‐CCS subreads by ToFu v 2.3.0 based on the presence and absence of sequencing adapters.^65^ Full‐length nonchimeric (FLNC) transcripts were determined by simultaneous detection of both primer sequences and the polyA tail signals in ROIs. A clustering algorithm, ICE (Iterative Clustering for Error Correction), was then used to obtain consensus sequences for all full‐length transcripts, which were further grouped into clusters based on sequence similarity. Quiver (PacBio) was used to polish the consensus sequences to generate high‐quality full‐length transcripts with more than 99% postcorrection accuracy.

*LncRNA Identification from Isoform Sequencing*: Four computational approaches (CPC, CNCI, CPAT, and Pfam) were combined to identify nonprotein coding RNA candidates from putative protein coding RNAs in the transcripts. Transcripts with the length more than 200 nt and containing more than two exons were selected as lncRNA candidates. These candidates were further distinguished using CPC/CNCI/CPAT/Pfam for potential protein coding assessment. Only candidates identified as strong noncoding RNAs were assigned as confident lncRNAs.

*Gene Model Prediction and Annotation*: Gene annotation was performed using a combination of three methods, including ab initio prediction, homology‐based gene prediction, and transcript evidence from RNA‐seq data. Two ab initio prediction tools, Genscan and Augustus v2.4,^66^ were used for de novo annotation. GeneWise v1.3.1 was employed for homology‐based gene prediction using model training based on coding sequences of *C. reinhardtii*, *K. nitens*, *P. patens*, *A. thaliana, Z. mays*, and *O. sativa*. The isoform transcriptome data generated from different culture conditions were used to predict genes using PASA v2.0.2.^67^ Finally, the gene model sets were integrated from the above three methods through EVM v1.1.1 tool. All gene models were annotated using BLASTP of blast+ package v2.2.6 (*E* value = 1e−5) according to the best match of the alignment against the protein databases,^68^ including GO,^69^ KEGG,^70^ Swiss‐Prot,^71^ TrEMBL, and nonredundant protein database (NR).

*Noncoding RNA Annotation*: Two strategies were considered for noncoding RNA annotation in the *M. viride* genome, including de novo prediction and direct ncRNA sequencing of small RNAs and lncRNAs. The tRNAscan‐SE v1.23 was applied to detect reliable tRNAs through two embedded searching methods (tRNA‐scan and EufindtRNA).^72^ miRNAs were identified using miRBase (Release 21) as a reference by homolog searching with one mismatch.^73^ The secondary structure of the putative sequences was predicted by miRDeep2.^74^ Putative miRNAs with hairpin structure were considered as confident miRNAs. Other types of noncoding RNAs were predicted by Infernal (*E* value < 0.01).^75^ By comparing the secondary structure between *M. viride* genome sequences and Rfam (v12.0) database,^75^ the ncRNAs were classified into respective families. Genome‐wide ncRNAs were also inspected through lncRNA‐seq and small RNA‐seq, with two biological replicates. In total, six types of ncRNAs were identified, including tRNAs, rRNAs, miRNAs, snRNAs and snoRNAs, and lncRNAs. A total of 2540 ncRNAs were annotated, which accounted for 461 312 bp of the *M. viride* genome.

*Pseudogene Identification*: Four protein datasets from *K. niten*, *M. polymorpha*, *C. reinhardtii*, and *M. viride* were aligned to the *M. viride* reference genome assembled in this study with tblastp for identification of candidate homologous regions.^68^ The candidate pseudogenes were identified through GeneWise.^76^ Only candidate pseudogenes with frame shift and/or premature stop codon were considered as confident pseudogenes. After redundant filtering and manual inspection, a total of 7570 confident pseudogenes were annotated for *M. viride*.

*Gene Synteny Inspection*: To identify the internal synteny blocks of the genome, virtually translated protein sequences of *M. viride* were aligned to each other using blastp with *E* value < 1e−5.^68^ Synteny blocks were then called using McScanX and those with at least five gene pairs were retained.^77^ If multiple alignments were found, the longest synteny block was kept.

*Identification of Segmental Duplication*: All‐by‐all synteny and Ks comparisons were made among *M. viride, C. reinhardtii*, and *M. polymorpha*. Synteny blocks (regions with at least five collinear genes) were identified within these species using MCScanX with default parameters.^77^ Ks values of paralogous gene pairs originating from segmental duplication were calculated using the yn00 method from the PAML package.^78^ The peaks of Ks distribution derived from internal homolog gene pairs of three species were used to reconstruct the history of segmental duplication or tandem duplication.

*Gene Expression Quantification*: Total RNA for two biological replicates was extracted from *M. viride* cultured under different conditions using RNeasy Plus Mini kit (QIAGEN). Subsequent mRNA purification and cDNA library construction were performed using TruSeq Stranded mRNA Library Prep Kit (Illumina). Sixteen paired‐end libraries were constructed and sequenced according to the Illumina HiSeq platform sequencing protocols. After removing the adapter and primer sequences, low‐quality reads with more than 20% low quality bases (quality < 20) were filtered out using FastQC packages (http://www.bioinformatics.babraham.ac.uk/projects/fastqc). Clean reads were mapped to the *M. viride* genome using Hisat2 v2.1.0.[qv: 59b] Gene expression levels (FPKM) were calculated using Cufflinks with default parameters.^79^ Differentially expressed genes (DEGs) were identified by DEseq2 package,^80^ and only transcripts with fold change ≥2 and FDR ≤0.01 were considered as DEGs.

*Examination of Phylogenetic Relationships among Plant Species*: Protein‐coding genes of *C. reinhardtii* (http://plants.ensembl.org/Chlamydomonas_reinhardtii/Info/Index), *V. carteri* (https://bioinformatics.psb.ugent.be/plaza/versions/plaza_v2_5/download/index), *U. mutabilis* (https://bioinformatics.psb.ugent.be/orcae/overview/Ulvmu), *C. variabilis* (https://mycocosm.jgi.doe.gov/ChlNC64A_1/ChlNC64A_1.home.html), *C. subellipsoidea* (https://mycocosm.jgi.doe.gov/Coc_C169_1/Coc_C169_1.home.html), *M. pusilla* (https://phytozome.jgi.doe.gov/pz/portal.html#!info?alias=Org_MpusillaCCMP1545), *O. tauri* (https://genome.jgi.doe.gov/Ostta4221_3/Ostta4221_3.home.html), *K. nitens* (http://www.plantmorphogenesis.bio.titech.ac.jp/~algae_genome_project/klebsormidium/kf_download.htm), *C. braunii* (https://bioinformatics.psb.ugent.be/orcae/overview/Chbra), *M. polymorpha* (http://marchantia.info/download/), *P. patens* (https://plants.ensembl.org/Physcomitrella_patens/Info/Index), *S. moellendorffii* (http://plants.ensembl.org/Selaginella_moellendorffii/Info/Index), *P. abies* (http://ftp://plantgenie.org/Data/ConGenIE/Picea_abies/v1.0/), *A. thaliana* (http://plants.ensembl.org/Arabidopsis_thaliana/Info/Index), *O. sativa* (https://plants.ensembl.org/Oryza_sativa/Info/Index), *P. trichocarpa* (http://plants.ensembl.org/Populus_trichocarpa/Info/Index), *G. max* (http://plants.ensembl.org/Glycine_max/Info/Index), and *Z. mays* (http://plants.ensembl.org/Zea_mays/Info/Index) were downloaded from the respective websites. The longest transcript was used to represent a gene. After clustering protein sequences using OrthoMCL v2.0.9 with default parameters,^81^ single copy orthologs were identified from one‐copy families of selected species. A total of 247 single‐copy orthologs were obtained for further analysis. The protein sequences of single‐copy orthologs were aligned by mafft v7.058,^82^ and low‐quality alignment regions were removed by Gblocks v0.91b^83^ using default parameters. Phylogenetic relationships among plant species were then examined using the maximum likelihood (ML) algorithm with the model GTRGAMMA of nucleotide substitution implemented in RAxML v8.0.19 software (‐m GTRGAMMA ‐p 12345 ‐b 12345).^84^ The divergence time was estimated using the MCMCtree program in the PAML (Phylogenetic Analysis of ML) package.^78^ Six calibration points (*Z. mays* vs *O. sativa*: 40–53 MYA; *A. thaliana* vs *P. trichocarpa*: 97–109 MYA; *P. abies* vs *P. trichocarpa*: 289–337 MYA; *M. polymorpha* vs *P. patens*: 425–557 MYA; *K. nitens* vs *P. patens*: 481–584 MYA; *C. reinhardtii* vs *K. nitens*: 773–1174 MYA) were derived from the TimeTree database (http://www.timetree.org/) and applied to constrain the divergence time of the nodes.^85^

*Gene Family Evolution Analysis*: To define gene families that descended from a single gene in the last common ancestor, OrthoMCL v2.0.9,^81^ which implemented the Markov Cluster (MCL) algorithm, was used to perform gene family clustering analysis. All‐against‐all BLASTP comparisons of the proteins were performed using a *p* value cutoff of 1e−5. The resulting pairs were grouped based on their relationships using the MCL program of the OrthoMCL package. The gene families generated from OrthoMCL were sorted into groups based on the following clades (**Table** **2**):

**Table 2:** Groups for gene family evolution analysis

Common ancestor	Present in at least four chlorophyte species, *M. viride*, *K. nitens*, *C.braunii*, *P. patens*, *M. polymorpha*, *S. moellendorffii* and at least two angiosperm species. Present in both a streptophyte species and a chlorophyte species
Angiosperm –	Absent in all angiosperms but present in at least three of the followings: four chlorophyte species, *M. viride, K. nitens*, *C.braunii*, *P. patens*, *M. polymorpha*, and *S. moellendorffii*
Angiosperm +	Present only in at least three angiosperm species
*S. moellendorffii* −	Present in *M. viride*, *K. nitens*, *C. braunii*, *P. patens*, and *M. polymorpha*, and at least two angiosperm species, but not in *S. moellendorffii*
*S. moellendorffii* +	Present in *S. moellendorffii* and at least two angiosperm species, but not in *M. viride*, *K. nitens*, *C. braunii*, *P. patens*, and *M. polymorpha*
Early diverging land plant −	Present in *S. moellendorffii*, at least two angiosperm species, *M. viride*, *K. nitens*, and *C. braunii*, but not in *P. patens* and *M. polymorpha*
Early diverging land plant +	Present in *P. patens* and *M. polymorpha*, and at least two angiosperm species, but not in *M. viride*, *K. nitens*, and *C. braunii*
*C. braunii* −	Present in *M. viride*, *K. nitens*, *P. patens*, *M. polymorpha*, and *S. moellendorffii* and at least two angiosperm species, but not in *C.braunii*
*C. braunii +*	Present in *P. patens*, *M. polymorpha*, and *C. braunii* and at least two angiosperm species, but not in *M. viride* and *K. nitens*
*K. nitens* −	Present in *M. viride*, *C.braunii*, *P. patens*, *M. polymorpha*, and *S. moellendorffii*, and at least two angiosperm species, but not in *K. nitens*
*K. nitens* +	Present in *K. nitens*, *C.braunii*, *P. patens*, *M. polymorpha*, *S. moellendorffii*, and at least two angiosperm species, but not in *M. viride*
*M. viride* +	Present in *M. viride*, *K. nitens*, *C. braunii*, *P. patens*, *M. polymorpha*, and *S. moellendorffii*, and at least two angiosperm species, but not in common ancestor
Chlorophyte +	Present in *C. reinhardtii* or *V. carteri* or *U. mutabilis*, *C. subellipsoidea* or *C. variabilis*, and *M. pusilla* or *O. tauri*, but not in common ancestor

*Phylogenetic Analysis of Gene Families*: To explore the origin and evolutionary relationship of gene families in plants, a BLASTP search was performed using well‐studied proteins (mostly from *A. thaliana* and *O. sativa*) as queries with 11 selected plants that have available genome sequences. They are from chlorophyte (*V. carteri* and *C. reinhardtii*), charophyte (*M. viride* and *K. nitens*), bryophyte (*P. patens* and *M. polymorpha*), lycophyte (*S. moellendorffii*), gymnosperm (*P. abies*), basal angiosperm (*A. trichopoda*), monocot (*O. sativa*), and eudicot (*A. thaliana*). To ensure that no protein was eliminated by lack of correspondence to the consensus sequence, a low‐stringency criterion (*p* value cutoff < 1e−1) was used. Following the deletion of redundant sequences, candidates were examined for the typical domain(s) of respective gene families using SMART tool (http://smart.embl-heidelberg.de/), and the sequence(s) without the typical domain(s) were filtered out. The same procedure and criteria were applied for all species and gene families. Multiple alignments of candidate proteins were performed using MAFFT version 7 software with default parameters.^82^ The alignments were then manually inspected using MEGA 7 software.^86^ Further analysis only included unambiguously aligned positions. A neighbor‐joining (NJ) tree was constructed using MEGA 7 software based on the alignment of candidate proteins.^86^ To determine the statistical reliability, bootstrap analysis was conducted for 1000 replicates with the following parameters: p‐distance and pairwise deletion.

*LC‐MS/MS Analysis of DNA and mRNA Methylation Levels*: LC‐MS/MS was performed as previously described.^87^ Briefly, DNA or mRNA samples were digested into single nucleosides or ribonucleosides, respectively. Individual nucleosides and ribonucleosides were resolved on a Hypersil GOLD aQ reverse phase column (Thermo Scientific), and the samples were then subjected to LC‐MS/MS analysis on an Agilent 6490 Triple Quadrupole mass spectrometer. Nucleosides were quantified using the nucleoside‐to‐base ion mass transitions of 242.1–126.1 for 5mC and 228.1–112.1 for C. Ribonucleosides were quantified using the nucleoside‐to‐base ion mass transitions of 258.1–126.1 for m^5^C, 244–112 for C, 282.1–150.1 for m^6^A, and 267.9–136.1 for A.

*Whole‐Genome Bisulfite Sequencing (WGBS): M. viride* DNA was sheared to ≈250 bp fragments by sonication using a Bioruptor (Diagenode). The fragments were end‐repaired, A‐tailed and ligated to methylated Illumina adapters (Illumina) using KAPA's Illumina Library Creation Kit (KAPA Biosystems). The adaptor‐ligated DNA was bisulfite‐treated using EZ DNA Methylation Lightning Kit (Zymo Research), converting the nonmethylated nucleotides from cytosine to uracil. Lambda‐phage genomic DNA was used as a negative control to determine the efficiency of the sodium bisulfite conversion reaction. Products were purified using QIAquick Gel Extraction Kit (Qiagen) and amplified with ten cycles of PCR. DNA libraries were constructed according to the Epitect WGBS Workflow (Illumina), and quantified using qPCR. WGBS data were generated on the Illumina Hiseq X Ten sequencing platform, following a 2 x 150 indexed model.

*Analysis of 5mC Data*: Raw sequencing data were processed to filter out reads containing adapters by cutadapt v1.9.1 and low‐quality bases. Low‐quality reads included those containing more than 10% unknown or poor‐quality bases. The clean sequences were then aligned to the reference genome using the Bismark aligner (v0.18.2) with the parameters (‐N 1,‐L 20, –bowtie2). Methylated cytosines were extracted from aligned reads using the Bismark methylation extractor with default parameters. The methylation level for an individual cytosine was determined by the number of methylated reads divided by the total number of reads. Considering inefficiencies in the bisulfite conversion reaction and sequencing errors, the binomial test was used to determine if the observed methylation frequency was above the background (FDR < 0.05). All the analyses were done using sites covered by a minimum of ten reads in both samples. Only 5mC sites supported by both biological replicates were considered for further analysis. 5mC sites were classified into CG, CHG and CHH methylation motifs. The average methylation level at a 100 bp sliding window (step = 20 bp) was calculated for both gene bodies and its 2 kb flanking region (TSS plot). Methylation levels of the genes, which were grouped into deciles from the lowest first to the highest tenth based on their expression levels (FPKM), were calculated to assess the correlation between methylation and gene expression levels.

*qPCR Analysis of pre‐miRNA Expression*: The expression of ten random selected pre‐miRNAs was examined by qPCR analysis on three biological replicates using 7900HT Fast Real‐Time PCR systems (Applied Biosystems) with Maxima SYBR Green/ROX qPCR Master Mix (Fermentas). The expression of *C. reinhardtii beta subunit‐like polypeptide* (*Cblp*) was used as an internal control. The difference between the cycle threshold (Ct) of target genes and the Ct of control primers (ΔCt = Ct_target gene_ − Ct_control_) was used to calculate the normalized expression of target genes. The qPCR primers are listed in Table S4 in the Supporting Information.

*Gene Ontology Analysis*: Virtually translated *M. viride* proteins were searched against NR database using BLASTP (best hit with *E* value < 1e−5 ) in Blast2GO.^88^ Plant database (PLN) and bacterial database (BCT) were selected for BLASTP alignment. For sequences commonly supported by two databases, the corresponding best hit of plant or bacterial databases was assigned to the predicted genes. GO enrichment was assessed using Fisher's exact test, and the adjusted *p* value was then calculated using the Benjamini–Hochberg method.

*Measurement of Photosynthesis Activities*: Photosynthesis activities of *M. viride* were monitored by steady‐state oxygen evolution rates with a Clark‐type oxygen electrode (DW1, Hansatech) at 23 °C. Light response curves were measured by exposure of cells to illumination with different light intensities. Before measurement, the cells were collected and resuspended in fresh growth medium at a chlorophyll concentration of 10 µg mL^−1^. Measurements were performed by incubating 1 mL cell suspension with specific electron acceptors. For net photosynthesis measurement, 10 × 10^−3^ m NaHCO_3_ was supplemented. Dark respiratory rate was recorded immediately after saturating light illumination of 2 min. The PSII activity was measured in the presence of 0.4 × 10^−3^ m 2,6‐dichloro‐*p*‐benzoquinone (DCBQ) and 1 × 10^−3^ m K_3_Fe(CN)_6_. The PSI electron transfer rate was measured in the presence of 20 × 10^−6^ m 3‐(3,4‐dichlorophenyl)‐1,1‐dimethylurea (DCMU), 1 × 10^−3^ m sodium ascorbate, 1 × 10^−3^ m diaminodurene, and 1.5 × 10^−3^ m methyl viologen. P_700_ oxidation and P_700_^+^ reduction were monitored by the absorbance changes at 705 nm using a Joliot‐Type Spectrophotometer (JTS‐10, Bio‐Logic Scientific Instruments). Dark‐adapted cell suspensions in the presence of 20 × 10^−6^ m DCMU were illuminated with orange (630 nm) actinic light for 20 s and followed by darkness for 20 s.

*Protein Isolation and Western Blot Analysis: M. viride* and *C. reinhardtii* thylakoids were isolated as previously reported with minor modifications.^89^ Briefly, cells were resuspended in isolation buffer [25 × 10^−3^ m HEPES‐KOH (pH 7.5), 0.3 m sucrose, and 1 × 10^−3^ m MgCl_2_] and broken by vortexing six times for 1 min each at 4 °C in the presence of glass beads. The cell extract was centrifuged at 3000 × *g* for 5 min to remove glass beads and unbroken cells. Thylakoid membranes were pelleted by centrifugation at 30 000 × *g* for 20 min, resuspended in storage buffer [25 × 10^−3^ m HEPES‐KOH (pH 7.5), 0.3 m sucrose, and 1 × 10^−3^ m MgCl_2_], frozen in liquid N_2_, and kept at −80 °C. Samples containing thylakoid membrane protein were quantified based on their chlorophyll contents before being denatured in Laemmli SDS sample buffer containing 5% β‐mercaptoethanol and 6 m urea at room temperature for at least 1 h, and resolved by 12% polyacrylamide gel containing 6 m urea. Separated proteins were electro‐transferred to a polyvinylidene fluoride (PVDF) membrane (Immobilon‐P, Millipore) using a semidry apparatus (Bio‐Rad). The polyclonal antibodies against D1 (Cat#: AS111786), PsaB (Cat#: AS10695), Cytf (Cat#: AS08306), and AptB (Cat#: AS05085) proteins were purchased from Agrisera.

*Data Availability*: The genome assembly for *M. viride* has been deposited in the NCBI Genome with the accession number: RPFO00000000. The raw data of PacBio SMRT sequencing, including genome sequencing and full‐length transcriptome sequencing, have been deposited in the NCBI BioProject with the accession numbers: PRJNA510214 and PRJNA509752, respectively. The raw data of Illumina sequencing, including LncRNA sequencing, small RNA sequencing, RNA‐seq under different treatments, and whole‐genome bisulfite sequencing, have been deposited in the NCBI Gene Expression Omnibus (GEO) with the accession number: GSE123852. All the other data are available from the corresponding authors upon request.

## Conflict of Interest

The authors declare no conflict of interest.

## Supporting information

Supporting Information

Supporting Information

Supplemental Table 1

Supplemental Table 2

Supplemental Table 3
