# Supplementary material for: Mesostigma viride Genome and Transcriptome Provide Insights into the Origin and Evolution of Streptophyta
Source: Adv Sci (Weinh). 2019 Oct 24;7(1):1901850. doi: 10.1002/advs.201901850 (PMC6947507; doi:10.1002/advs.201901850)
Supplement: Supplementary file 1 — Supporting Information [file ADVS-7-1901850-s001.pdf]

Copyright WILEY-VCH Verlag GmbH & Co. KGaA, 69469 Weinheim, Germany, 2019.

Supporting Information

for *Adv. Sci.,* DOI: 10.1002/advs.201901850

Mesostigma viride Genome and Transcriptome Provide Insights into the Origin and Evolution of Streptophyta

Zhe Liang, Yuke Geng, Changmian Ji, Hai Du, Chui Eng Wong, Qian Zhang, Ye Zhang, Pingxian Zhang, Adeel Riaz, Sadaruddin Chachar, Yike Ding, Jing Wen, Yunwen Wu, Mingcheng Wang, Hongkun Zheng, Yanmin Wu, Viktor Demko, Lisha Shen, Xiao Han, Pengpeng Zhang,* Xiaofeng Gu,* and Hao Yu**

Supporting Information

Mesostigma viride Genome and Transcriptome Provide Insights into the Origin and Evolution of Streptophyta

Zhe Liang, Yuke Geng, Changmian Ji, Hai Du, Chui Eng Wong, Qian Zhang, Ye Zhang, Pingxian Zhang, Adeel Riaz, Sadaruddin Chachar, Yike Ding, Jing Wen, Yunwen Wu, Mingcheng Wang, Hongkun Zheng, Yanmin Wu, Viktor Demko, Lisha Shen, Xiao Han, Pengpeng Zhang,* Xiaofeng Gu,* and Hao Yu**

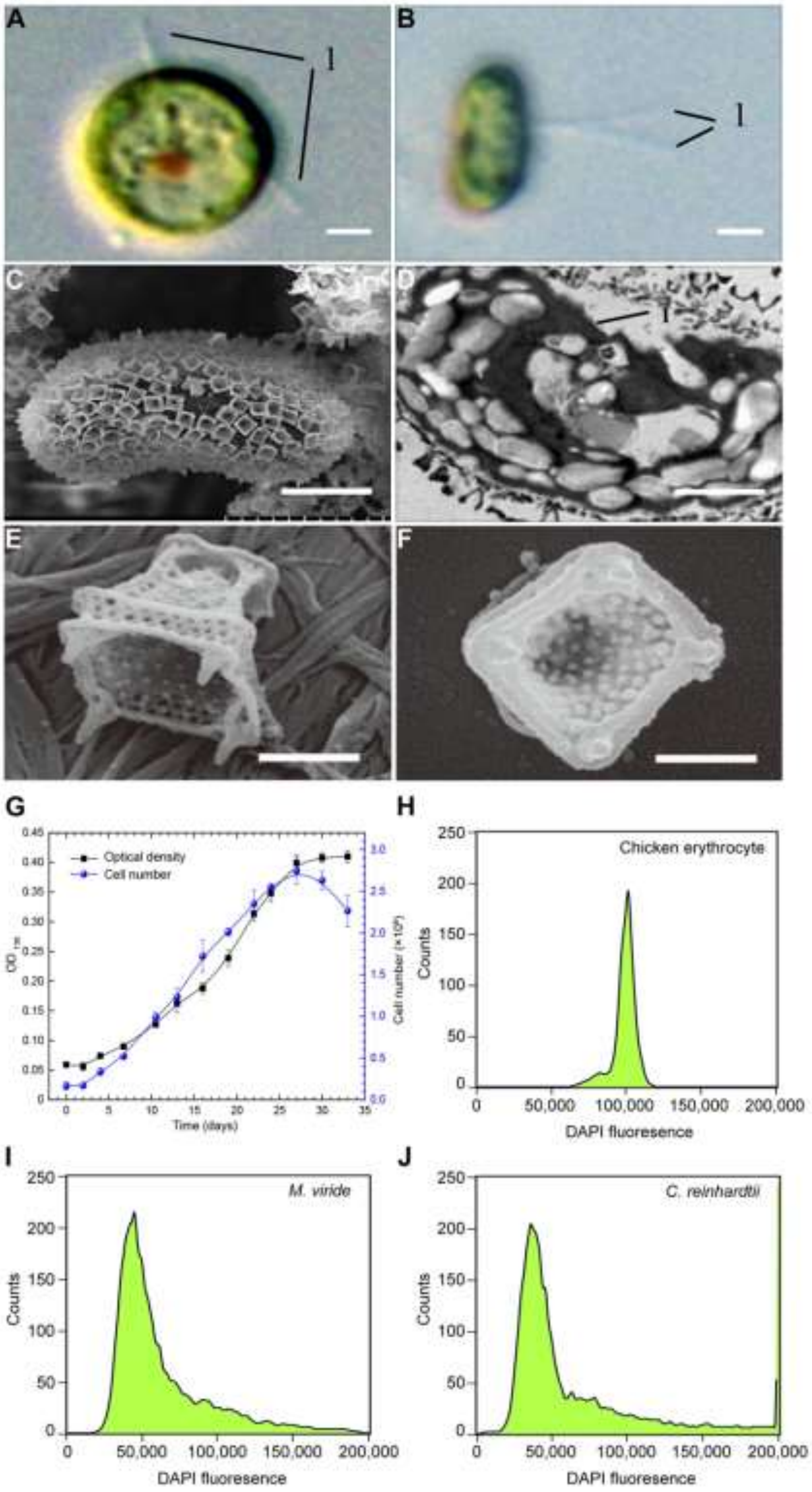

Figure S1. Morphology, growth rate and flow cytometry analysis of *M. viride.* A,B) Optical micrograph of two *M. viride* cells. 1, flagellum. Scale bars, 2.5 µm. C) Scanning electron micrograph of *M. viride* cell surface shows its unified basket-like scales. Scale bars, 2.5 µm. D) Ultrastructure of a *M. viride* cell observed under transmission electron microscope. 1, chloroplast. Scale bars, 2.5 µm. E,F) Scanning electron micrograph of two basket-like scales. Scale bars, 0.25 µm. G) Growth curves of *M. viride* cells. Error bars, mean ± SD; $n = 3$ biological replicates. H-J) Flow cytometry analysis of *M. viride*. There was no obvious

change in the DNA content of *M. viride* cells during the first 24 h of growth, suggesting that *M. viride* grows as a haploid organism under our experimental conditions. Chicken erythrocyte and *C. reinhardtii* cells, which are known haploid organisms, served as internal reference standards.

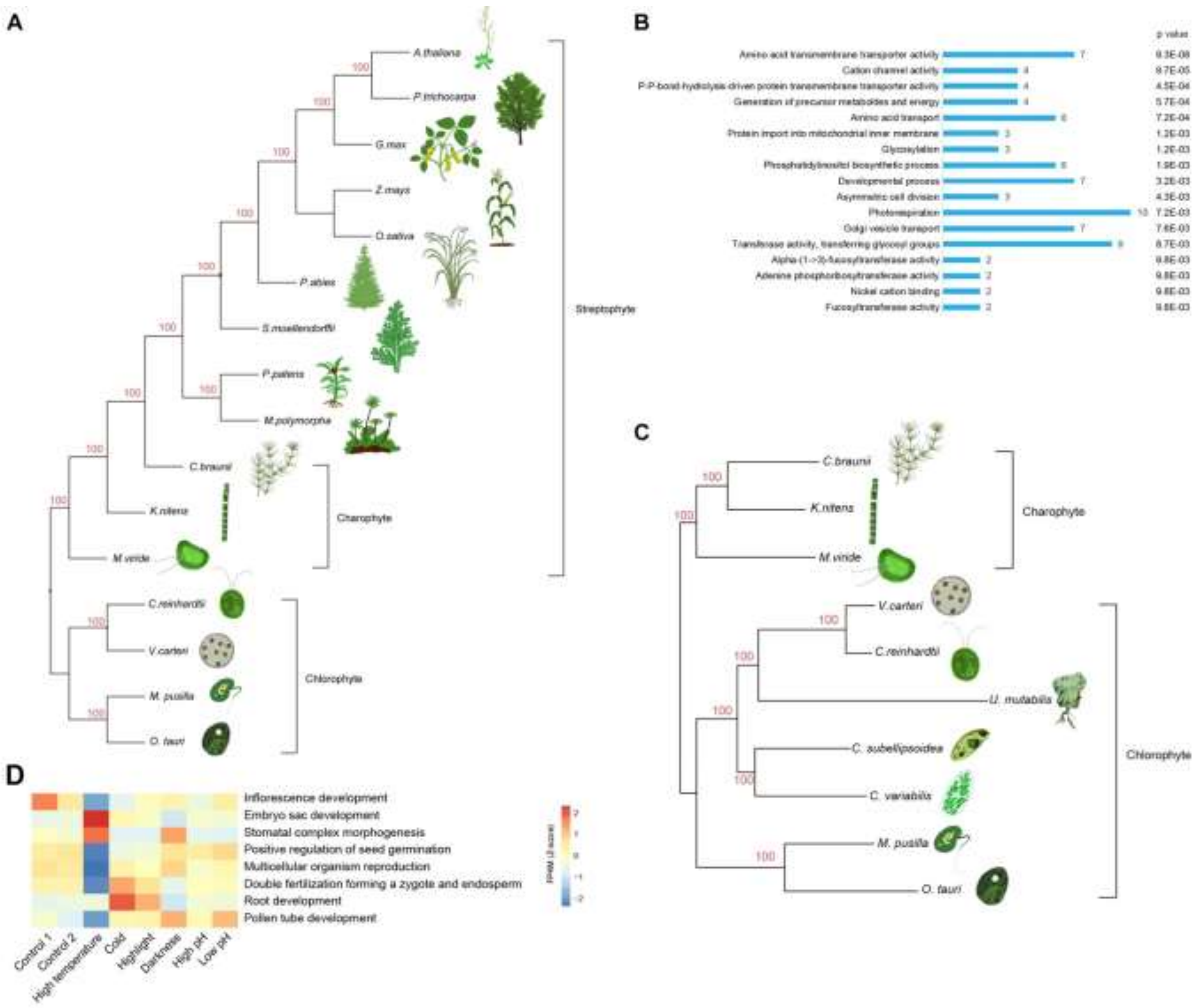

Figure S2. Evolutionary analysis of *M. viride* with other selected green plant species. A) Species tree of *M. viride* and 15 evolutionarily representative green algae and land plant species inferred from Maximum Likelihood (ML) method based on 117 single copy orthologs. Numbers represent support values after 1,000 bootstrap replicates. B) Gene ontology (GO) enrichment analysis of genes gained in *K. nitens* from *M. viride*. *p* values are calculated using the Fisher's Exact test. C) Species tree of 3 charophyte species and 7 chlorophyte species inferred from ML method based on 1,091 single copy orthologs. Numbers represent support values after 1,000 bootstrap replicates. D) Heatmap showing expression profiles of the genes related to GO terms specifically found in charophytes but not in chlorophytes.

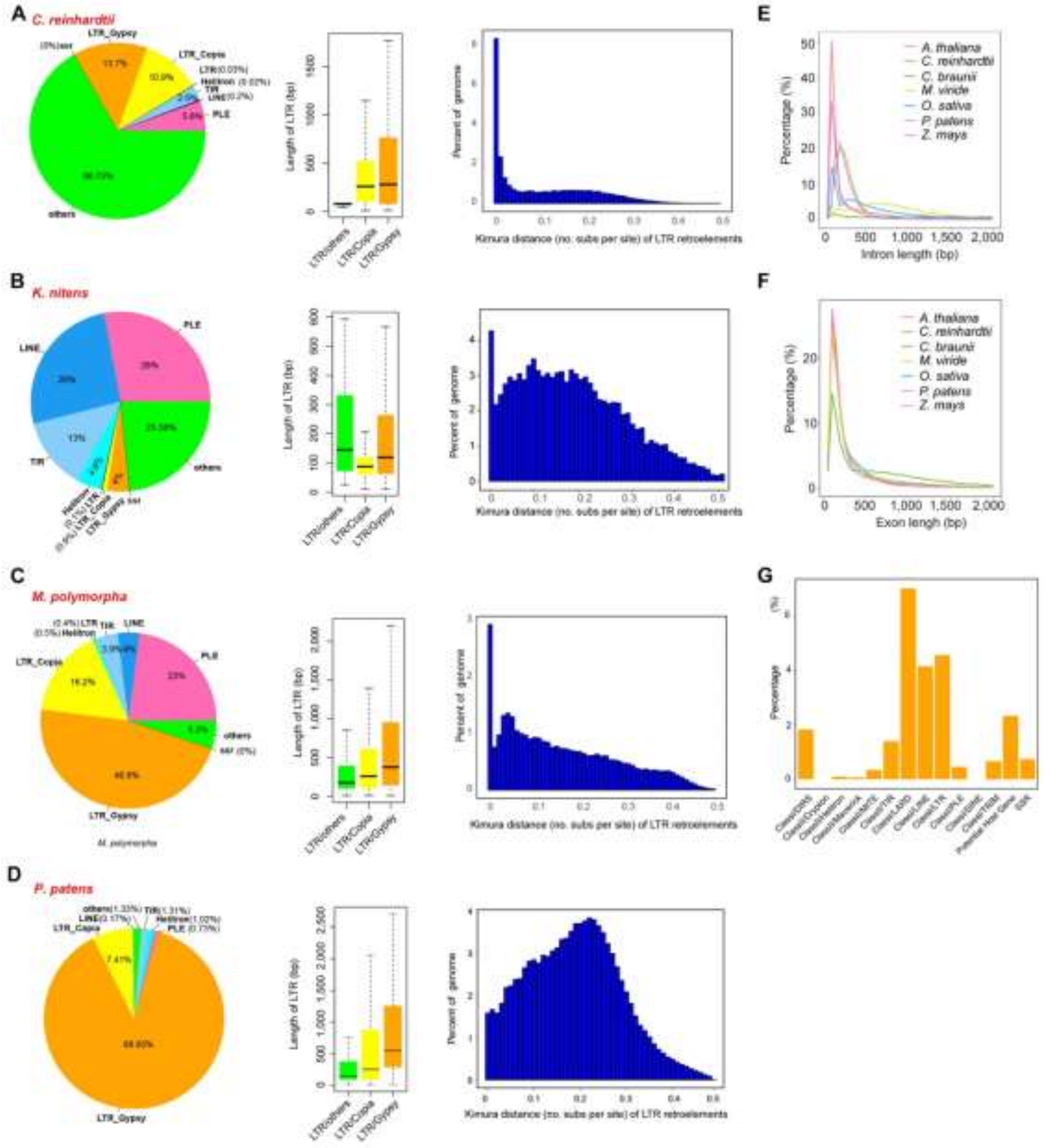

Figure S3. Analysis of repetitive elements and length of exons and introns. A-D) Left panels show pie charts of major repeat classes (LTR, long terminal repeat; LINE, long interspersed nuclear element; PLE, Penelope-like element; TIR, terminal inverted repeat) in the genomes of *C. reinhardtii* (A), *K. nitens* (B), *M. polymorpha* (C) and *P. patens* (D). Middle panels show box plots of the length distribution of LTR families in different genomes. Boxes indicate the first quartile, median and third quartile with whiskers extending up to 1.5 times the interquartile distance. Right panels show Relative age (Kimura distance) computed for LTR retroelements, suggesting a prolonged transposition activity of LTR retroelements in the three genomes with an apparent transposition burst in *K. nitens* and *M. polymorpha*. Y-axis represents genome coverage for LTR retrotransposons, while X-axis represents Kimura distance. E) Comparison of the exon length among *M. viride* and other selected species shows no obvious difference in the exon length. F) Comparison of the intron length among *M. viride* and other selected species. G) Different categories of repeat elements found in *M. viride* introns.

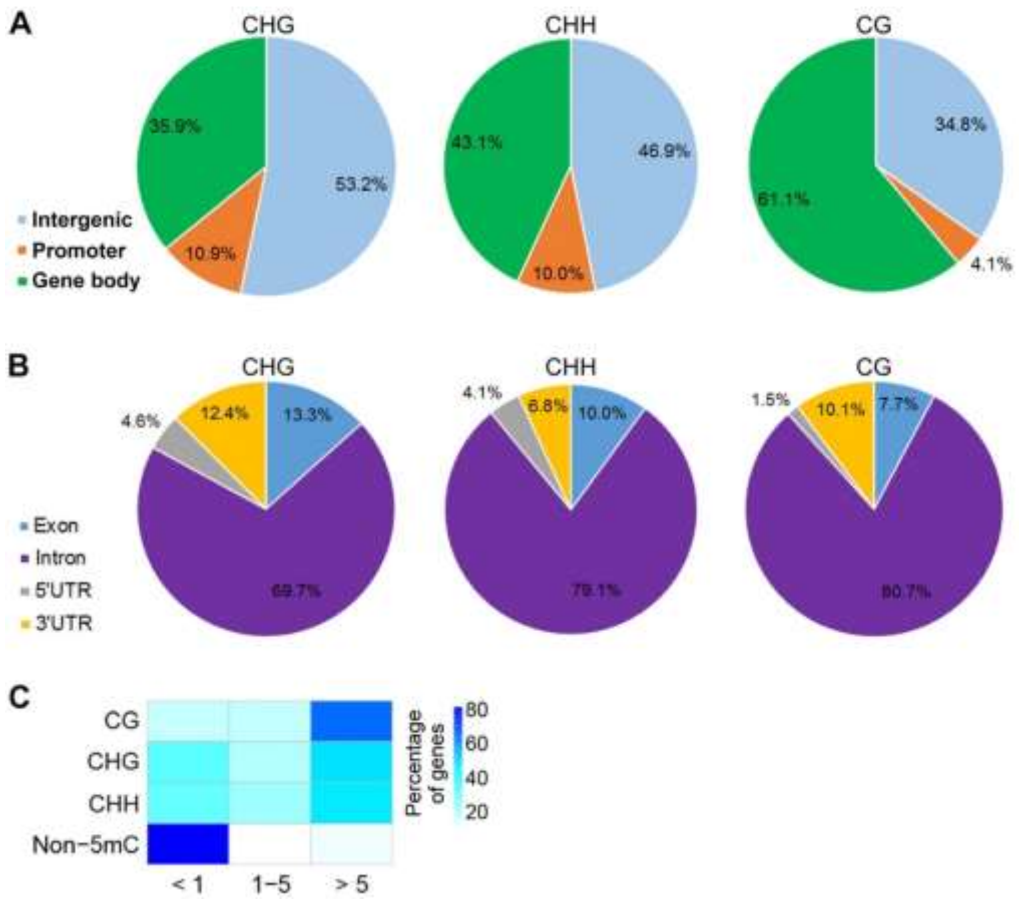

Figure S4. 5mC DNA methylation in *M. viride*. A) Distribution of 5mC sites in genomic regions divided into gene bodies, promoters (within 2 kb upstream of TSS) and intergenic regions. B) Distribution of 5mC sites within gene bodies divided into exons (excluding 5'and 3'UTRs in protein-coding genes), introns, and 5' UTRs and 3'UTRs. C) Percentages of 5mC-methylated and unmethylated genes at a given FPKM level (< 1, 1–5 or > 5).

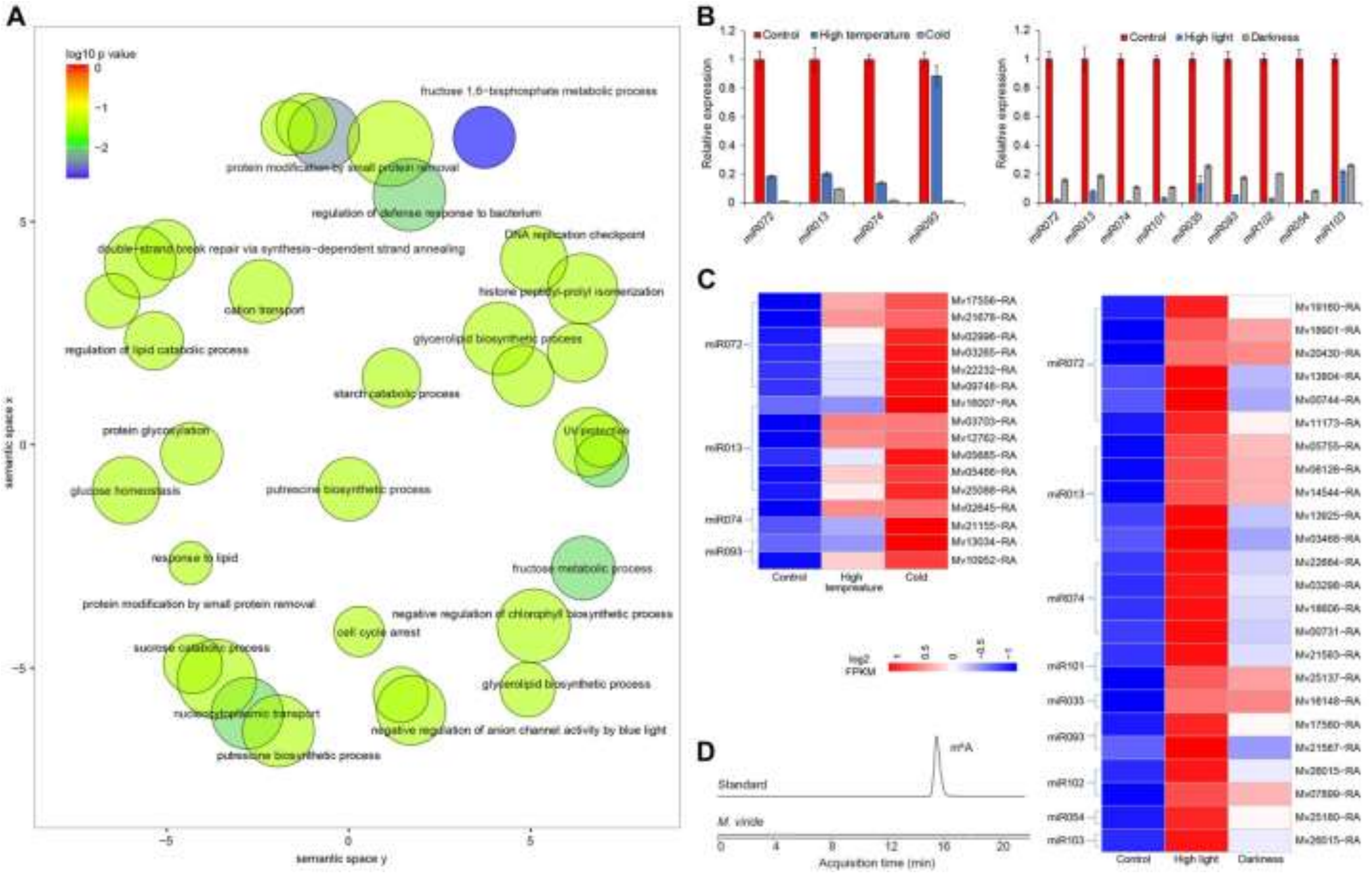

Figure S5. miRNA regulation and m^{6}A modification in *M. viride*. A) Gene ontology (GO) analysis of *M. viride* miRNA target genes. Scatter plots showing biological processes that are significantly enriched in miRNA target genes. B) qPCR analysis of randomly selected pri-miRNA under different temperature and light conditions. Gene expression levels in the control are set as 1. Error bars, mean ± SD; n = 3 biological replicates. C) Heatmap showing the expression of miRNA target genes under different temperature (left) and light (right) conditions. Their expression negatively correlates with the expression of their corresponding pre-miRNAs (B) under different growth conditions. D) Ion chromatograms of m^{6}A levels in standard and mRNA from *M. viride* show undetectable m^{6}A modification in *M. viride* mRNA.

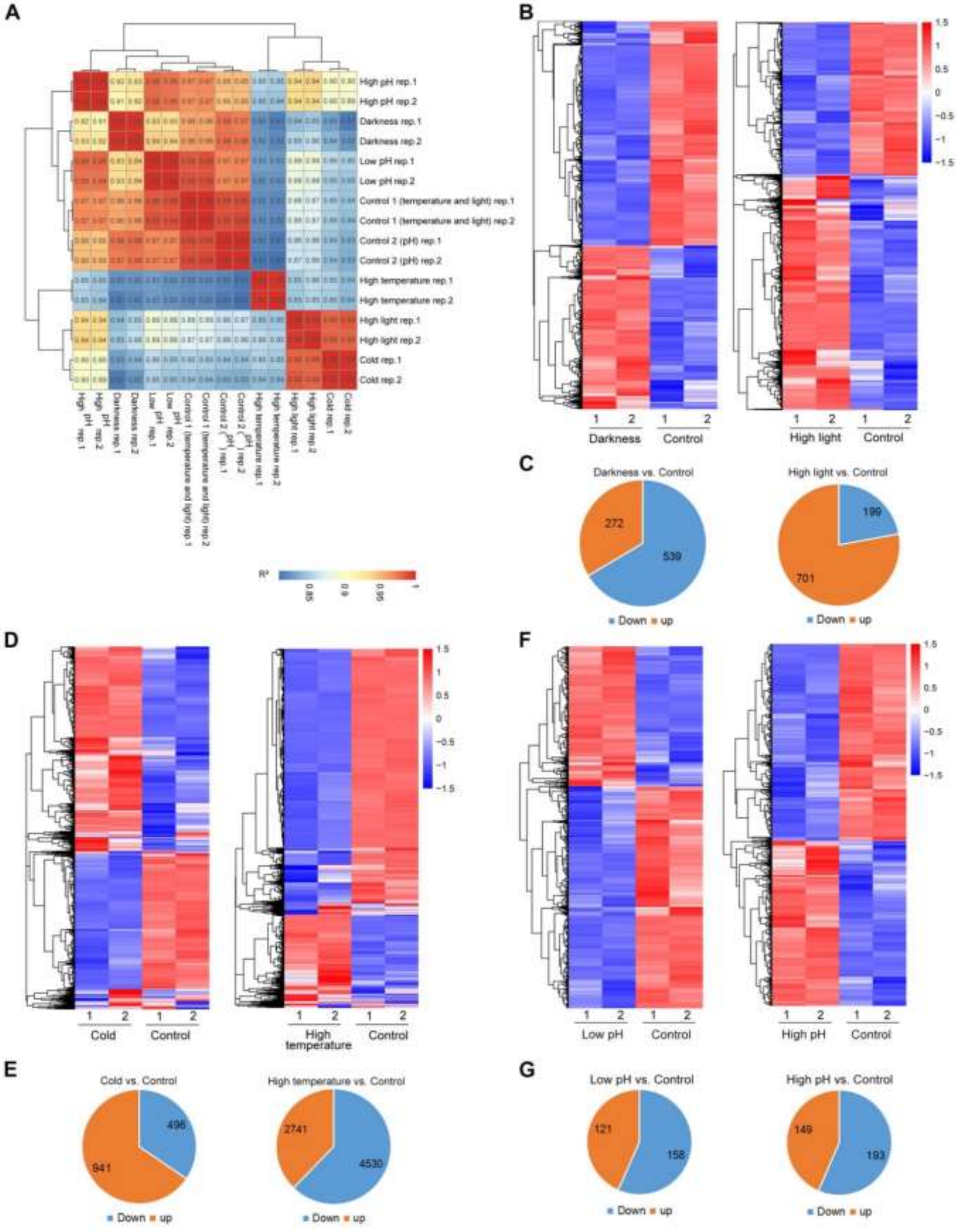

Figure S6. RNA-seq analysis of *M. viride* gene expression under different environment conditions. A) Heatmap showing Pearson correlations between RNA-seq experiments. There

were two biological replicates for each experimental condition. B) Heatmap showing differentially expressed genes (DEGs) under darkness (left panel) and high light (right panel) conditions. C) Number of up- or down-regulated genes under darkness (left panel) and high light (right panel) conditions. D) Heatmap showing DEGs under cold (left panel) and high temperature (right panel) conditions. E) Number of up- or down-regulated genes under cold (left panel) and high temperature (right panel) conditions. F) Heatmap showing DEGs under low pH (left panel) and high pH (right panel) conditions. G) Number of up- or down-regulated genes under low pH (left panel) and high pH (right panel) conditions.

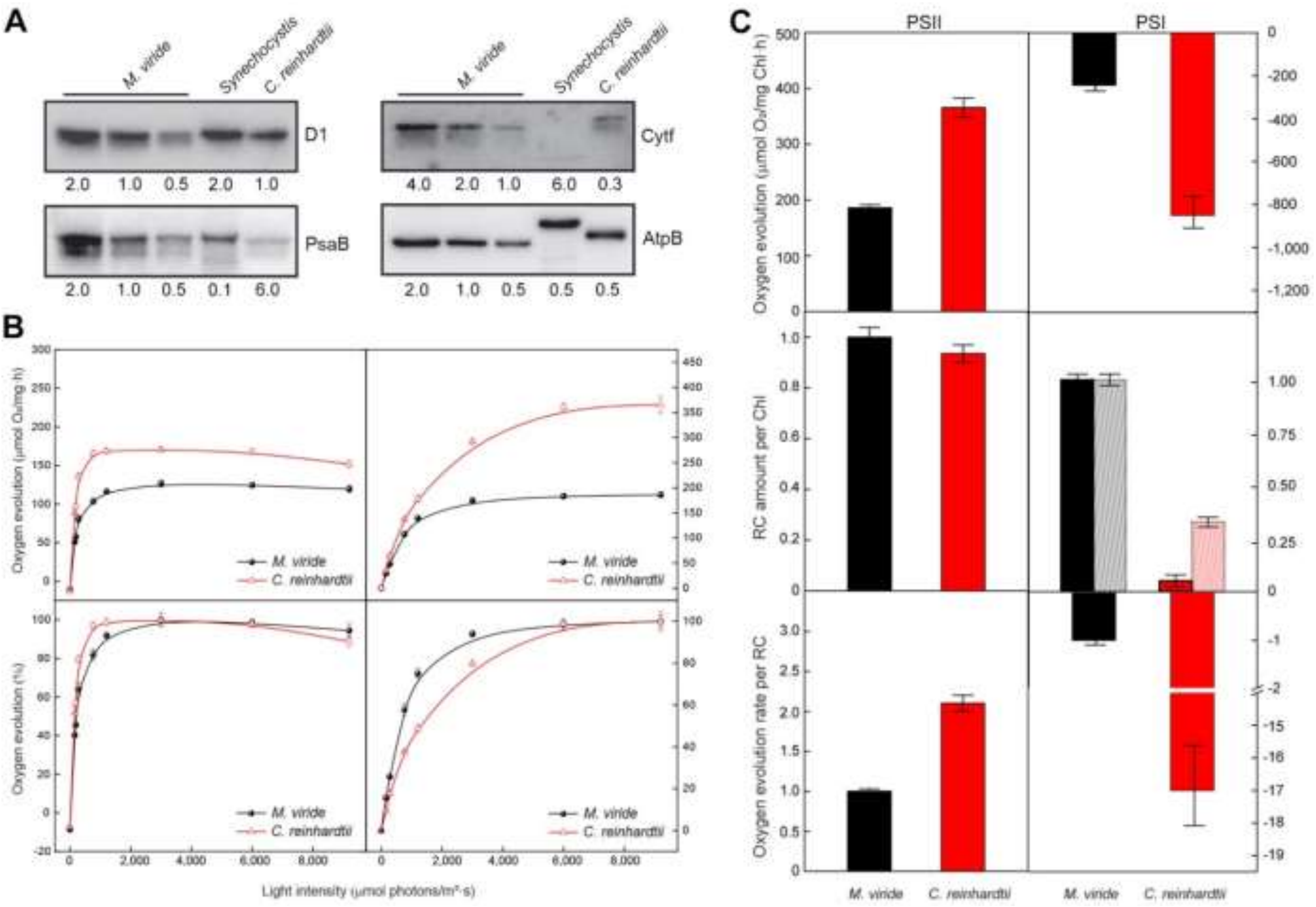

Figure S7. Examination of photosynthesis in *M. viride*. A) Western blot analysis of the major thylakoid membrane proteins in *M. viride*, including D1 of Photosystem II (PSII), PsaB of Photosystem I (PSI), Cytf of the cytochrome b_6f complex, and AtpB of the ATP synthase. Numbers under each blot indicate the loading amount (μg) of chlorophyll measured by spectrophotometer. B) The light response curves of *M. viride* and *C. reinhardtii*. Oxygen evolution rates were measured under different intensities of white light. The left two panels indicate net photosynthesis (H_2O to CO_2) measured in the presence of 10 mM $NaHCO_3$, while the right two panels show PSII activity (H_2O to DCBQ) measured in the presence of 0.35 mM DCBQ and 1 mM $K_3Fe(CN)_6$. The upper panels show the absolute oxygen evolution rates, while the lower panels show the relative values normalized to the respective maximum rates in the corresponding upper panels. Error bars, mean ± SD, $n = 3$. C) Comparison of PSII (left panels) and PSI (right panels) activities of *M. viride* and *C. reinhardtii*. The absolute oxygen evolution (top panels) rates were measured with a Clark-type oxygen electrode under saturating light (6,000 μmol photons m^{-2} s^{-1}). The relative amount of PSII reaction center (RC) (middle left panel) was calculated by normalizing D1 signal intensity in the Western blot using ImageJ to the chlorophyll amount shown in (A**)**. The relative amount of PSI RC (middle right panel) was calculated by normalizing either PsaB signal intensity in the Western blot (solid fill) or the changes in P700 absorbance (upward diagnonal fill) to the chlorophyll amount shown in (B**)**. The relative oxygen evolution rate per RC (bottom panels) was calculated by dividing the oxygen evolution rate (top panels) by the corresponding RC amount (middle panels). Error bars, mean ± SD, $n = 3$.

Table S1. Statistics of the *M. viride* genome sequencing and annotation.
This table is available online as a Microsoft Excel file.

Table S2. List of cell division, cell wall synthesis, transcription factor, phytohormone, epigenetic and photosynthesis genes and charophyte/chlorophyte-specific GO terms.
This table is available online as a Microsoft Excel file.

Table S3. List of differentially expressed genes in different treatments.
This table is available online as a Microsoft Excel file.

Table S4. List of primers used in this study.

Primer pairs to detect pre-miRNA expression levels

Primer ID	Primers
072	AAACCAGATGATGGATGCGG<br>CCGGCGTCATCTCCAACAG
013	CTTGGTGGAGTCGCAGCTC<br>ATCATAATGCTGCTGCTCCC
074	TCCGGAGCTGTTCGAAGCA<br>TTTATTGCTCCACCCCCACA
101	TAAGGCCATTCCCATGTGCG<br>AGCATATTTGCGACAGTCCC
035	GACTACGGAGTTGCACGTTG<br>GCACATGGCAACCCAGCAA
093	CATCTGAAGGTCCTGTGGC<br>CCGGTGGGAT TTGAACCCA
102	GTGGATGGGTGTATGGGGTG<br>CATGACACCC ATCCATGCAT
054	CAGACCACCTCCAGACGC<br>AGCTGCTGAC TCCTGTCAG
103	TGGGTGTAATGGATGGCTGG<br>ATGCCAGCCA CCCATCACA
007	GCGGTTTGTTTCAACCACAA<br>CTCTCGATGA GGGCGACG
CBLP	TGCTGTGGGACCTGGCTGA<br>GCCTTCTTGCTGGTGATGTTG

Data S1. Phylogenetic trees.
This Data is available online as a PDF file.
