# Supplementary material for: Mesostigma viride Genome and Transcriptome Provide Insights into the Origin and Evolution of Streptophyta
Source: Adv Sci (Weinh). 2019 Oct 24;7(1):1901850. doi: 10.1002/advs.201901850 (PMC6947507; doi:10.1002/advs.201901850)
Supplement: Supplementary file 2 — Supporting Information [file ADVS-7-1901850-s002.pdf]

Data S1. Phylogenetic trees.

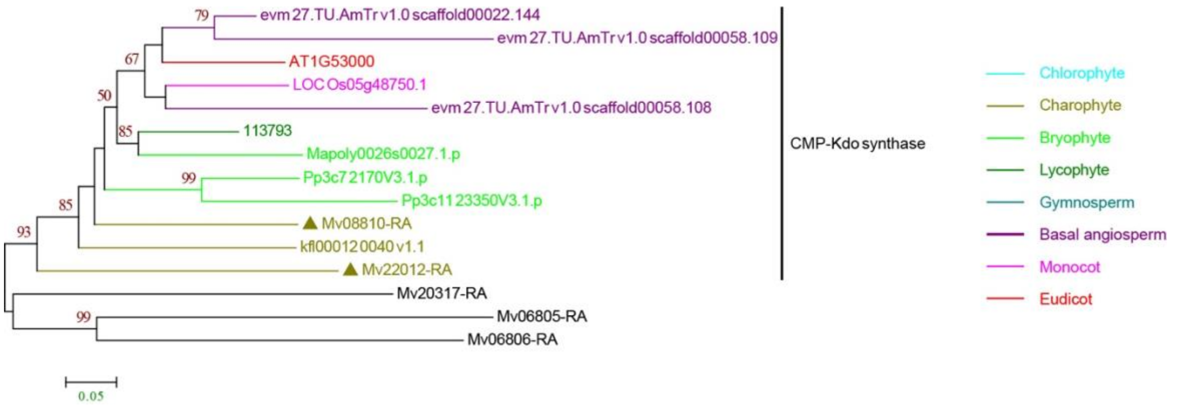

Data S1A. Phylogenetic relationships of CMP-Kdo synthetase proteins from *M. viride* and other evolutionarily representative species. The *M. viride* sequences were highlighted by triangles. Numbers on the major branches indicate bootstrap values (> 50%) in 1,000 replicates.

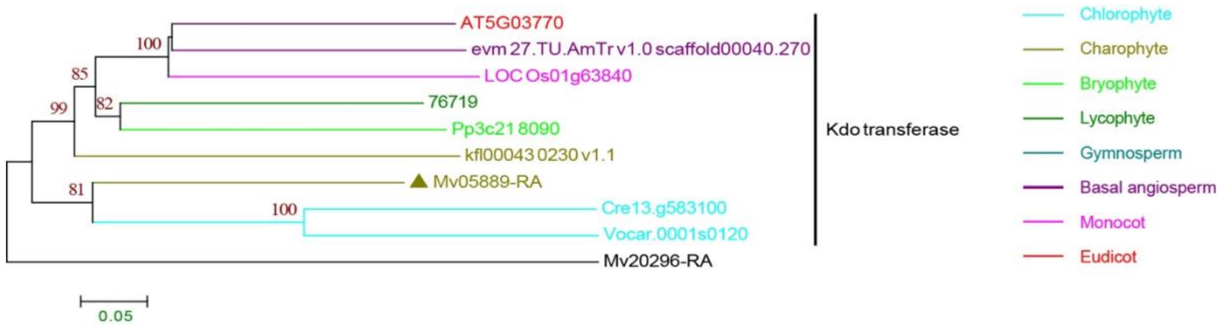

Data S1B. Phylogenetic relationships of Kdo transferase proteins from *M. viride* and other evolutionarily representative species. The *M. viride* sequences were highlighted by triangles. Numbers on the major branches indicate bootstrap values (> 50%) in 1,000 replicates.

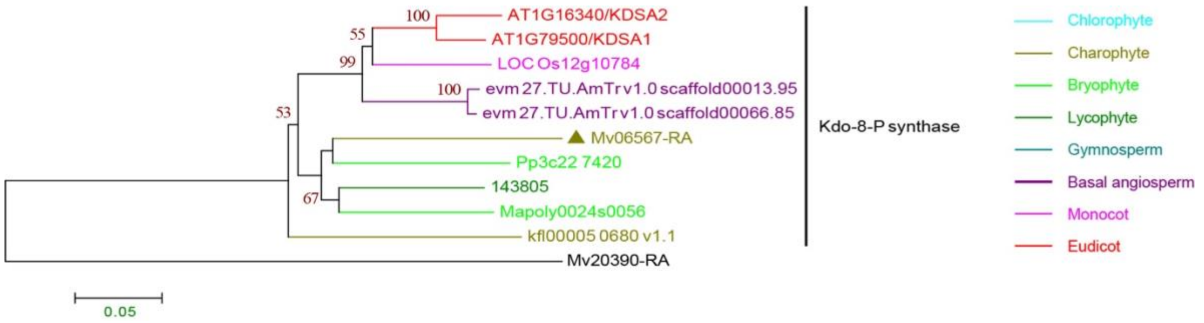

Data S1C. Phylogenetic relationships of Kdo-8-phosphate (Kdo-8-p) synthase proteins from *M. viride* and other evolutionarily representative species. The *M. viride* sequences were highlighted by triangles. Numbers on the major branches indicate bootstrap values (> 50%) in 1,000 replicates.

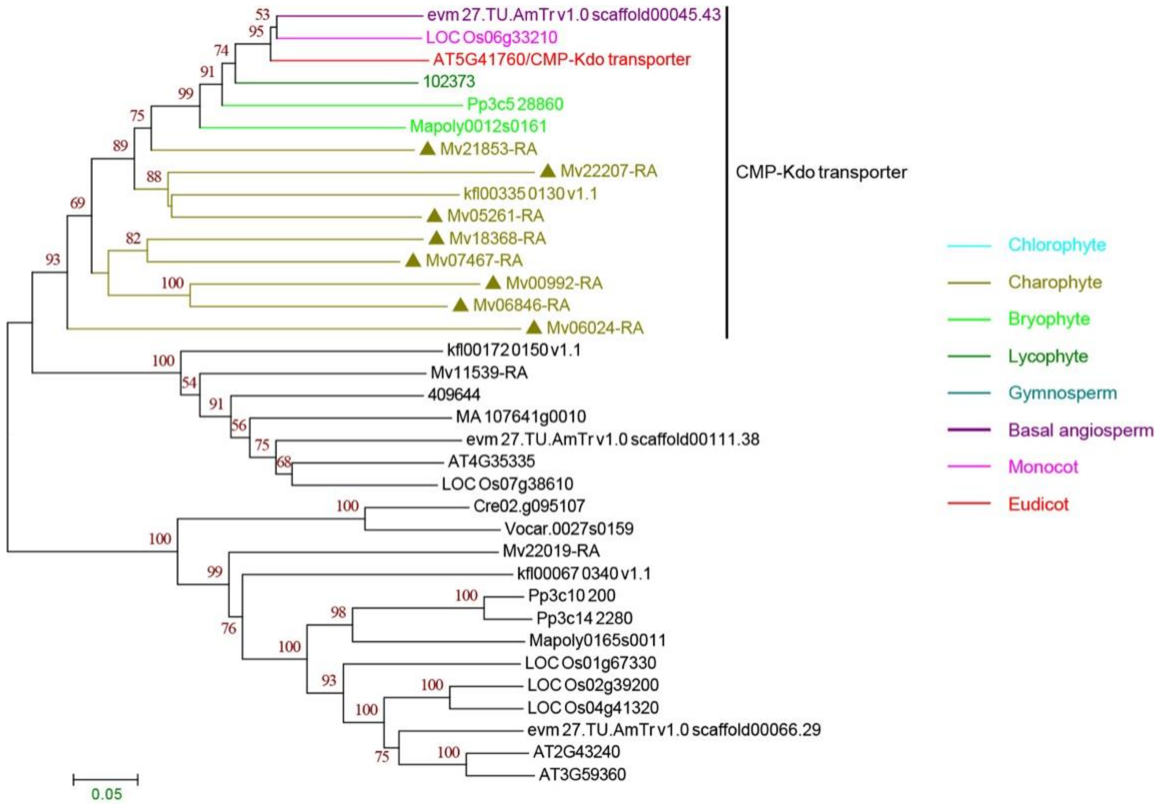

Data S1D. Phylogenetic relationships of CMP-Kdo transporter proteins from *M. viride* and other evolutionarily representative species. The *M. viride* sequences were highlighted by triangles. Numbers on the major branches indicate bootstrap values (> 50%) in 1,000 replicates.

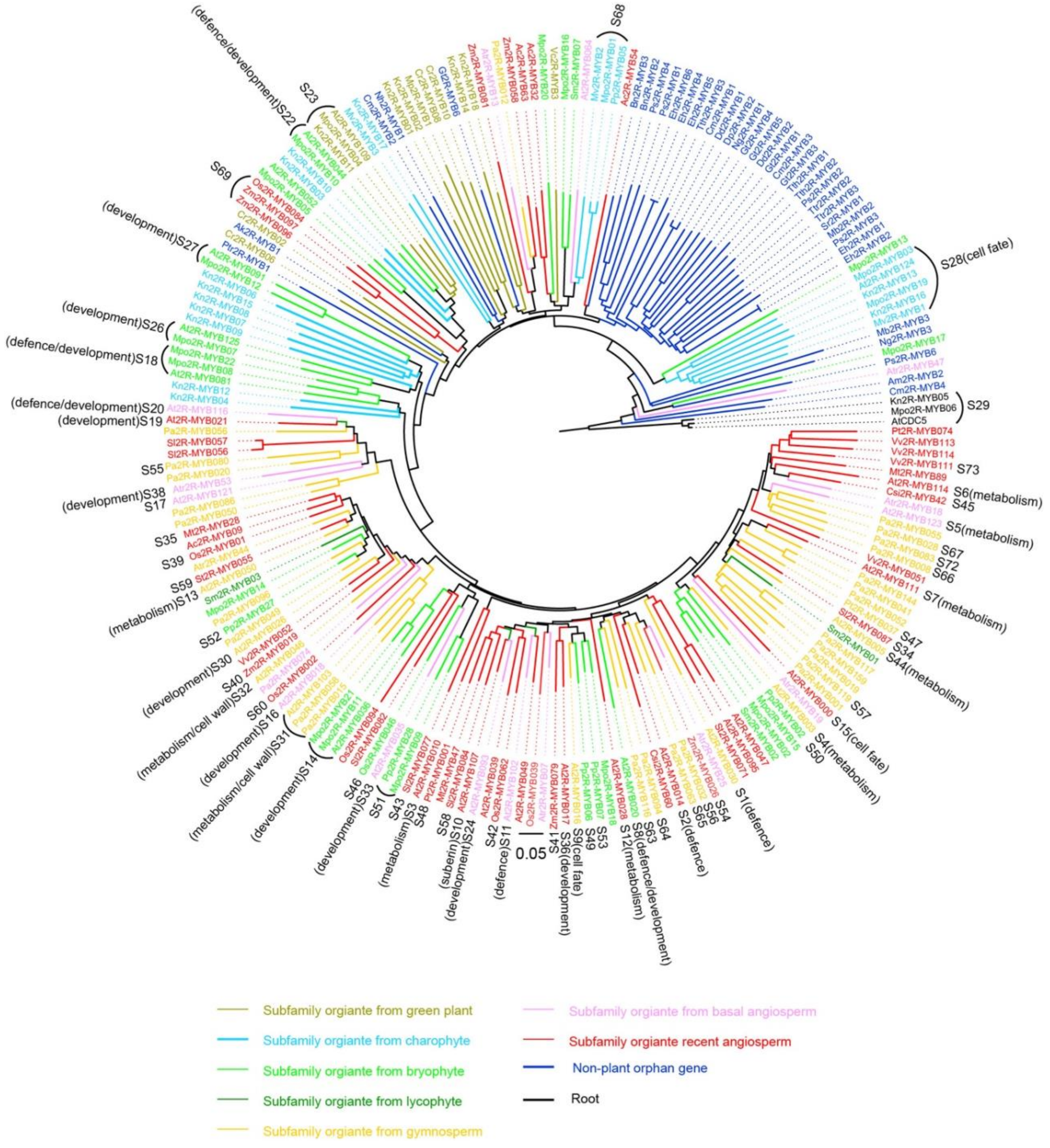

Data S1E. Phylogenetic relationships of R2R3-MYB TFs. The R2R3-MYB neighbor-joining (NJ) phylogenetic tree includes representative sequences from previously identified 73 subfamilies and 95 non-plant orphan genes based on 50 eukaryotes,[1] and all R2R3-MYB proteins from *K. nitens*, *M. polymorpha*, and *M. viride*. The subfamily names and related functions were indicated. The three shows that *M. viride* proteins (Mv2R-MYB1-3) belong to the subfamilies of S28, S68 and one yet-to-be defined subfamily, respectively.

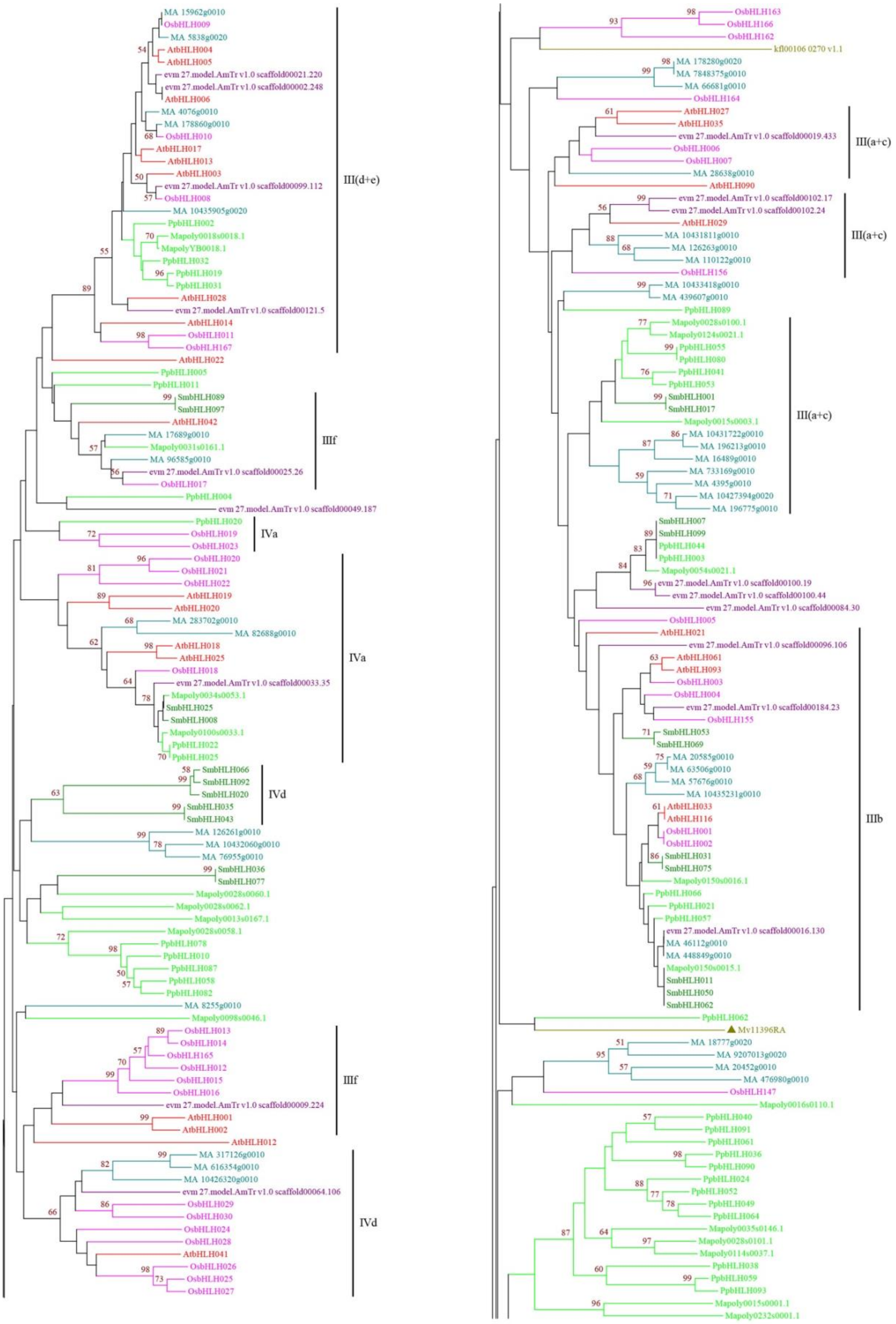
MA 15962g0010
OsbHLH009
MA 5838g0020
AtbHLH004
AtbHLH005
evm 27.model.AmTr v1.0 scaffold00021.220
evm 27.model.AmTr v1.0 scaffold00002.248
AtbHLH006
MA 4076g0010
MA 178860g0010
OsbHLH010
AtbHLH017
AtbHLH013
AtbHLH003
evm 27.model.AmTr v1.0 scaffold00099.112
OsbHLH008
MA 10435905g0020
PpbHLH002
Mapoly0018s0018.1
MapolyYB0018.1
PpbHLH032
PpbHLH019
PpbHLH031
AtbHLH028
evm 27.model.AmTr v1.0 scaffold00121.5
AtbHLH014
OsbHLH011
OsbHLH167
AtbHLH022
III(d+e)
PpbHLH005
PpbHLH011
SmbHLH089
SmbHLH097
AtbHLH042
MA 17689g0010
Mapoly0031s0161.1
MA 96585g0010
evm 27.model.AmTr v1.0 scaffold00025.26
OsbHLH017
IIIf
PpbHLH004
evm 27.model.AmTr v1.0 scaffold00049.187
PpbHLH020
OsbHLH019
OsbHLH023
IVa
OsbHLH020
OsbHLH021
OsbHLH022
AtbHLH019
AtbHLH020
MA 283702g0010
MA 82688g0010
AtbHLH018
AtbHLH025
OsbHLH018
evm 27.model.AmTr v1.0 scaffold00033.35
Mapoly0034s0053.1
SmbHLH025
SmbHLH008
Mapoly0100s0033.1
PpbHLH022
PpbHLH025
IVa
SmbHLH066
SmbHLH092
SmbHLH020
SmbHLH035
SmbHLH043
IVd
MA 126261g0010
MA 10432060g0010
MA 76955g0010
SmbHLH036
SmbHLH077
Mapoly0028s0060.1
Mapoly0028s0062.1
Mapoly0013s0167.1
Mapoly0028s0058.1
PpbHLH078
PpbHLH010
PpbHLH087
PpbHLH058
PpbHLH082
MA 8255g0010
Mapoly0098s0046.1
OsbHLH013
OsbHLH014
OsbHLH165
OsbHLH012
OsbHLH015
OsbHLH016
evm 27.model.AmTr v1.0 scaffold00009.224
AtbHLH001
AtbHLH002
AtbHLH012
IIIf
MA 317126g0010
MA 616354g0010
MA 10426320g0010
evm 27.model.AmTr v1.0 scaffold00064.106
OsbHLH029
OsbHLH030
OsbHLH024
OsbHLH028
AtbHLH041
OsbHLH026
OsbHLH025
OsbHLH027
IVd
OsbHLH163
OsbHLH166
OsbHLH162
kfl00106 0270 v1.1
MA 178280g0020
MA 7848375g0010
MA 66681g0010
OsbHLH164
AtbHLH027
AtbHLH035
evm 27.model.AmTr v1.0 scaffold00019.433
OsbHLH006
OsbHLH007
MA 28638g0010
AtbHLH090
III(a+c)
evm 27.model.AmTr v1.0 scaffold00102.17
evm 27.model.AmTr v1.0 scaffold00102.24
AtbHLH029
MA 10431811g0010
MA 126263g0010
MA 110122g0010
OsbHLH156
III(a+c)
MA 10433418g0010
MA 439607g0010
PpbHLH089
Mapoly0028s0100.1
Mapoly0124s0021.1
PpbHLH055
PpbHLH080
PpbHLH041
PpbHLH053
SmbHLH001
SmbHLH017
Mapoly0015s0003.1
MA 10431722g0010
MA 196213g0010
MA 16489g0010
MA 733169g0010
MA 4395g0010
MA 10427394g0020
MA 196775g0010
III(a+c)
SmbHLH007
SmbHLH099
PpbHLH044
PpbHLH003
Mapoly0054s0021.1
evm 27.model.AmTr v1.0 scaffold00100.19
evm 27.model.AmTr v1.0 scaffold00100.44
evm 27.model.AmTr v1.0 scaffold00084.30
OsbHLH005
AtbHLH021
evm 27.model.AmTr v1.0 scaffold00096.106
AtbHLH061
AtbHLH093
OsbHLH003
OsbHLH004
evm 27.model.AmTr v1.0 scaffold00184.23
OsbHLH155
SmbHLH053
SmbHLH069
MA 20585g0010
MA 63506g0010
MA 57676g0010
MA 10435231g0010
AtbHLH033
AtbHLH116
OsbHLH001
OsbHLH002
SmbHLH031
SmbHLH075
Mapoly0150s0016.1
PpbHLH066
PpbHLH021
PpbHLH057
evm 27.model.AmTr v1.0 scaffold00016.130
MA 46112g0010
MA 448849g0010
Mapoly0150s0015.1
SmbHLH011
SmbHLH050
SmbHLH062
IIIb
PpbHLH062
Mv11396RA
MA 18777g0020
MA 9207013g0020
MA 20452g0010
MA 476980g0010
OsbHLH147
Mapoly0016s0110.1
PpbHLH040
PpbHLH091
PpbHLH061
PpbHLH036
PpbHLH090
PpbHLH024
PpbHLH052
PpbHLH049
PpbHLH064
Mapoly0035s0146.1
Mapoly0028s0101.1
Mapoly0114s0037.1
PpbHLH038
PpbHLH059
PpbHLH093
Mapoly0015s0001.1
Mapoly0232s0001.1

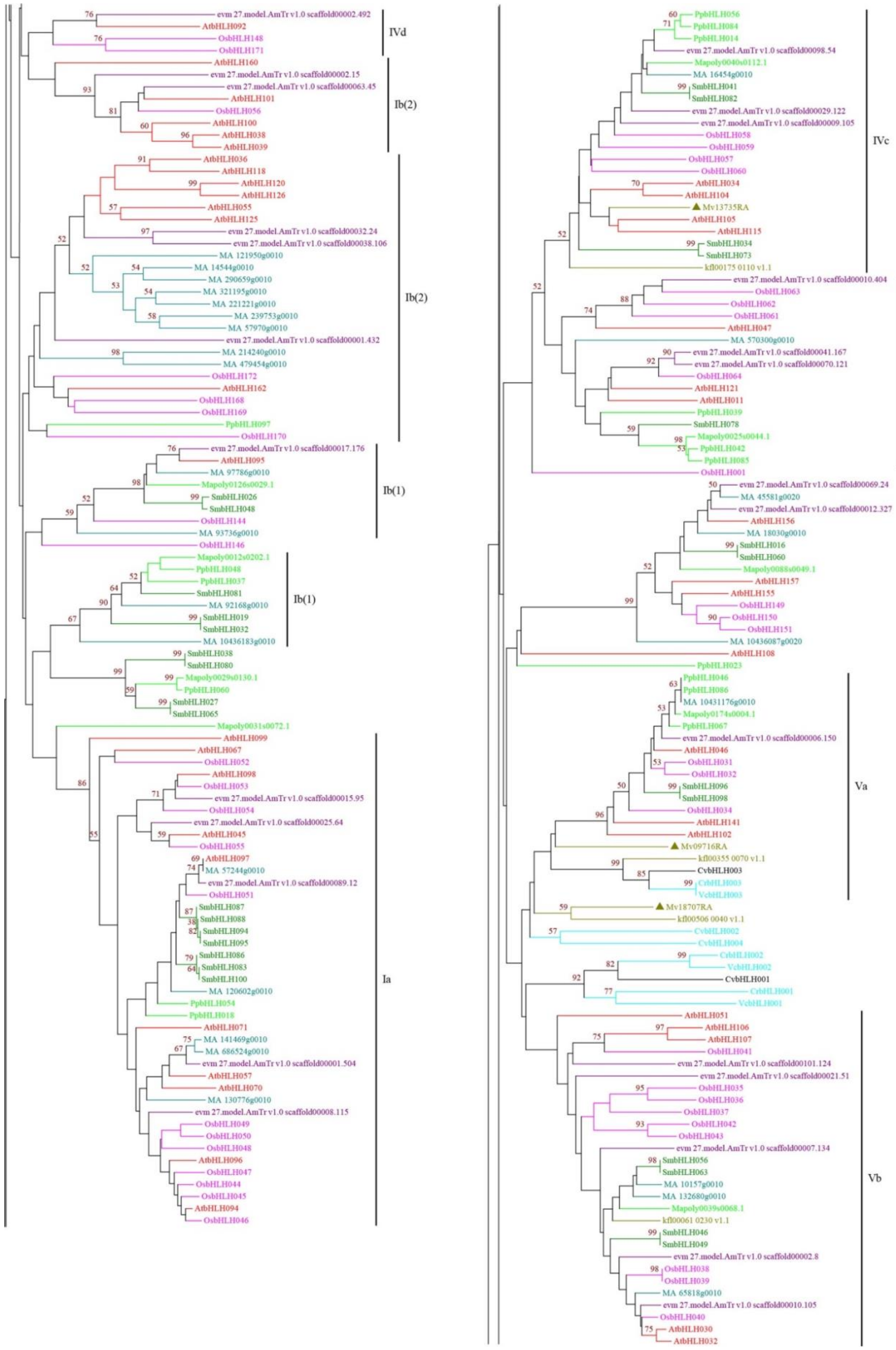
evm 27.model.AmTr v1.0 scaffold00002.492
AtbHLH092
OsbHLH148
OsbHLH171
IVd
AtbHLH160
evm 27.model.AmTr v1.0 scaffold00002.15
evm 27.model.AmTr v1.0 scaffold00063.45
AtbHLH101
OsbHLH056
AtbHLH100
AtbHLH038
AtbHLH039
Ib(2)
AtbHLH036
AtbHLH118
AtbHLH120
AtbHLH126
AtbHLH055
AtbHLH125
evm 27.model.AmTr v1.0 scaffold00032.24
evm 27.model.AmTr v1.0 scaffold00038.106
MA 121950g0010
MA 14544g0010
MA 290659g0010
MA 321195g0010
MA 221221g0010
MA 239753g0010
MA 57970g0010
evm 27.model.AmTr v1.0 scaffold00001.432
MA 214240g0010
MA 479454g0010
OsbHLH172
AtbHLH162
OsbHLH168
OsbHLH169
PpbHLH097
OsbHLH170
Ib(2)
evm 27.model.AmTr v1.0 scaffold00017.176
AtbHLH095
MA 97786g0010
Mapoly0126s0029.1
SmbHLH026
SmbHLH048
OsbHLH144
MA 93736g0010
OsbHLH146
Ib(1)
Mapoly0012s0202.1
PpbHLH048
PpbHLH037
SmbHLH081
MA 92168g0010
SmbHLH019
SmbHLH032
MA 10436183g0010
Ib(1)
SmbHLH038
SmbHLH080
Mapoly0029s0130.1
PpbHLH060
SmbHLH027
SmbHLH065
Mapoly0031s0072.1
AtbHLH099
AtbHLH067
OsbHLH052
AtbHLH098
OsbHLH053
evm 27.model.AmTr v1.0 scaffold00015.95
OsbHLH054
evm 27.model.AmTr v1.0 scaffold00025.64
AtbHLH045
OsbHLH055
AtbHLH097
MA 57244g0010
evm 27.model.AmTr v1.0 scaffold00089.12
OsbHLH051
SmbHLH087
SmbHLH088
SmbHLH094
SmbHLH095
SmbHLH086
SmbHLH083
SmbHLH100
MA 120602g0010
PpbHLH054
PpbHLH018
AtbHLH071
MA 141469g0010
MA 686524g0010
evm 27.model.AmTr v1.0 scaffold00001.504
AtbHLH057
AtbHLH070
MA 130776g0010
evm 27.model.AmTr v1.0 scaffold00008.115
OsbHLH049
OsbHLH050
OsbHLH048
AtbHLH096
OsbHLH047
OsbHLH044
OsbHLH045
AtbHLH094
OsbHLH046
Ia
PpbHLH056
PpbHLH084
PpbHLH014
evm 27.model.AmTr v1.0 scaffold00098.54
Mapoly0040s0112.1
MA 16454g0010
SmbHLH041
SmbHLH082
evm 27.model.AmTr v1.0 scaffold00029.122
evm 27.model.AmTr v1.0 scaffold00009.105
OsbHLH058
OsbHLH059
OsbHLH057
OsbHLH060
AtbHLH034
AtbHLH104
Mv13735RA
AtbHLH105
AtbHLH115
SmbHLH034
SmbHLH073
kfl00175 0110 v1.1
IVc
evm 27.model.AmTr v1.0 scaffold00010.404
OsbHLH063
OsbHLH062
OsbHLH061
AtbHLH047
MA 570300g0010
evm 27.model.AmTr v1.0 scaffold00041.167
evm 27.model.AmTr v1.0 scaffold00070.121
OsbHLH064
AtbHLH121
AtbHLH011
PpbHLH039
SmbHLH078
Mapoly0025s0044.1
PpbHLH042
PpbHLH085
OsbHLH001
IVb
evm 27.model.AmTr v1.0 scaffold00069.24
MA 45581g0020
evm 27.model.AmTr v1.0 scaffold00012.327
AtbHLH156
MA 18030g0010
SmbHLH016
SmbHLH060
Mapoly0088s0049.1
AtbHLH157
AtbHLH155
OsbHLH149
OsbHLH150
OsbHLH151
MA 10436087g0020
XIII
AtbHLH108
PpbHLH023
PpbHLH046
PpbHLH086
MA 10431176g0010
Mapoly0174s0004.1
PpbHLH067
evm 27.model.AmTr v1.0 scaffold00006.150
AtbHLH046
OsbHLH031
OsbHLH032
SmbHLH096
SmbHLH098
OsbHLH034
AtbHLH141
AtbHLH102
Mv09716RA
kfl00355 0070 v1.1
CvbHLH003
CrbHLH003
VcbHLH003
Va
Mv18707RA
kfl00506 0040 v1.1
CvbHLH002
CvbHLH004
CrbHLH002
VcbHLH002
CvbHLH001
CrbHLH001
VcbHLH001
AtbHLH051
AtbHLH106
AtbHLH107
OsbHLH041
evm 27.model.AmTr v1.0 scaffold00101.124
evm 27.model.AmTr v1.0 scaffold00021.51
OsbHLH035
OsbHLH036
OsbHLH037
OsbHLH042
OsbHLH043
evm 27.model.AmTr v1.0 scaffold00007.134
SmbHLH056
SmbHLH063
MA 10157g0010
MA 132680g0010
Mapoly0039s0068.1
kfl00061 0230 v1.1
SmbHLH046
SmbHLH049
evm 27.model.AmTr v1.0 scaffold00002.8
OsbHLH038
OsbHLH039
MA 65818g0010
evm 27.model.AmTr v1.0 scaffold00010.105
OsbHLH040
AtbHLH030
AtbHLH032
Vb

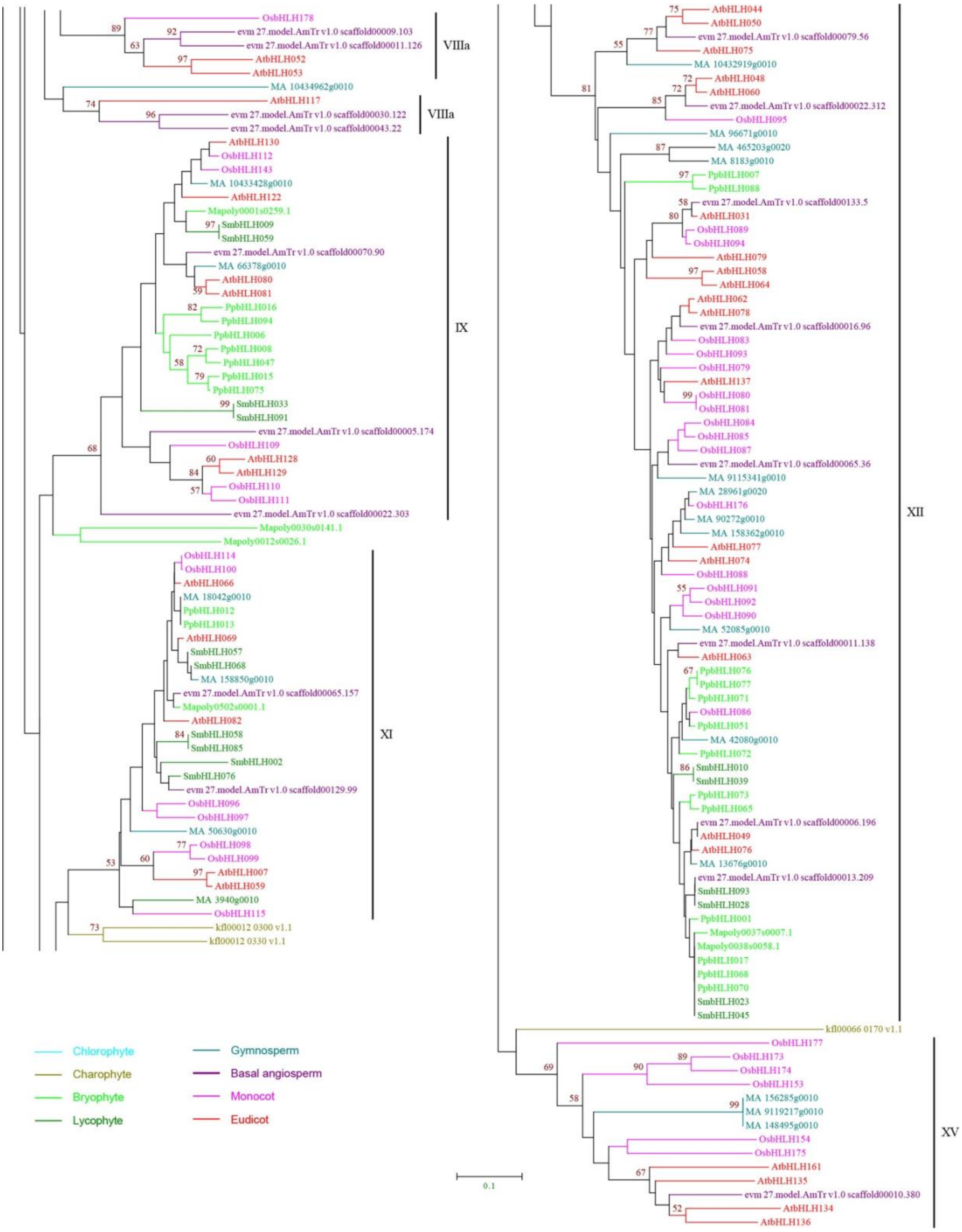

Data S1F. Phylogenetic relationships of basic helix-loop-helix (bHLH) TFs from *M. viride* and 10 evolutionarily representative species. The *M. viride* sequences were highlighted by triangles. Numbers on the major branches indicate bootstrap values (> 50%) in 1,000 replicates. The classification/clade was named according to a previous study.[2]

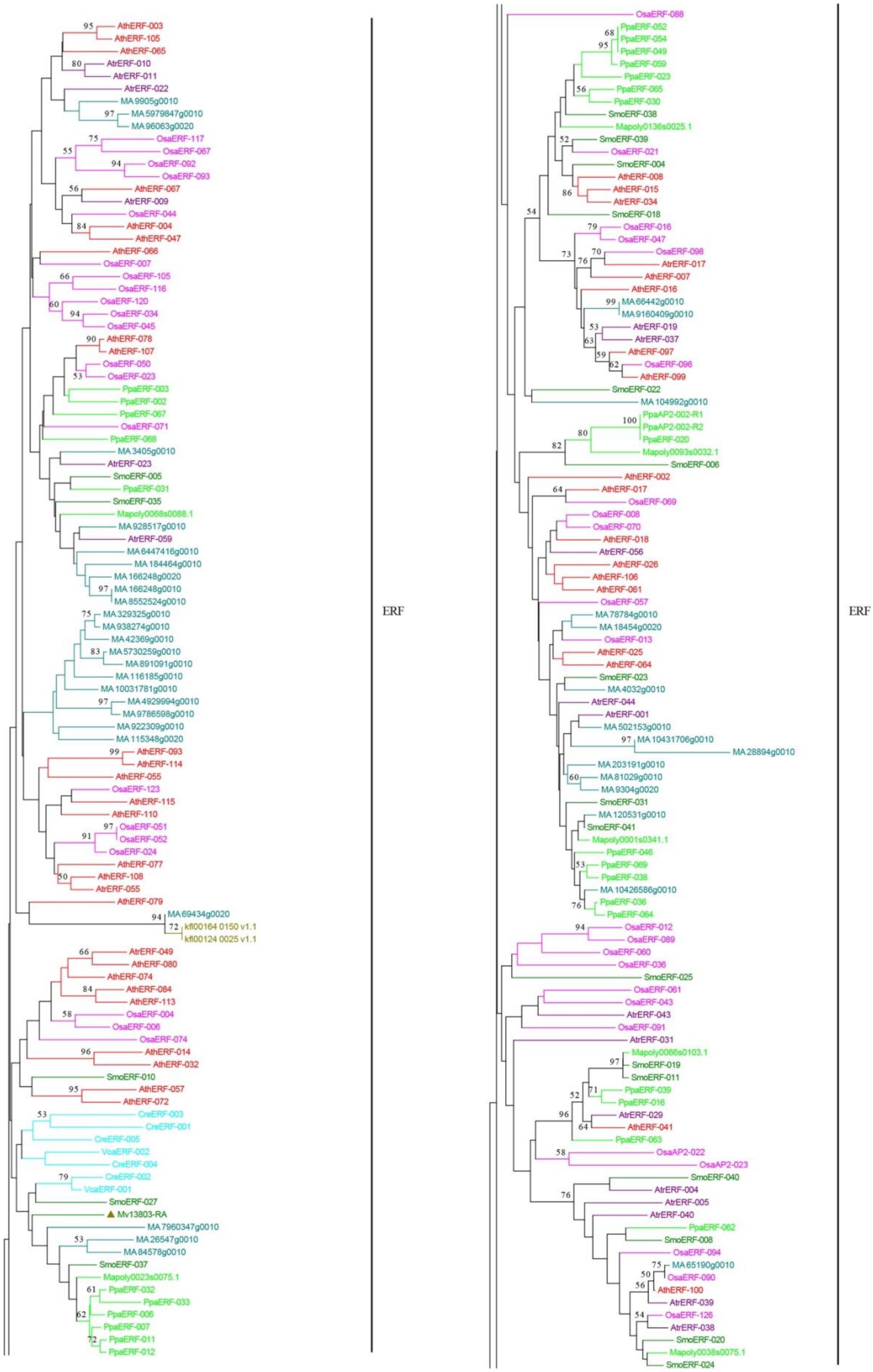
AthERF-003
AthERF-105
AthERF-065
AtrERF-010
AtrERF-011
AtrERF-022
MA 9905g0010
MA 5979847g0010
MA 96063g0020
OsaERF-117
OsaERF-067
OsaERF-092
OsaERF-093
AthERF-067
AtrERF-009
OsaERF-044
AthERF-004
AthERF-047
AthERF-066
OsaERF-007
OsaERF-105
OsaERF-116
OsaERF-120
OsaERF-034
OsaERF-045
AthERF-078
AthERF-107
OsaERF-050
OsaERF-023
PpaERF-003
PpaERF-002
PpaERF-067
OsaERF-071
PpaERF-068
MA 3405g0010
AtrERF-023
SmoERF-005
PpaERF-031
SmoERF-035
Mapoly0068s0088.1
MA 928517g0010
AtrERF-059
MA 6447416g0010
MA 184464g0010
MA 166248g0020
MA 166248g0010
MA 8552524g0010
MA 329325g0010
MA 938274g0010
MA 42369g0010
MA 5730259g0010
MA 891091g0010
MA 116185g0010
MA 10031781g0010
MA 4929994g0010
MA 9786598g0010
MA 922309g0010
MA 115348g0020
AthERF-093
AthERF-114
AthERF-055
OsaERF-123
AthERF-115
AthERF-110
OsaERF-051
OsaERF-052
OsaERF-024
AthERF-077
AthERF-108
AtrERF-055
AthERF-079
MA 69434g0020
kfl00164 0150 v1.1
kfl00124 0025 v1.1
AtrERF-049
AthERF-080
AthERF-074
AthERF-084
AthERF-113
OsaERF-004
OsaERF-006
OsaERF-074
AthERF-014
AthERF-032
SmoERF-010
AthERF-057
AthERF-072
CreERF-003
CreERF-001
CreERF-005
VcaERF-002
CreERF-004
CreERF-002
VcaERF-001
SmoERF-027
Mv13803-RA
MA 7960347g0010
MA 26547g0010
MA 84578g0010
SmoERF-037
Mapoly0023s0075.1
PpaERF-032
PpaERF-033
PpaERF-006
PpaERF-007
PpaERF-011
PpaERF-012
ERF
OsaERF-088
PpaERF-052
PpaERF-054
PpaERF-049
PpaERF-059
PpaERF-023
PpaERF-065
PpaERF-030
SmoERF-038
Mapoly0136s0025.1
SmoERF-039
OsaERF-021
SmoERF-004
AthERF-008
AthERF-015
AtrERF-034
SmoERF-018
OsaERF-016
OsaERF-047
OsaERF-098
AtrERF-017
AthERF-007
AthERF-016
MA 66442g0010
MA 9160409g0010
AtrERF-019
AtrERF-037
AthERF-097
OsaERF-096
AthERF-099
SmoERF-022
MA 104992g0010
PpaAP2-002-R1
PpaAP2-002-R2
PpaERF-020
Mapoly0093s0032.1
SmoERF-006
AthERF-002
AthERF-017
OsaERF-069
OsaERF-008
OsaERF-070
AthERF-018
AtrERF-056
AthERF-026
AthERF-106
AthERF-061
OsaERF-057
MA 78784g0010
MA 18454g0020
OsaERF-013
AthERF-025
AthERF-064
SmoERF-023
MA 4032g0010
AtrERF-044
AtrERF-001
MA 502153g0010
MA 10431706g0010
MA 28894g0010
MA 203191g0010
MA 81029g0010
MA 9304g0020
SmoERF-031
MA 120531g0010
SmoERF-041
Mapoly0001s0341.1
PpaERF-046
PpaERF-069
PpaERF-038
MA 10426586g0010
PpaERF-036
PpaERF-064
OsaERF-012
OsaERF-089
OsaERF-060
OsaERF-036
SmoERF-025
OsaERF-061
OsaERF-043
AtrERF-043
OsaERF-091
AtrERF-031
Mapoly0066s0103.1
SmoERF-019
SmoERF-011
PpaERF-039
PpaERF-016
AtrERF-029
AthERF-041
PpaERF-063
OsaAP2-022
OsaAP2-023
SmoERF-040
AtrERF-004
AtrERF-005
AtrERF-040
PpaERF-062
SmoERF-008
OsaERF-094
MA 65190g0010
OsaERF-090
AthERF-100
AtrERF-039
OsaERF-126
AtrERF-038
SmoERF-020
Mapoly0038s0075.1
SmoERF-024
ERF

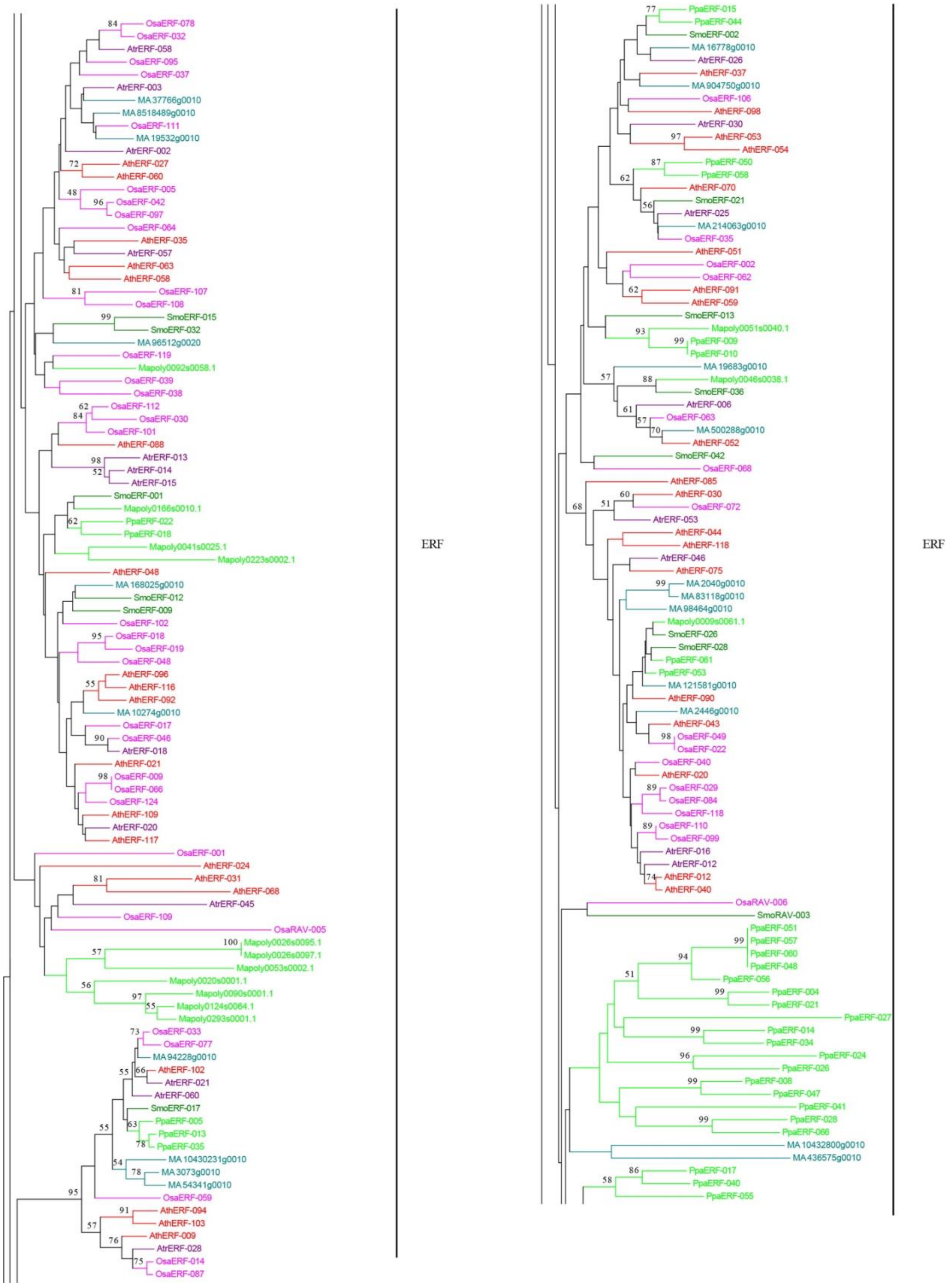
OsaERF-078
OsaERF-032
AtrERF-058
OsaERF-095
OsaERF-037
AtrERF-003
MA 37766g0010
MA 8518489g0010
OsaERF-111
MA 19532g0010
AtrERF-002
AthERF-027
AthERF-060
OsaERF-005
OsaERF-042
OsaERF-097
OsaERF-064
AthERF-035
AtrERF-057
AthERF-063
AthERF-058
OsaERF-107
OsaERF-108
SmoERF-015
SmoERF-032
MA 96512g0020
OsaERF-119
Mapoly0092s0058.1
OsaERF-039
OsaERF-038
OsaERF-112
OsaERF-030
OsaERF-101
AthERF-088
AtrERF-013
AtrERF-014
AtrERF-015
SmoERF-001
Mapoly0166s0010.1
PpaERF-022
PpaERF-018
Mapoly0041s0025.1
Mapoly0223s0002.1
AthERF-048
MA 168025g0010
SmoERF-012
SmoERF-009
OsaERF-102
OsaERF-018
OsaERF-019
OsaERF-048
AthERF-096
AthERF-116
AthERF-092
MA 10274g0010
OsaERF-017
OsaERF-046
AtrERF-018
AthERF-021
OsaERF-009
OsaERF-066
OsaERF-124
AthERF-109
AtrERF-020
AthERF-117
OsaERF-001
AthERF-024
AthERF-031
AthERF-068
AtrERF-045
OsaERF-109
OsaRAV-005
Mapoly0026s0095.1
Mapoly0026s0097.1
Mapoly0053s0002.1
Mapoly0020s0001.1
Mapoly0090s0001.1
Mapoly0124s0064.1
Mapoly0293s0001.1
OsaERF-033
OsaERF-077
MA 94228g0010
AthERF-102
AtrERF-021
AtrERF-060
SmoERF-017
PpaERF-005
PpaERF-013
PpaERF-035
MA 10430231g0010
MA 3073g0010
MA 54341g0010
OsaERF-059
AthERF-094
AthERF-103
AthERF-009
AtrERF-028
OsaERF-014
OsaERF-087
ERF
PpaERF-015
PpaERF-044
SmoERF-002
MA 16778g0010
AtrERF-026
AthERF-037
MA 904750g0010
OsaERF-106
AthERF-098
AtrERF-030
AthERF-053
AthERF-054
PpaERF-050
PpaERF-058
AthERF-070
SmoERF-021
AtrERF-025
MA 214063g0010
OsaERF-035
AthERF-051
OsaERF-002
OsaERF-062
AthERF-091
AthERF-059
SmoERF-013
Mapoly0051s0040.1
PpaERF-009
PpaERF-010
MA 19683g0010
Mapoly0046s0038.1
SmoERF-036
AtrERF-006
OsaERF-063
MA 500288g0010
AthERF-052
SmoERF-042
OsaERF-068
AthERF-085
AthERF-030
OsaERF-072
AtrERF-053
AthERF-044
AthERF-118
AtrERF-046
AthERF-075
MA 2040g0010
MA 83118g0010
MA 98464g0010
Mapoly0009s0061.1
SmoERF-026
SmoERF-028
PpaERF-061
PpaERF-053
MA 121581g0010
AthERF-090
MA 2446g0010
AthERF-043
OsaERF-049
OsaERF-022
OsaERF-040
AthERF-020
OsaERF-029
OsaERF-084
OsaERF-118
OsaERF-110
OsaERF-099
AtrERF-016
AtrERF-012
AthERF-012
AthERF-040
OsaRAV-006
SmoRAV-003
PpaERF-051
PpaERF-057
PpaERF-060
PpaERF-048
PpaERF-056
PpaERF-004
PpaERF-021
PpaERF-027
PpaERF-014
PpaERF-034
PpaERF-024
PpaERF-026
PpaERF-008
PpaERF-047
PpaERF-041
PpaERF-028
PpaERF-066
MA 10432800g0010
MA 436575g0010
PpaERF-017
PpaERF-040
PpaERF-055
ERF

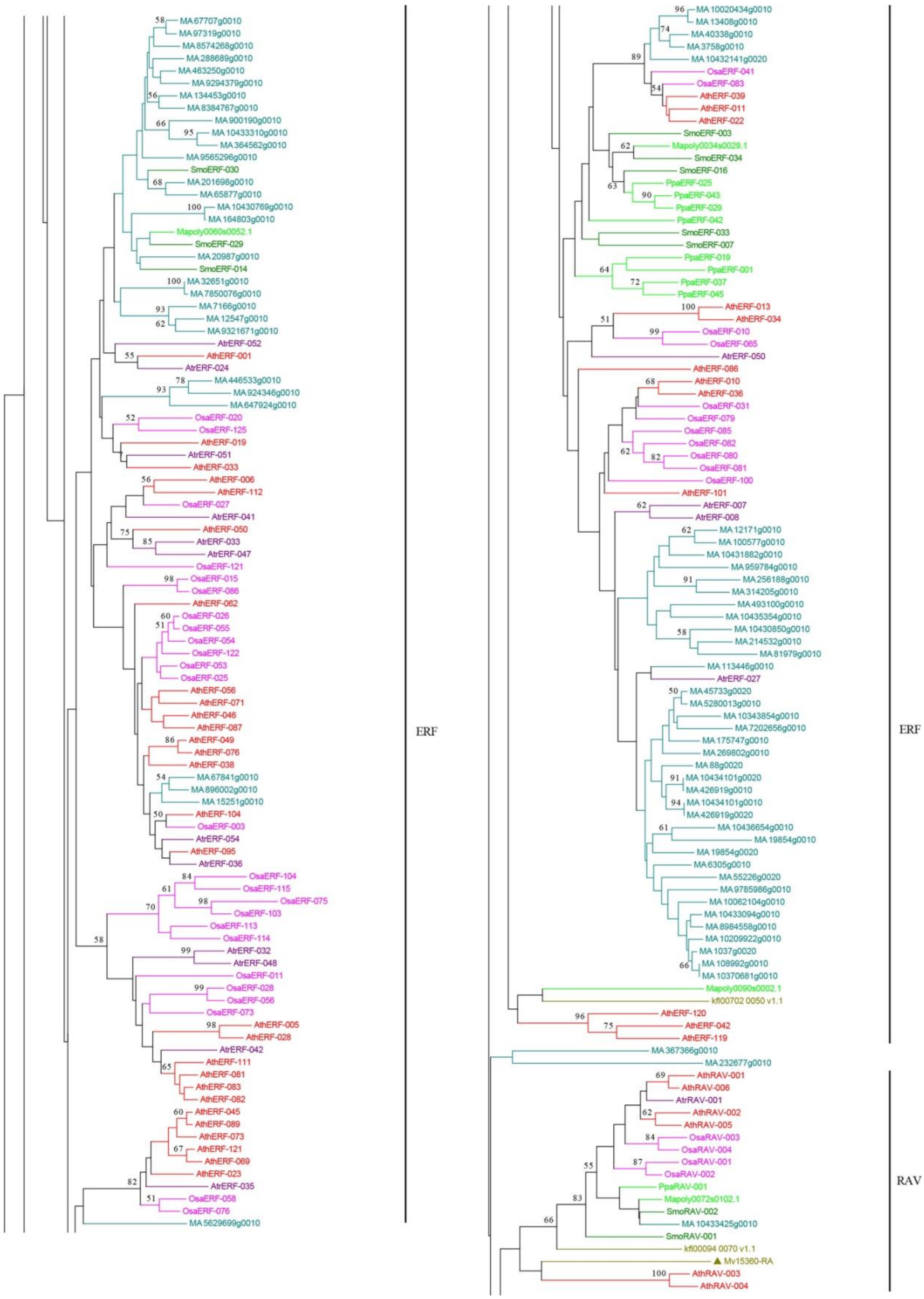
MA 67707g0010
MA 97319g0010
MA 8574268g0010
MA 288689g0010
MA 463250g0010
MA 9294379g0010
MA 134453g0010
MA 8384767g0010
MA 900190g0010
MA 10433310g0010
MA 364562g0010
MA 9565296g0010
SmoERF-030
MA 201698g0010
MA 65877g0010
MA 10430769g0010
MA 164803g0010
Mapoly0060s0052.1
SmoERF-029
MA 20987g0010
SmoERF-014
MA 32651g0010
MA 7850076g0010
MA 7166g0010
MA 12547g0010
MA 9321671g0010
AtrERF-052
AthERF-001
AtrERF-024
MA 446533g0010
MA 924346g0010
MA 647924g0010
OsaERF-020
OsaERF-125
AthERF-019
AtrERF-051
AthERF-033
AthERF-006
AthERF-112
OsaERF-027
AtrERF-041
AthERF-050
AtrERF-033
AtrERF-047
OsaERF-121
OsaERF-015
OsaERF-086
AthERF-062
OsaERF-026
OsaERF-055
OsaERF-054
OsaERF-122
OsaERF-053
OsaERF-025
AthERF-056
AthERF-071
AthERF-046
AthERF-087
AthERF-049
AthERF-076
AthERF-038
MA 67841g0010
MA 896002g0010
MA 15251g0010
AthERF-104
OsaERF-003
AtrERF-054
AthERF-095
AtrERF-036
OsaERF-104
OsaERF-115
OsaERF-075
OsaERF-103
OsaERF-113
OsaERF-114
AtrERF-032
AtrERF-048
OsaERF-011
OsaERF-028
OsaERF-056
OsaERF-073
AthERF-005
AthERF-028
AtrERF-042
AthERF-111
AthERF-081
AthERF-083
AthERF-082
AthERF-045
AthERF-089
AthERF-073
AthERF-121
AthERF-069
AthERF-023
AtrERF-035
OsaERF-058
OsaERF-076
MA 5629699g0010
ERF
MA 10020434g0010
MA 13408g0010
MA 40338g0010
MA 3758g0010
MA 10432141g0020
OsaERF-041
OsaERF-083
AthERF-039
AthERF-011
AthERF-022
SmoERF-003
Mapoly0034s0029.1
SmoERF-034
SmoERF-016
PpaERF-025
PpaERF-043
PpaERF-029
PpaERF-042
SmoERF-033
SmoERF-007
PpaERF-019
PpaERF-001
PpaERF-037
PpaERF-045
AthERF-013
AthERF-034
OsaERF-010
OsaERF-065
AtrERF-050
AthERF-086
AthERF-010
AthERF-036
OsaERF-031
OsaERF-079
OsaERF-085
OsaERF-082
OsaERF-080
OsaERF-081
OsaERF-100
AthERF-101
AtrERF-007
AtrERF-008
MA 12171g0010
MA 100577g0010
MA 10431882g0010
MA 959784g0010
MA 256188g0010
MA 314205g0010
MA 493100g0010
MA 10435354g0010
MA 10430850g0010
MA 214532g0010
MA 81979g0010
MA 113446g0010
AtrERF-027
MA 45733g0020
MA 5280013g0010
MA 10343854g0010
MA 7202656g0010
MA 175747g0010
MA 269802g0010
MA 88g0020
MA 10434101g0020
MA 426919g0010
MA 10434101g0010
MA 426919g0020
MA 10436654g0010
MA 19854g0010
MA 19854g0020
MA 6305g0010
MA 55226g0020
MA 9785986g0010
MA 10062104g0010
MA 10433094g0010
MA 8984558g0010
MA 10209922g0010
MA 1037g0020
MA 108992g0010
MA 10370681g0010
Mapoly0090s0002.1
kfl00702 0050 v1.1
AthERF-120
AthERF-042
AthERF-119
ERF
MA 367366g0010
MA 232677g0010
AthRAV-001
AthRAV-006
AtrRAV-001
AthRAV-002
AthRAV-005
OsaRAV-003
OsaRAV-004
OsaRAV-001
OsaRAV-002
PpaRAV-001
Mapoly0072s0102.1
SmoRAV-002
MA 10433425g0010
SmoRAV-001
kfl00094 0070 v1.1
Mv15360-RA
AthRAV-003
AthRAV-004
RAV

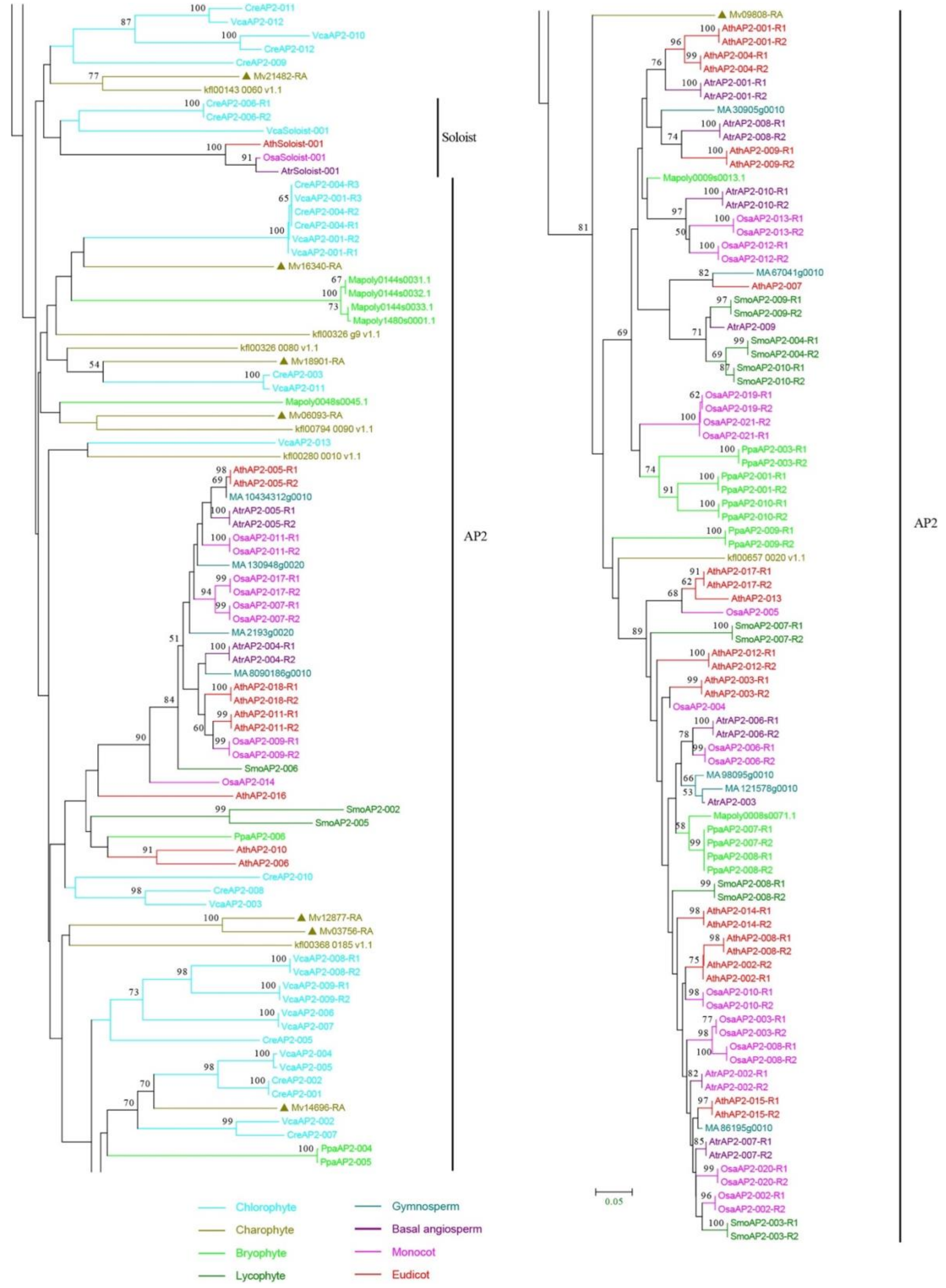

Data S1G. Phylogenetic relationships of APETALA 2/ERE binding factor (AP2/ERF) TFs from *M. viride* and 10 evolutionarily representative species. The *M. viride* sequences were highlighted by triangles. Numbers on the major branches indicate bootstrap values (> 50%) in 1,000 replicates. The classification/clade was named based on previous studies.[3-6]

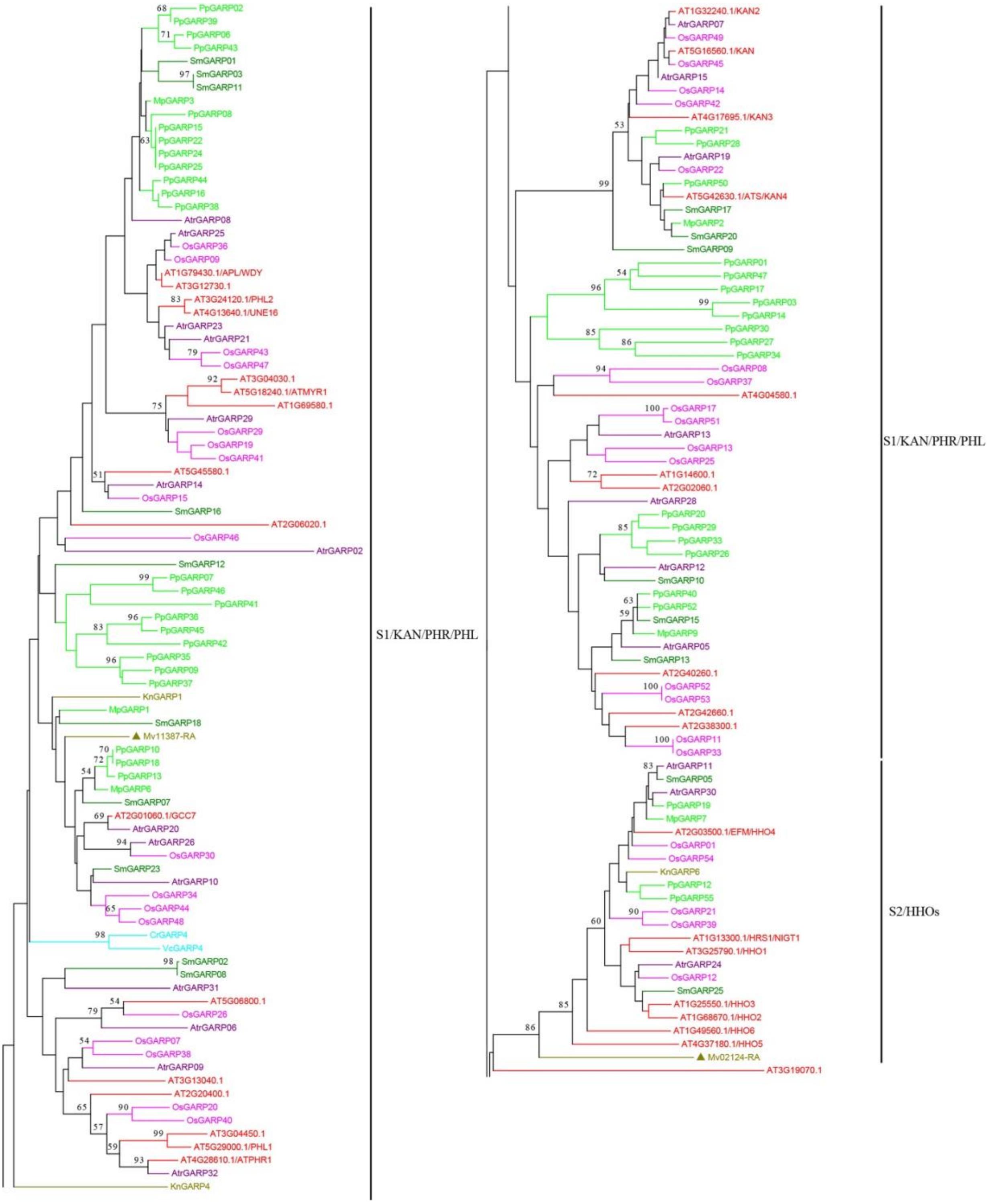
S1/KAN/PHR/PHL
S1/KAN/PHR/PHL
S2/HHOs
PpGARP02
PpGARP39
PpGARP06
PpGARP43
SmGARP01
SmGARP03
SmGARP11
MpGARP3
PpGARP08
PpGARP15
PpGARP22
PpGARP24
PpGARP25
PpGARP44
PpGARP16
PpGARP38
AtrGARP08
AtrGARP25
OsGARP36
OsGARP09
AT1G79430.1/APL/WDY
AT3G12730.1
AT3G24120.1/PHL2
AT4G13640.1/UNE16
AtrGARP23
AtrGARP21
OsGARP43
OsGARP47
AT3G04030.1
AT5G18240.1/ATMYR1
AT1G69580.1
AtrGARP29
OsGARP29
OsGARP19
OsGARP41
AT5G45580.1
AtrGARP14
OsGARP15
SmGARP16
AT2G06020.1
OsGARP46
AtrGARP02
SmGARP12
PpGARP07
PpGARP46
PpGARP41
PpGARP36
PpGARP45
PpGARP42
PpGARP35
PpGARP09
PpGARP37
KnGARP1
MpGARP1
SmGARP18
Mv11387-RA
PpGARP10
PpGARP18
PpGARP13
MpGARP6
SmGARP07
AT2G01060.1/GCC7
AtrGARP20
AtrGARP26
OsGARP30
SmGARP23
AtrGARP10
OsGARP34
OsGARP44
OsGARP48
CrGARP4
VcGARP4
SmGARP02
SmGARP08
AtrGARP31
AT5G06800.1
OsGARP26
AtrGARP06
OsGARP07
OsGARP38
AtrGARP09
AT3G13040.1
AT2G20400.1
OsGARP20
OsGARP40
AT3G04450.1
AT5G29000.1/PHL1
AT4G28610.1/ATPHR1
AtrGARP32
KnGARP4
AT1G32240.1/KAN2
AtrGARP07
OsGARP49
AT5G16560.1/KAN
OsGARP45
AtrGARP15
OsGARP14
OsGARP42
AT4G17695.1/KAN3
PpGARP21
PpGARP28
AtrGARP19
OsGARP22
PpGARP50
AT5G42630.1/ATS/KAN4
SmGARP17
MpGARP2
SmGARP20
SmGARP09
PpGARP01
PpGARP47
PpGARP17
PpGARP03
PpGARP14
PpGARP30
PpGARP27
PpGARP34
OsGARP08
OsGARP37
AT4G04580.1
OsGARP17
OsGARP51
AtrGARP13
OsGARP13
OsGARP25
AT1G14600.1
AT2G02060.1
AtrGARP28
PpGARP20
PpGARP29
PpGARP33
PpGARP26
AtrGARP12
SmGARP10
PpGARP40
PpGARP52
SmGARP15
MpGARP9
AtrGARP05
SmGARP13
AT2G40260.1
OsGARP52
OsGARP53
AT2G42660.1
AT2G38300.1
OsGARP11
OsGARP33
AtrGARP11
SmGARP05
AtrGARP30
PpGARP19
MpGARP7
AT2G03500.1/EFM/HHO4
OsGARP01
OsGARP54
KnGARP6
PpGARP12
PpGARP55
OsGARP21
OsGARP39
AT1G13300.1/HRS1/NIGT1
AT3G25790.1/HHO1
AtrGARP24
OsGARP12
SmGARP25
AT1G25550.1/HHO3
AT1G68670.1/HHO2
AT1G49560.1/HHO6
AT4G37180.1/HHO5
Mv02124-RA
AT3G19070.1

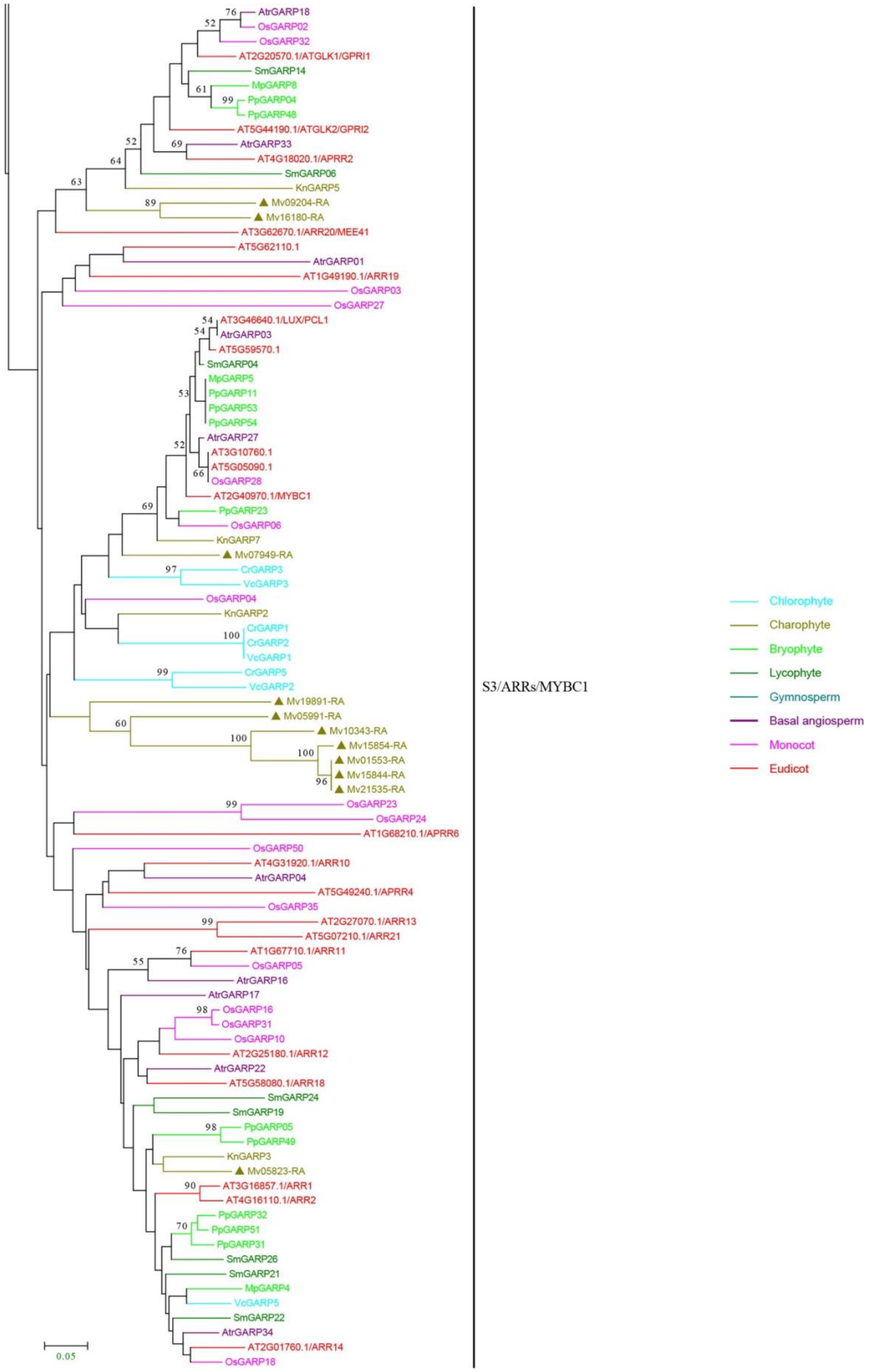
AtrGARP18
OsGARP02
OsGARP32
AT2G20570.1/ATGLK1/GPRI1
SmGARP14
MpGARP8
PpGARP04
PpGARP48
AT5G44190.1/ATGLK2/GPRI2
AtrGARP33
AT4G18020.1/APRR2
SmGARP06
KnGARP5
Mv09204-RA
Mv16180-RA
AT3G62670.1/ARR20/MEE41
AT5G62110.1
AtrGARP01
AT1G49190.1/ARR19
OsGARP03
OsGARP27
AT3G46640.1/LUX/PCL1
AtrGARP03
AT5G59570.1
SmGARP04
MpGARP5
PpGARP11
PpGARP53
PpGARP54
AtrGARP27
AT3G10760.1
AT5G05090.1
OsGARP28
AT2G40970.1/MYBC1
PpGARP23
OsGARP06
KnGARP7
Mv07949-RA
CrGARP3
VcGARP3
OsGARP04
KnGARP2
CrGARP1
CrGARP2
VcGARP1
CrGARP5
VcGARP2
Mv19891-RA
Mv05991-RA
Mv10343-RA
Mv15854-RA
Mv01553-RA
Mv15844-RA
Mv21535-RA
OsGARP23
OsGARP24
AT1G68210.1/APRR6
OsGARP50
AT4G31920.1/ARR10
AtrGARP04
AT5G49240.1/APRR4
OsGARP35
AT2G27070.1/ARR13
AT5G07210.1/ARR21
AT1G67710.1/ARR11
OsGARP05
AtrGARP16
AtrGARP17
OsGARP16
OsGARP31
OsGARP10
AT2G25180.1/ARR12
AtrGARP22
AT5G58080.1/ARR18
SmGARP24
SmGARP19
PpGARP05
PpGARP49
KnGARP3
Mv05823-RA
AT3G16857.1/ARR1
AT4G16110.1/ARR2
PpGARP32
PpGARP51
PpGARP31
SmGARP26
SmGARP21
MpGARP4
VcGARP5
SmGARP22
AtrGARP34
AT2G01760.1/ARR14
OsGARP18
0.05
S3/ARRs/MYBC1
Chlorophyte
Charophyte
Bryophyte
Lycophyte
Gymnosperm
Basal angiosperm
Monocot
Eudicot

Data S1H. Phylogenetic relationships of GARP TFs from *M. viride* and 10 evolutionarily representative species. The *M. viride* sequences were highlighted by triangles. Numbers on the major branches indicate bootstrap values (> 50%) in 1,000 replicates. The classification/clade was named based on a previous study.[7]

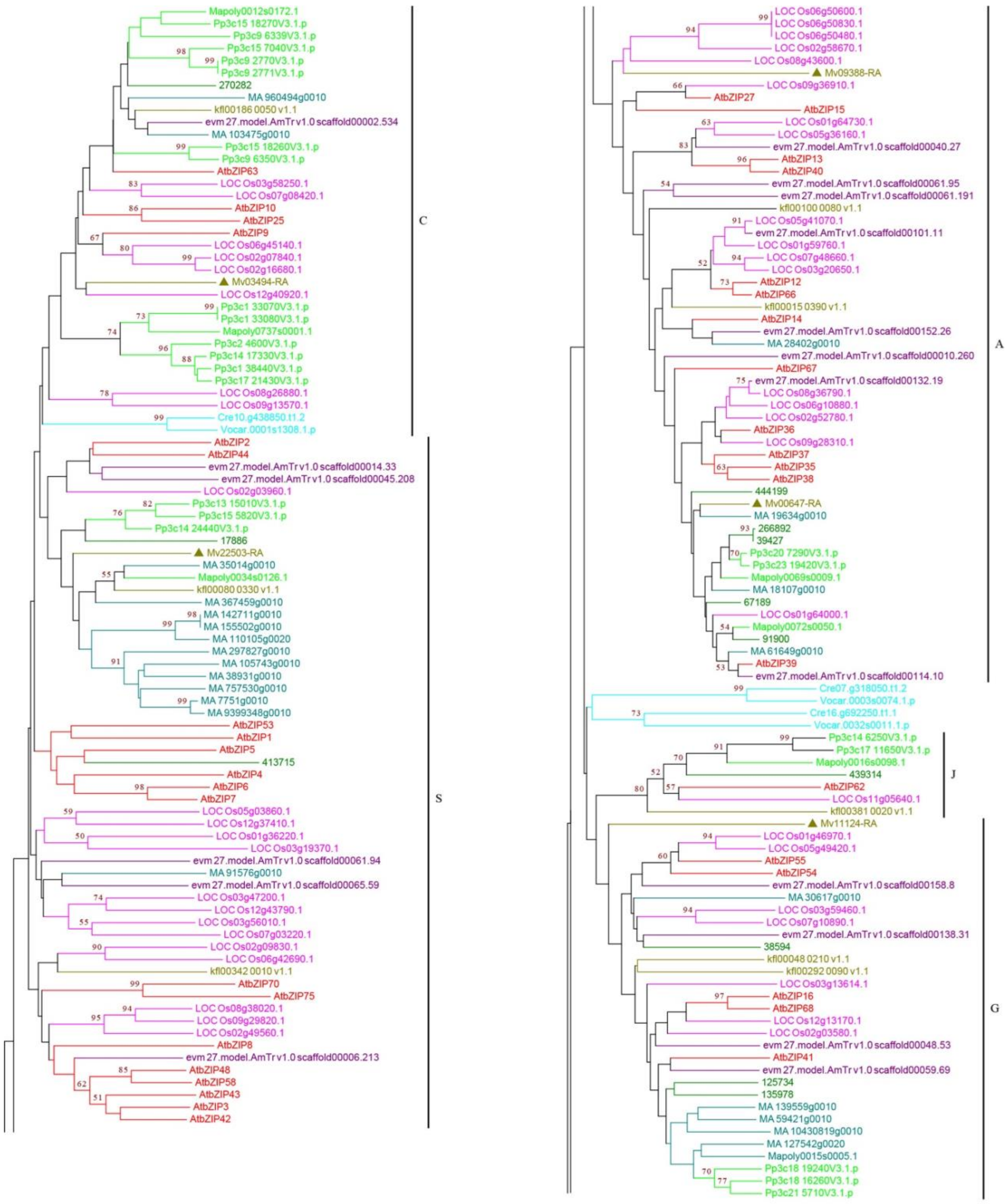
Mapoly0012s0172.1
Pp3c15 18270V3.1.p
Pp3c9 6339V3.1.p
Pp3c15 7040V3.1.p
Pp3c9 2770V3.1.p
Pp3c9 2771V3.1.p
270282
MA 960494g0010
kfl00186 0050 v1.1
evm 27.model.AmTr v1.0 scaffold00002.534
MA 103475g0010
Pp3c15 18260V3.1.p
Pp3c9 6350V3.1.p
AtbZIP63
LOC Os03g58250.1
LOC Os07g08420.1
AtbZIP10
AtbZIP25
AtbZIP9
LOC Os06g45140.1
LOC Os02g07840.1
LOC Os02g16680.1
Mv03494-RA
LOC Os12g40920.1
Pp3c1 33070V3.1.p
Pp3c1 33080V3.1.p
Mapoly0737s0001.1
Pp3c2 4600V3.1.p
Pp3c14 17330V3.1.p
Pp3c1 38440V3.1.p
Pp3c17 21430V3.1.p
LOC Os08g26880.1
LOC Os09g13570.1
Cre10.g438850.t1.2
Vocar.0001s1308.1.p
C
AtbZIP2
AtbZIP44
evm 27.model.AmTr v1.0 scaffold00014.33
evm 27.model.AmTr v1.0 scaffold00045.208
LOC Os02g03960.1
Pp3c13 15010V3.1.p
Pp3c15 5820V3.1.p
Pp3c14 24440V3.1.p
17886
Mv22503-RA
MA 35014g0010
Mapoly0034s0126.1
kfl00080 0330 v1.1
MA 367459g0010
MA 142711g0010
MA 155502g0010
MA 110105g0020
MA 297827g0010
MA 105743g0010
MA 38931g0010
MA 757530g0010
MA 7751g0010
MA 9399348g0010
AtbZIP53
AtbZIP1
AtbZIP5
413715
AtbZIP4
AtbZIP6
AtbZIP7
LOC Os05g03860.1
LOC Os12g37410.1
LOC Os01g36220.1
LOC Os03g19370.1
evm 27.model.AmTr v1.0 scaffold00061.94
MA 91576g0010
evm 27.model.AmTr v1.0 scaffold00065.59
LOC Os03g47200.1
LOC Os12g43790.1
LOC Os03g56010.1
LOC Os07g03220.1
LOC Os02g09830.1
LOC Os06g42690.1
kfl00342 0010 v1.1
AtbZIP70
AtbZIP75
LOC Os08g38020.1
LOC Os09g29820.1
LOC Os02g49560.1
AtbZIP8
evm 27.model.AmTr v1.0 scaffold00006.213
AtbZIP48
AtbZIP58
AtbZIP43
AtbZIP3
AtbZIP42
S
LOC Os06g50600.1
LOC Os06g50830.1
LOC Os06g50480.1
LOC Os02g58670.1
LOC Os08g43600.1
Mv09388-RA
LOC Os09g36910.1
AtbZIP27
AtbZIP15
LOC Os01g64730.1
LOC Os05g36160.1
evm 27.model.AmTr v1.0 scaffold00040.27
AtbZIP13
AtbZIP40
evm 27.model.AmTr v1.0 scaffold00061.95
evm 27.model.AmTr v1.0 scaffold00061.191
kfl00100 0080 v1.1
LOC Os05g41070.1
evm 27.model.AmTr v1.0 scaffold00101.11
LOC Os01g59760.1
LOC Os07g48660.1
LOC Os03g20650.1
AtbZIP12
AtbZIP66
kfl00015 0390 v1.1
AtbZIP14
evm 27.model.AmTr v1.0 scaffold00152.26
MA 28402g0010
evm 27.model.AmTr v1.0 scaffold00010.260
AtbZIP67
evm 27.model.AmTr v1.0 scaffold00132.19
LOC Os08g36790.1
LOC Os06g10880.1
LOC Os02g52780.1
AtbZIP36
LOC Os09g28310.1
AtbZIP37
AtbZIP35
AtbZIP38
444199
Mv00647-RA
MA 19634g0010
266892
39427
Pp3c20 7290V3.1.p
Pp3c23 19420V3.1.p
Mapoly0069s0009.1
MA 18107g0010
67189
LOC Os01g64000.1
Mapoly0072s0050.1
91900
MA 61649g0010
AtbZIP39
evm 27.model.AmTr v1.0 scaffold00114.10
A
Cre07.g318050.t1.2
Vocar.0003s0074.1.p
Cre16.g692250.t1.1
Vocar.0032s0011.1.p
Pp3c14 6250V3.1.p
Pp3c17 11650V3.1.p
Mapoly0016s0098.1
439314
AtbZIP62
LOC Os11g05640.1
kfl00381 0020 v1.1
J
Mv11124-RA
LOC Os01g46970.1
LOC Os05g49420.1
AtbZIP55
AtbZIP54
evm 27.model.AmTr v1.0 scaffold00158.8
MA 30617g0010
LOC Os03g59460.1
LOC Os07g10890.1
evm 27.model.AmTr v1.0 scaffold00138.31
38594
kfl00048 0210 v1.1
kfl00292 0090 v1.1
LOC Os03g13614.1
AtbZIP16
AtbZIP68
LOC Os12g13170.1
LOC Os02g03580.1
evm 27.model.AmTr v1.0 scaffold00048.53
AtbZIP41
evm 27.model.AmTr v1.0 scaffold00059.69
125734
135978
MA 139559g0010
MA 59421g0010
MA 10430819g0010
MA 127542g0020
Mapoly0015s0005.1
Pp3c18 19240V3.1.p
Pp3c18 16260V3.1.p
Pp3c21 5710V3.1.p
G

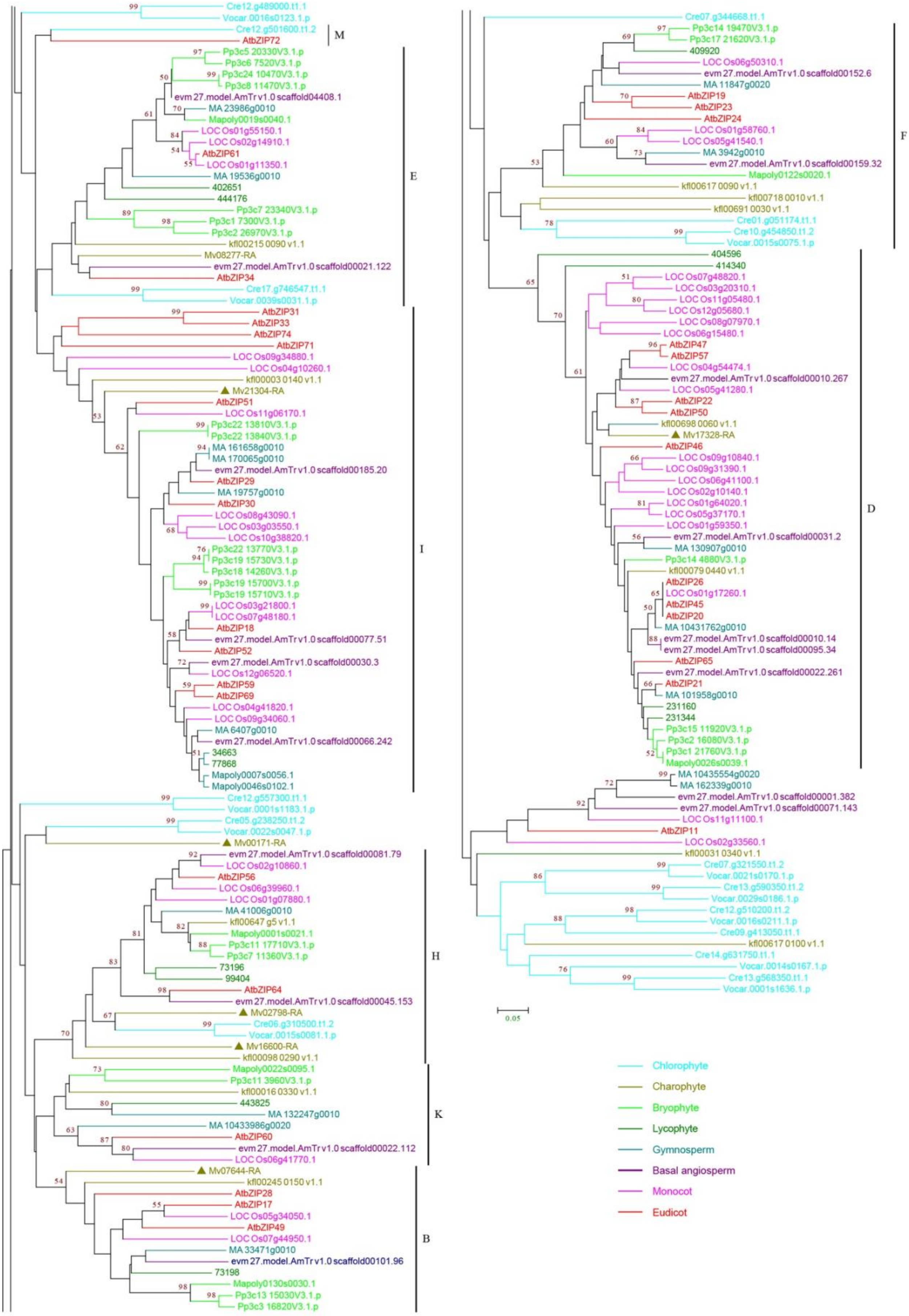
Cre12.g489000.t1.1
Vocar.0016s0123.1.p
Cre12.g501600.t1.2
AtbZIP72
M
Pp3c5 20330V3.1.p
Pp3c6 7520V3.1.p
Pp3c24 10470V3.1.p
Pp3c8 11470V3.1.p
evm 27.model.AmTr v1.0 scaffold04408.1
MA 23986g0010
Mapoly0019s0040.1
LOC Os01g55150.1
LOC Os02g14910.1
AtbZIP61
LOC Os01g11350.1
MA 19536g0010
402651
444176
Pp3c7 23340V3.1.p
Pp3c1 7300V3.1.p
Pp3c2 26970V3.1.p
kfl00215 0090 v1.1
Mv08277-RA
evm 27.model.AmTr v1.0 scaffold00021.122
AtbZIP34
Cre17.g746547.t1.1
Vocar.0039s0031.1.p
E
AtbZIP31
AtbZIP33
AtbZIP74
AtbZIP71
LOC Os09g34880.1
LOC Os04g10260.1
kfl00003 0140 v1.1
Mv21304-RA
AtbZIP51
LOC Os11g06170.1
Pp3c22 13810V3.1.p
Pp3c22 13840V3.1.p
MA 161658g0010
MA 170065g0010
evm 27.model.AmTr v1.0 scaffold00185.20
AtbZIP29
MA 19757g0010
AtbZIP30
LOC Os08g43090.1
LOC Os03g03550.1
LOC Os10g38820.1
Pp3c22 13770V3.1.p
Pp3c19 15730V3.1.p
Pp3c18 14260V3.1.p
Pp3c19 15700V3.1.p
Pp3c19 15710V3.1.p
LOC Os03g21800.1
LOC Os07g48180.1
AtbZIP18
evm 27.model.AmTr v1.0 scaffold00077.51
AtbZIP52
evm 27.model.AmTr v1.0 scaffold00030.3
LOC Os12g06520.1
AtbZIP59
AtbZIP69
LOC Os04g41820.1
LOC Os09g34060.1
MA 6407g0010
evm 27.model.AmTr v1.0 scaffold00066.242
34663
77868
Mapoly0007s0056.1
Mapoly0046s0102.1
I
Cre12.g557300.t1.1
Vocar.0001s1183.1.p
Cre05.g238250.t1.2
Vocar.0022s0047.1.p
Mv00171-RA
evm 27.model.AmTr v1.0 scaffold00081.79
LOC Os02g10860.1
AtbZIP56
LOC Os06g39960.1
LOC Os01g07880.1
MA 41006g0010
kfl00647 g5 v1.1
Mapoly0001s0021.1
Pp3c11 17710V3.1.p
Pp3c7 11360V3.1.p
73196
99404
AtbZIP64
evm 27.model.AmTr v1.0 scaffold00045.153
Mv02798-RA
Cre06.g310500.t1.2
Vocar.0015s0081.1.p
Mv16600-RA
kfl00098 0290 v1.1
H
Mapoly0022s0095.1
Pp3c11 3960V3.1.p
kfl00016 0330 v1.1
443825
MA 132247g0010
MA 10433986g0020
AtbZIP60
evm 27.model.AmTr v1.0 scaffold00022.112
LOC Os06g41770.1
K
Mv07644-RA
kfl00245 0150 v1.1
AtbZIP28
AtbZIP17
LOC Os05g34050.1
AtbZIP49
LOC Os07g44950.1
MA 33471g0010
evm 27.model.AmTr v1.0 scaffold00101.96
73198
Mapoly0130s0030.1
Pp3c13 15030V3.1.p
Pp3c3 16820V3.1.p
B
Cre07.g344668.t1.1
Pp3c14 19470V3.1.p
Pp3c17 21620V3.1.p
409920
LOC Os06g50310.1
evm 27.model.AmTr v1.0 scaffold00152.6
MA 11847g0020
AtbZIP19
AtbZIP23
AtbZIP24
LOC Os01g58760.1
LOC Os05g41540.1
MA 3942g0010
evm 27.model.AmTr v1.0 scaffold00159.32
Mapoly0122s0020.1
kfl00617 0090 v1.1
kfl00718 0010 v1.1
kfl00691 0030 v1.1
Cre01.g051174.t1.1
Cre10.g454850.t1.2
Vocar.0015s0075.1.p
F
404596
414340
LOC Os07g48820.1
LOC Os03g20310.1
LOC Os11g05480.1
LOC Os12g05680.1
LOC Os08g07970.1
LOC Os06g15480.1
AtbZIP47
AtbZIP57
LOC Os04g54474.1
evm 27.model.AmTr v1.0 scaffold00010.267
LOC Os05g41280.1
AtbZIP22
AtbZIP50
kfl00698 0060 v1.1
Mv17328-RA
AtbZIP46
LOC Os09g10840.1
LOC Os09g31390.1
LOC Os06g41100.1
LOC Os02g10140.1
LOC Os01g64020.1
LOC Os05g37170.1
LOC Os01g59350.1
evm 27.model.AmTr v1.0 scaffold00031.2
MA 130907g0010
Pp3c14 4880V3.1.p
kfl00079 0440 v1.1
AtbZIP26
LOC Os01g17260.1
AtbZIP45
AtbZIP20
MA 10431762g0010
evm 27.model.AmTr v1.0 scaffold00010.14
evm 27.model.AmTr v1.0 scaffold00095.34
AtbZIP65
evm 27.model.AmTr v1.0 scaffold00022.261
AtbZIP21
MA 101958g0010
231160
231344
Pp3c15 11920V3.1.p
Pp3c2 16080V3.1.p
Pp3c1 21760V3.1.p
Mapoly0026s0039.1
D
MA 10435554g0020
MA 162339g0010
evm 27.model.AmTr v1.0 scaffold00001.382
evm 27.model.AmTr v1.0 scaffold00071.143
LOC Os11g11100.1
AtbZIP11
LOC Os02g33560.1
kfl00031 0340 v1.1
Cre07.g321550.t1.2
Vocar.0021s0170.1.p
Cre13.g590350.t1.2
Vocar.0029s0186.1.p
Cre12.g510200.t1.2
Vocar.0016s0211.1.p
Cre09.g413050.t1.1
kfl00617 0100 v1.1
Cre14.g631750.t1.1
Vocar.0014s0167.1.p
Cre13.g568350.t1.1
Vocar.0001s1636.1.p
0.05
Chlorophyte
Charophyte
Bryophyte
Lycophyte
Gymnosperm
Basal angiosperm
Monocot
Eudicot

Data S1I. Phylogenetic relationships of Basic Leucine Zipper (bZIP) TFs from *M. viride* and 10 evolutionarily representative species. The *M. viride* sequences were highlighted by triangles. Numbers on the major branches indicate bootstrap values (> 50%) in 1,000 replicates. The classification/clade was named based on previous studies.[8-9]

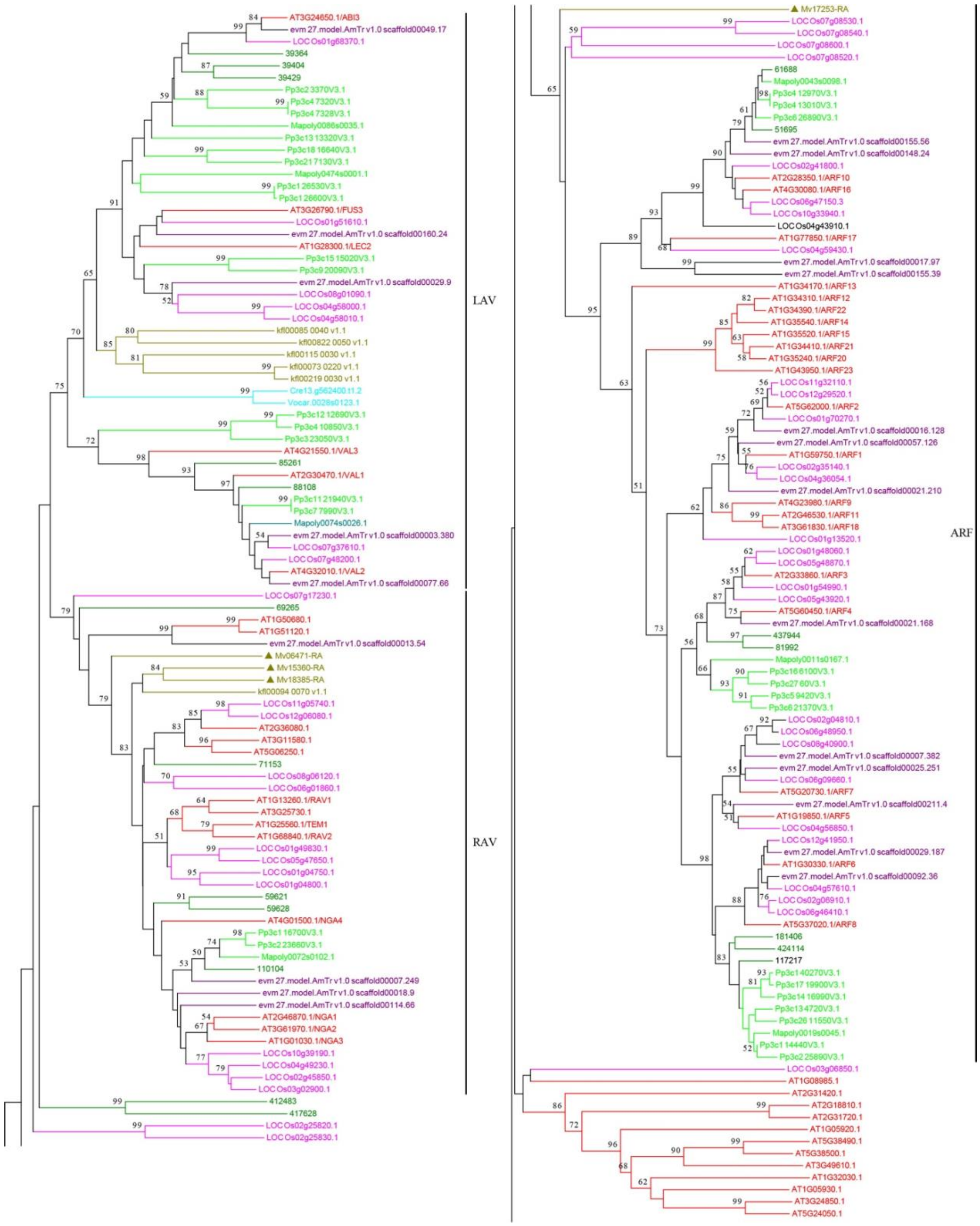
AT3G24650.1/ABI3
evm 27.model.AmTr v1.0 scaffold00049.17
LOC Os01g68370.1
39364
39404
39429
Pp3c2 3370V3.1
Pp3c4 7320V3.1
Pp3c4 7328V3.1
Mapoly0086s0035.1
Pp3c13 13320V3.1
Pp3c18 16640V3.1
Pp3c2 17130V3.1
Mapoly0474s0001.1
Pp3c1 26530V3.1
Pp3c1 26600V3.1
AT3G26790.1/FUS3
LOC Os01g51610.1
evm 27.model.AmTr v1.0 scaffold00160.24
AT1G28300.1/LEC2
Pp3c15 15020V3.1
Pp3c9 20090V3.1
evm 27.model.AmTr v1.0 scaffold00029.9
LOC Os08g01090.1
LOC Os04g58000.1
LOC Os04g58010.1
kfl00085 0040 v1.1
kfl00822 0050 v1.1
kfl00115 0030 v1.1
kfl00073 0220 v1.1
kfl00219 0030 v1.1
Cre13.g562400.t1.2
Vocar.0028s0123.1
Pp3c12 12690V3.1
Pp3c4 10850V3.1
Pp3c3 23050V3.1
AT4G21550.1/VAL3
85261
AT2G30470.1/VAL1
88108
Pp3c11 21940V3.1
Pp3c7 7990V3.1
Mapoly0074s0026.1
evm 27.model.AmTr v1.0 scaffold00003.380
LOC Os07g37610.1
LOC Os07g48200.1
AT4G32010.1/VAL2
evm 27.model.AmTr v1.0 scaffold00077.66
LAV
LOC Os07g17230.1
69265
AT1G50680.1
AT1G51120.1
evm 27.model.AmTr v1.0 scaffold00013.54
Mv06471-RA
Mv15360-RA
Mv18385-RA
kfl00094 0070 v1.1
LOC Os11g05740.1
LOC Os12g06080.1
AT2G36080.1
AT3G11580.1
AT5G06250.1
71153
LOC Os08g06120.1
LOC Os06g01860.1
AT1G13260.1/RAV1
AT3G25730.1
AT1G25560.1/TEM1
AT1G68840.1/RAV2
LOC Os01g49830.1
LOC Os05g47650.1
LOC Os01g04750.1
LOC Os01g04800.1
59621
59628
AT4G01500.1/NGA4
Pp3c1 16700V3.1
Pp3c2 23660V3.1
Mapoly0072s0102.1
110104
evm 27.model.AmTr v1.0 scaffold00007.249
evm 27.model.AmTr v1.0 scaffold00018.9
evm 27.model.AmTr v1.0 scaffold00114.66
AT2G46870.1/NGA1
AT3G61970.1/NGA2
AT1G01030.1/NGA3
LOC Os10g39190.1
LOC Os04g49230.1
LOC Os02g45850.1
LOC Os03g02900.1
RAV
412483
417628
LOC Os02g25820.1
LOC Os02g25830.1
Mv17253-RA
LOC Os07g08530.1
LOC Os07g08540.1
LOC Os07g08600.1
LOC Os07g08520.1
61688
Mapoly0043s0098.1
Pp3c4 12970V3.1
Pp3c4 13010V3.1
Pp3c6 26890V3.1
51695
evm 27.model.AmTr v1.0 scaffold00155.56
evm 27.model.AmTr v1.0 scaffold00148.24
LOC Os02g41800.1
AT2G28350.1/ARF10
AT4G30080.1/ARF16
LOC Os06g47150.3
LOC Os10g33940.1
LOC Os04g43910.1
AT1G77850.1/ARF17
LOC Os04g59430.1
evm 27.model.AmTr v1.0 scaffold00017.97
evm 27.model.AmTr v1.0 scaffold00155.39
AT1G34170.1/ARF13
AT1G34310.1/ARF12
AT1G34390.1/ARF22
AT1G35540.1/ARF14
AT1G35520.1/ARF15
AT1G34410.1/ARF21
AT1G35240.1/ARF20
AT1G43950.1/ARF23
LOC Os11g32110.1
LOC Os12g29520.1
AT5G62000.1/ARF2
LOC Os01g70270.1
evm 27.model.AmTr v1.0 scaffold00016.128
evm 27.model.AmTr v1.0 scaffold00057.126
AT1G59750.1/ARF1
LOC Os02g35140.1
LOC Os04g36054.1
evm 27.model.AmTr v1.0 scaffold00021.210
AT4G23980.1/ARF9
AT2G46530.1/ARF11
AT3G61830.1/ARF18
LOC Os01g13520.1
ARF
LOC Os01g48060.1
LOC Os05g48870.1
AT2G33860.1/ARF3
LOC Os01g54990.1
LOC Os05g43920.1
AT5G60450.1/ARF4
evm 27.model.AmTr v1.0 scaffold00021.168
437944
81992
Mapoly0011s0167.1
Pp3c16 6100V3.1
Pp3c27 60V3.1
Pp3c5 9420V3.1
Pp3c8 21370V3.1
LOC Os02g04810.1
LOC Os06g48950.1
LOC Os08g40900.1
evm 27.model.AmTr v1.0 scaffold00007.382
evm 27.model.AmTr v1.0 scaffold00025.251
LOC Os06g09660.1
AT5G20730.1/ARF7
evm 27.model.AmTr v1.0 scaffold00211.4
AT1G19850.1/ARF5
LOC Os04g56850.1
LOC Os12g41950.1
evm 27.model.AmTr v1.0 scaffold00029.187
AT1G30330.1/ARF6
evm 27.model.AmTr v1.0 scaffold00092.36
LOC Os04g57610.1
LOC Os02g06910.1
LOC Os06g46410.1
AT5G37020.1/ARF8
181406
424114
117217
Pp3c1 40270V3.1
Pp3c17 19900V3.1
Pp3c14 16990V3.1
Pp3c13 4720V3.1
Pp3c26 11550V3.1
Mapoly0019s0045.1
Pp3c1 14440V3.1
Pp3c2 25890V3.1
LOC Os03g06850.1
AT1G08985.1
AT2G31420.1
AT2G18810.1
AT2G31720.1
AT1G05920.1
AT5G38490.1
AT5G38500.1
AT3G49610.1
AT1G32030.1
AT1G05930.1
AT3G24850.1
AT5G24050.1

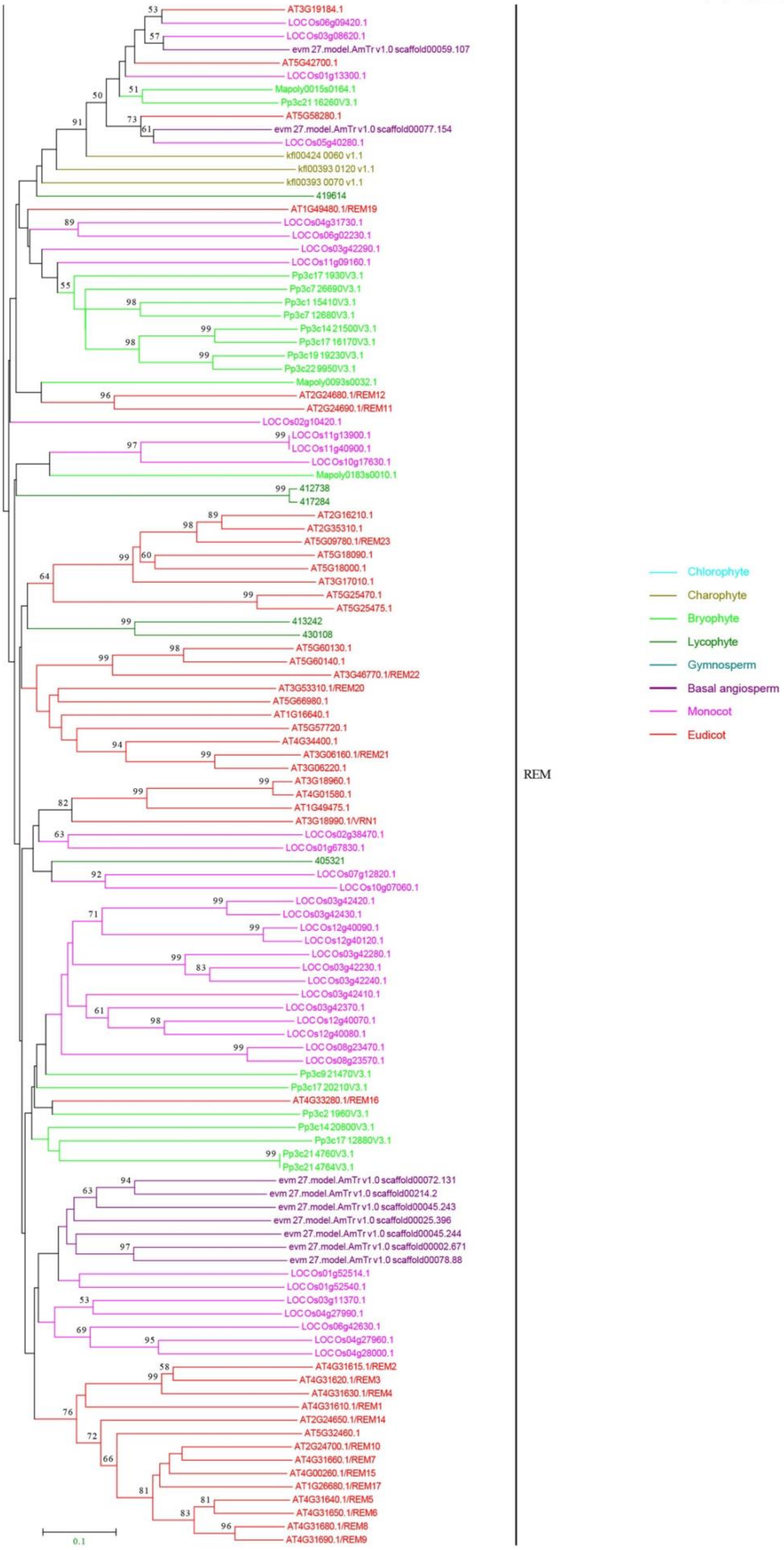
AT3G19184.1
LOC Os06g09420.1
LOC Os03g08620.1
evm 27.model.AmTr v1.0 scaffold00059.107
AT5G42700.1
LOC Os01g13300.1
Mapoly0015s0164.1
Pp3c21 16260V3.1
AT5G58280.1
evm 27.model.AmTr v1.0 scaffold00077.154
LOC Os05g40280.1
kfl00424 0060 v1.1
kfl00393 0120 v1.1
kfl00393 0070 v1.1
419614
AT1G49480.1/REM19
LOC Os04g31730.1
LOC Os06g02230.1
LOC Os03g42290.1
LOC Os11g09160.1
Pp3c17 1930V3.1
Pp3c7 26690V3.1
Pp3c1 15410V3.1
Pp3c7 12680V3.1
Pp3c14 21500V3.1
Pp3c17 16170V3.1
Pp3c19 19230V3.1
Pp3c22 9950V3.1
Mapoly0093s0032.1
AT2G24680.1/REM12
AT2G24690.1/REM11
LOC Os02g10420.1
LOC Os11g13900.1
LOC Os11g40900.1
LOC Os10g17630.1
Mapoly0183s0010.1
412738
417284
AT2G16210.1
AT2G35310.1
AT5G09780.1/REM23
AT5G18090.1
AT5G18000.1
AT3G17010.1
AT5G25470.1
AT5G25475.1
413242
430108
AT5G60130.1
AT5G60140.1
AT3G46770.1/REM22
AT3G53310.1/REM20
AT5G66980.1
AT1G16640.1
AT5G57720.1
AT4G34400.1
AT3G06160.1/REM21
AT3G06220.1
AT3G18960.1
AT4G01580.1
AT1G49475.1
AT3G18990.1/VRN1
LOC Os02g38470.1
LOC Os01g67830.1
405321
LOC Os07g12820.1
LOC Os10g07060.1
LOC Os03g42420.1
LOC Os03g42430.1
LOC Os12g40090.1
LOC Os12g40120.1
LOC Os03g42280.1
LOC Os03g42230.1
LOC Os03g42240.1
LOC Os03g42410.1
LOC Os03g42370.1
LOC Os12g40070.1
LOC Os12g40080.1
LOC Os08g23470.1
LOC Os08g23570.1
Pp3c9 21470V3.1
Pp3c17 20210V3.1
AT4G33280.1/REM16
Pp3c2 1960V3.1
Pp3c14 20800V3.1
Pp3c17 12880V3.1
Pp3c21 4760V3.1
Pp3c21 4764V3.1
evm 27.model.AmTr v1.0 scaffold00072.131
evm 27.model.AmTr v1.0 scaffold00214.2
evm 27.model.AmTr v1.0 scaffold00045.243
evm 27.model.AmTr v1.0 scaffold00025.396
evm 27.model.AmTr v1.0 scaffold00045.244
evm 27.model.AmTr v1.0 scaffold00002.671
evm 27.model.AmTr v1.0 scaffold00078.88
LOC Os01g52514.1
LOC Os01g52540.1
LOC Os03g11370.1
LOC Os04g27990.1
LOC Os06g42630.1
LOC Os04g27960.1
LOC Os04g28000.1
AT4G31615.1/REM2
AT4G31620.1/REM3
AT4G31630.1/REM4
AT4G31610.1/REM1
AT2G24650.1/REM14
AT5G32460.1
AT2G24700.1/REM10
AT4G31660.1/REM7
AT4G00260.1/REM15
AT1G26680.1/REM17
AT4G31640.1/REM5
AT4G31650.1/REM6
AT4G31680.1/REM8
AT4G31690.1/REM9
0.1
REM
Chlorophyte
Charophyte
Bryophyte
Lycophyte
Gymnosperm
Basal angiosperm
Monocot
Eudicot

Data S1J. Phylogenetic relationships of B3 TFs from *M. viride* and 10 evolutionarily representative species. The *M. viride* sequences were highlighted by triangles. Numbers on the major branches indicate bootstrap values (> 50%) in 1,000 replicates. The classification/clade was named based on previous studies.[10-12]

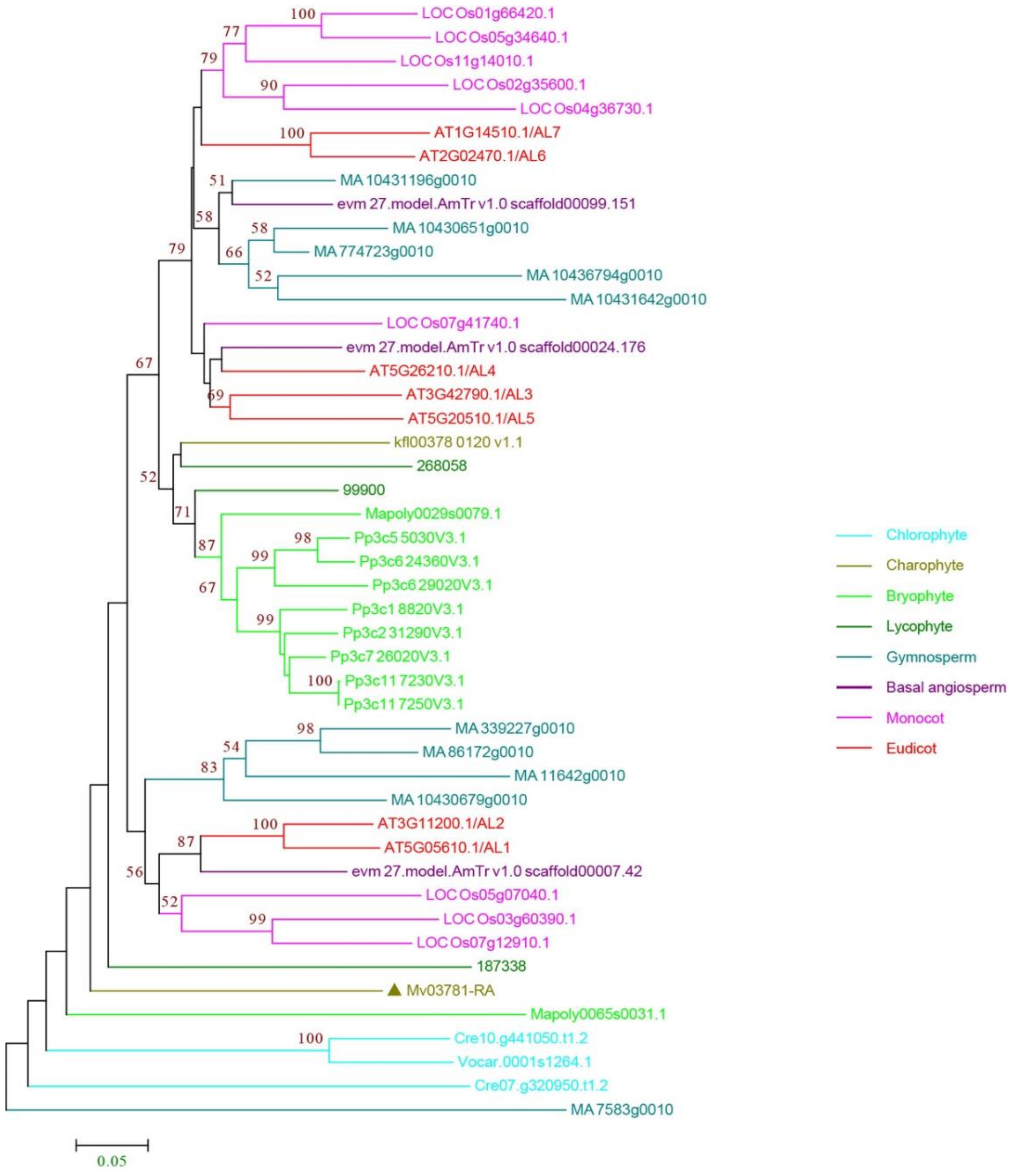

Data S1K. Phylogenetic relationships of alfin1-like TFs from *M. viride* and 10 evolutionarily representative species. The *M. viride* sequence was highlighted by a triangle. Numbers on the major branches indicate bootstrap values (> 50%) in 1,000 replicates.

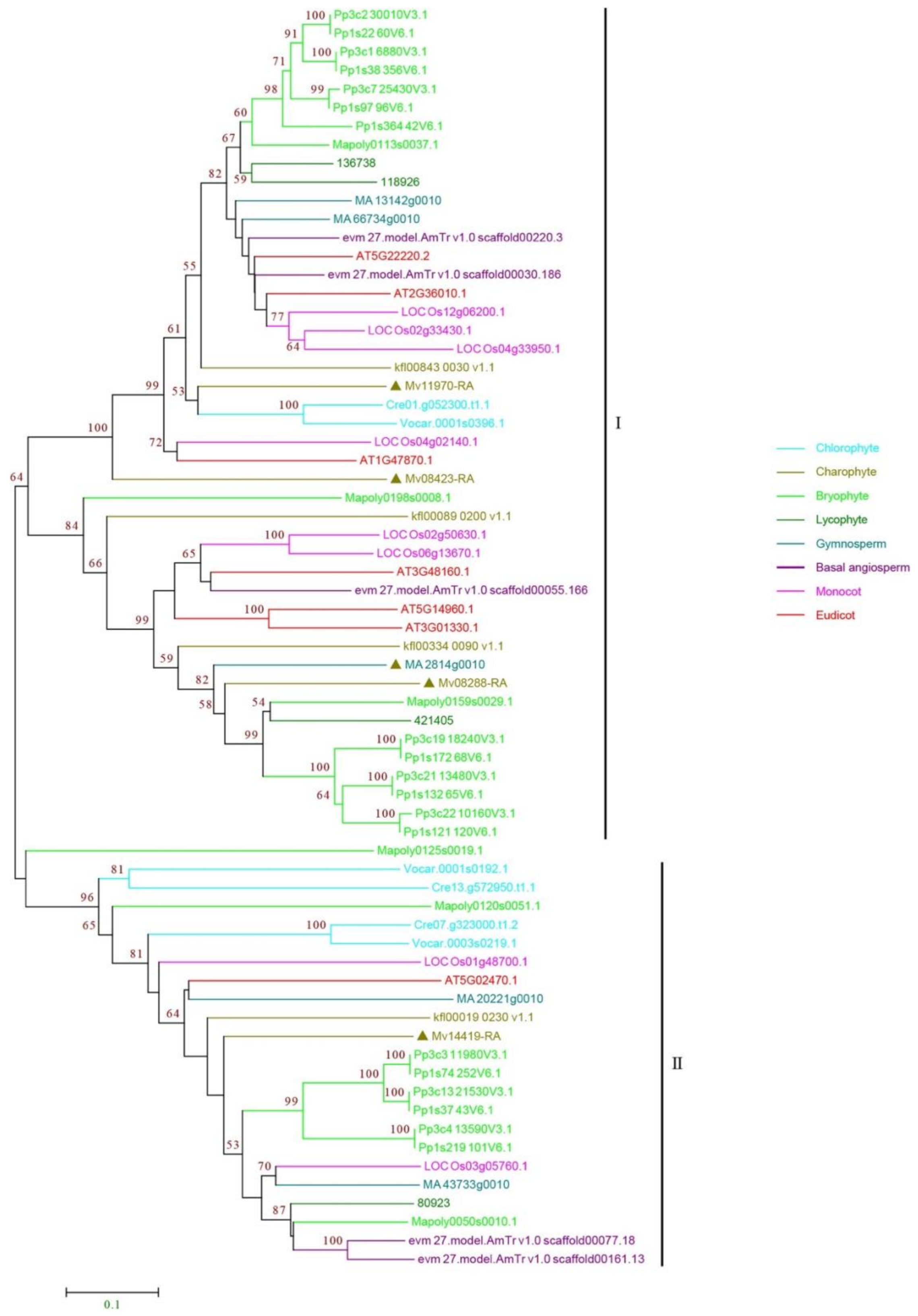

Data S1L. Phylogenetic relationships of E2 factor (E2F) TFs from *M. viride* and 10 evolutionarily representative species. The *M. viride* sequences were highlighted by triangles. Numbers on the major branches indicate bootstrap values (> 50%) in 1,000 replicates. The classification/clade was named based on previous studies.[13-14]

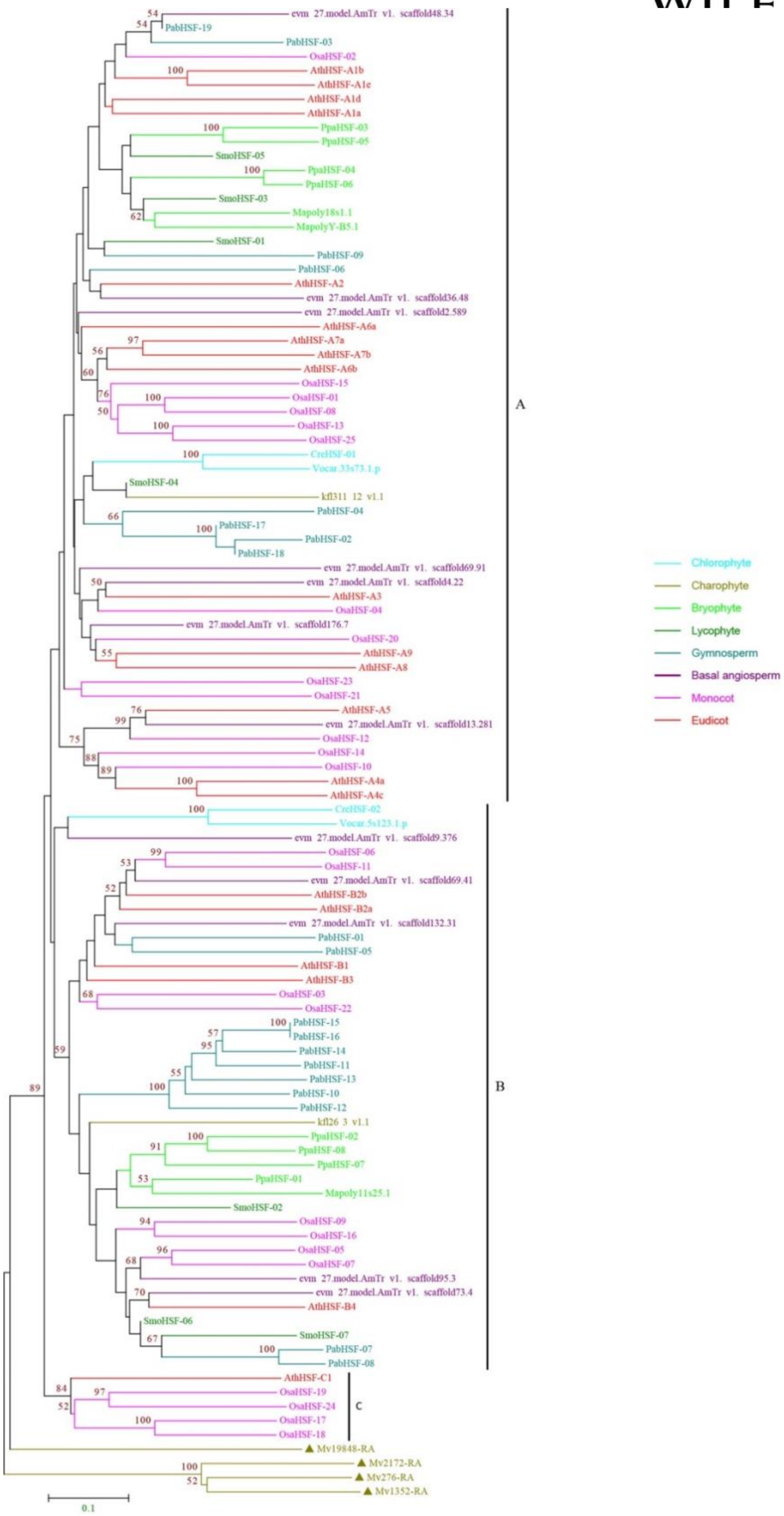

Data S1M. Phylogenetic relationships of heat shock transcription factor (HSF) TFs from *M. viride* and 10 evolutionarily representative species. The *M. viride* sequences were highlighted by triangles. Numbers on the major branches indicate bootstrap values (> 50%) in 1,000 replicates. The classification/clade was named based on a previous study.[15]

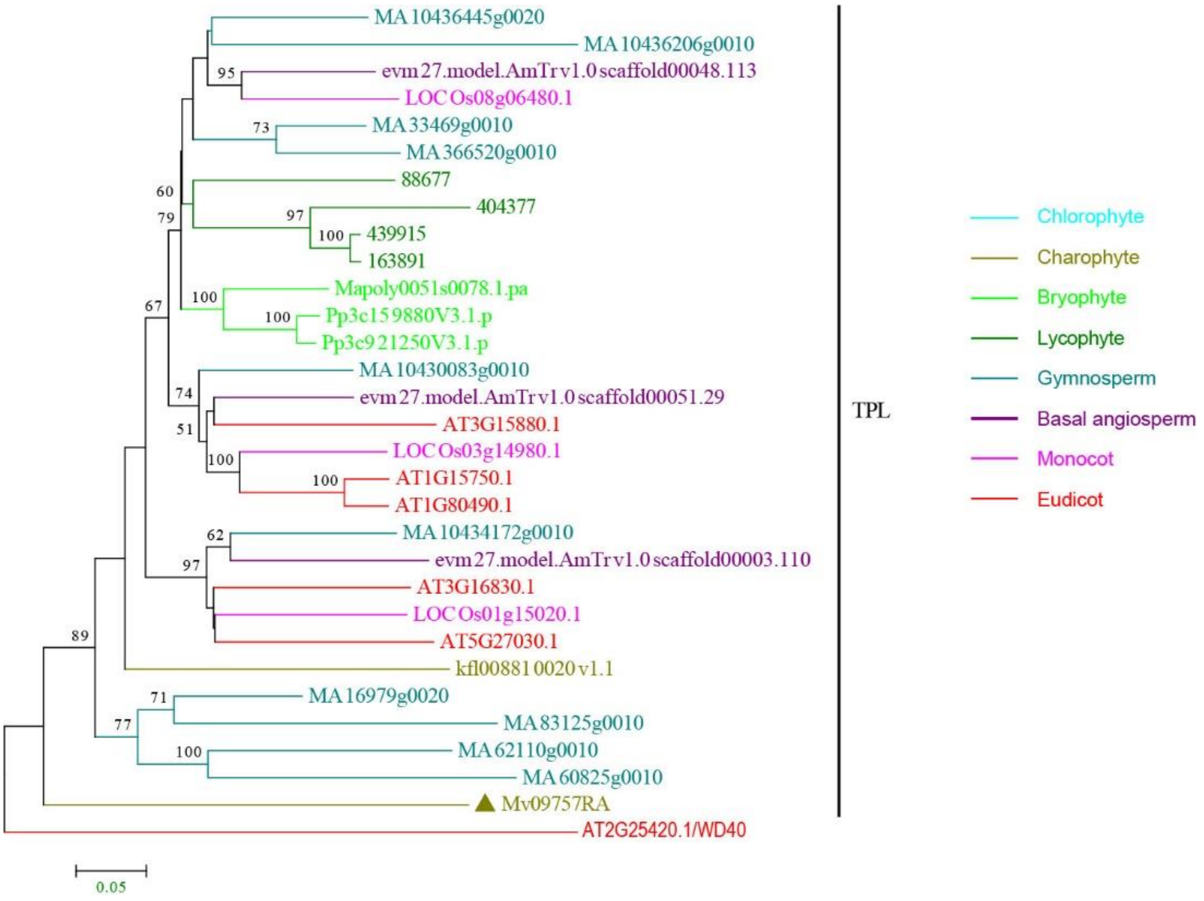

Data S1N. Phylogenetic relationships of TOPLESS (TPL) proteins from *M. viride* and other evolutionarily representative species. The *M. viride* sequence was highlighted by a triangle. Numbers on the major branches indicate bootstrap values (> 50%) in 1,000 replicates.

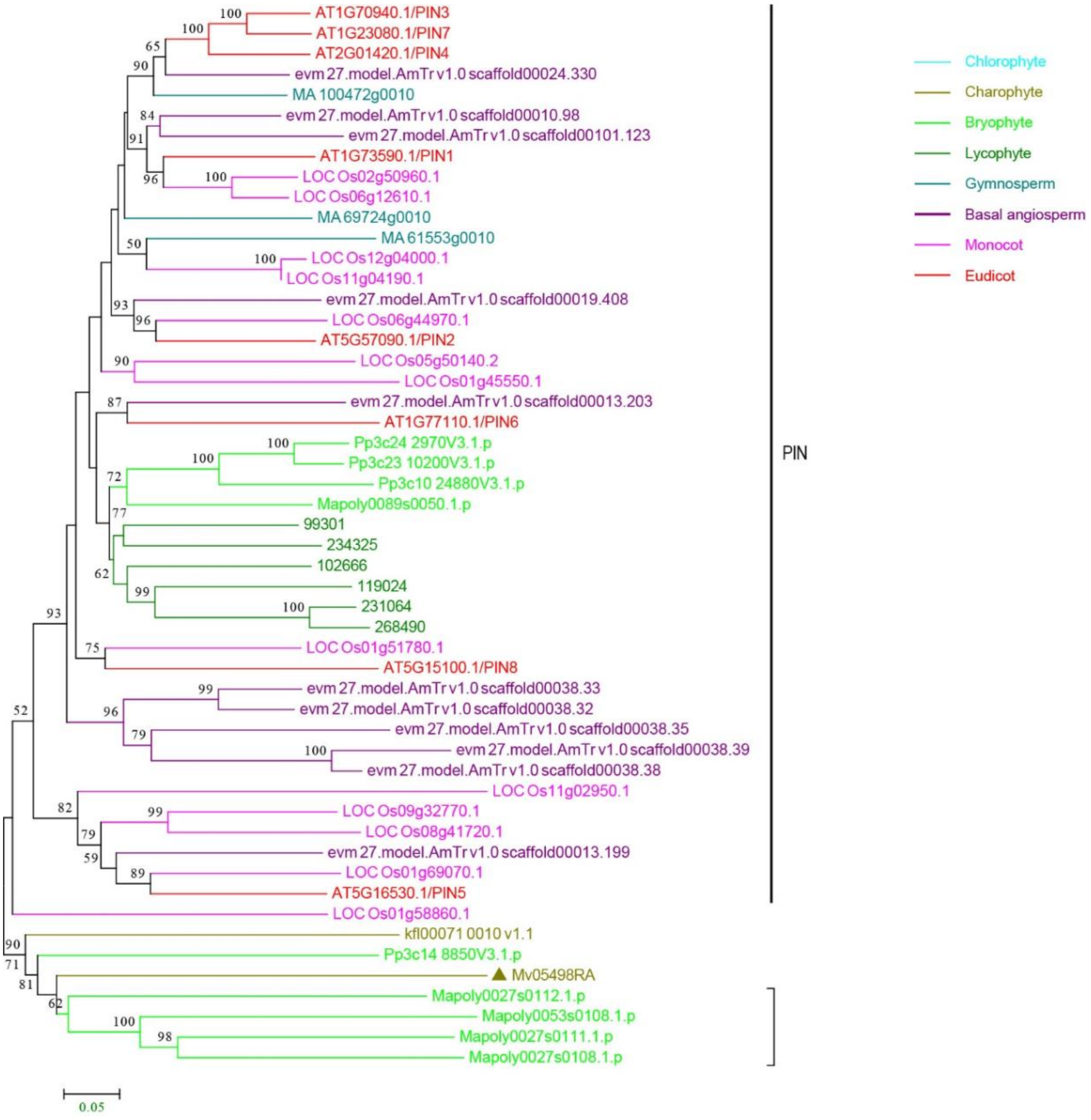

Data S1O. Phylogenetic relationships of PIN-FORMED (PIN) proteins from *M. viride* and other evolutionarily representative species. The *M. viride* sequence was highlighted by a triangle. Numbers on the major branches indicate bootstrap values (> 50%) in 1,000 replicates.

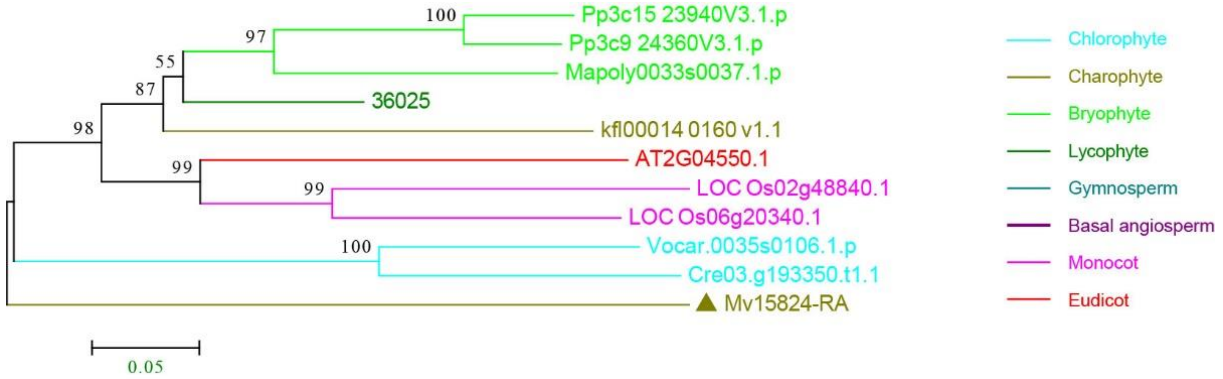

Data S1P. Phylogenetic relationships of INDOLE-3-BUTYRIC ACID RESPONSE (IBR) proteins from *M. viride* and other evolutionarily representative species. The *M. viride* sequence was highlighted by a triangle. Numbers on the major branches indicate bootstrap values (> 50%) in 1,000 replicates.

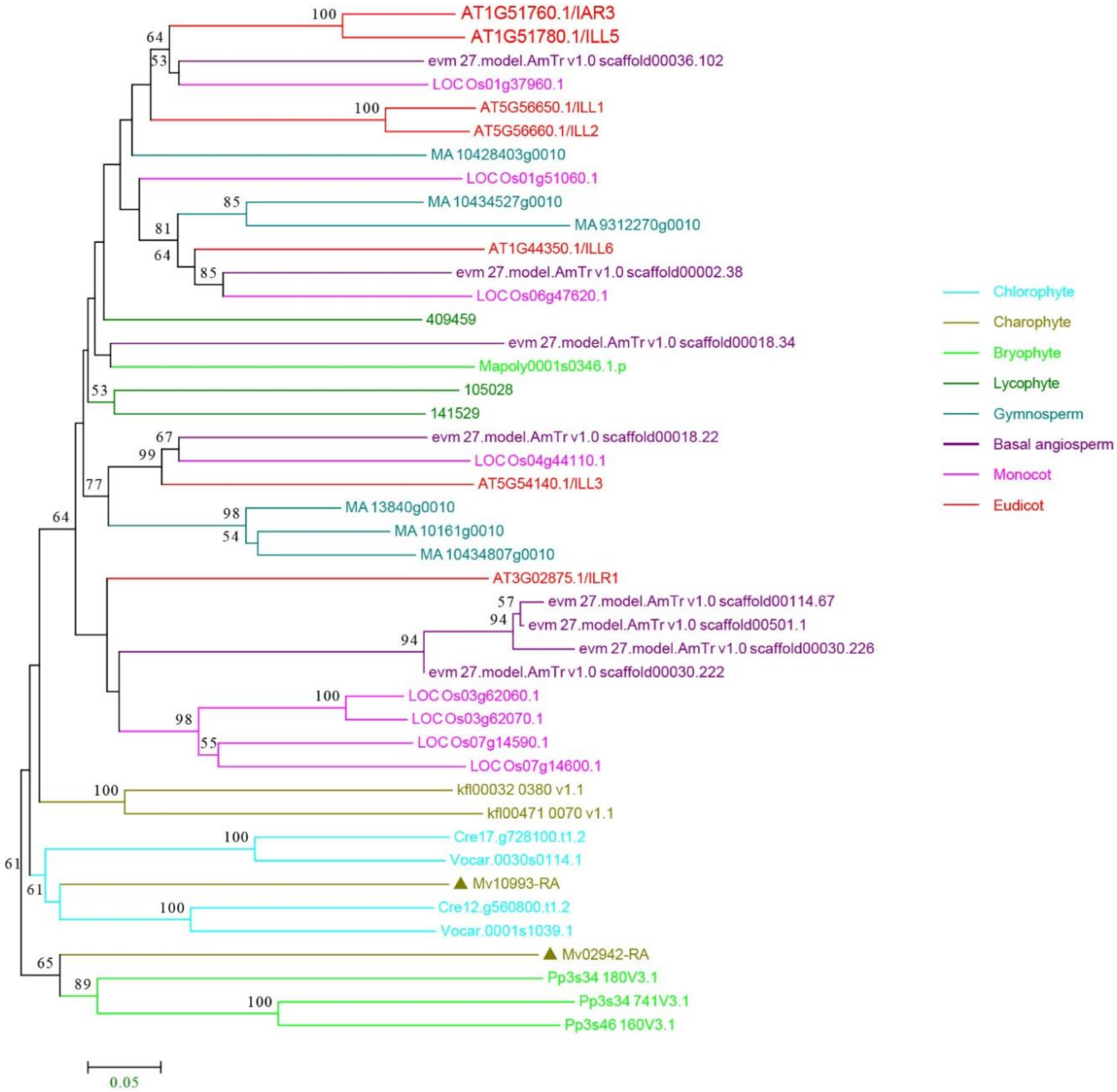

Data S1Q. Phylogenetic relationships of IAA-LEUCINE RESISTANT (ILR) proteins from *M. viride* and other evolutionarily representative species. The *M. viride* sequences were highlighted by triangles. Numbers on the major branches indicate bootstrap values (> 50%) in 1,000 replicates.

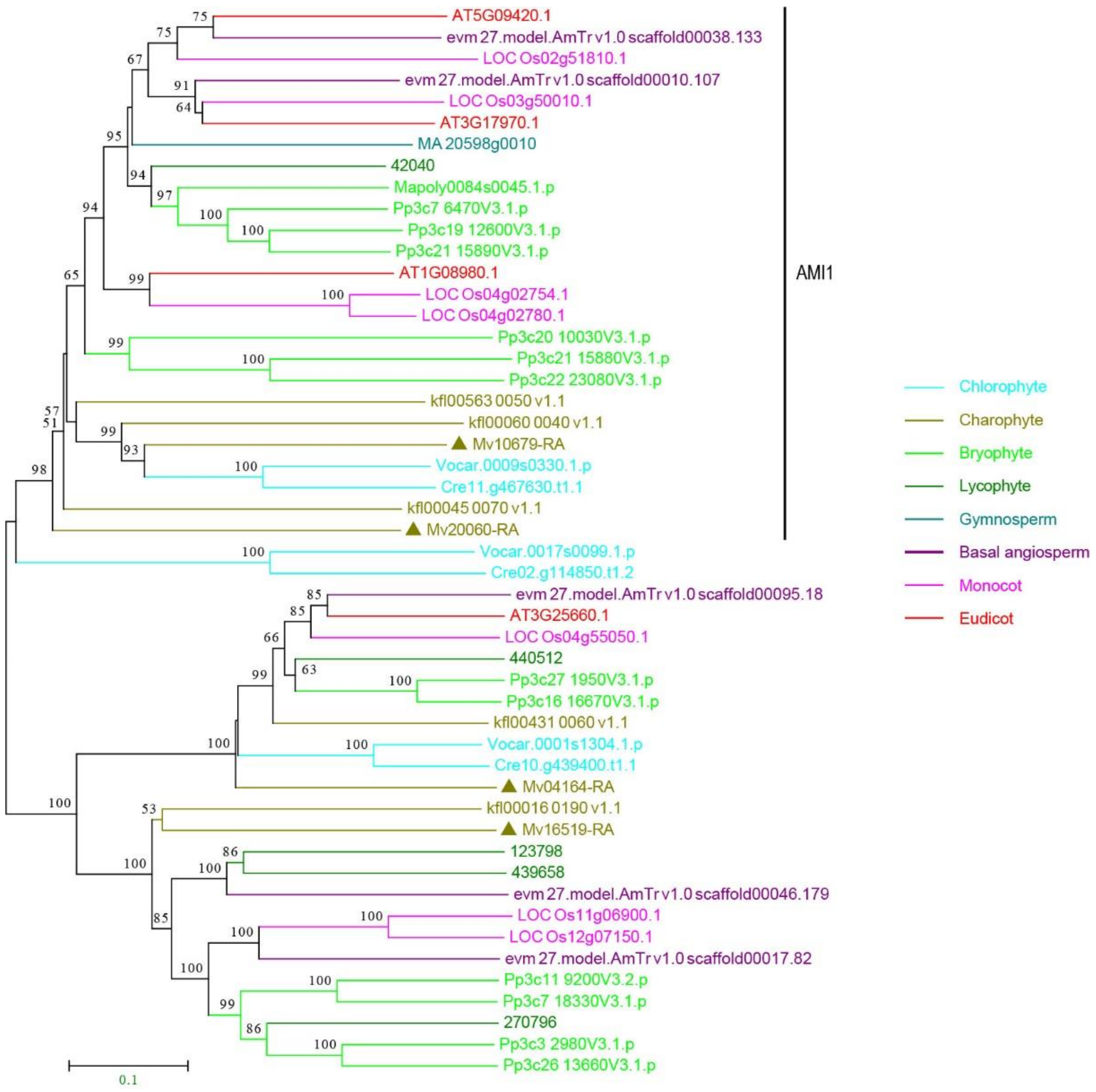

Data S1R. Phylogenetic relationships of AMIDASEA1 (AMI1) proteins from *M. viride* and other evolutionarily representative species. The *M. viride* sequences were highlighted by triangles. Numbers on the major branches indicate bootstrap values (> 50%) in 1,000 replicates.

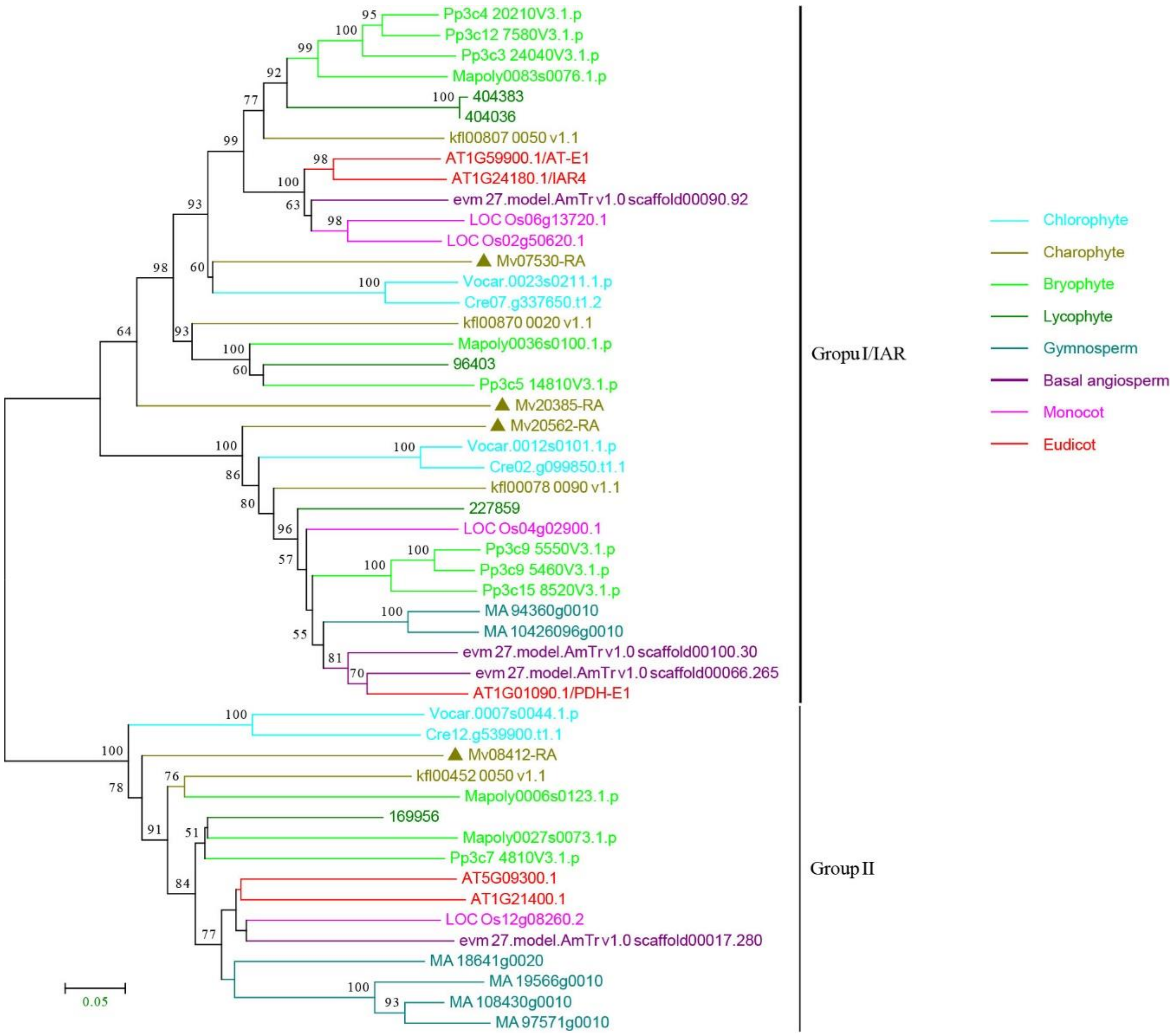

Data S1S. Phylogenetic relationships of IAA-CONJUGATE-RESISTANT (IAR) proteins from *M. viride* and other evolutionarily representative species. The *M. viride* sequences were highlighted by triangles. Numbers on the major branches indicate bootstrap values (> 50%) in 1,000 replicates.

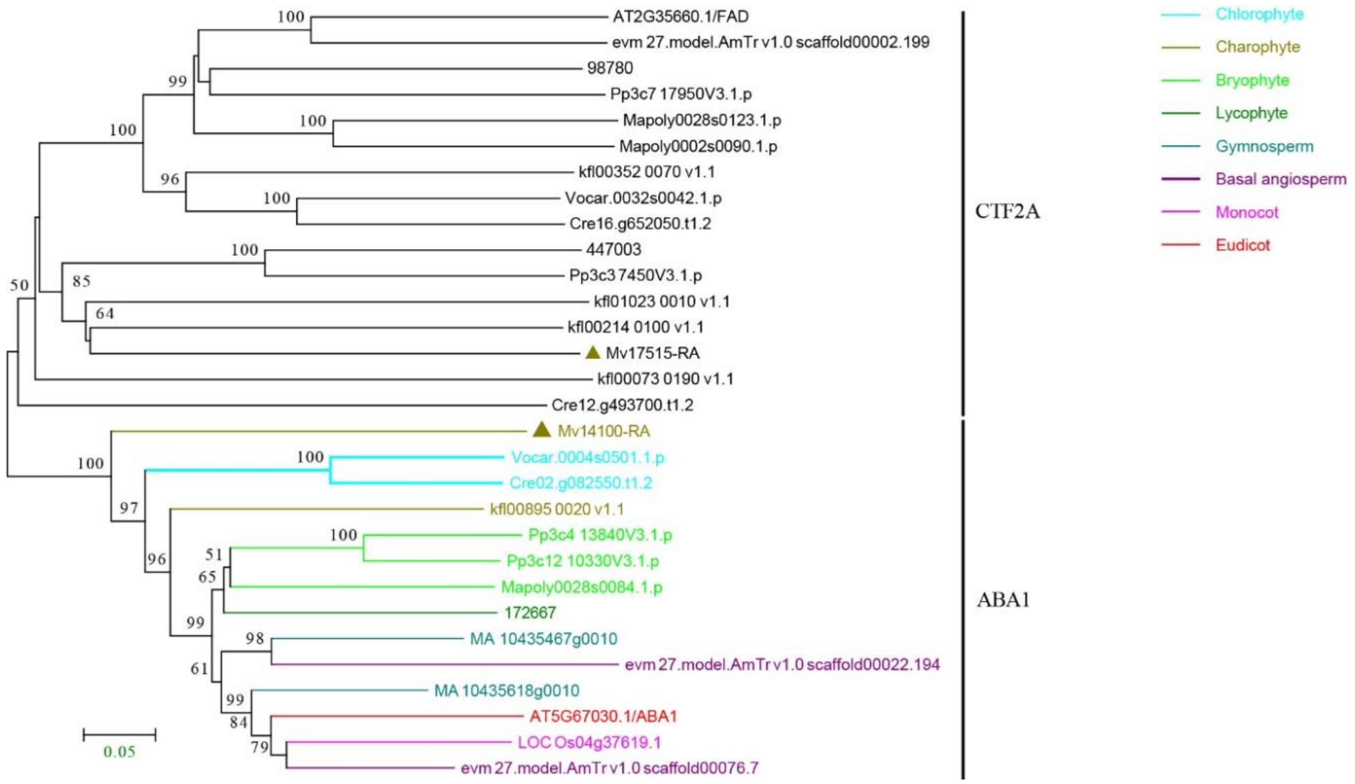

Data S1T. Phylogenetic relationships of ABA DEFICIENT 1 (ABA1) proteins from *M. viride* and other evolutionarily representative species. *M. viride* sequences were highlighted by triangles. Numbers on the major branches indicate bootstrap values (> 50%) in 1,000 replicates.

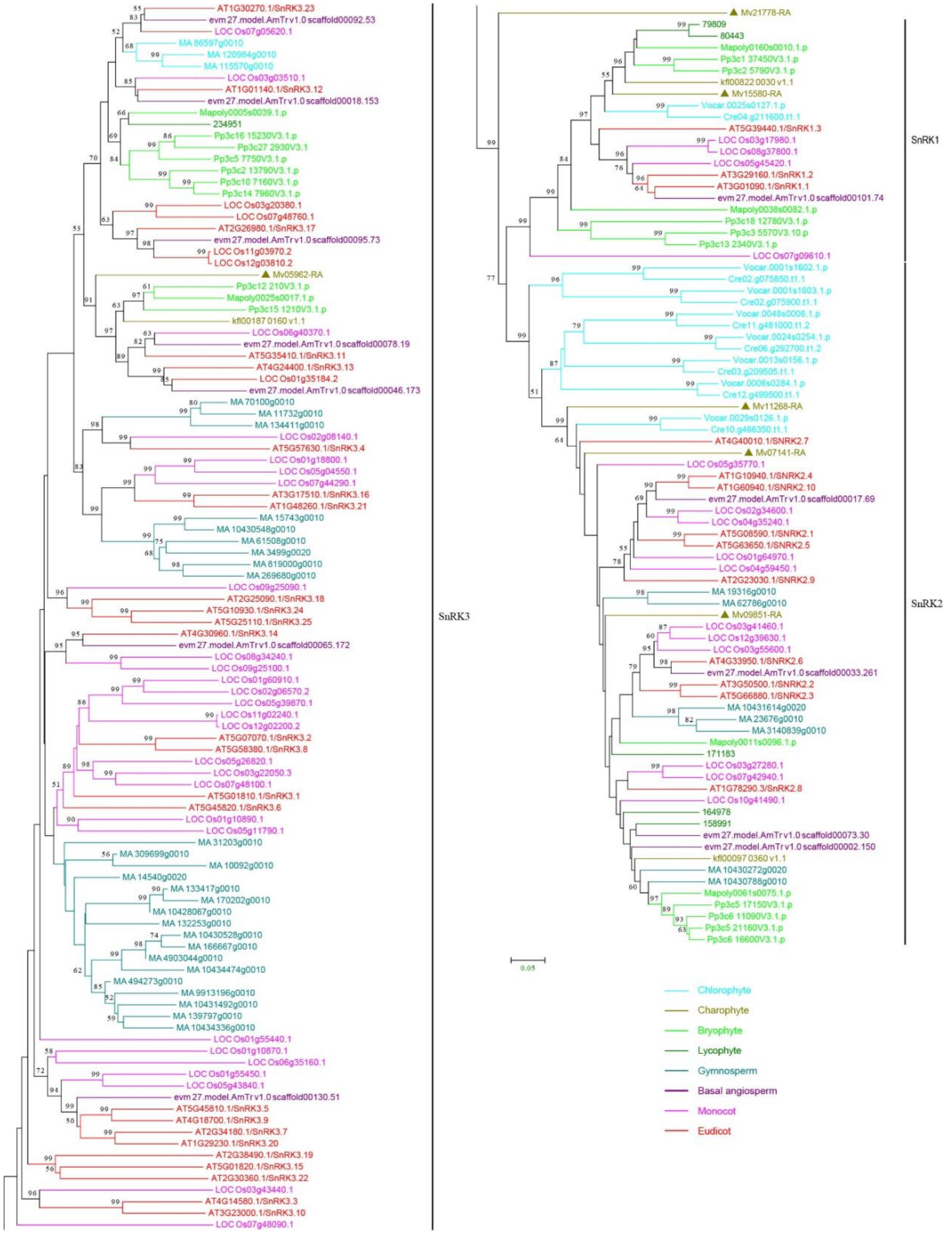

Data S1U. Phylogenetic relationships of SNF1-RELATED PROTEIN KINASE (SnRK) proteins from *M. viride* and other evolutionarily representative species. The *M. viride* sequences were highlighted by triangles. Numbers on the major branches indicate bootstrap values (> 50%) in 1,000 replicates.

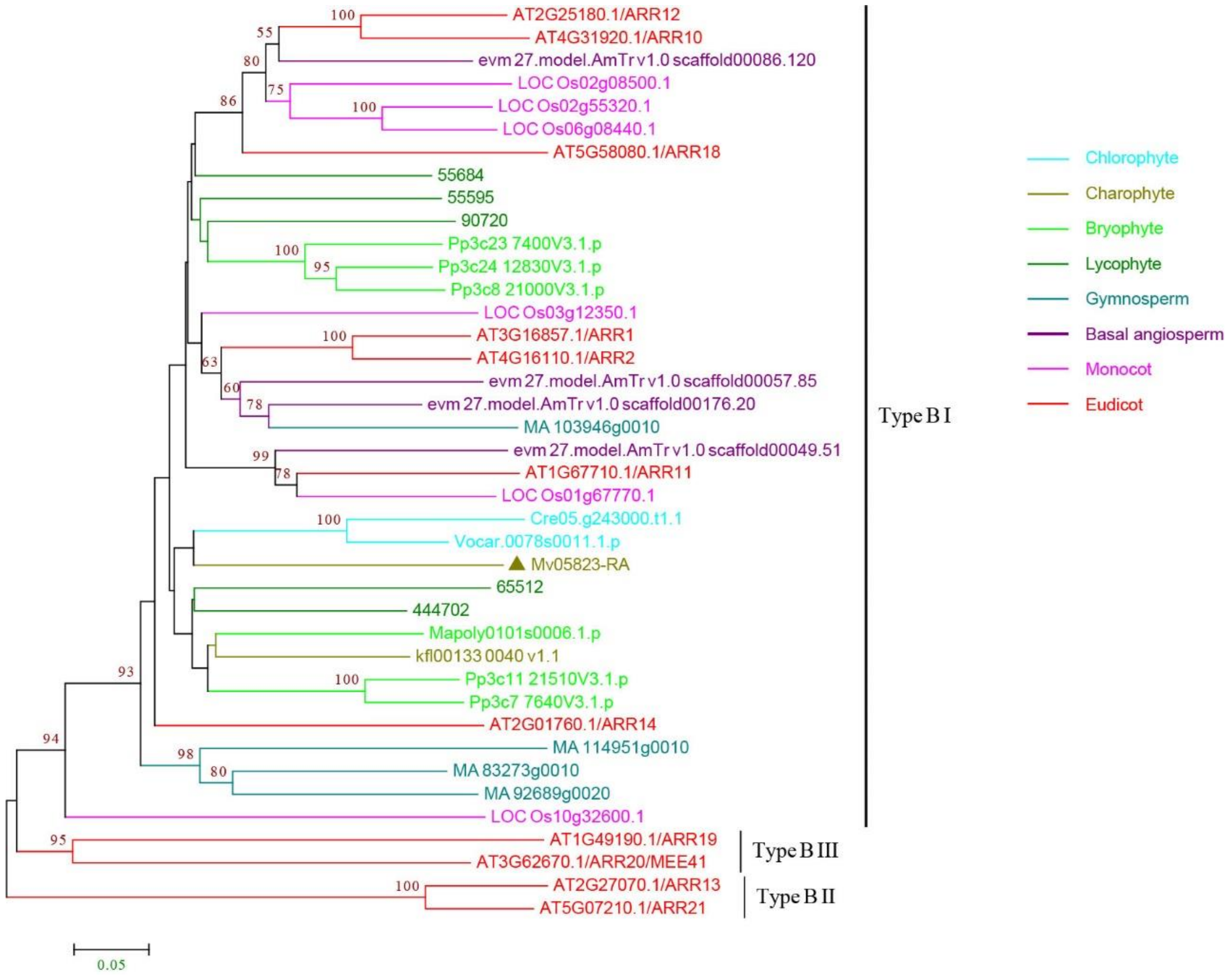

Data S1V. Phylogenetic relationships of Type B RESPONSE REGULATOR (ARR) proteins from *M. viride* and other evolutionarily representative species. The *M. viride* sequence was highlighted by a triangle. Numbers on the major branches indicate bootstrap values (> 50%) in 1,000 replicates.

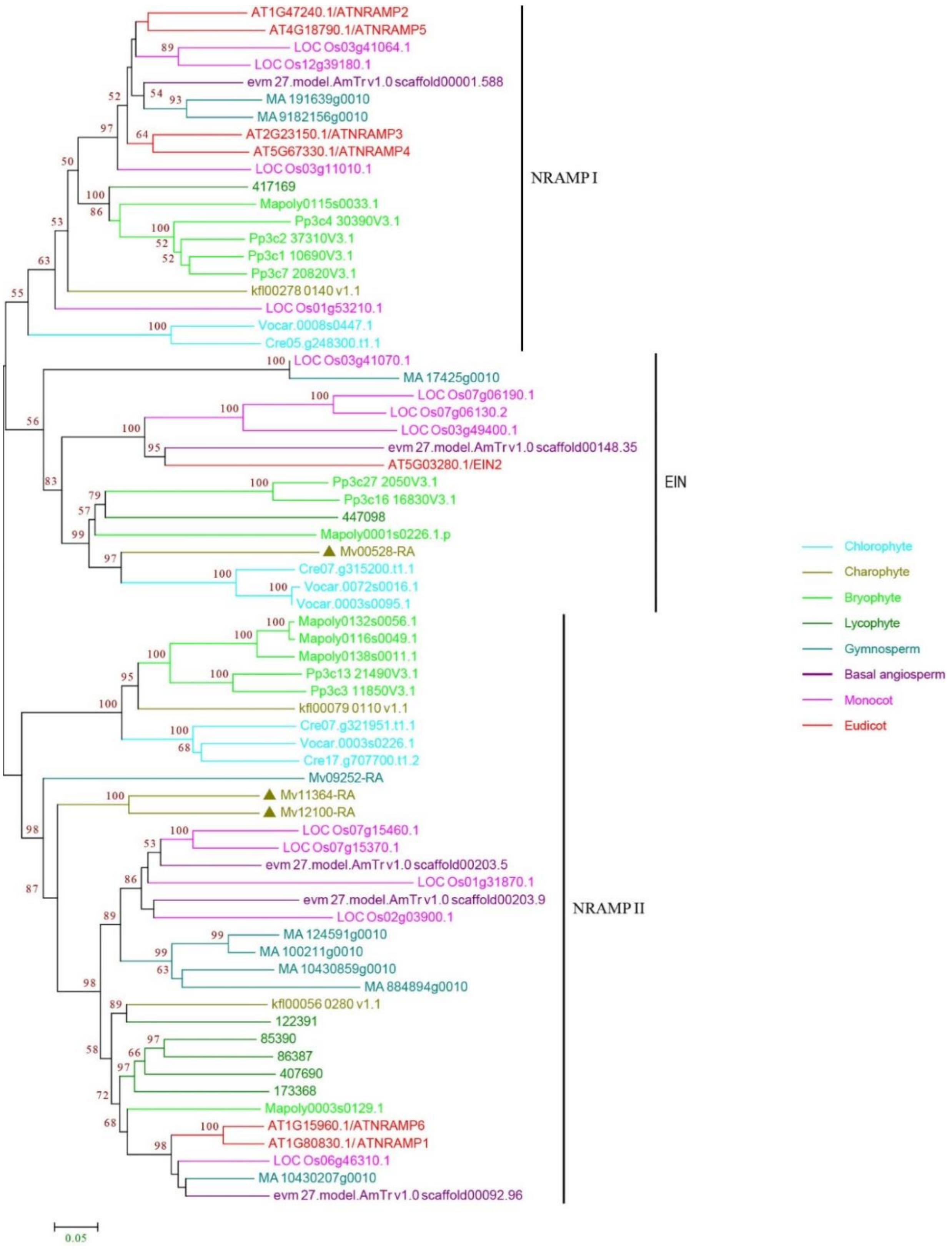

Data S1W. Phylogenetic relationships of ETHYLENE INSENSITIVE2 (EIN2) proteins from *M. viride* and other evolutionarily representative species. The *M. viride* sequences were highlighted by triangles. Numbers on the major branches indicate bootstrap values (> 50%) in 1,000 replicates.

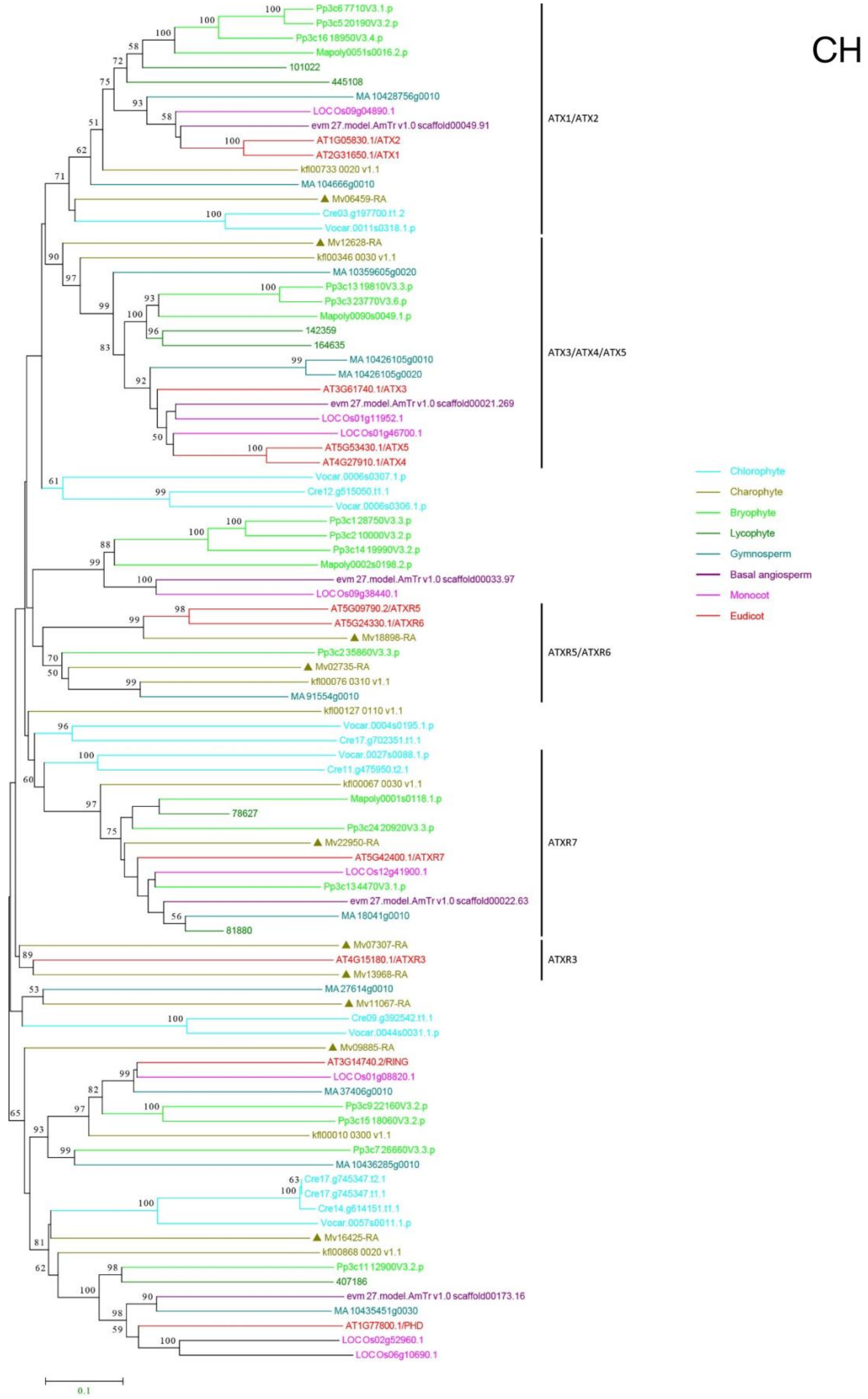

Data S1X. Phylogenetic relationships of TRITHORAX-LIKE PROTEIN (ATX) proteins from *M. viride* and other evolutionarily representative species. The *M. viride* sequences were highlighted by triangles. Numbers on the major branches indicate bootstrap values (> 50%) in 1,000 replicates.

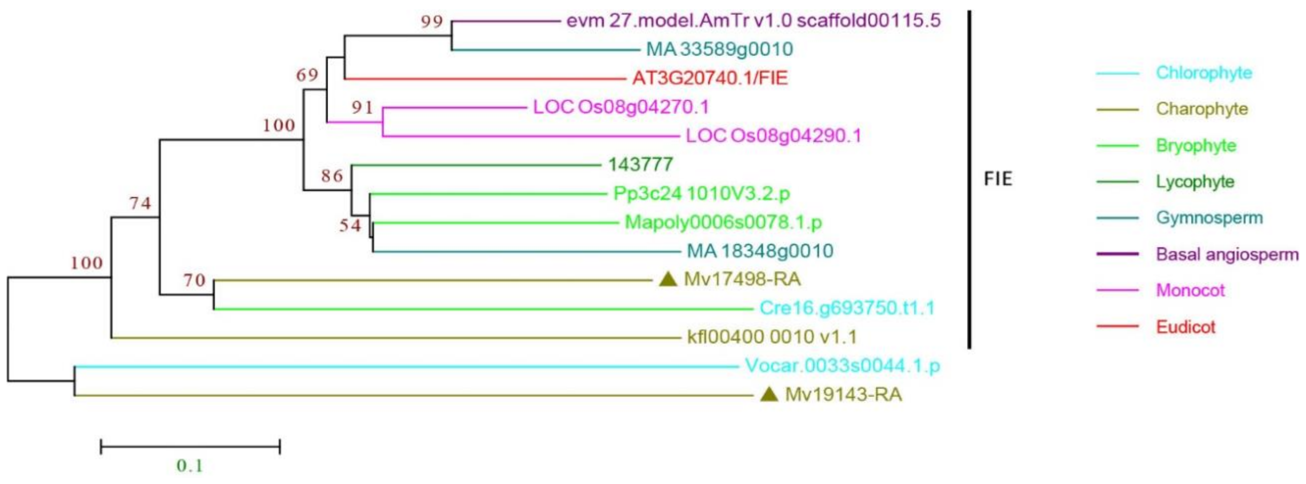

Data S1Y. Phylogenetic relationships of FERTILIZATION-INDEPENDENT ENDOSPERM (FIE) proteins from *M. viride* and other evolutionarily representative species. The *M. viride* sequences were highlighted by triangles. Numbers on the major branches indicate bootstrap values (> 50%) in 1,000 replicates.

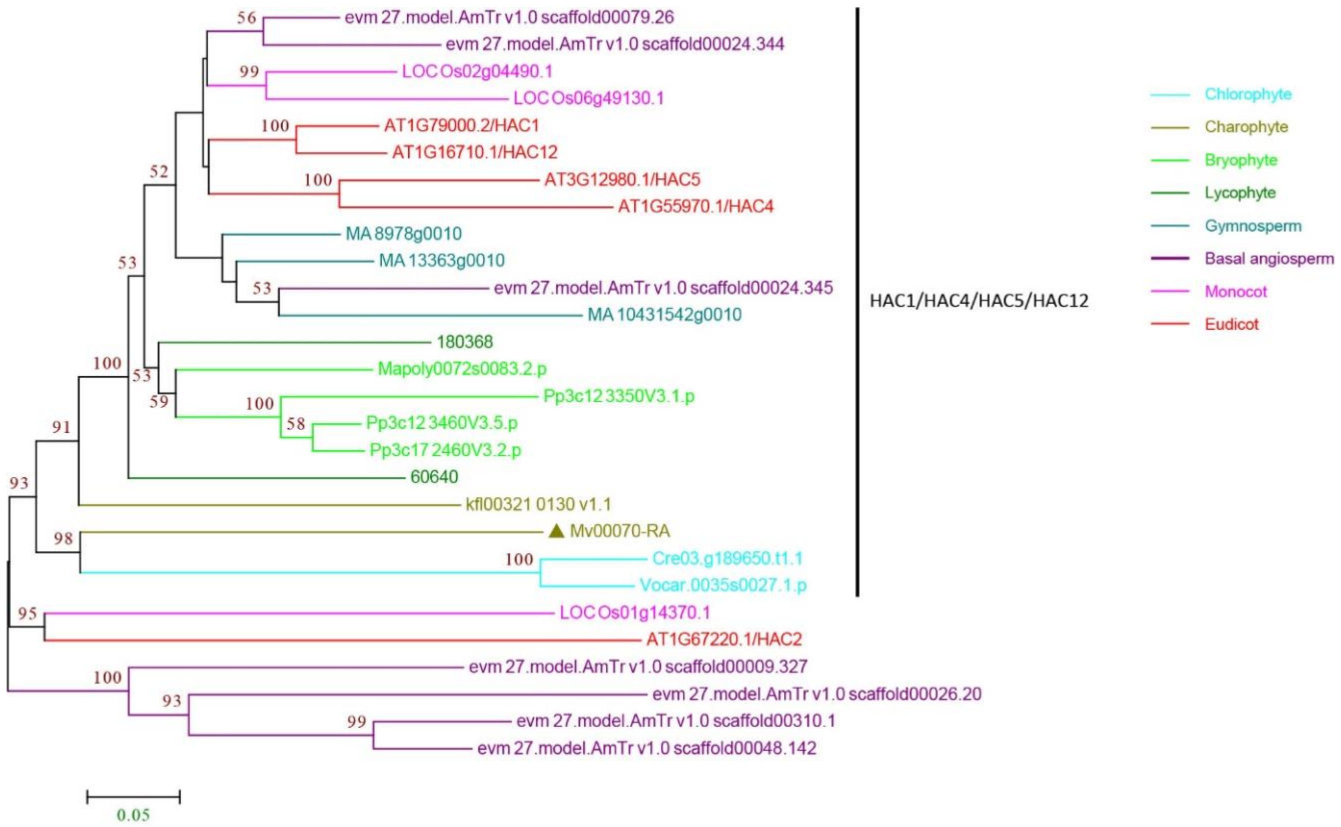

Data S1Z. Phylogenetic relationships of HISTONE ACETYLTRANSFERASE OF THE CBP FAMILY (HAC) proteins from *M. viride* and other evolutionarily representative species. The *M. viride* sequence was highlighted by a triangle. Numbers on the major branches indicate bootstrap values (> 50%) in 1,000 replicates.

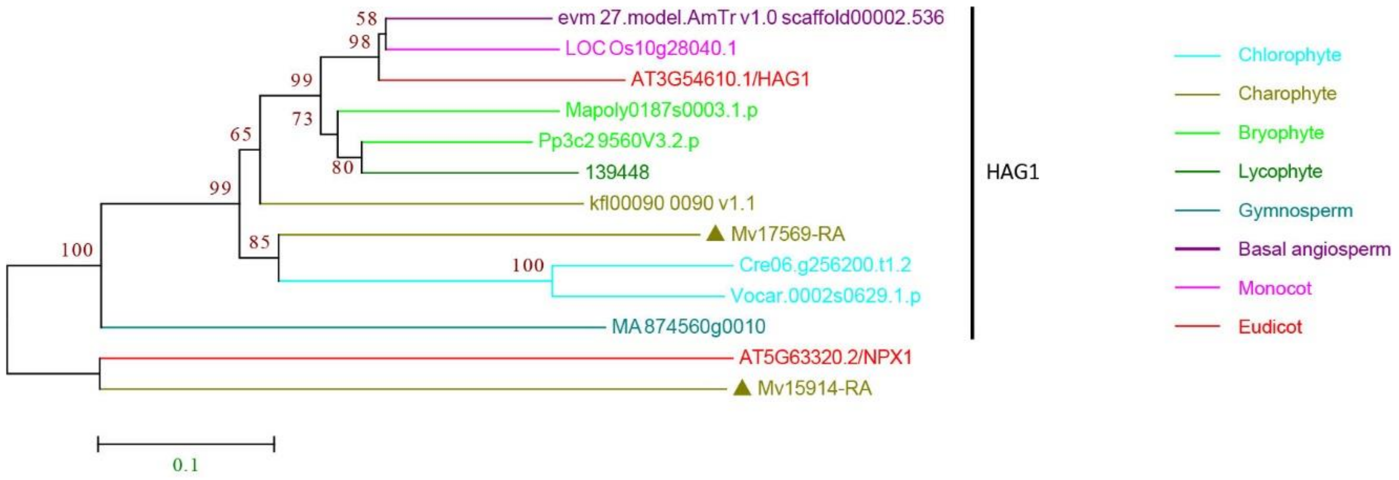

Data S1AA. Phylogenetic relationships of HISTONE ACETYLTRANSFERASE OF THE GNAT FAMILY 1 (HAG1) proteins from *M. viride* and other evolutionarily representative species. The *M. viride* sequences were highlighted by triangles. Numbers on the major branches indicate bootstrap values (> 50%) in 1,000 replicates.

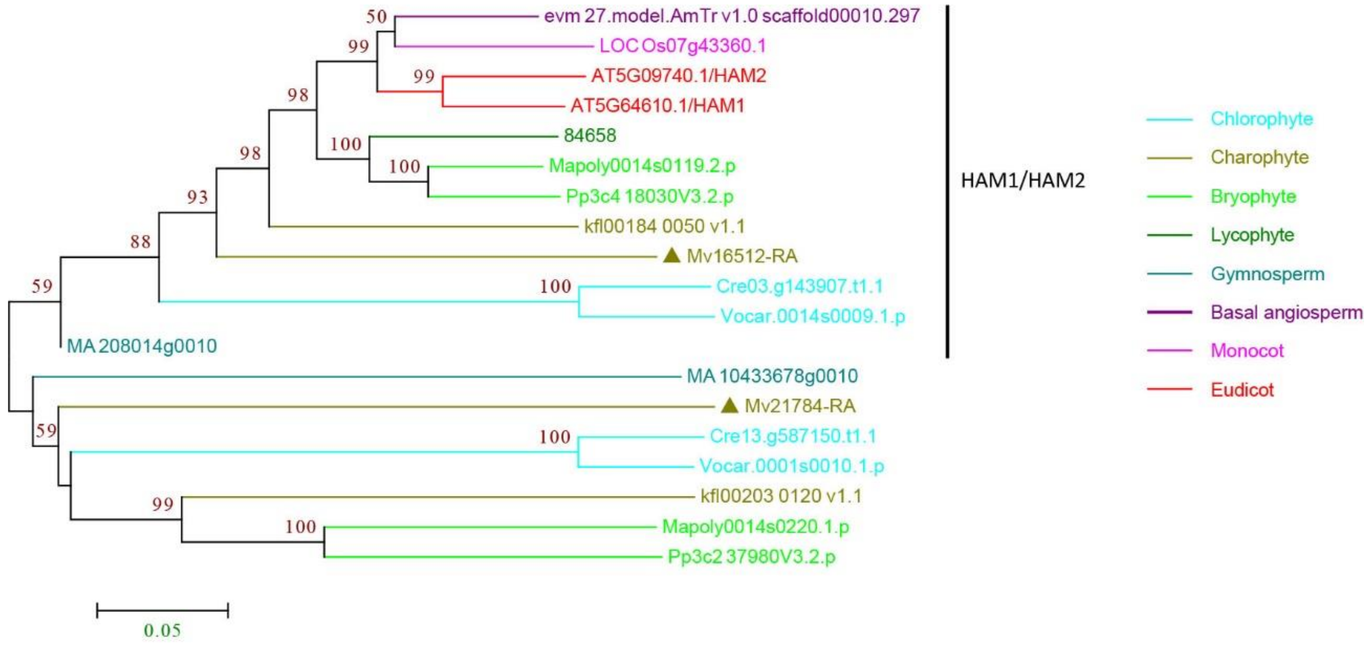

Data S1AB. Phylogenetic relationships of HISTONE ACETYLTRANSFERASE OF THE MYST FAMILY (HAM) proteins from *M. viride* and other evolutionarily representative species. The *M. viride* sequences were highlighted by triangles. Numbers on the major branches indicate bootstrap values (> 50%) in 1,000 replicates.

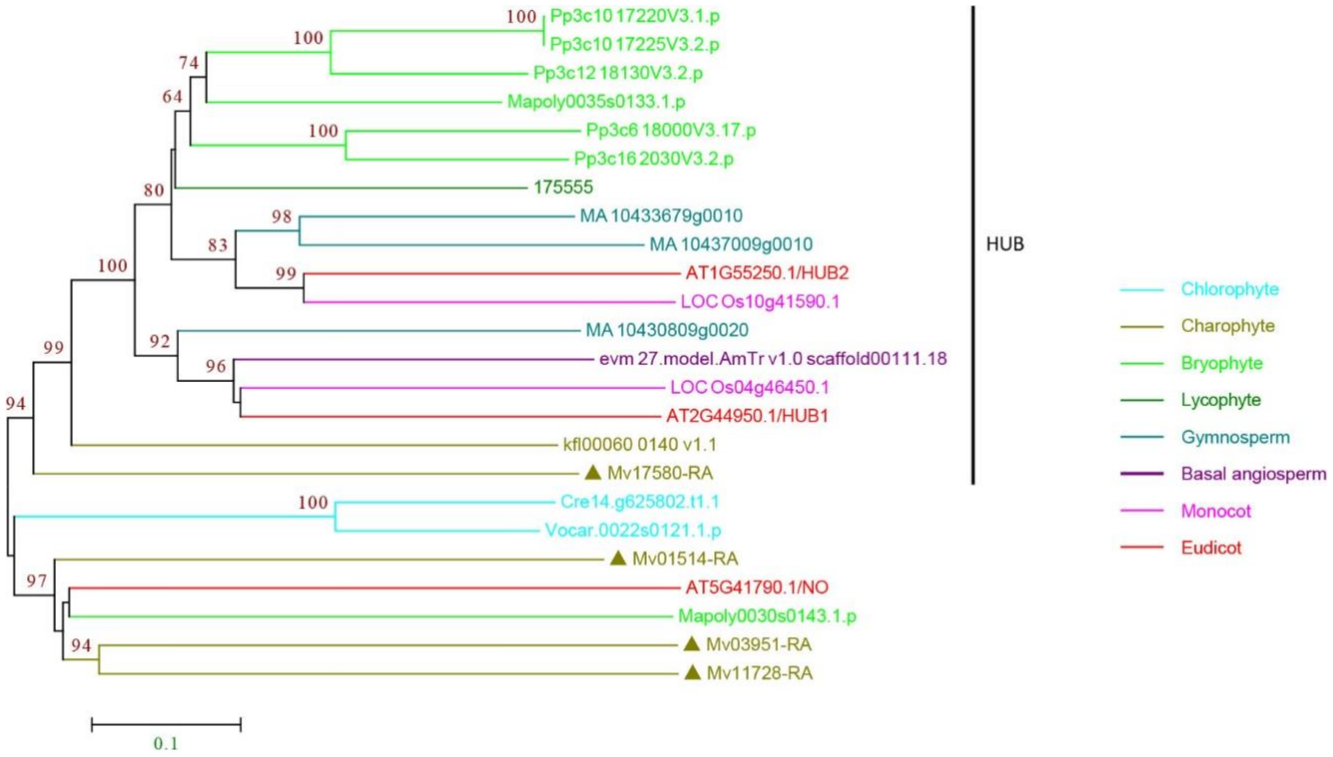

Data S1AC. Phylogenetic relationships of HISTONE MONO-UBIQUITINATION (HUB) proteins from *M. viride* and other evolutionarily representative species. The *M. viride* sequences were highlighted by triangles. Numbers on the major branches indicate bootstrap values (> 50%) in 1,000 replicates.

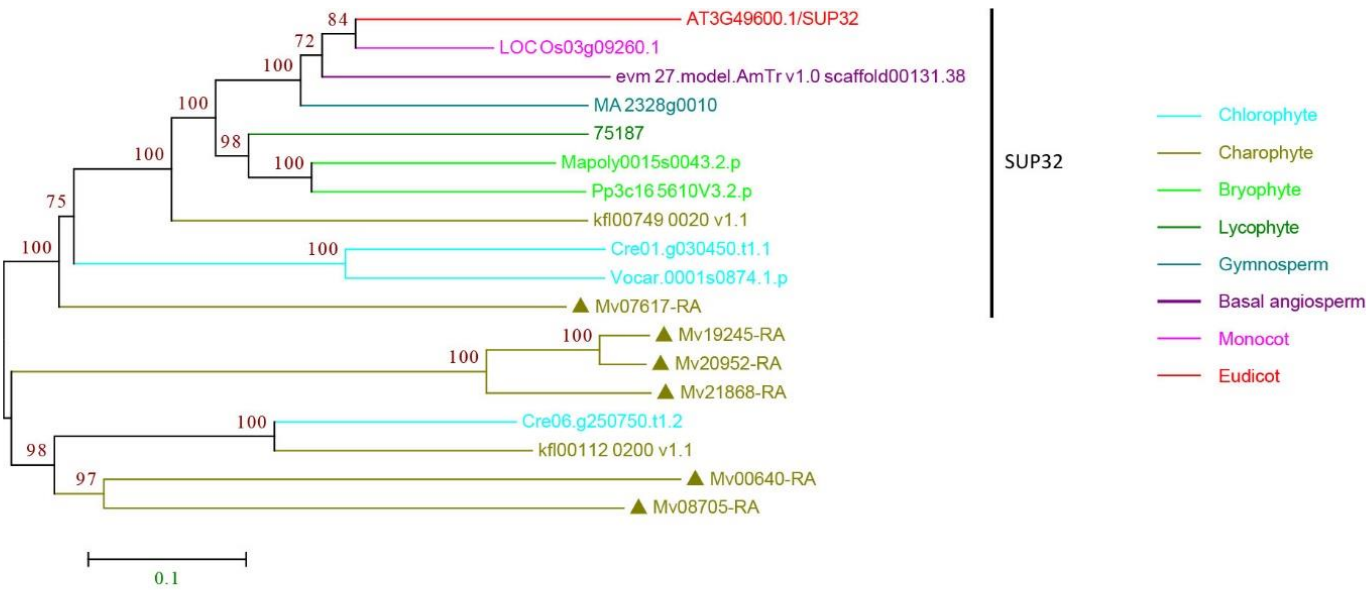

Data S1AD. Phylogenetic relationships of UBIQUITIN-SPECIFIC PROTEASE (also called SUP) proteins from *M. viride* and other evolutionarily representative species. The *M. viride* sequences were highlighted by triangles. Numbers on the major branches indicate bootstrap values (> 50%) in 1,000 replicates.

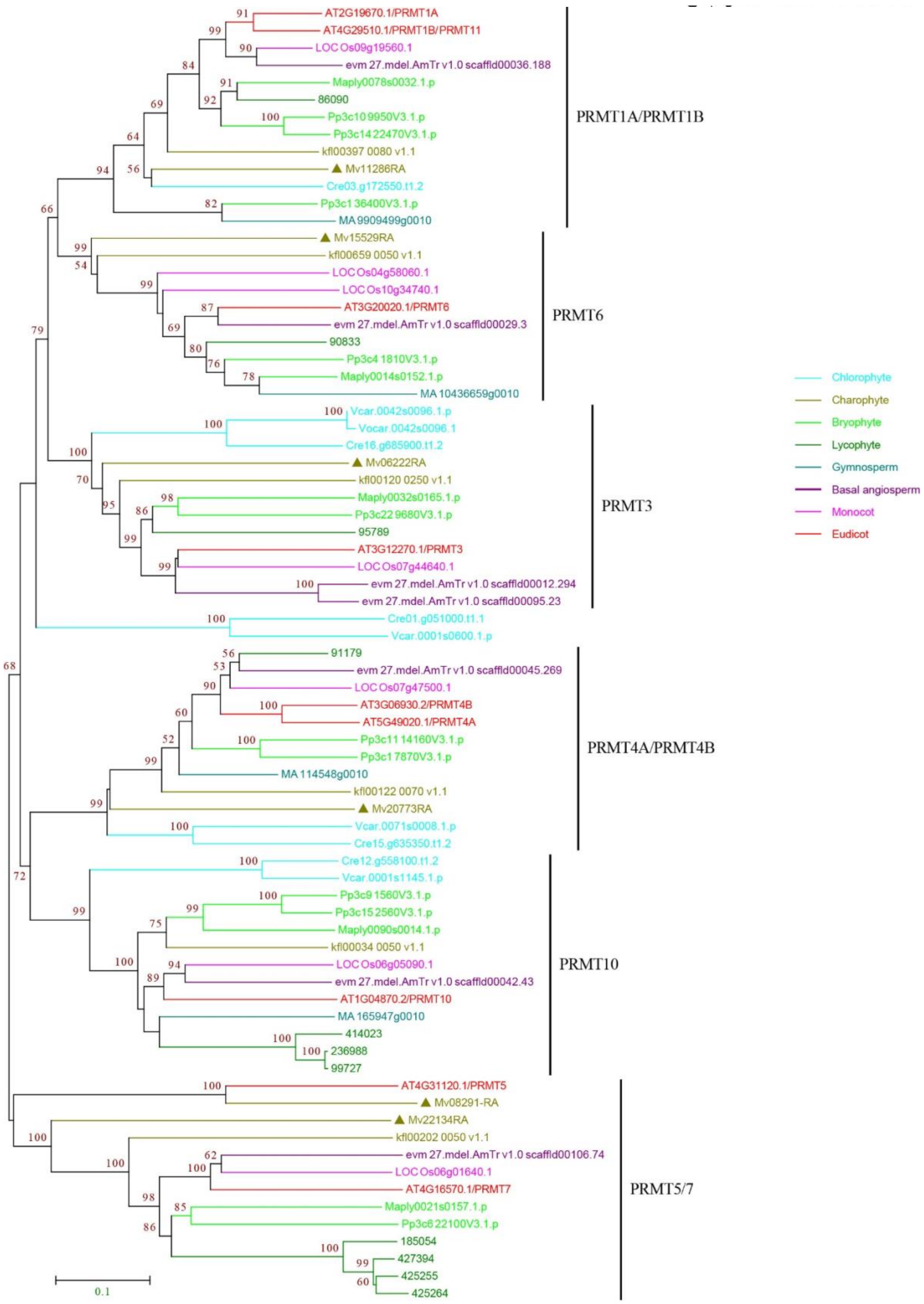

Data S1AE. Phylogenetic relationships of PROTEIN ARGININE METHYLTRANSFERASE (PRMT) proteins from *M. viride* and other evolutionarily representative species. The *M. viride* sequences were highlighted by triangles. Numbers on the major branches indicate bootstrap values (> 50%) in 1,000 replicates.

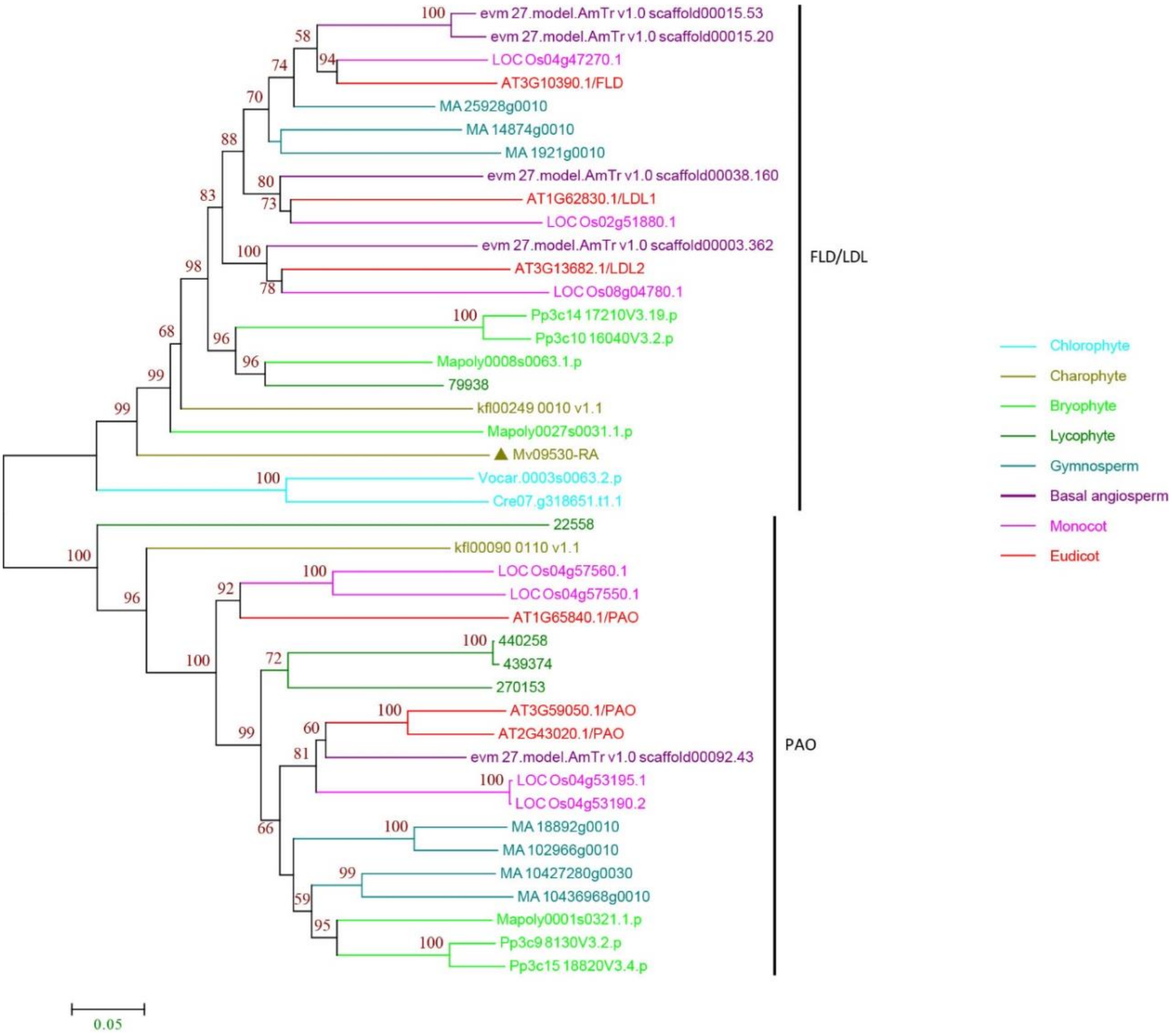

Data S1AF. Phylogenetic relationships of FLOWERING LOCUS D (FLD) proteins from *M. viride* and other evolutionarily representative species. The *M. viride* sequence was highlighted by a triangle. Numbers on the major branches indicate bootstrap values (> 50%) in 1,000 replicates.

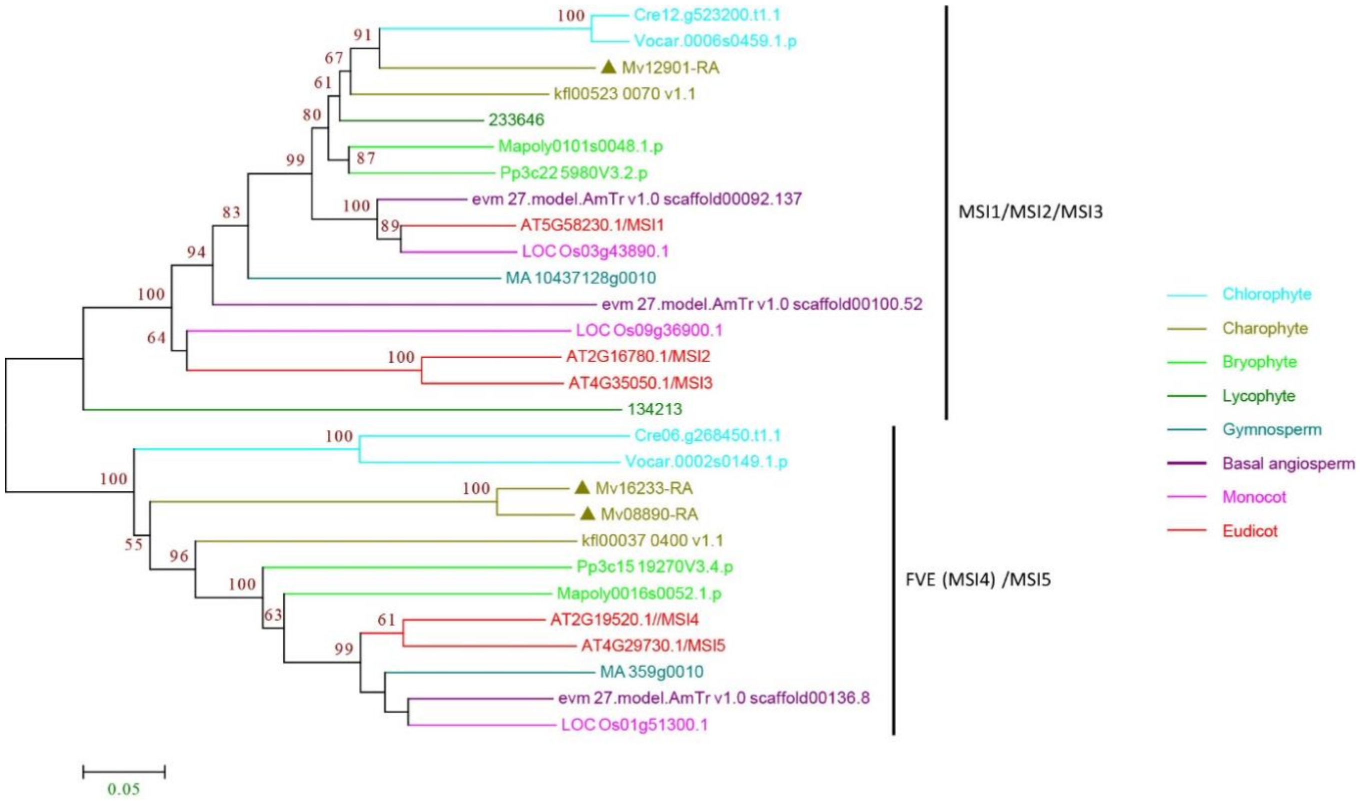

Data S1AG. Phylogenetic relationships of MULTICOPY SUPRESSOR OF IRA (MSI) proteins from *M. viride* and other evolutionarily representative species. The *M. viride* sequences were highlighted by triangles. Numbers on the major branches indicate bootstrap values (> 50%) in 1,000 replicates.

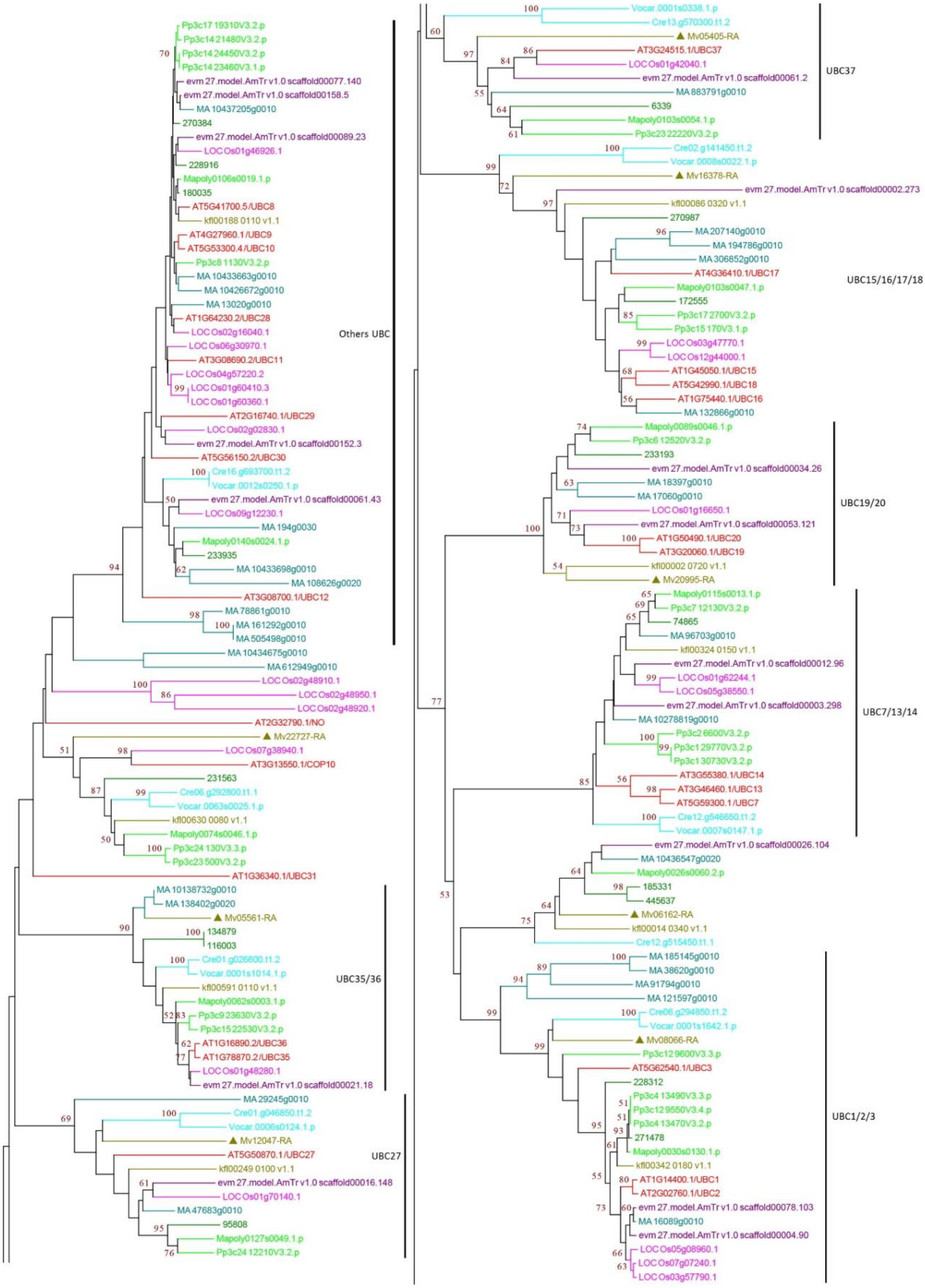
Pp3c17 19310V3.2.p
Pp3c14 21480V3.2.p
Pp3c14 24450V3.2.p
Pp3c14 23460V3.1.p
evm 27.model.AmTr v1.0 scaffold00077.140
evm 27.model.AmTr v1.0 scaffold00158.5
MA 10437205g0010
270384
evm 27.model.AmTr v1.0 scaffold00089.23
LOC Os01g46926.1
228916
Mapoly0106s0019.1.p
180035
AT5G41700.5/UBC8
kfl00188 0110 v1.1
AT4G27960.1/UBC9
AT5G53300.4/UBC10
Pp3c8 1130V3.2.p
MA 10433663g0010
MA 10426672g0010
MA 13020g0010
AT1G64230.2/UBC28
LOC Os02g16040.1
LOC Os06g30970.1
AT3G08690.2/UBC11
LOC Os04g57220.2
LOC Os01g60410.3
LOC Os01g60360.1
AT2G16740.1/UBC29
LOC Os02g02830.1
evm 27.model.AmTr v1.0 scaffold00152.3
AT5G56150.2/UBC30
Cre16.g693700.t1.2
Vocar.0012s0250.1.p
evm 27.model.AmTr v1.0 scaffold00061.43
LOC Os09g12230.1
MA 194g0030
Mapoly0140s0024.1.p
233935
MA 10433698g0010
MA 108626g0020
AT3G08700.1/UBC12
MA 78861g0010
MA 161292g0010
MA 505498g0010
MA 10434675g0010
MA 612949g0010
LOC Os02g48910.1
LOC Os02g48950.1
LOC Os02g48920.1
AT2G32790.1/NO
Mv22727-RA
LOC Os07g38940.1
AT3G13550.1/COP10
231563
Cre06.g292800.t1.1
Vocar.0063s0025.1.p
kfl00630 0080 v1.1
Mapoly0074s0046.1.p
Pp3c24 130V3.3.p
Pp3c23 500V3.2.p
AT1G36340.1/UBC31
Others UBC
MA 10138732g0010
MA 138402g0020
Mv05561-RA
134879
116003
Cre01.g026600.t1.2
Vocar.0001s1014.1.p
kfl00591 0110 v1.1
Mapoly0062s0003.1.p
Pp3c9 23630V3.2.p
Pp3c15 22530V3.2.p
AT1G16890.2/UBC36
AT1G78870.2/UBC35
LOC Os01g48280.1
evm 27.model.AmTr v1.0 scaffold00021.18
UBC35/36
MA 29245g0010
Cre01.g046850.t1.2
Vocar.0006s0124.1.p
Mv12047-RA
AT5G50870.1/UBC27
kfl00249 0100 v1.1
evm 27.model.AmTr v1.0 scaffold00016.148
LOC Os01g70140.1
MA 47683g0010
95808
Mapoly0127s0049.1.p
Pp3c24 12210V3.2.p
UBC27
Vocar.0001s0338.1.p
Cre13.g570300.t1.2
Mv05405-RA
AT3G24515.1/UBC37
LOC Os01g42040.1
evm 27.model.AmTr v1.0 scaffold00061.2
MA 883791g0010
6339
Mapoly0103s0054.1.p
Pp3c23 22220V3.2.p
UBC37
Cre02.g141450.t1.2
Vocar.0008s0022.1.p
Mv16378-RA
evm 27.model.AmTr v1.0 scaffold00002.273
kfl00086 0320 v1.1
270987
MA 207140g0010
MA 194786g0010
MA 306852g0010
AT4G36410.1/UBC17
Mapoly0103s0047.1.p
172555
Pp3c17 2700V3.2.p
Pp3c15 170V3.1.p
LOC Os03g47770.1
LOC Os12g44000.1
AT1G45050.1/UBC15
AT5G42990.1/UBC18
AT1G75440.1/UBC16
MA 132866g0010
UBC15/16/17/18
Mapoly0089s0046.1.p
Pp3c6 12520V3.2.p
233193
evm 27.model.AmTr v1.0 scaffold00034.26
MA 18397g0010
MA 17060g0010
LOC Os01g16650.1
evm 27.model.AmTr v1.0 scaffold00053.121
AT1G50490.1/UBC20
AT3G20060.1/UBC19
kfl00002 0720 v1.1
Mv20995-RA
UBC19/20
Mapoly0115s0013.1.p
Pp3c7 12130V3.2.p
74865
MA 96703g0010
kfl00324 0150 v1.1
evm 27.model.AmTr v1.0 scaffold00012.96
LOC Os01g62244.1
LOC Os05g38550.1
evm 27.model.AmTr v1.0 scaffold00003.298
MA 10278819g0010
Pp3c2 6600V3.2.p
Pp3c1 29770V3.2.p
Pp3c1 30730V3.2.p
AT3G55380.1/UBC14
AT3G46460.1/UBC13
AT5G59300.1/UBC7
Cre12.g546650.t1.2
Vocar.0007s0147.1.p
UBC7/13/14
evm 27.model.AmTr v1.0 scaffold00026.104
MA 10436547g0020
Mapoly0026s0060.2.p
185331
445637
Mv06162-RA
kfl00014 0340 v1.1
Cre12.g515450.t1.1
MA 185145g0010
MA 38620g0010
MA 91794g0010
MA 121597g0010
Cre06.g294850.t1.2
Vocar.0001s1642.1.p
Mv08066-RA
Pp3c12 9600V3.3.p
AT5G62540.1/UBC3
228312
Pp3c4 13490V3.3.p
Pp3c12 9550V3.4.p
Pp3c4 13470V3.2.p
271478
Mapoly0030s0130.1.p
kfl00342 0180 v1.1
AT1G14400.1/UBC1
AT2G02760.1/UBC2
evm 27.model.AmTr v1.0 scaffold00078.103
MA 16089g0010
evm 27.model.AmTr v1.0 scaffold00004.90
LOC Os05g08960.1
LOC Os07g07240.1
LOC Os03g57790.1
UBC1/2/3

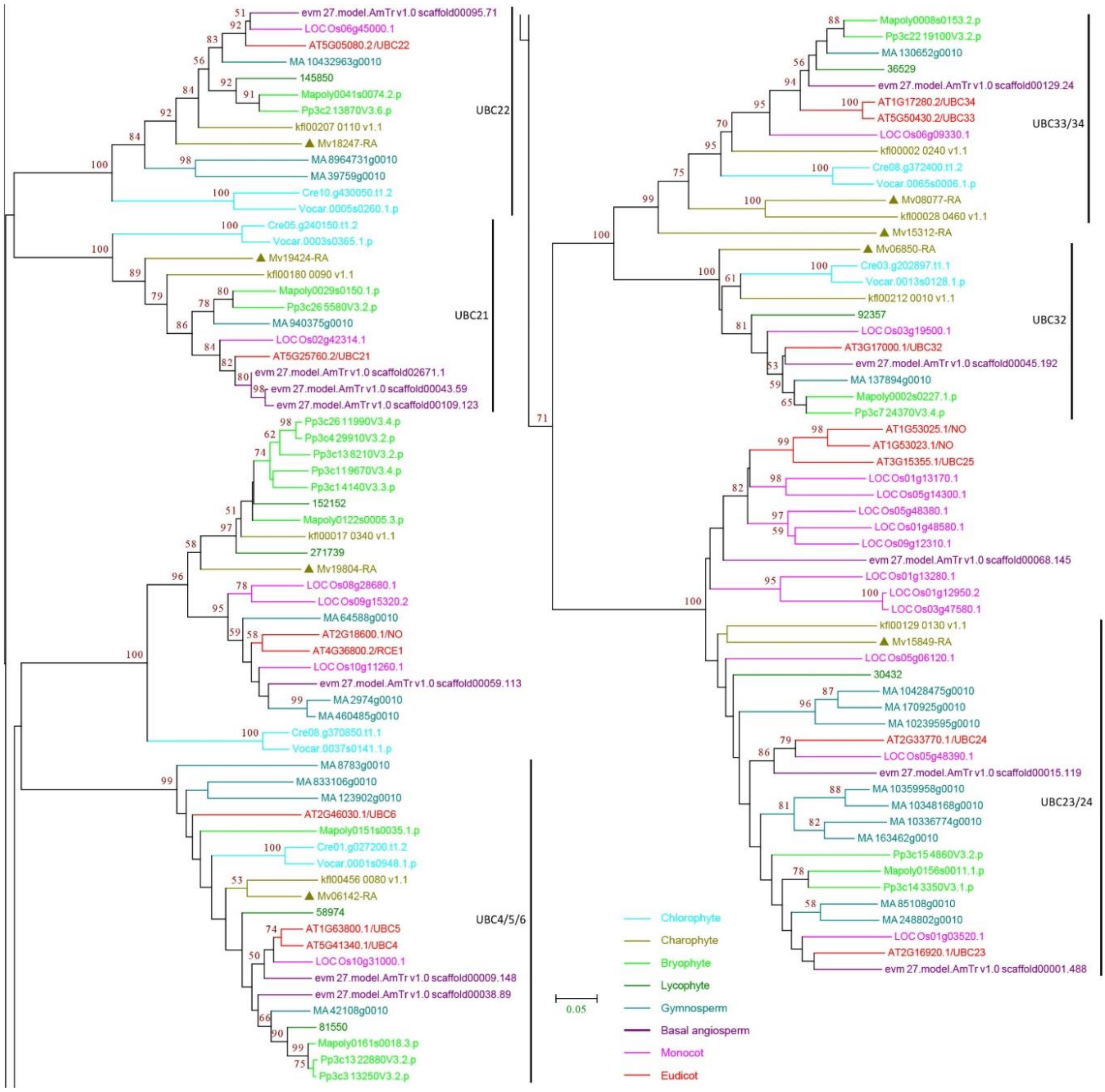

Data S1AH. Phylogenetic relationships of UBIQUITIN-CONJUGATING ENZYME (UBC) proteins from *M. viride* and other evolutionarily representative species. The *M. viride* sequences were highlighted by triangles. Numbers on the major branches indicate bootstrap values (> 50%) in 1,000 replicates.

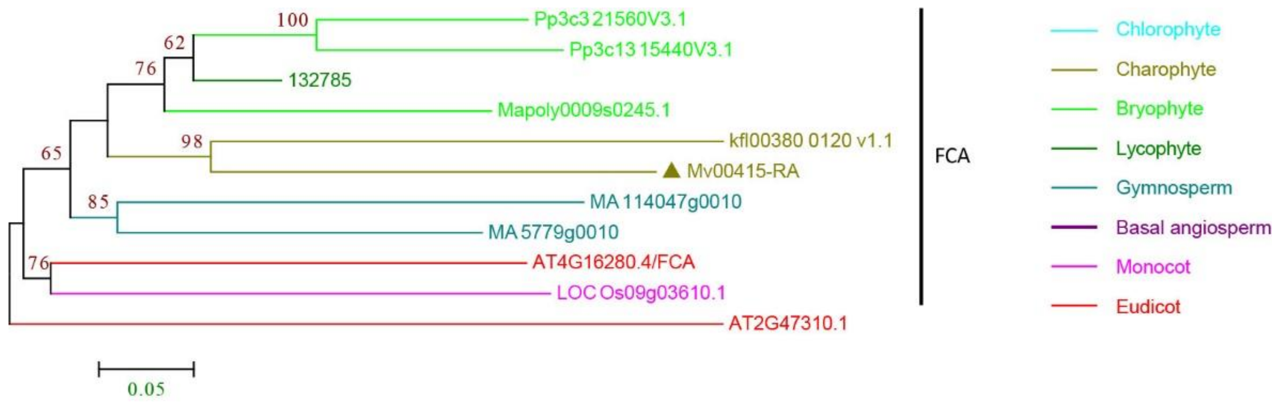

Data S1AI. Phylogenetic relationships of FLOWERING CONTROL LOCUS A (FCA) proteins from *M. viride* and other evolutionarily representative species. The *M. viride* sequence was highlighted by a triangle. Numbers on the major branches indicate bootstrap values (> 50%) in 1,000 replicates.

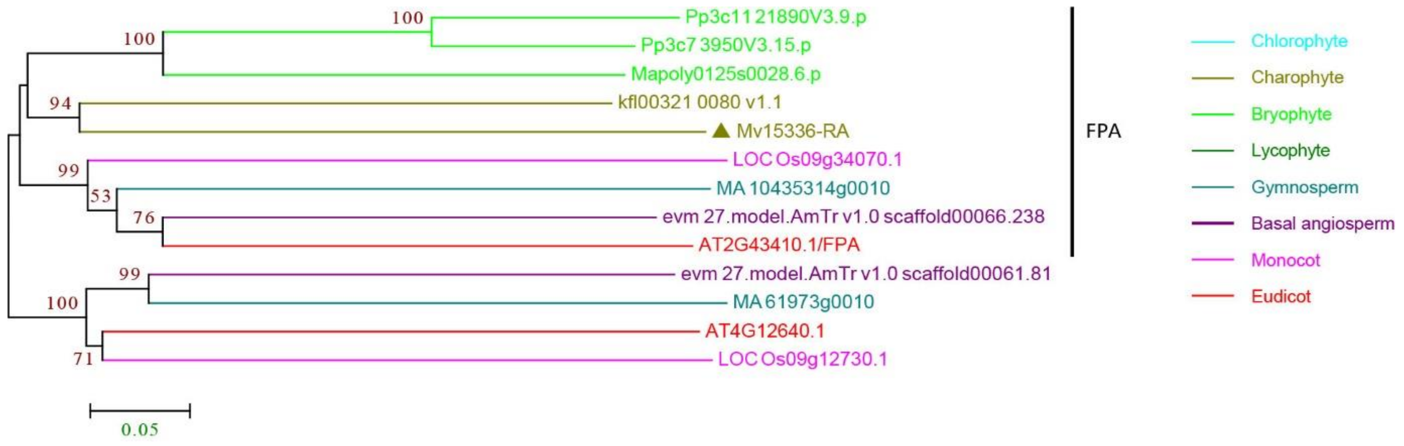

Data S1AJ. Phylogenetic relationships of FPA proteins from *M. viride* and other evolutionarily representative species. The *M. viride* sequence was highlighted by a triangle. Numbers on the major branches indicate bootstrap values (> 50%) in 1,000 replicates.

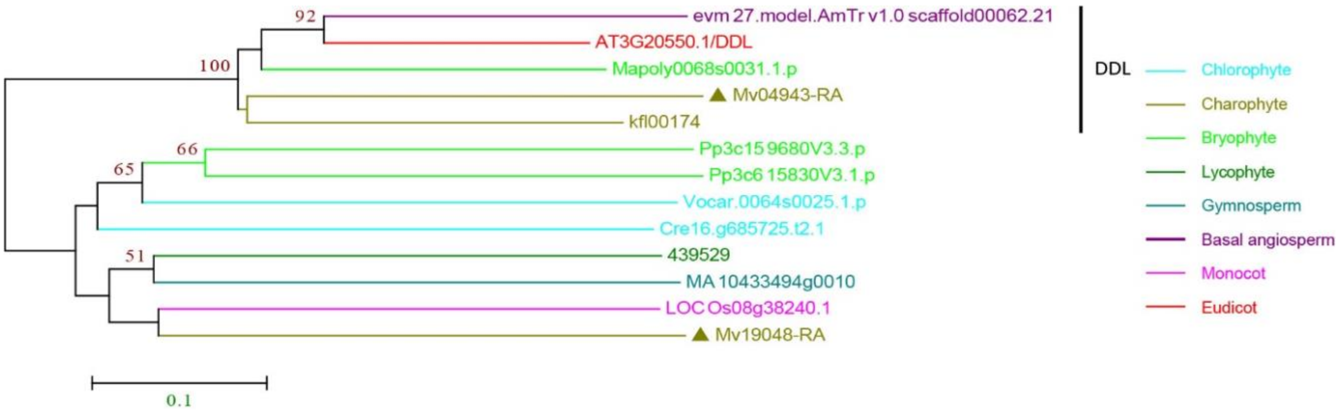

Data S1AK. Phylogenetic relationships of DAWDLE (DDL) proteins from *M. viride* and other evolutionarily representative species. The *M. viride* sequences were highlighted by triangles. Numbers on the major branches indicate bootstrap values (> 50%) in 1,000 replicates.

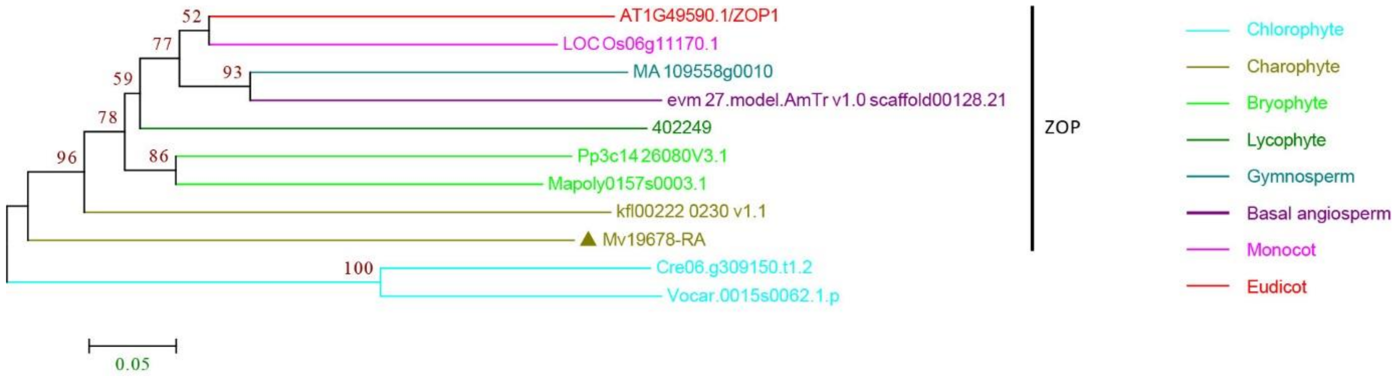

Data S1AL. Phylogenetic relationships of ZINC-FINGER AND OCRE DOMAIN-CONTAINING PROTEIN 1 (ZOP1) proteins from *M. viride* and other evolutionarily representative species. The *M. viride* sequence was highlighted by a triangle. Numbers on the major branches indicate bootstrap values (> 50%) in 1,000 replicates.

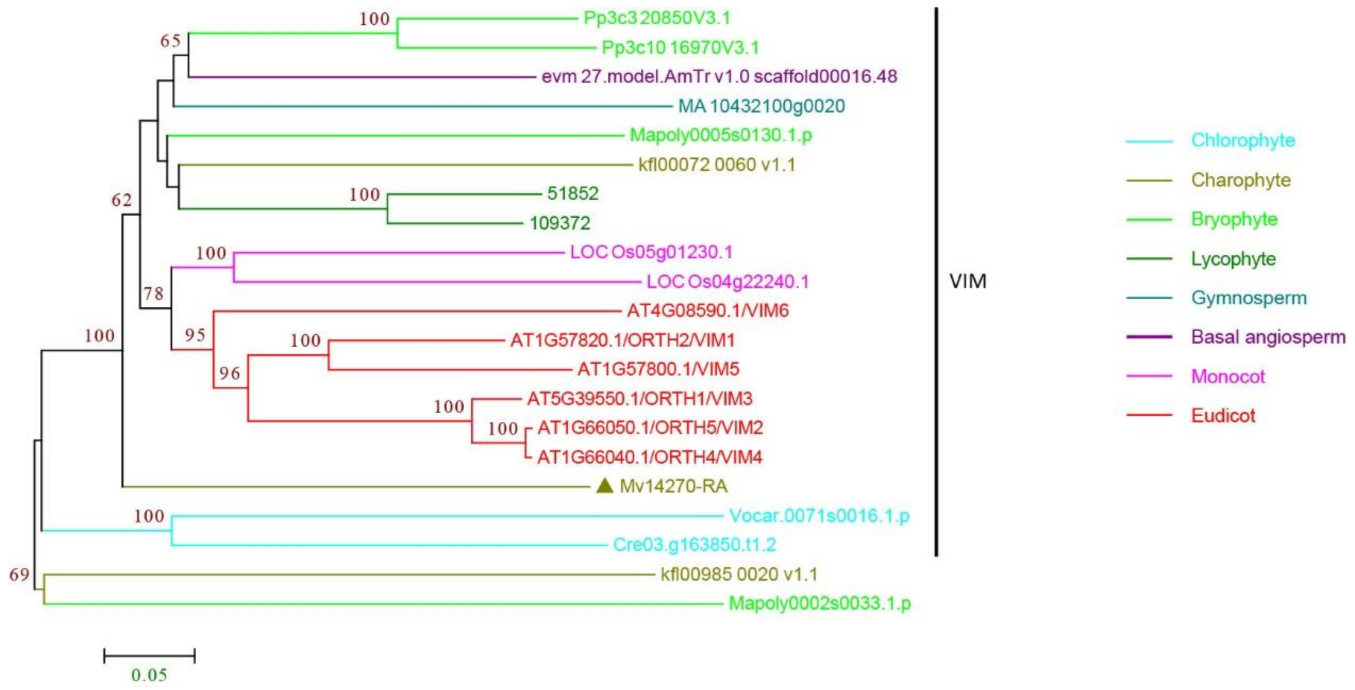

Data S1AM. Phylogenetic relationships of VARIANT IN METHYLATION (VIM) proteins from *M. viride* and other evolutionarily representative species. The *M. viride* sequence was highlighted by a triangle. Numbers on the major branches indicate bootstrap values (> 50%) in 1,000 replicates.

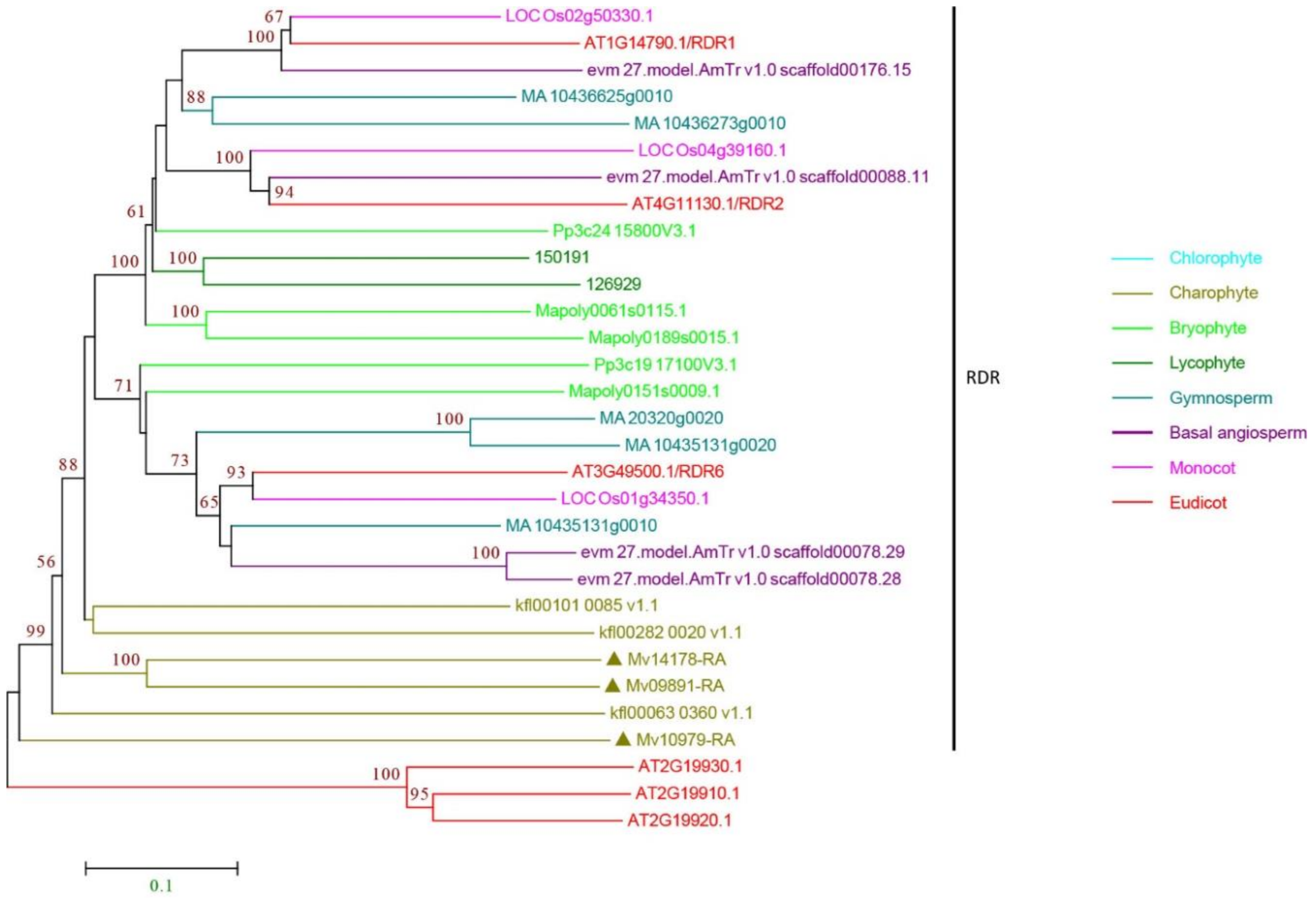

Data S1AN. Phylogenetic relationships of RNA-DEPENDENT RNA POLYMERASE (RDR) proteins from *M. viride* and other evolutionarily representative species. The *M. viride* sequences were highlighted by triangles. Numbers on the major branches indicate bootstrap values (> 50%) in 1,000 replicates.

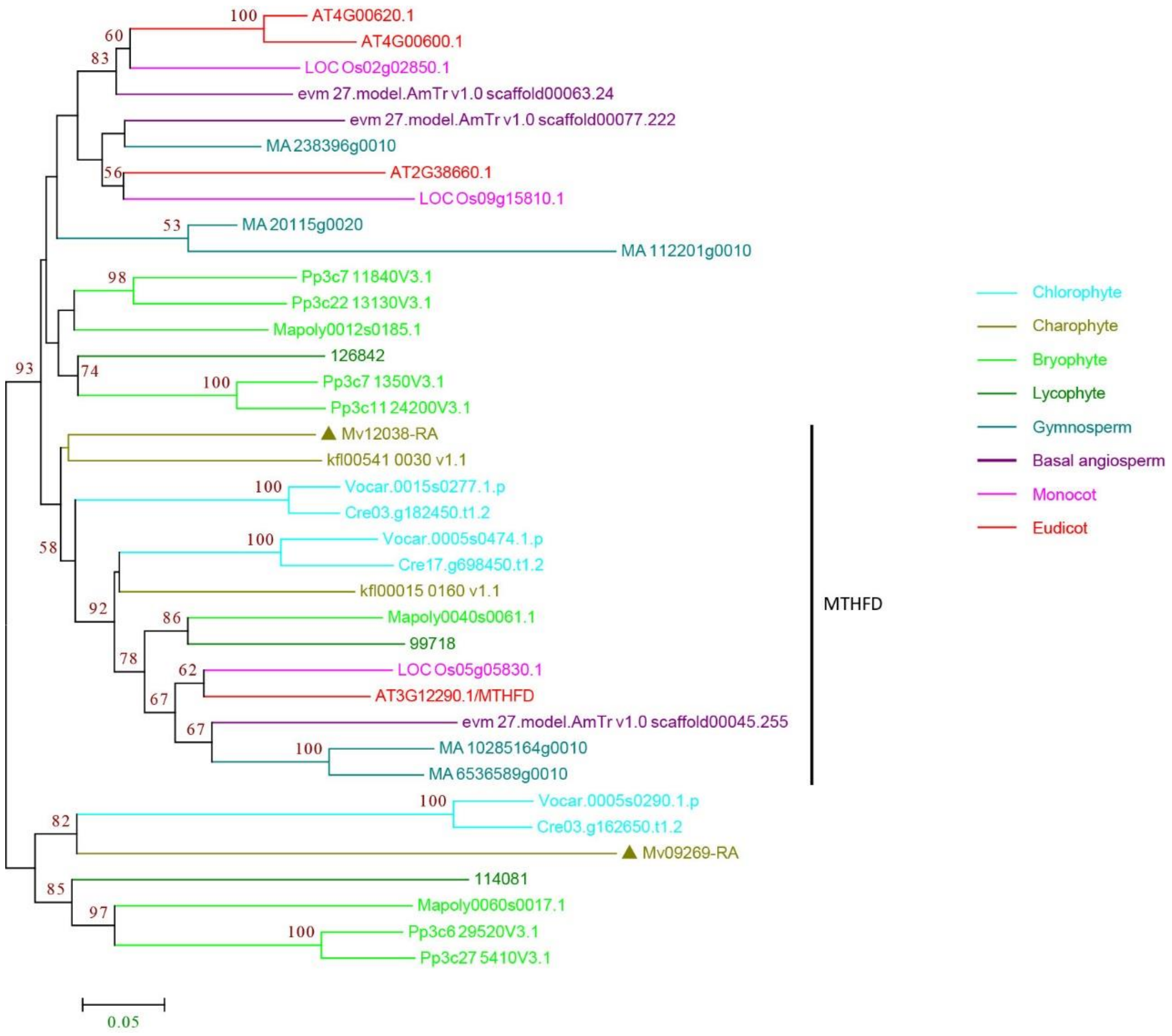

Data S1AO. Phylogenetic relationships of METHYLENETETRAHYDROFOLATE DEHYDROGENASE (MTHFD) proteins from *M. viride* and other evolutionarily representative species. The *M. viride* sequences were highlighted by triangles. Numbers on the major branches indicate bootstrap values (> 50%) in 1,000 replicates.

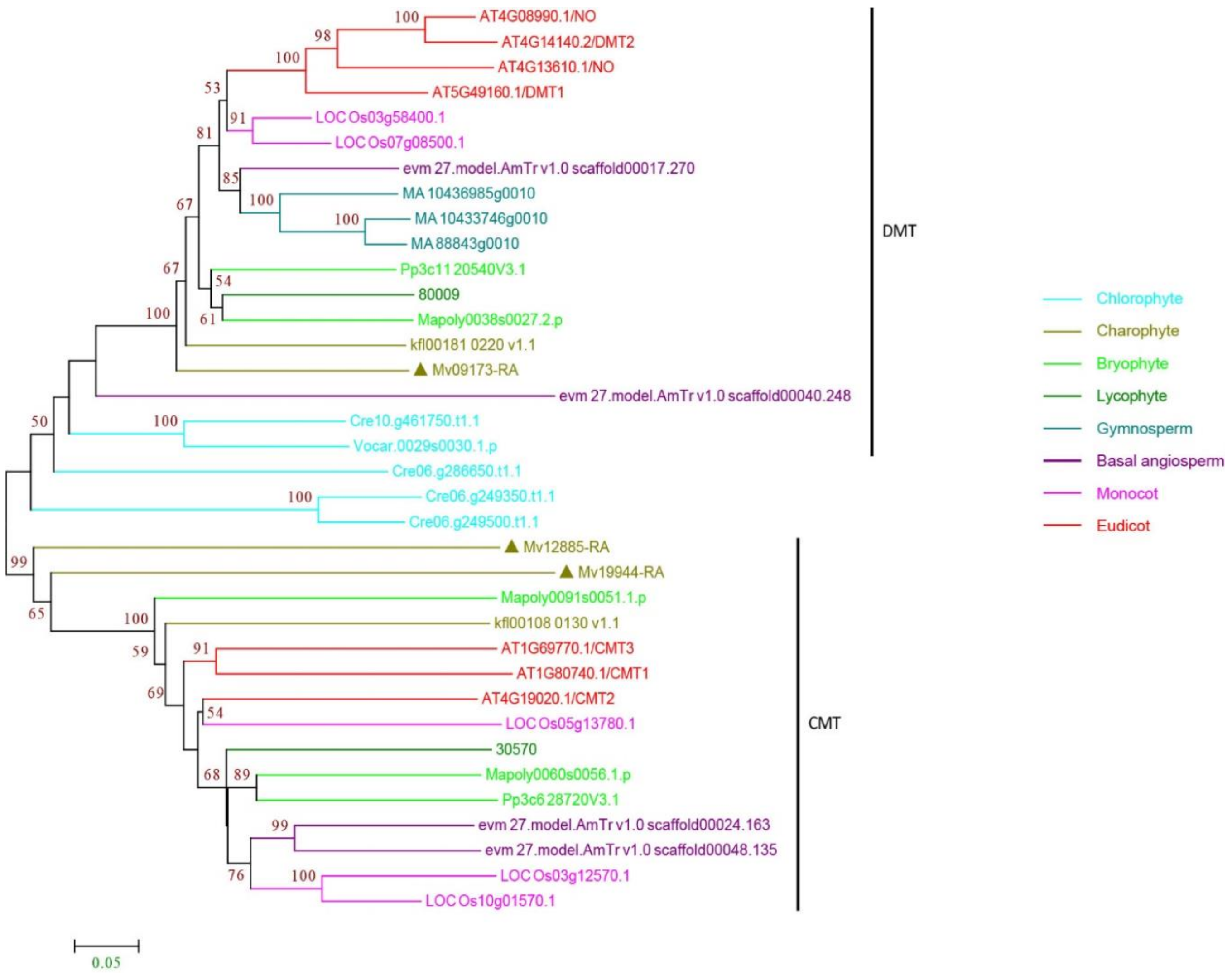

Data S1AP. Phylogenetic relationships of CHROMOMETHYLASE/DNA METHYLTRANSFERASE (CMT/DMT) proteins from *M. viride* and other evolutionarily representative species. The *M. viride* sequences were highlighted by triangles. Numbers on the major branches indicate bootstrap values (> 50%) in 1,000 replicates.

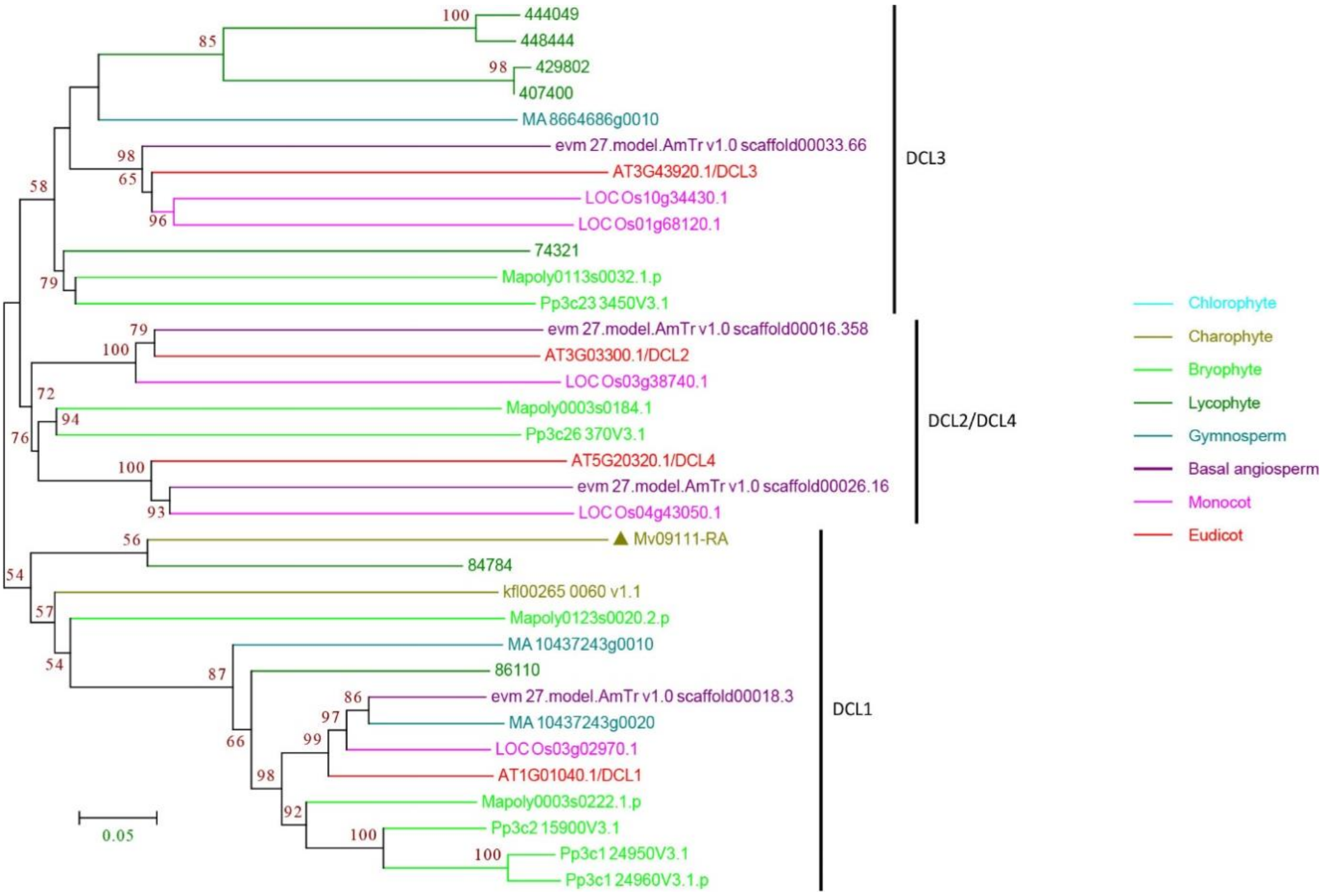

Data S1AQ. Phylogenetic relationships of DICER-LIKE (DCL) proteins from *M. viride* and other evolutionarily representative species. The *M. viride* sequence was highlighted by a triangle. Numbers on the major branches indicate bootstrap values (> 50%) in 1,000 replicates.

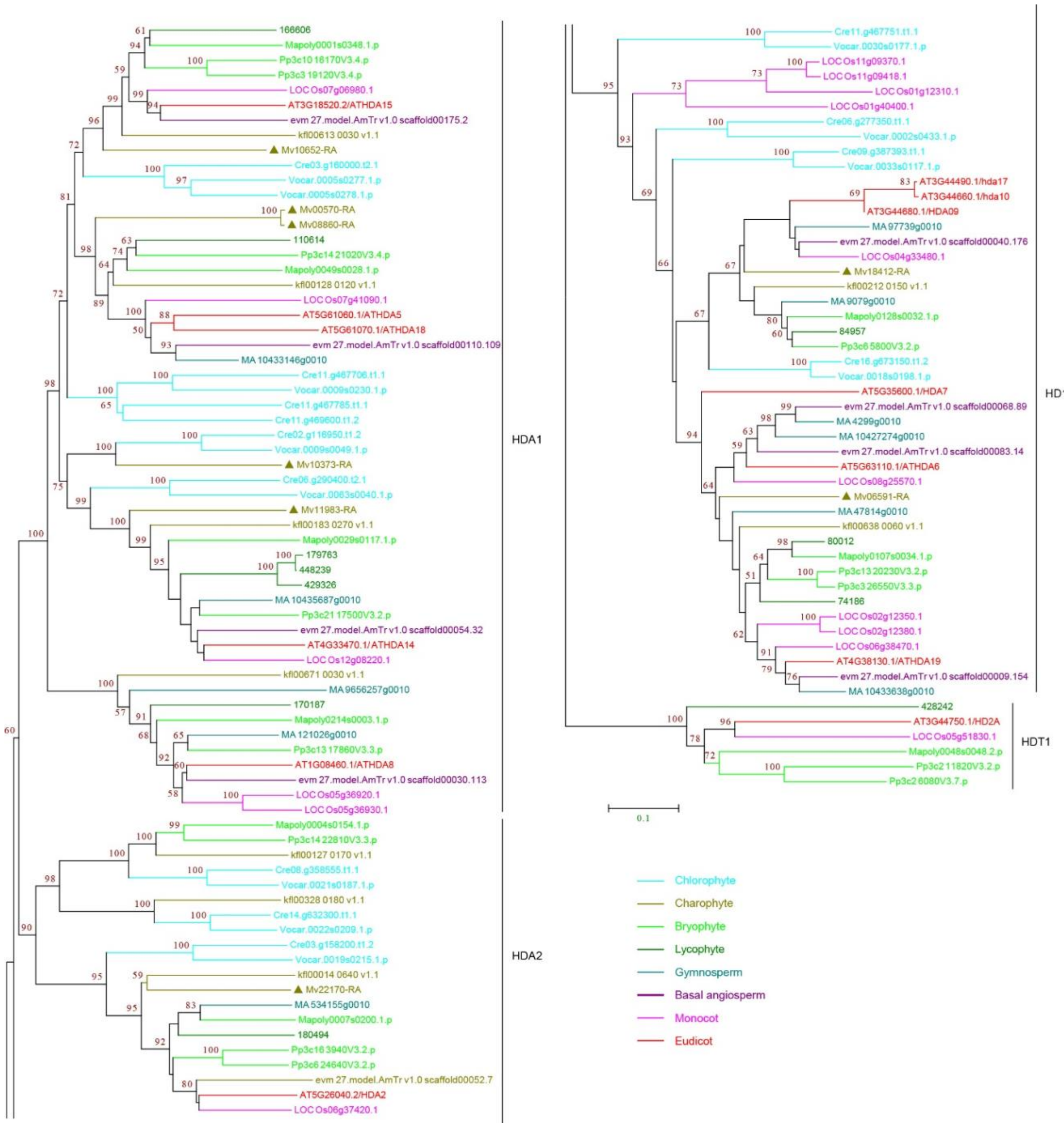

Data S1AR. Phylogenetic relationships of HISTONE DEACETYLASE (HD) proteins from *M. viride* and other evolutionarily representative species. The *M. viride* sequences were highlighted by triangles. Numbers on the major branches indicate bootstrap values (> 50%) in 1,000 replicates.

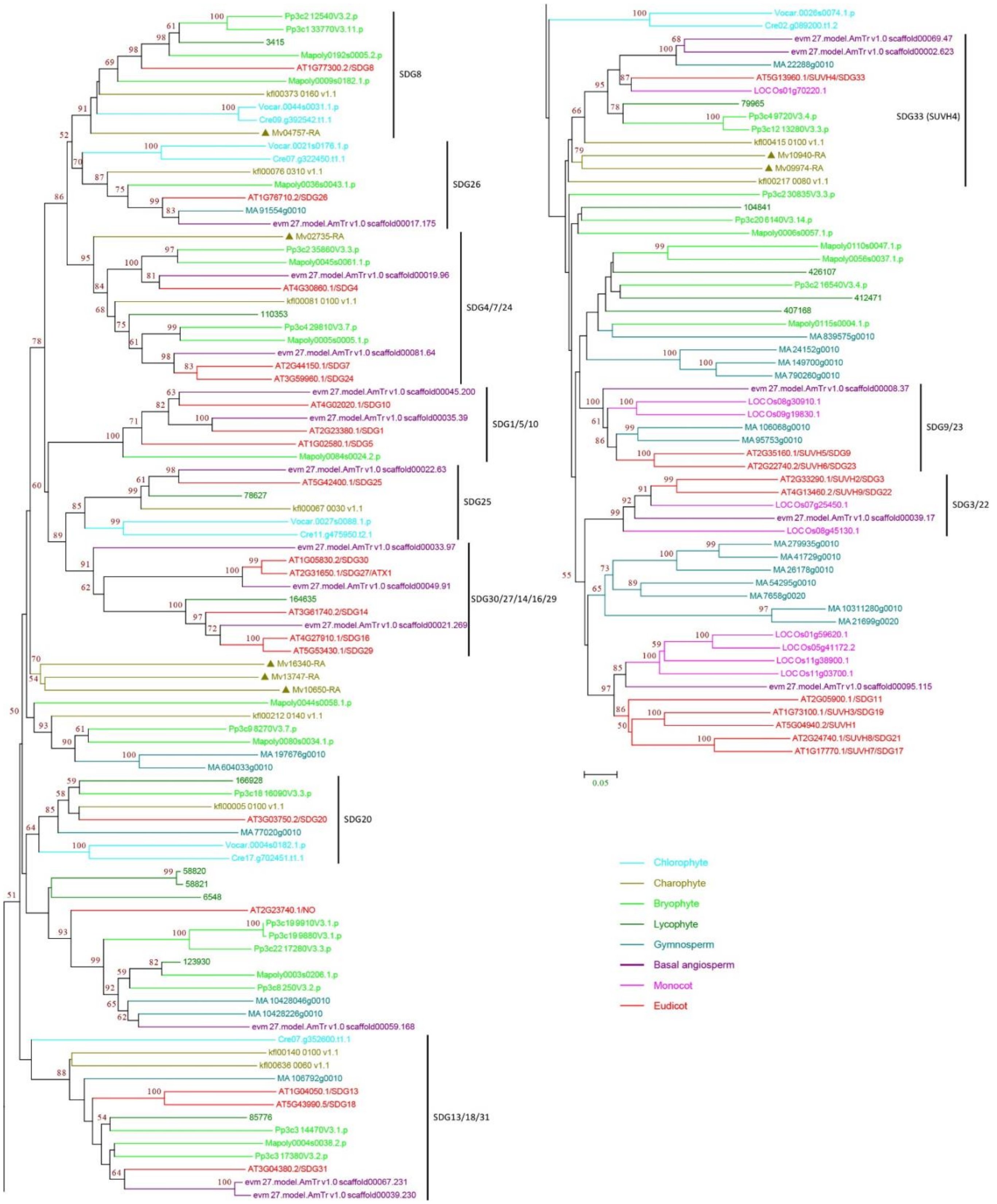

Data S1AS. Phylogenetic relationships of SET DOMAIN PROTEIN (SDG) proteins from *M. viride* and other evolutionarily representative species. The *M. viride* sequences were highlighted by triangles. Numbers on the major branches indicate bootstrap values (> 50%) in 1,000 replicates.

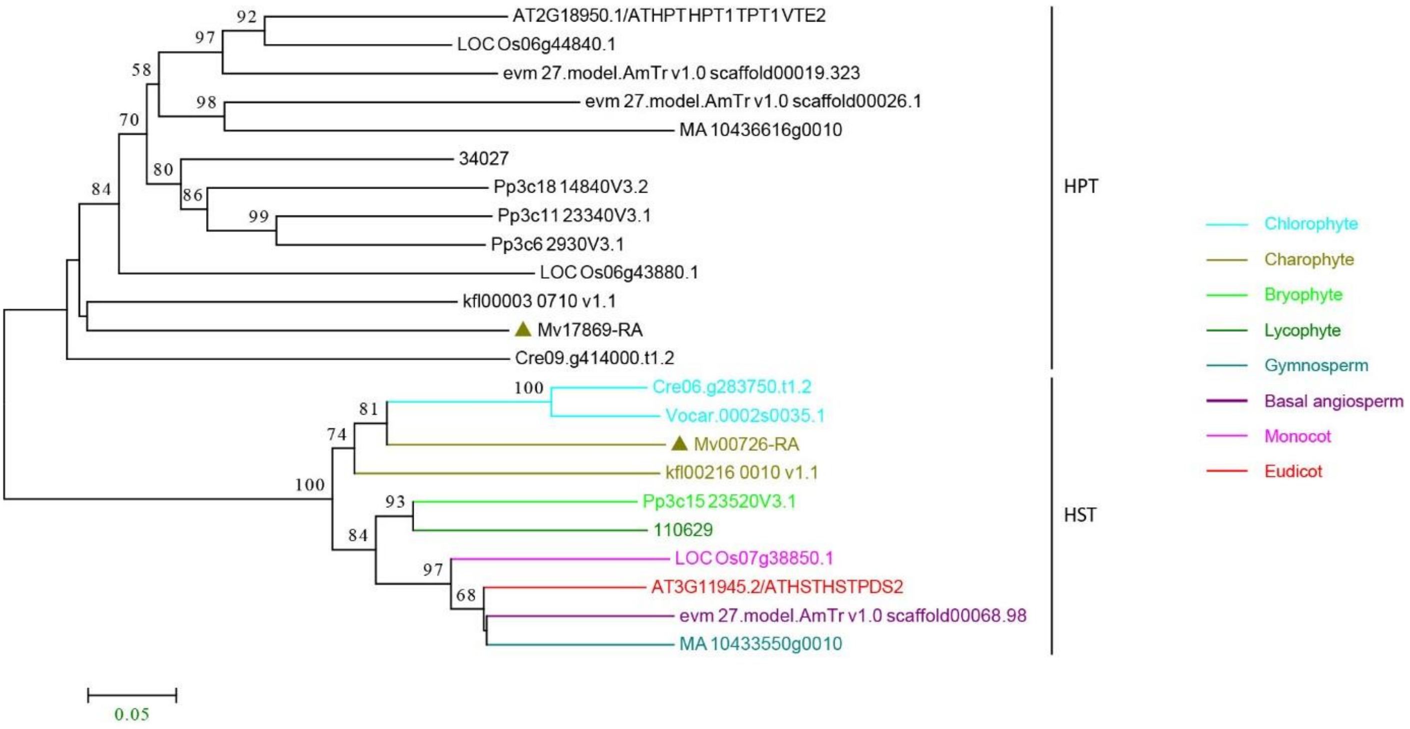

Data S1AT. Phylogenetic relationships of HOMOGENTISATE PRENYLTRANSFERASE (HST) proteins from *M. viride* and other evolutionarily representative species. The *M. viride* sequences were highlighted by triangles. Numbers on the major branches indicate bootstrap values (> 50%) in 1,000 replicates.

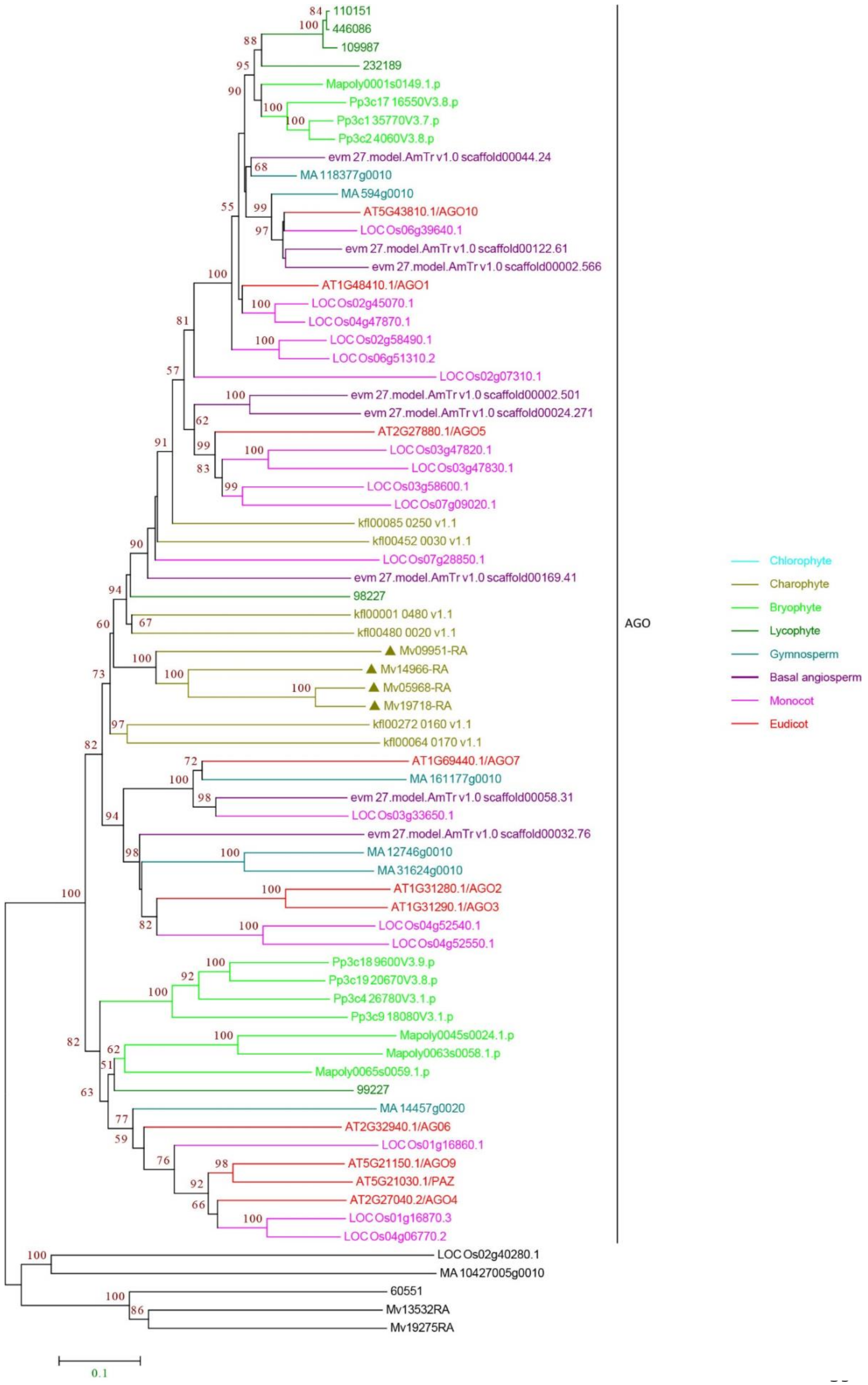
110151
446086
109987
232189
Mapoly0001s0149.1.p
Pp3c17 16550V3.8.p
Pp3c1 35770V3.7.p
Pp3c2 4060V3.8.p
evm 27.model.AmTr v1.0 scaffold00044.24
MA 118377g0010
MA 594g0010
AT5G43810.1/AGO10
LOC Os06g39640.1
evm 27.model.AmTr v1.0 scaffold00122.61
evm 27.model.AmTr v1.0 scaffold00002.566
AT1G48410.1/AGO1
LOC Os02g45070.1
LOC Os04g47870.1
LOC Os02g58490.1
LOC Os06g51310.2
LOC Os02g07310.1
evm 27.model.AmTr v1.0 scaffold00002.501
evm 27.model.AmTr v1.0 scaffold00024.271
AT2G27880.1/AGO5
LOC Os03g47820.1
LOC Os03g47830.1
LOC Os03g58600.1
LOC Os07g09020.1
kfl00085 0250 v1.1
kfl00452 0030 v1.1
LOC Os07g28850.1
evm 27.model.AmTr v1.0 scaffold00169.41
98227
kfl00001 0480 v1.1
kfl00480 0020 v1.1
Mv09951-RA
Mv14966-RA
Mv05968-RA
Mv19718-RA
kfl00272 0160 v1.1
kfl00064 0170 v1.1
AT1G69440.1/AGO7
MA 161177g0010
evm 27.model.AmTr v1.0 scaffold00058.31
LOC Os03g33650.1
evm 27.model.AmTr v1.0 scaffold00032.76
MA 12746g0010
MA 31624g0010
AT1G31280.1/AGO2
AT1G31290.1/AGO3
LOC Os04g52540.1
LOC Os04g52550.1
Pp3c18 9600V3.9.p
Pp3c19 20670V3.8.p
Pp3c4 26780V3.1.p
Pp3c9 18080V3.1.p
Mapoly0045s0024.1.p
Mapoly0063s0058.1.p
Mapoly0065s0059.1.p
99227
MA 14457g0020
AT2G32940.1/AG06
LOC Os01g16860.1
AT5G21150.1/AGO9
AT5G21030.1/PAZ
AT2G27040.2/AGO4
LOC Os01g16870.3
LOC Os04g06770.2
LOC Os02g40280.1
MA 10427005g0010
60551
Mv13532RA
Mv19275RA
AGO
0.1
Chlorophyte
Charophyte
Bryophyte
Lycophyte
Gymnosperm
Basal angiosperm
Monocot
Eudicot

Data S1AU. Phylogenetic relationships of ARGONAUTE (AGO) proteins from *M. viride* and other evolutionarily representative species. The *M. viride* sequences were highlighted by triangles. Numbers on the major branches indicate bootstrap values (> 50%) in 1,000 replicates.

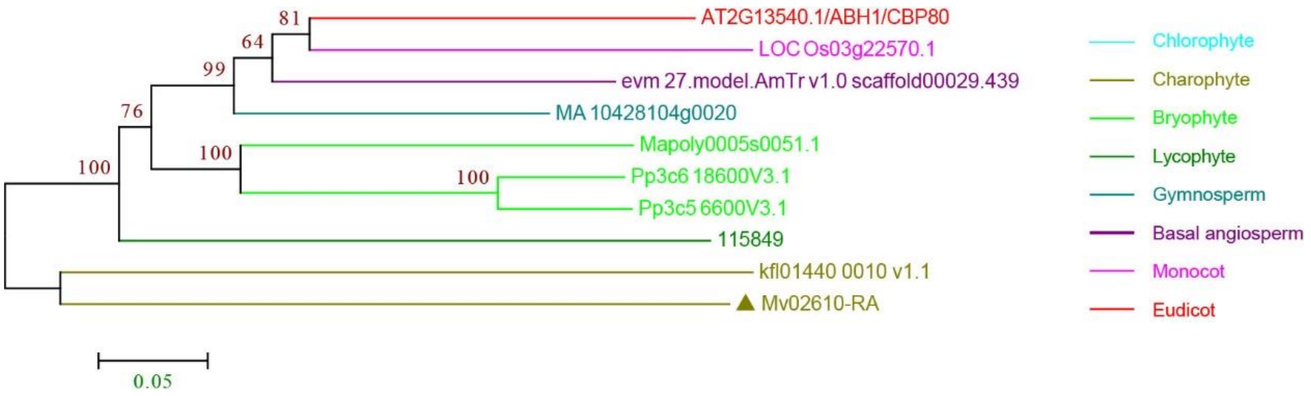

Data S1AV. Phylogenetic relationships of ABA HYPERSENSITIVE 1 (ABH1) proteins from *M. viride* and other evolutionarily representative species. The *M. viride* sequence was highlighted by a triangle. Numbers on the major branches indicate bootstrap values (> 50%) in 1,000 replicates.

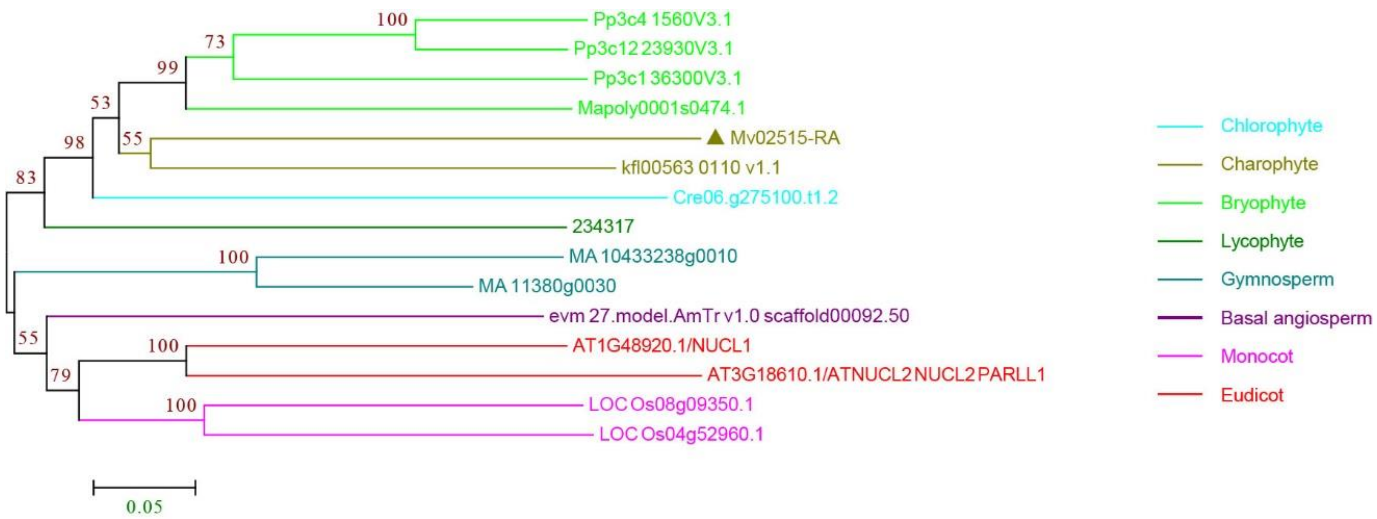

Data S1AW. Phylogenetic relationships of NUCLEOLIN-LIKE (NUCL) proteins from *M. viride* and other evolutionarily representative species. The *M. viride* sequence was highlighted by a triangle. Numbers on the major branches indicate bootstrap values (> 50%) in 1,000 replicates.

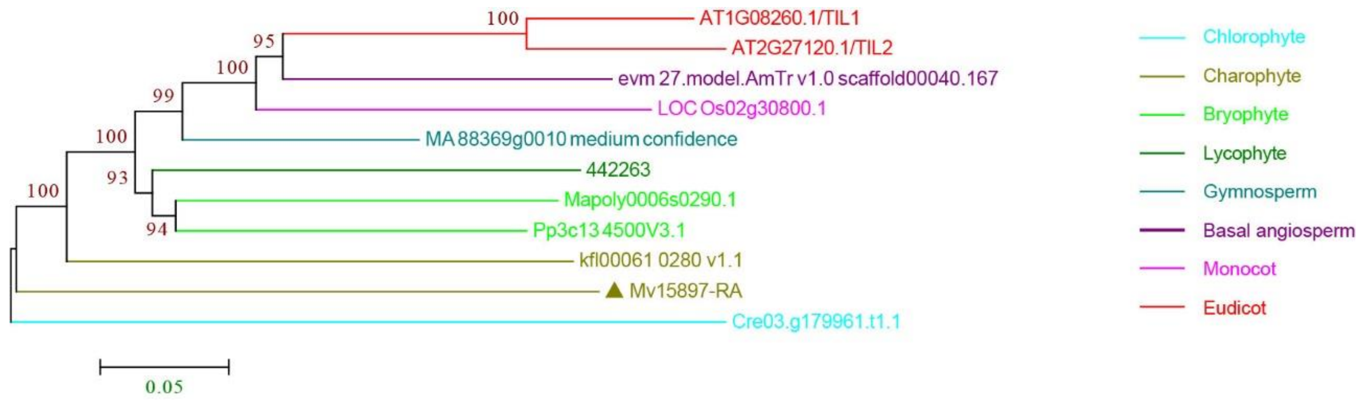

Data S1AX. Phylogenetic relationships of EARLY IN SHORT DAYS 7 (ESD7 or TIL) proteins from *M. viride* and other evolutionarily representative species. The *M. viride* sequence was highlighted by a triangle. Numbers on the major branches indicate bootstrap values (> 50%) in 1,000 replicates.

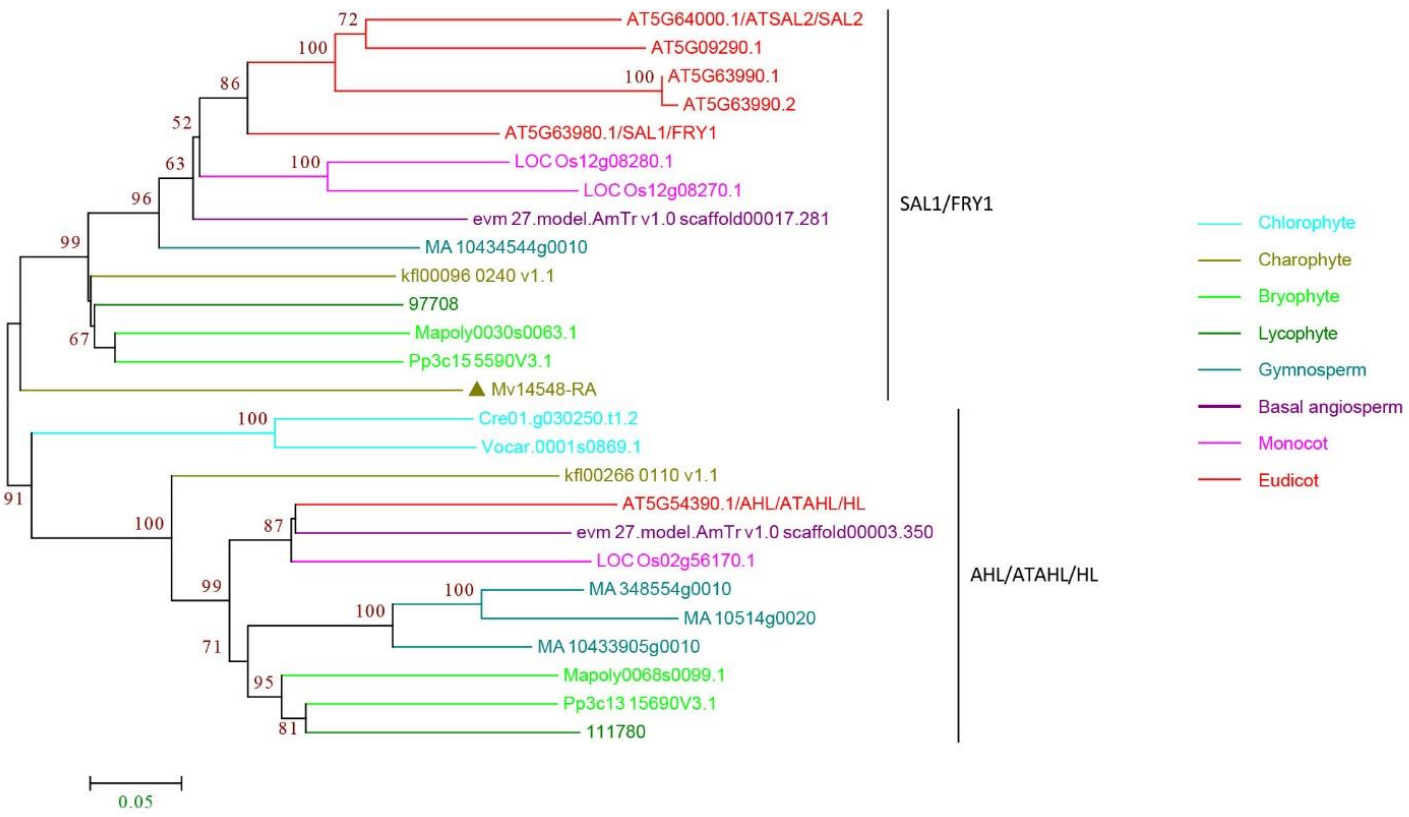

Data S1AY. Phylogenetic relationships of SAL1 proteins from *M. viride* and other evolutionarily representative species. The *M. viride* sequence was highlighted by a triangle. Numbers on the major branches indicate bootstrap values (> 50%) in 1,000 replicates.

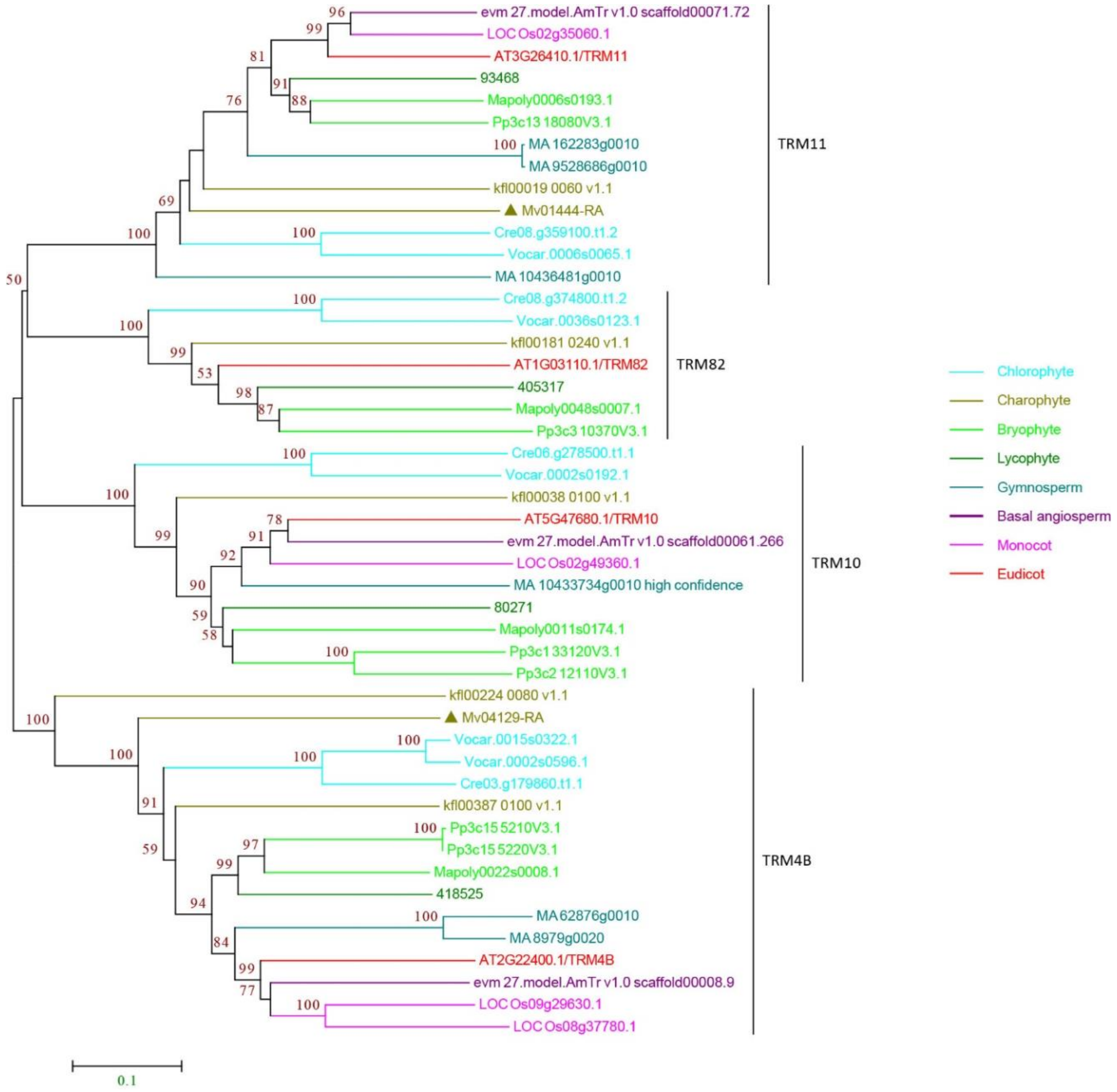

Data S1AZ. Phylogenetic relationships of TRNA MODIFICATION (TRM) proteins from *M. viride* and other evolutionarily representative species. The *M. viride* sequences were highlighted by triangles. Numbers on the major branches indicate bootstrap values (> 50%) in 1,000 replicates.

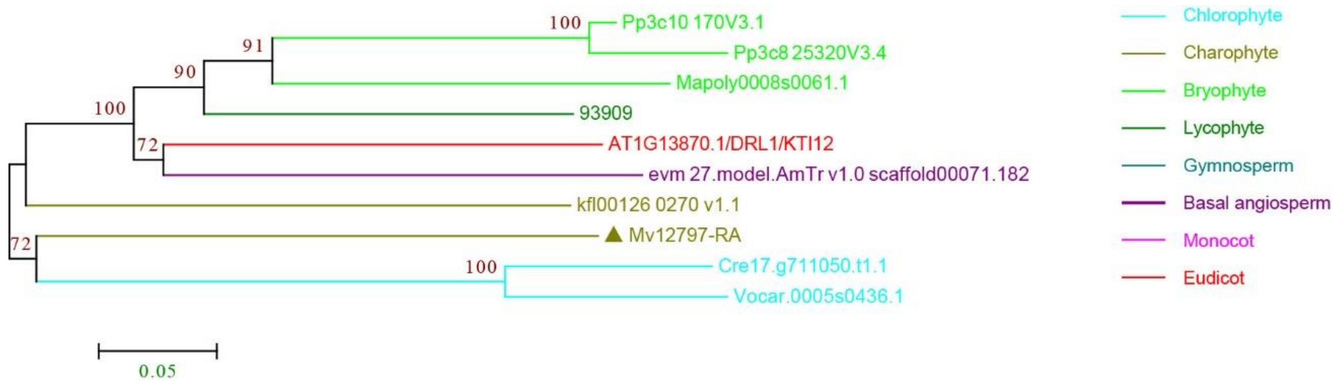

Data S1BA. Phylogenetic relationships of DEFORMED ROOTS AND LEAVES 1 (DRL1) proteins from *M. viride* and other evolutionarily representative species. The *M. viride* sequence was highlighted by a triangle. Numbers on the major branches indicate bootstrap values (> 50%) in 1,000 replicates.

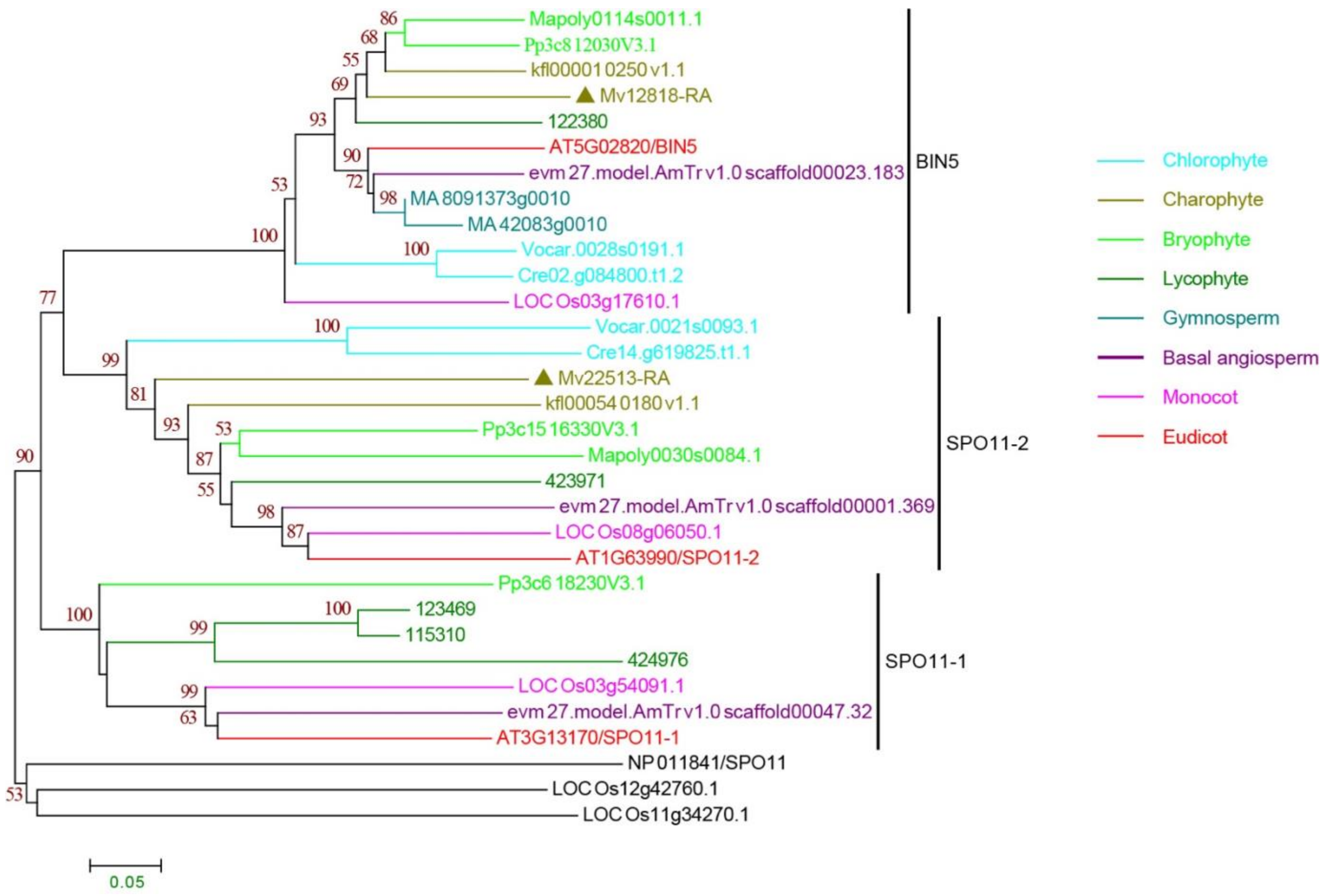

Data S1BB. Phylogenetic relationships of SPO11 proteins from *M. viride* and other evolutionarily representative species. The *M. viride* sequence was highlighted by a triangle. Numbers on the major branches indicate bootstrap values (> 50%) in 1,000 replicates.

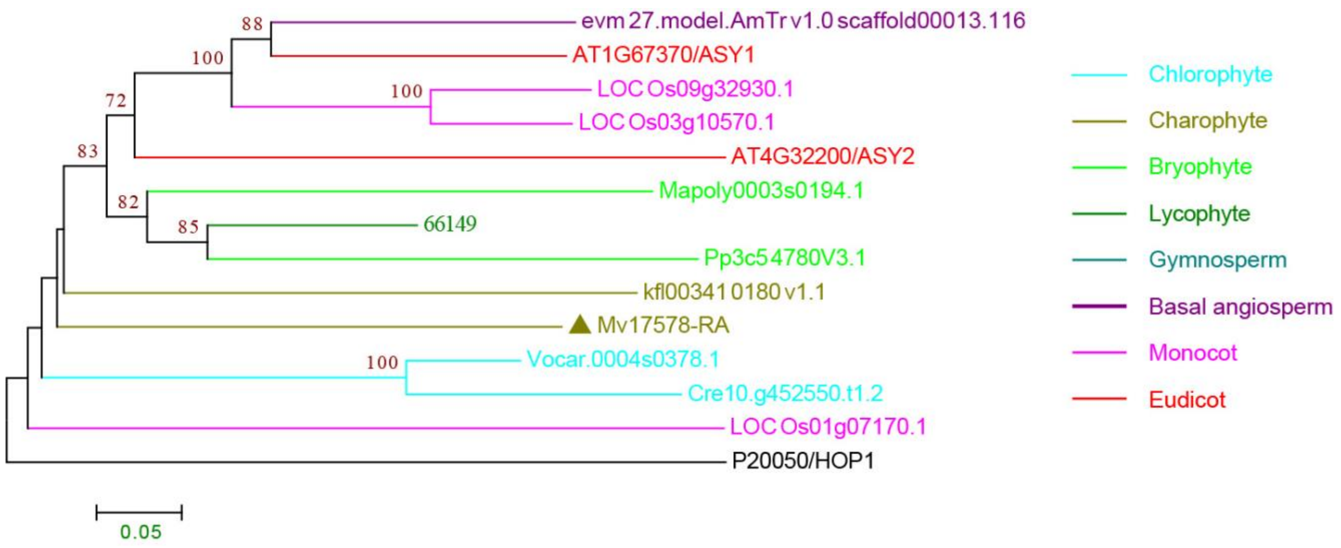

Data S1BC. Phylogenetic relationships of HOP1 proteins from *M. viride* and other evolutionarily representative species. The *M. viride* sequence was highlighted by a triangle. Numbers on the major branches indicate bootstrap values (> 50%) in 1,000 replicates.

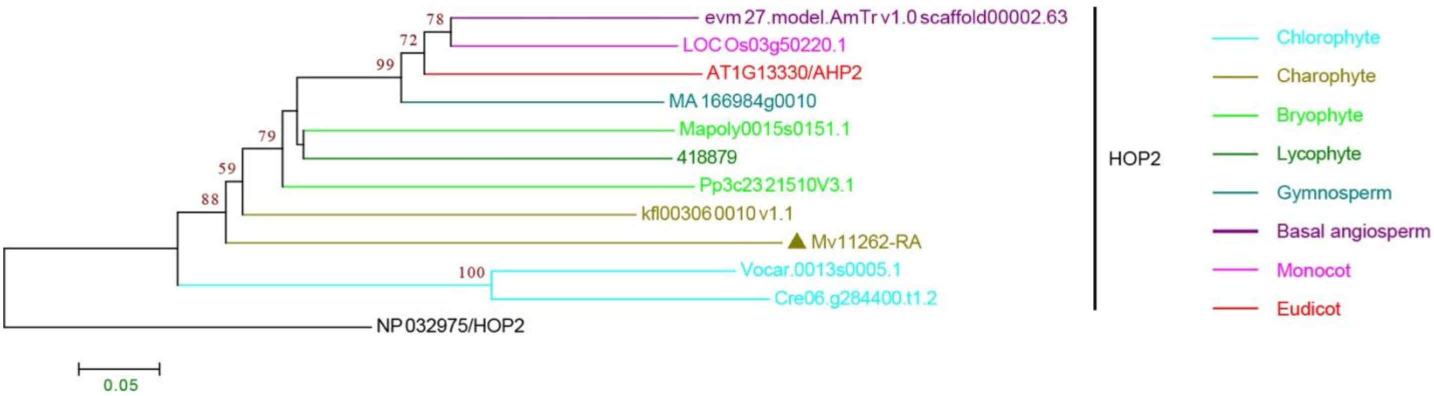

Data S1BD. Phylogenetic relationships of HOP2 proteins from *M. viride* and other evolutionarily representative species. The *M. viride* sequence was highlighted by a triangle. Numbers on the major branches indicate bootstrap values (> 50%) in 1,000 replicates.

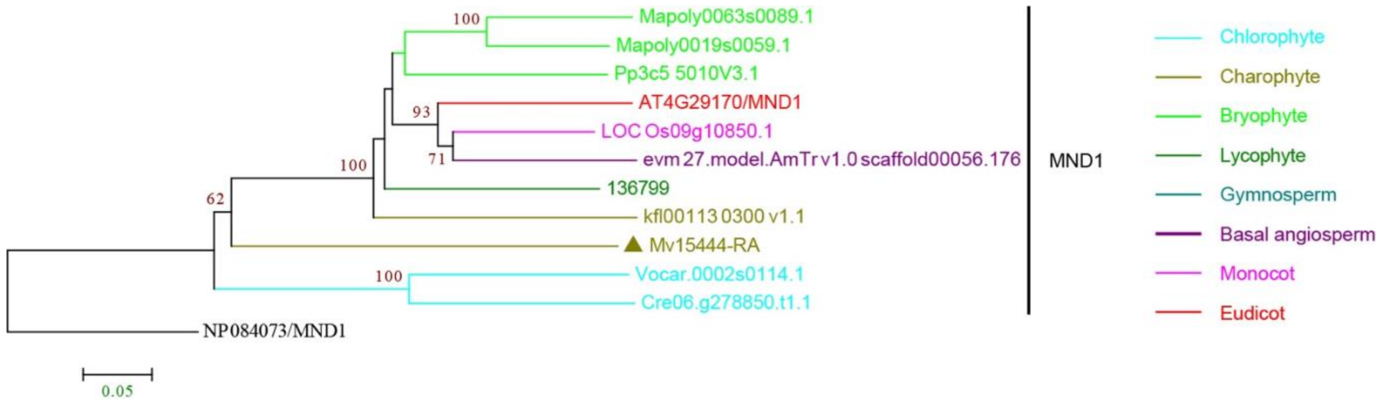

Data S1BE. Phylogenetic relationships of meiotic nuclear division protein 1 (MND1) proteins from *M. viride* and other evolutionarily representative species. The *M. viride* sequence was highlighted by a triangle. Numbers on the major branches indicate bootstrap values (> 50%) in 1,000 replicates.

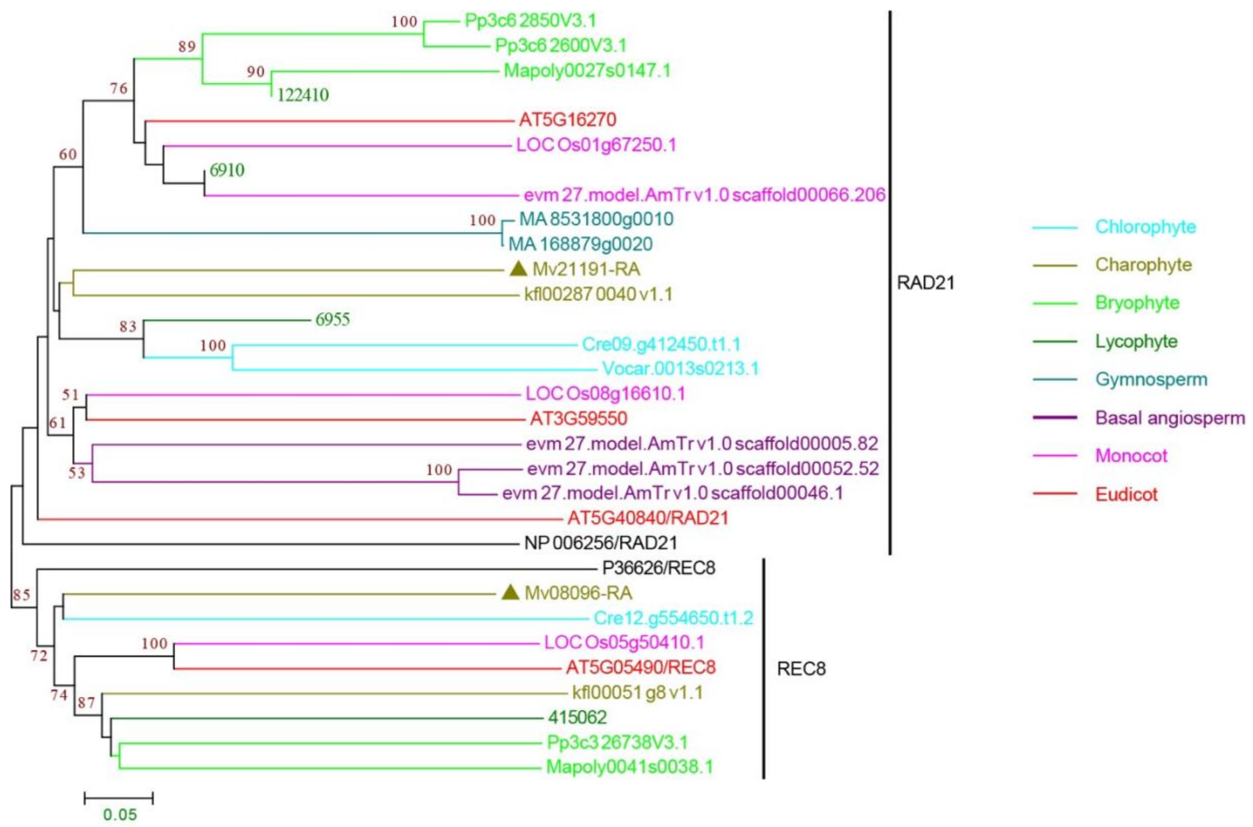

Data S1BF. Phylogenetic relationships of REC8 proteins from *M. viride* and other evolutionarily representative species. The *M. viride* sequence was highlighted by a triangle. Numbers on the major branches indicate bootstrap values (> 50%) in 1,000 replicates.

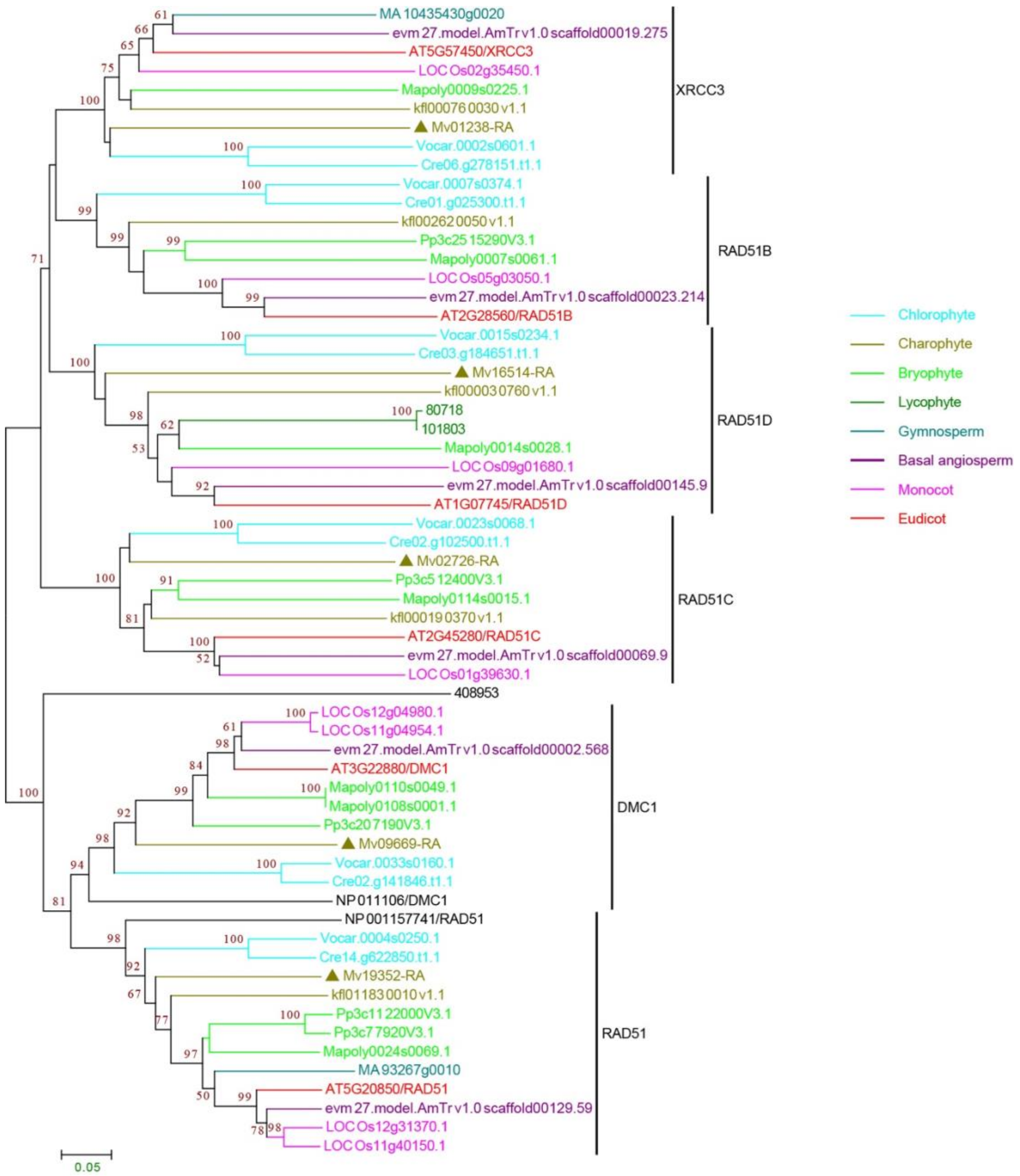

Data S1BG. Phylogenetic relationships of DMC1 proteins from *M. viride* and other evolutionarily representative species. The *M. viride* sequence was highlighted by a triangle. Numbers on the major branches indicate bootstrap values (> 50%) in 1,000 replicates.

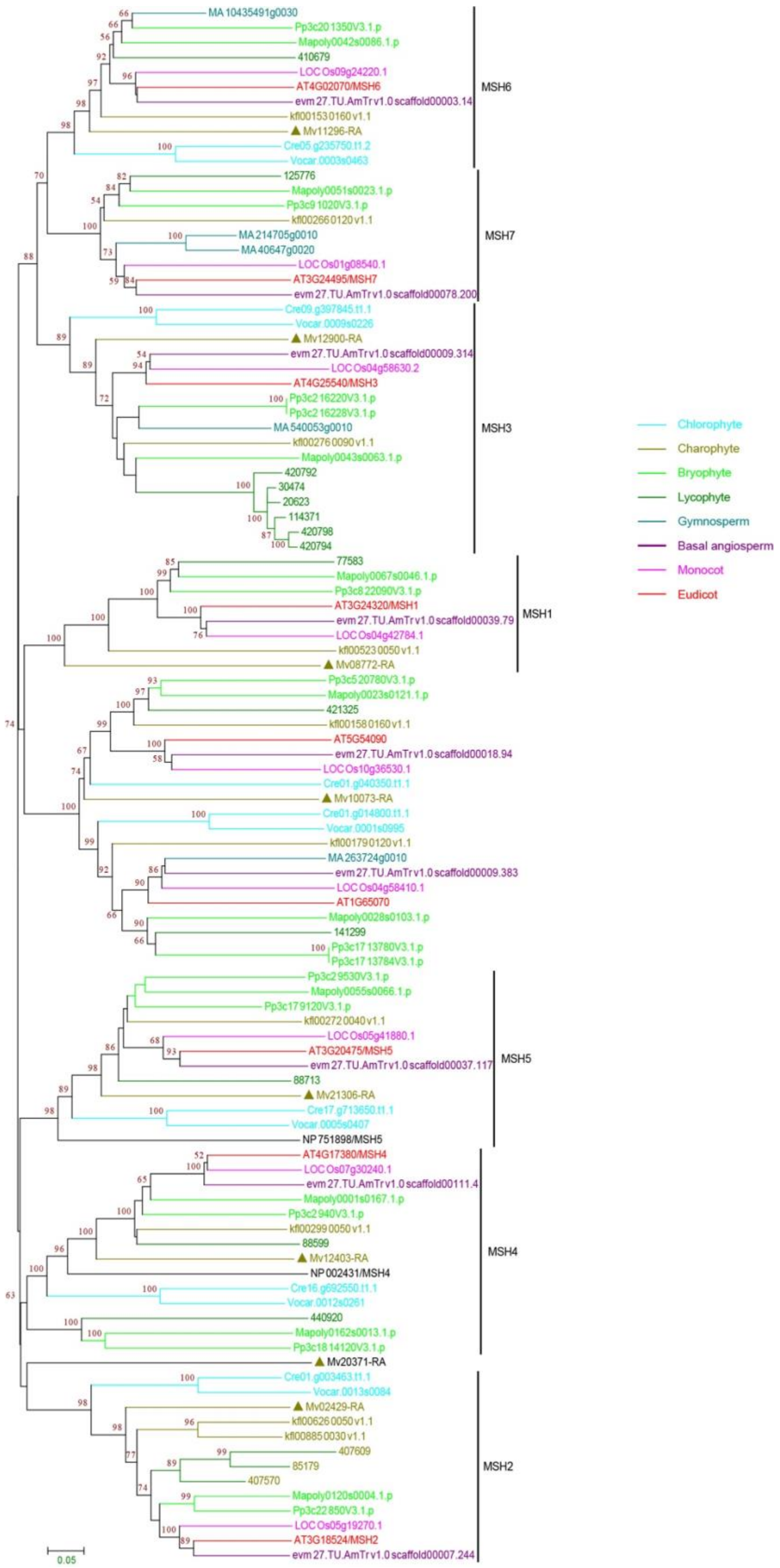

Data S1BH. Phylogenetic relationships of MutS protein homolog 4 (MSH4) proteins from *M. viride* and other evolutionarily representative species. The *M. viride* sequence was highlighted by a triangle. Numbers on the major branches indicate bootstrap values (> 50%) in 1,000 replicates.

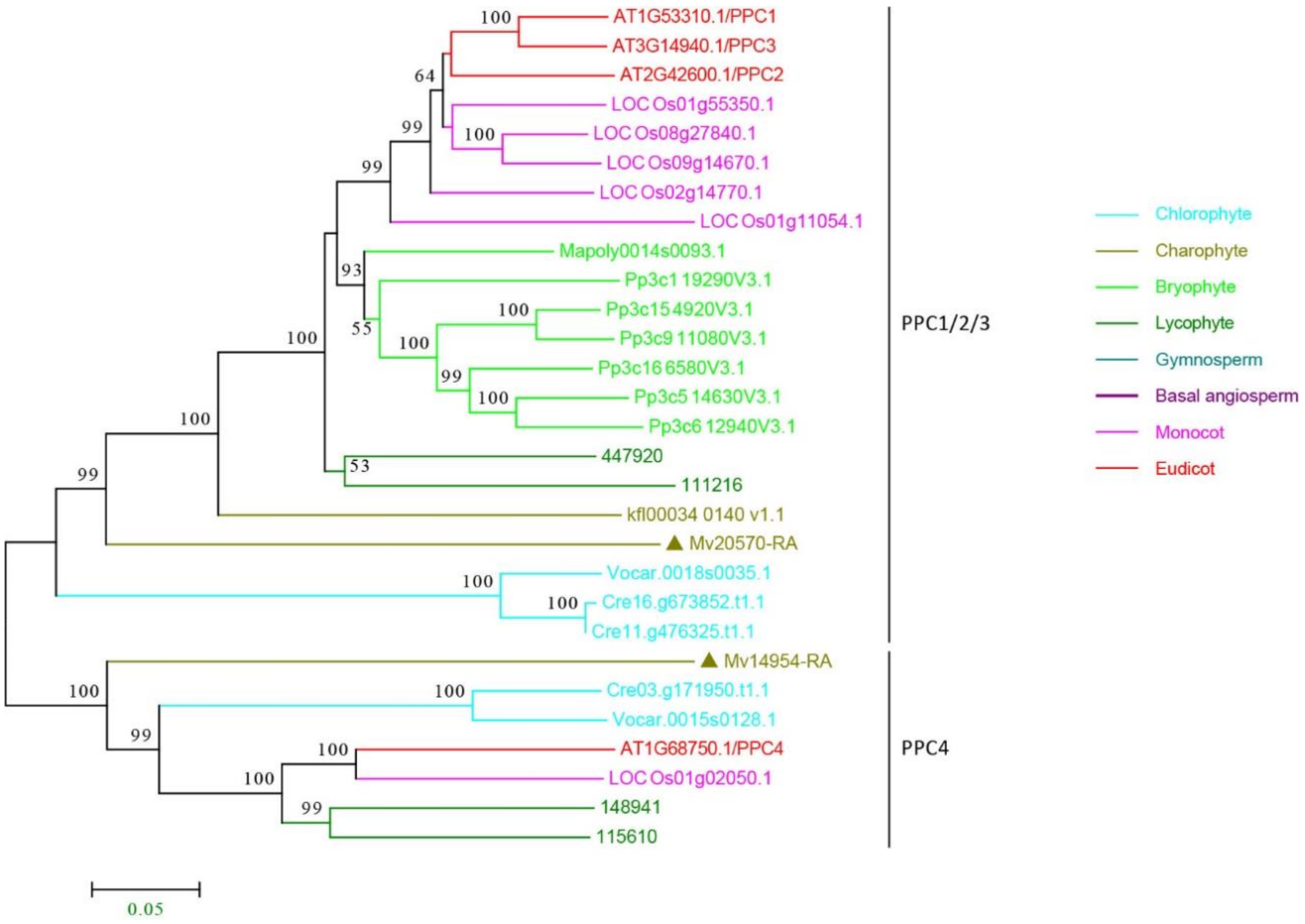

Data S1BI. Phylogenetic relationships of PHOSPHOENOLPYRUVATE CARBOXYLASE (PPC) proteins from *M. viride* and other evolutionarily representative species. The *M. viride* sequences were highlighted by triangles. Numbers on the major branches indicate bootstrap values (> 50%) in 1,000 replicates.

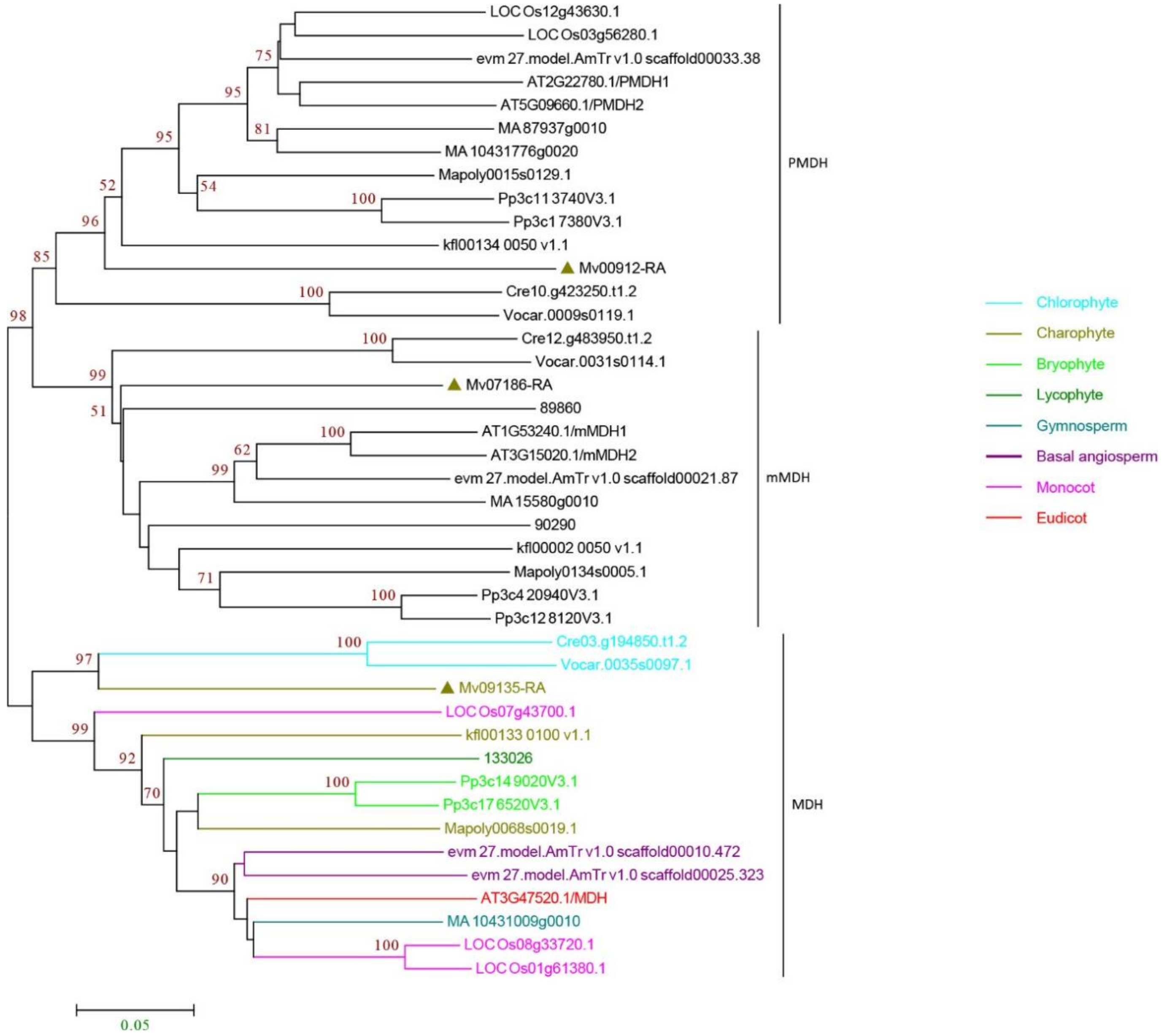

Data S1BJ. Phylogenetic relationships of MALATE DEHYDROGENASE (MDH) proteins from *M. viride* and other evolutionarily representative species. The *M. viride* sequences were highlighted by triangles. Numbers on the major branches indicate bootstrap values (> 50%) in 1,000 replicates.

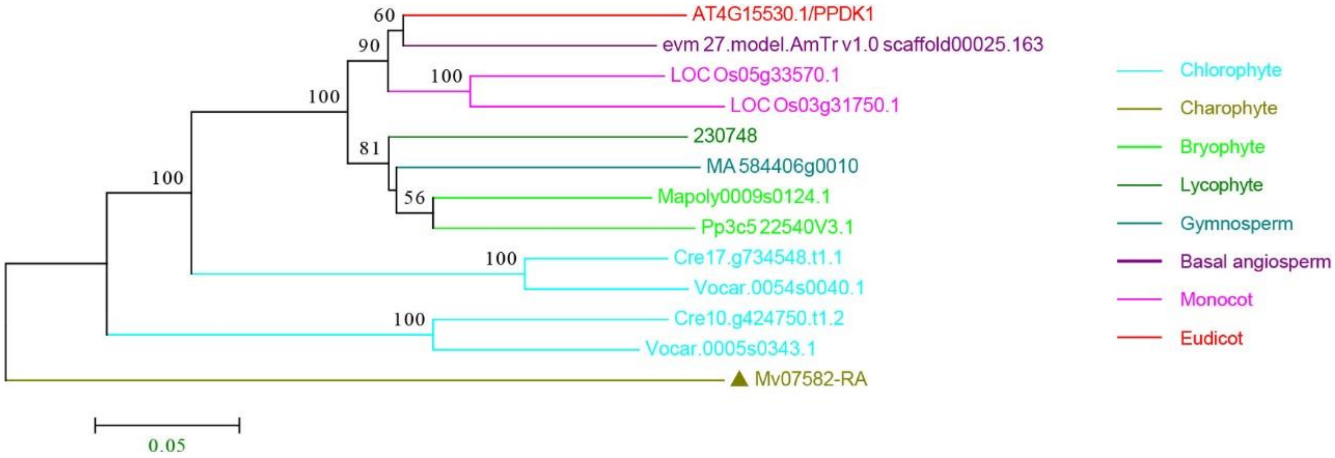

Data S1BK. Phylogenetic relationships of PYRUVATE ORTHOPHOSPHATE DIKINASE (PPDK) proteins from *M. viride* and other evolutionarily representative species. The *M. viride* sequence was highlighted by a triangle. Numbers on the major branches indicate bootstrap values (> 50%) in 1,000 replicates.

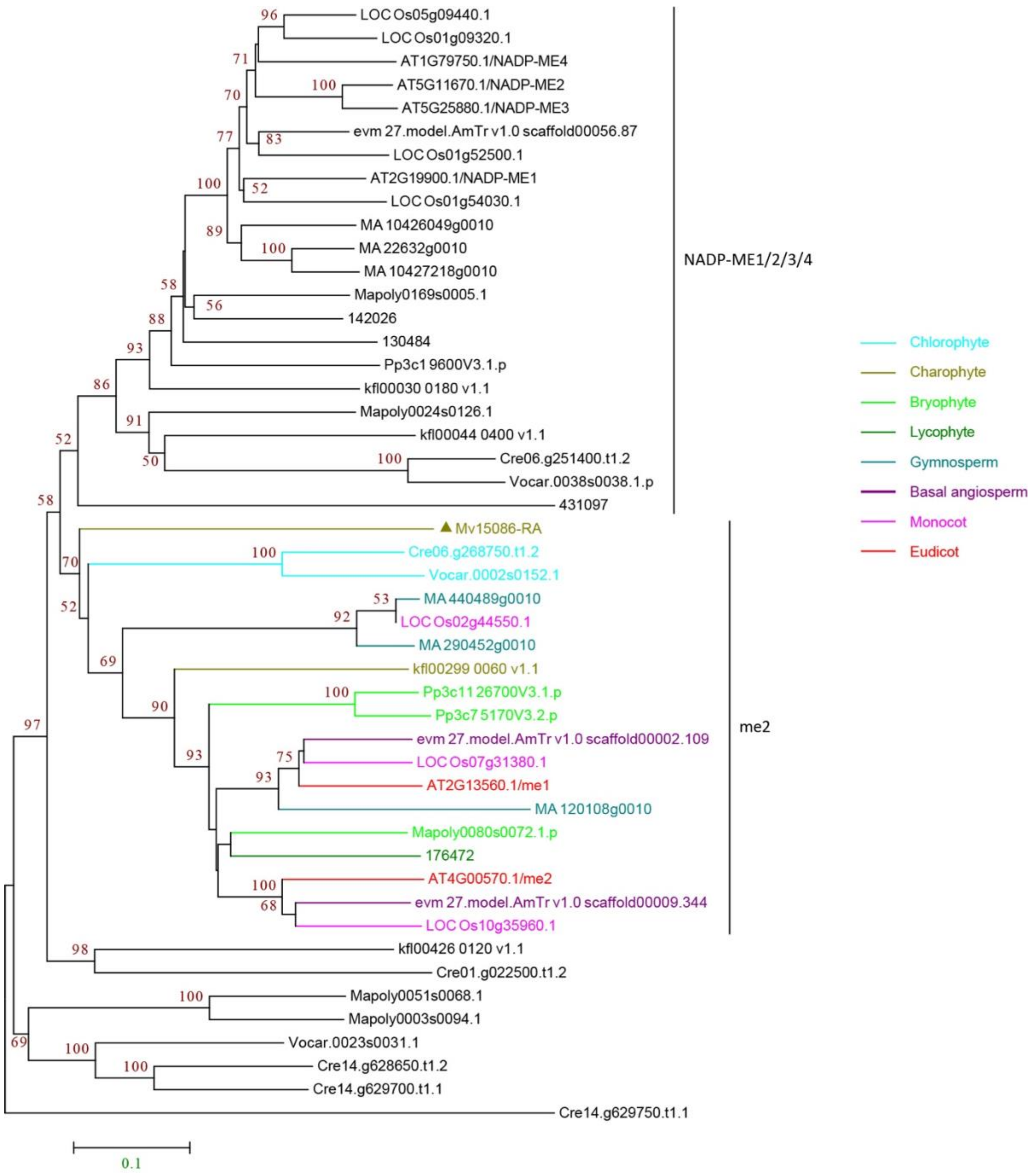

Data S1BL. Phylogenetic relationships of MALIC ENZYME 2 (me2) proteins from *M. viride* and other evolutionarily representative species. The *M. viride* sequence was highlighted by a triangle. Numbers on the major branches indicate bootstrap values (> 50%) in 1,000 replicates.

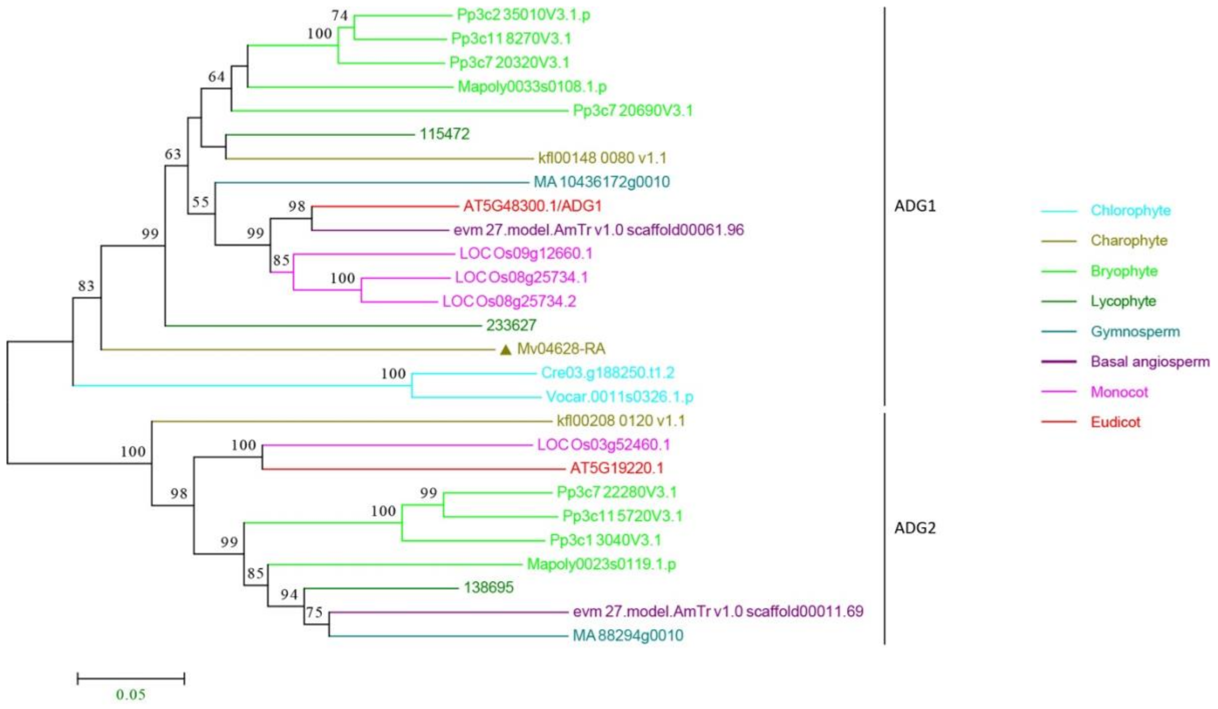

Data S1BM. Phylogenetic relationships of ADP GLUCOSE PYROPHOSPHORYLASE (ADG) proteins from *M. viride* and other evolutionarily representative species. The *M. viride* sequence was highlighted by a triangle. Numbers on the major branches indicate bootstrap values (> 50%) in 1,000 replicates.

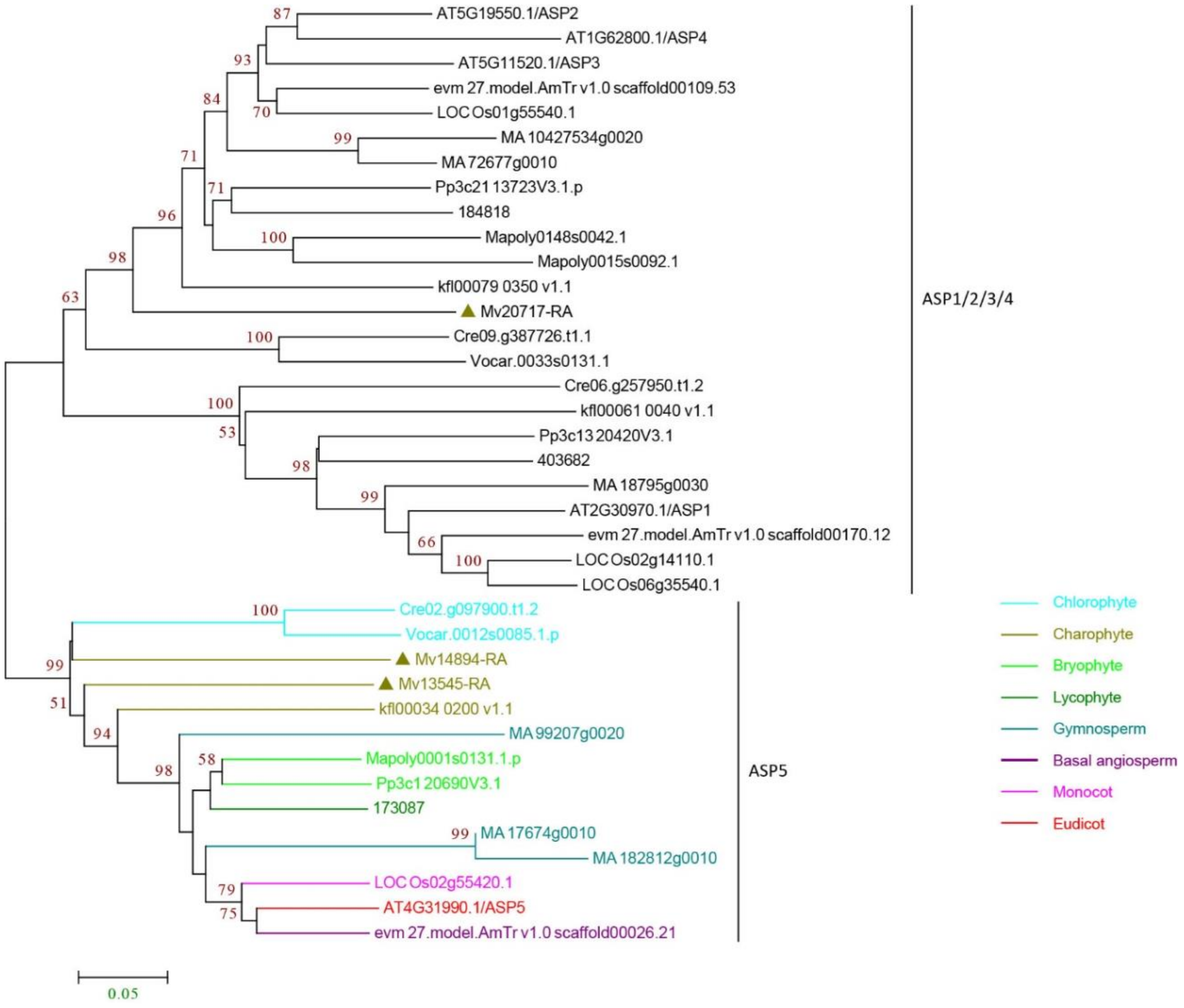

Data S1BN. Phylogenetic relationships of ASPARTATE AMINOTRANSFERASE (ASP) proteins from *M. viride* and other evolutionarily representative species. The *M. viride* sequences were highlighted by triangles. Numbers on the major branches indicate bootstrap values (> 50%) in 1,000 replicates.

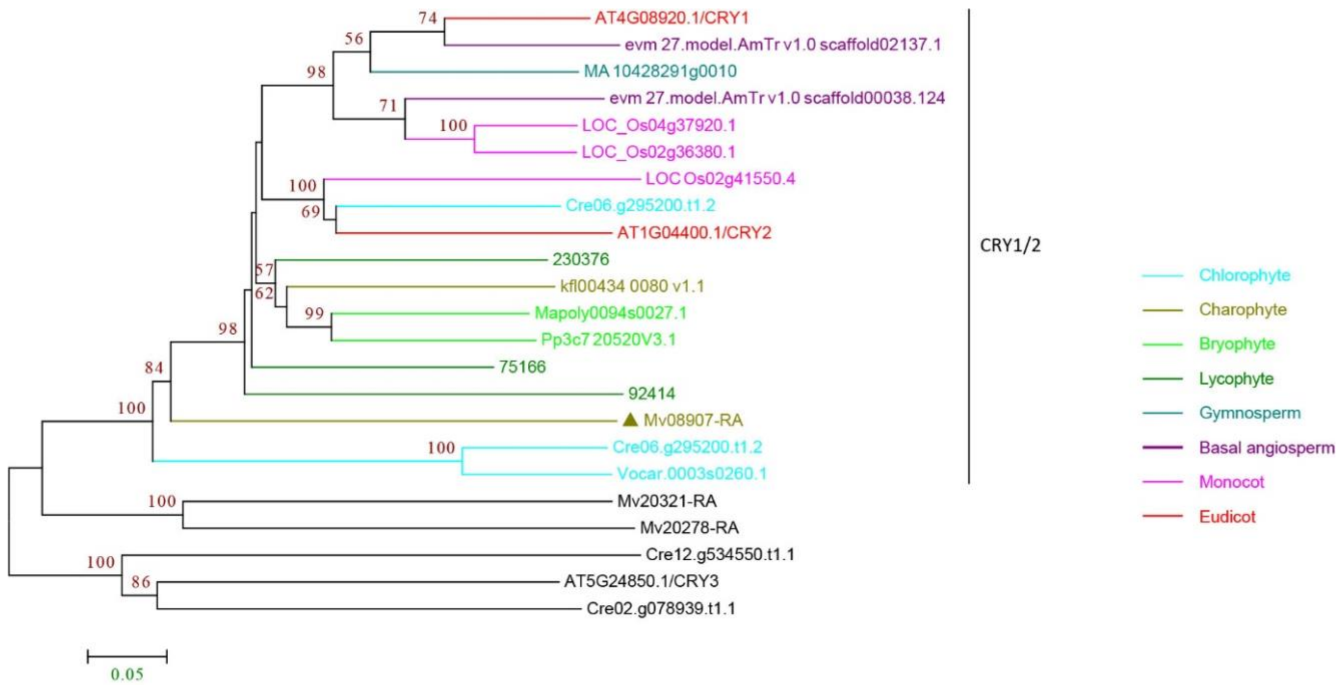

Data S1BO. Phylogenetic relationships of CRYPTOCHROME (CRY) proteins from *M. viride* and other evolutionarily representative species. The *M. viride* sequence was highlighted by a triangle. Numbers on the major branches indicate bootstrap values (> 50%) in 1,000 replicates.

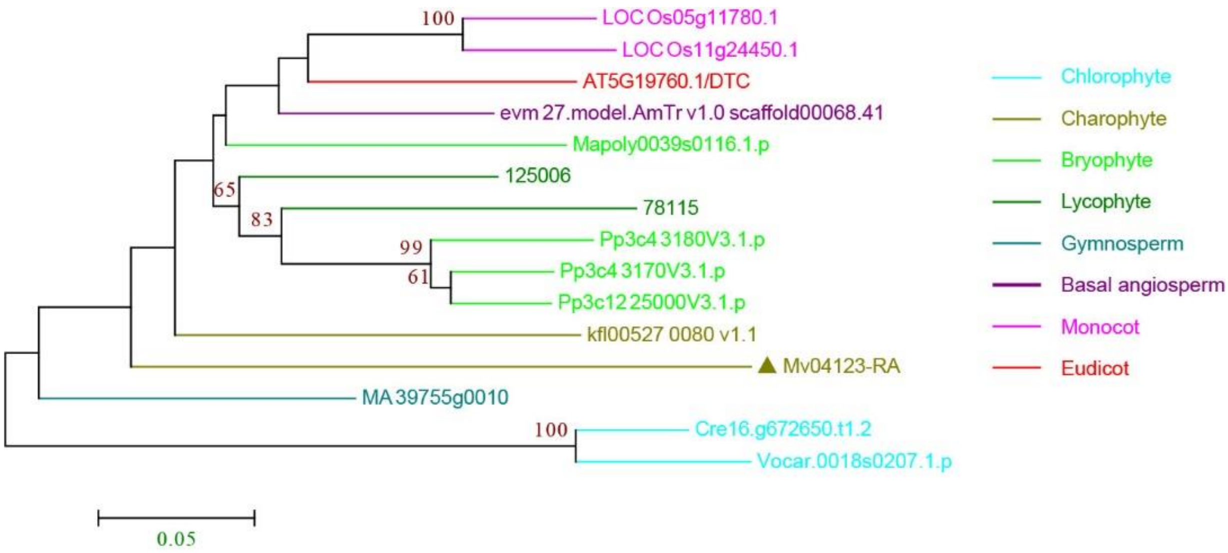

Data S1BP. Phylogenetic relationships of DTC proteins from *M. viride* and other evolutionarily representative species. The *M. viride* sequence was highlighted by a triangle. Numbers on the major branches indicate bootstrap values (> 50%) in 1,000 replicates.

VCH

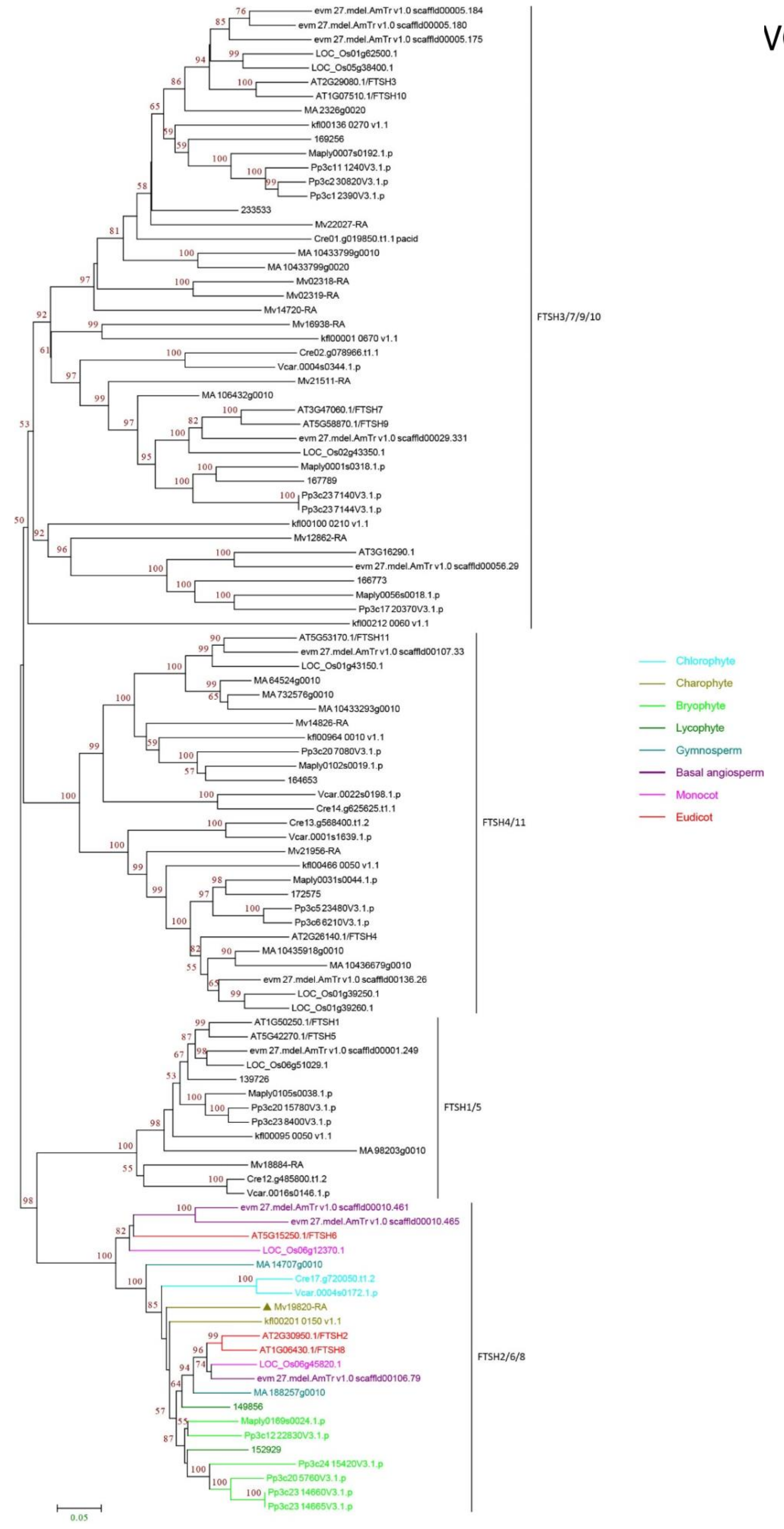
evm 27.mdel.AmTr v1.0 scaffld00005.184
evm 27.mdel.AmTr v1.0 scaffld00005.180
evm 27.mdel.AmTr v1.0 scaffld00005.175
LOC_Os01g62500.1
LOC_Os05g38400.1
AT2G29080.1/FTSH3
AT1G07510.1/FTSH10
MA 2326g0020
kfl00136 0270 v1.1
169256
Maply0007s0192.1.p
Pp3c11 1240V3.1.p
Pp3c2 30820V3.1.p
Pp3c1 2390V3.1.p
233533
Mv22027-RA
Cre01.g019850.t1.1 pacid
MA 10433799g0010
MA 10433799g0020
Mv02318-RA
Mv02319-RA
Mv14720-RA
Mv16938-RA
kfl00001 0670 v1.1
Cre02.g078966.t1.1
Vcar.0004s0344.1.p
Mv21511-RA
MA 106432g0010
AT3G47060.1/FTSH7
AT5G58870.1/FTSH9
evm 27.mdel.AmTr v1.0 scaffld00029.331
LOC_Os02g43350.1
Maply0001s0318.1.p
167789
Pp3c23 7140V3.1.p
Pp3c23 7144V3.1.p
kfl00100 0210 v1.1
Mv12862-RA
AT3G16290.1
evm 27.mdel.AmTr v1.0 scaffld00056.29
166773
Maply0056s0018.1.p
Pp3c17 20370V3.1.p
kfl00212 0060 v1.1
FTSH3/7/9/10
AT5G53170.1/FTSH11
evm 27.mdel.AmTr v1.0 scaffld00107.33
LOC_Os01g43150.1
MA 64524g0010
MA 732576g0010
MA 10433293g0010
Mv14826-RA
kfl00964 0010 v1.1
Pp3c20 7080V3.1.p
Maply0102s0019.1.p
164653
Vcar.0022s0198.1.p
Cre14.g625625.t1.1
Cre13.g568400.t1.2
Vcar.0001s1639.1.p
Mv21956-RA
kfl00466 0050 v1.1
Maply0031s0044.1.p
172575
Pp3c5 23480V3.1.p
Pp3c6 6210V3.1.p
AT2G26140.1/FTSH4
MA 10435918g0010
MA 10436679g0010
evm 27.mdel.AmTr v1.0 scaffld00136.26
LOC_Os01g39250.1
LOC_Os01g39260.1
FTSH4/11
AT1G50250.1/FTSH1
AT5G42270.1/FTSH5
evm 27.mdel.AmTr v1.0 scaffld00001.249
LOC_Os06g51029.1
139726
Maply0105s0038.1.p
Pp3c20 15780V3.1.p
Pp3c23 8400V3.1.p
kfl00095 0050 v1.1
MA 98203g0010
Mv18884-RA
Cre12.g485800.t1.2
Vcar.0016s0146.1.p
FTSH1/5
evm 27.mdel.AmTr v1.0 scaffld00010.461
evm 27.mdel.AmTr v1.0 scaffld00010.465
AT5G15250.1/FTSH6
LOC_Os06g12370.1
MA 14707g0010
Cre17.g720050.t1.2
Vcar.0004s0172.1.p
Mv19820-RA
kfl00201 0150 v1.1
AT2G30950.1/FTSH2
AT1G06430.1/FTSH8
LOC_Os06g45820.1
evm 27.mdel.AmTr v1.0 scaffld00106.79
MA 188257g0010
149856
Maply0169s0024.1.p
Pp3c12 22830V3.1.p
152929
Pp3c24 15420V3.1.p
Pp3c20 5760V3.1.p
Pp3c23 14660V3.1.p
Pp3c23 14665V3.1.p
FTSH2/6/8
0.05
Chlorophyte
Charophyte
Bryophyte
Lycophyte
Gymnosperm
Basal angiosperm
Monocot
Eudicot

Data S1BQ. Phylogenetic relationships of FTSH proteins from *M. viride* and other evolutionarily representative species. The *M. viride* sequence was highlighted by a triangle. Numbers on the major branches indicate bootstrap values (> 50%) in 1,000 replicates.

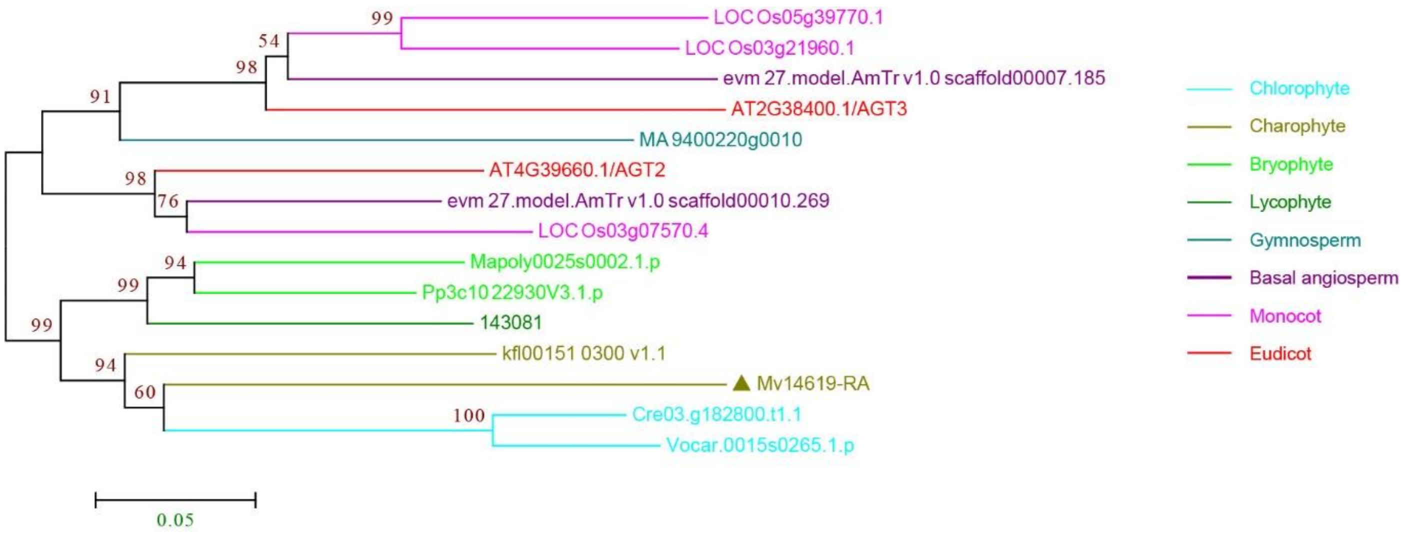

Data S1BR. Phylogenetic relationships of ALANINE:GLYOXYLATE AMINOTRANSFERASE (AGT) proteins from *M. viride* and other evolutionarily representative species. The *M. viride* sequence was highlighted by a triangle. Numbers on the major branches indicate bootstrap values (> 50%) in 1,000 replicates.

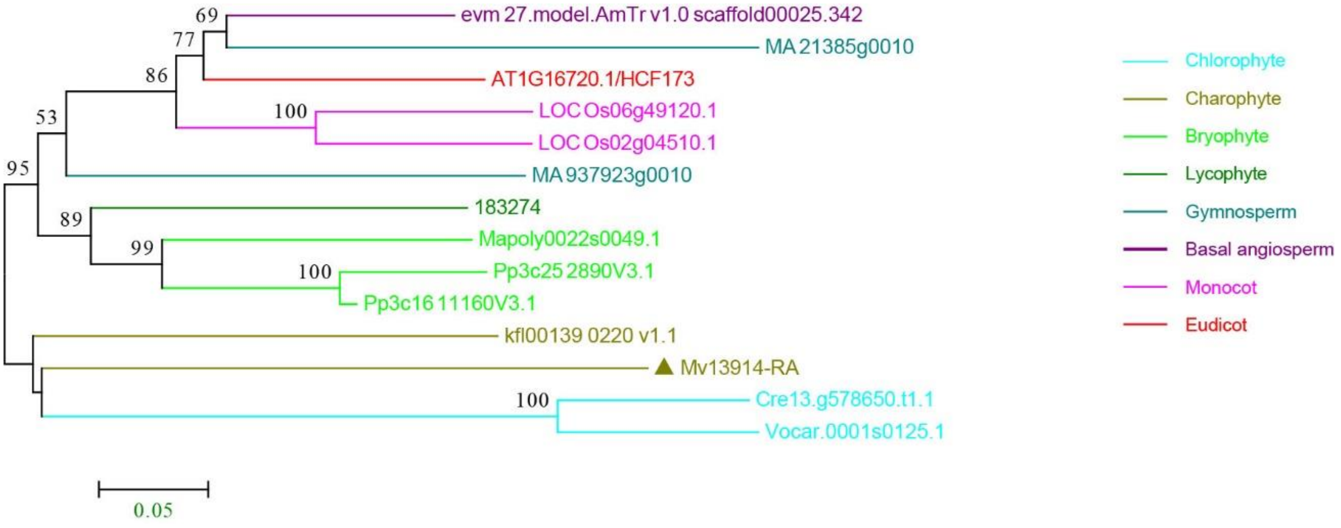

Data S1BS. Phylogenetic relationships of HIGH CHLOROPHYLL FLUORESCENCE PHENOTYPE 173 (HCF173) proteins from *M. viride* and other evolutionarily representative species. The *M. viride* sequence was highlighted by a triangle. Numbers on the major branches indicate bootstrap values (> 50%) in 1,000 replicates.

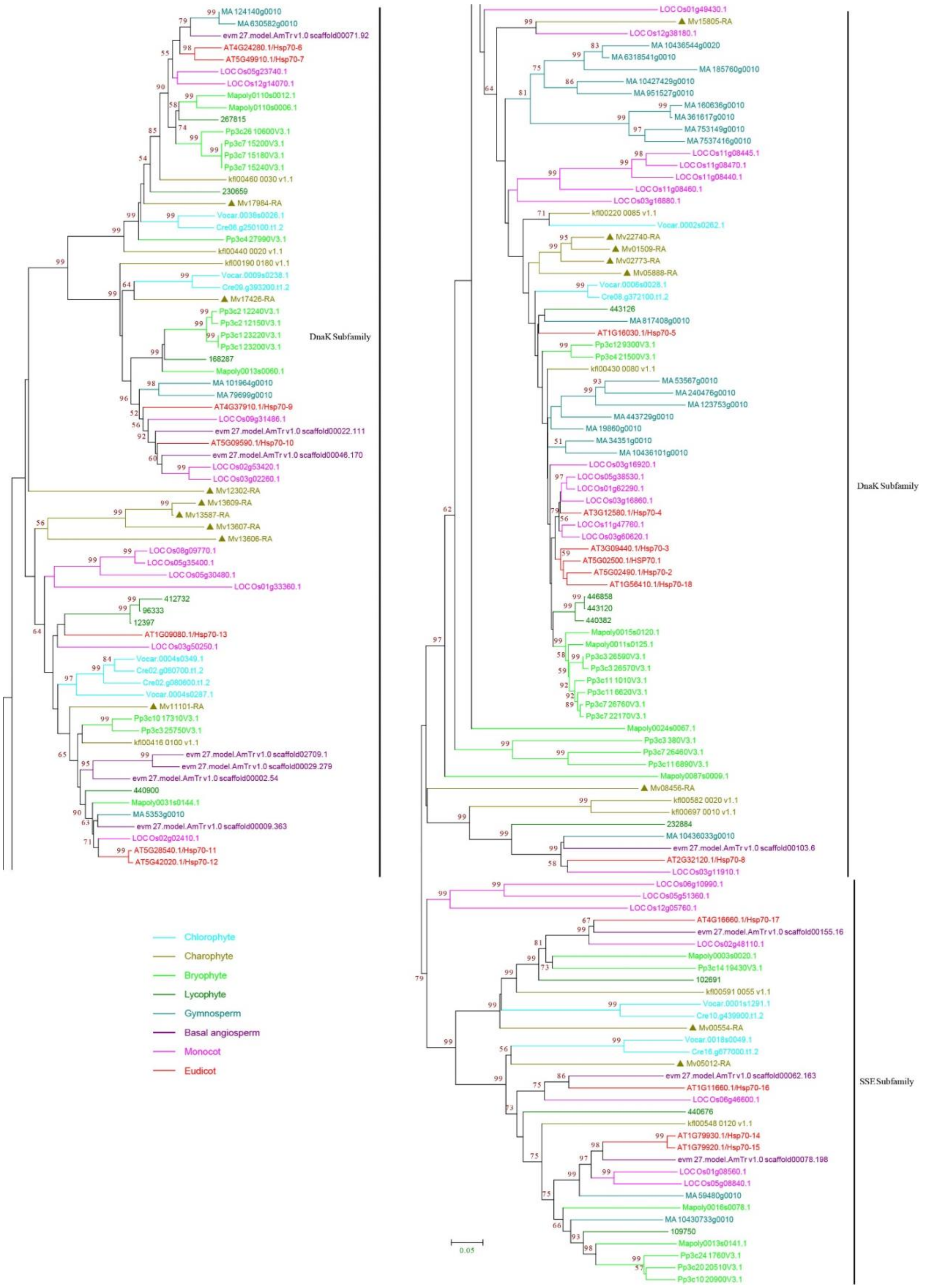

Data S1BT. Phylogenetic relationships of heat shock protein 70 (HSP70) proteins from *M. viride* and other evolutionarily representative species. The *M. viride* sequences were highlighted by triangles. Numbers on the major branches indicate bootstrap values (> 50%) in 1,000 replicates.

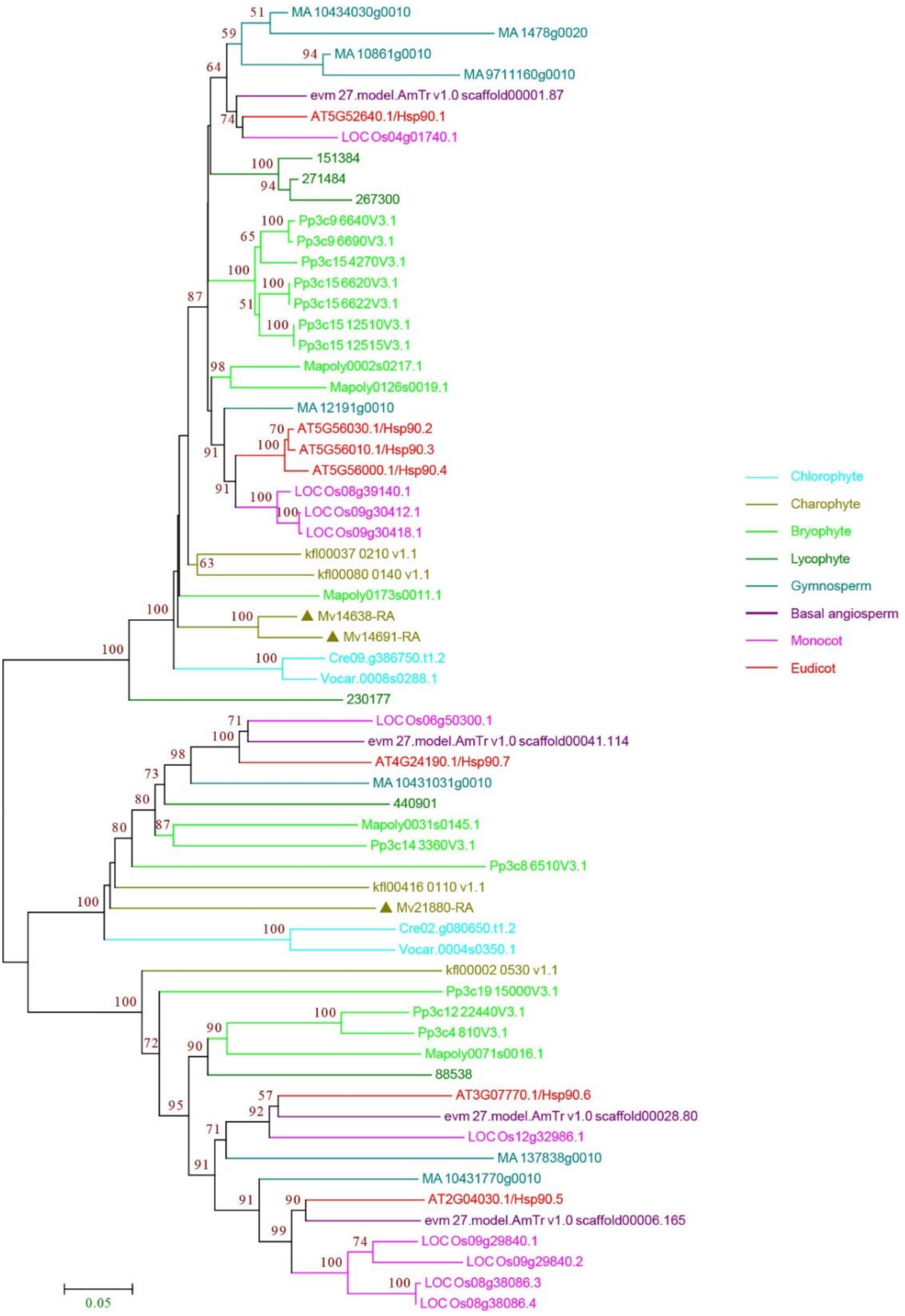

Data S1BU. Phylogenetic relationships of heat shock protein 90 (HSP90) proteins from *M. viride* and other evolutionarily representative species. The *M. viride* sequences were highlighted by triangles. Numbers on the major branches indicate bootstrap values (> 50%) in 1,000 replicates.

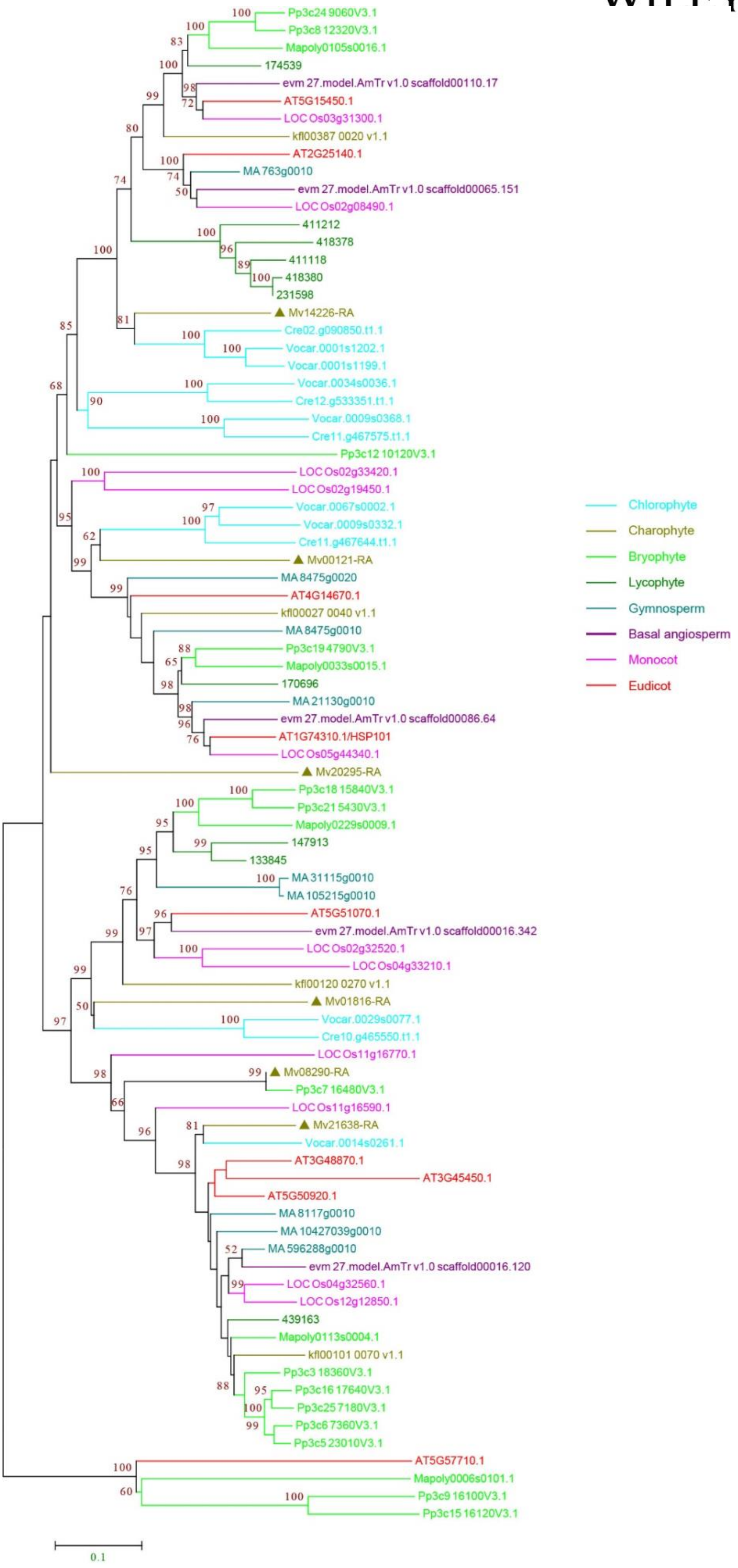
Pp3c24 9060V3.1
Pp3c8 12320V3.1
Mapoly0105s0016.1
174539
evm 27.model.AmTr v1.0 scaffold00110.17
AT5G15450.1
LOC Os03g31300.1
kfl00387 0020 v1.1
AT2G25140.1
MA 763g0010
evm 27.model.AmTr v1.0 scaffold00065.151
LOC Os02g08490.1
411212
418378
411118
418380
231598
Mv14226-RA
Cre02.g090850.t1.1
Vocar.0001s1202.1
Vocar.0001s1199.1
Vocar.0034s0036.1
Cre12.g533351.t1.1
Vocar.0009s0368.1
Cre11.g467575.t1.1
Pp3c12 10120V3.1
LOC Os02g33420.1
LOC Os02g19450.1
Vocar.0067s0002.1
Vocar.0009s0332.1
Cre11.g467644.t1.1
Mv00121-RA
MA 8475g0020
AT4G14670.1
kfl00027 0040 v1.1
MA 8475g0010
Pp3c19 4790V3.1
Mapoly0033s0015.1
170696
MA 21130g0010
evm 27.model.AmTr v1.0 scaffold00086.64
AT1G74310.1/HSP101
LOC Os05g44340.1
Mv20295-RA
Pp3c18 15840V3.1
Pp3c21 5430V3.1
Mapoly0229s0009.1
147913
133845
MA 31115g0010
MA 105215g0010
AT5G51070.1
evm 27.model.AmTr v1.0 scaffold00016.342
LOC Os02g32520.1
LOC Os04g33210.1
kfl00120 0270 v1.1
Mv01816-RA
Vocar.0029s0077.1
Cre10.g465550.t1.1
LOC Os11g16770.1
Mv08290-RA
Pp3c7 16480V3.1
LOC Os11g16590.1
Mv21638-RA
Vocar.0014s0261.1
AT3G48870.1
AT3G45450.1
AT5G50920.1
MA 8117g0010
MA 10427039g0010
MA 596288g0010
evm 27.model.AmTr v1.0 scaffold00016.120
LOC Os04g32560.1
LOC Os12g12850.1
439163
Mapoly0113s0004.1
kfl00101 0070 v1.1
Pp3c3 18360V3.1
Pp3c16 17640V3.1
Pp3c25 7180V3.1
Pp3c6 7360V3.1
Pp3c5 23010V3.1
AT5G57710.1
Mapoly0006s0101.1
Pp3c9 16100V3.1
Pp3c15 16120V3.1
0.1
Chlorophyte
Charophyte
Bryophyte
Lycophyte
Gymnosperm
Basal angiosperm
Monocot
Eudicot

Data S1BV. Phylogenetic relationships of heat shock protein 100 (HSP100) proteins from *M. viride* and other evolutionarily representative species. The *M. viride* sequences were highlighted by triangles. Numbers on the major branches indicate bootstrap values (> 50%) in 1,000 replicates.

References

[1] H. Du, Z. Liang, S. Zhao, M. G. Nan, L. S. Tran, K. Lu, Y. B. Huang, J. N. Li, *Sci. Rep.* **2015**, *5*, 11037.

[2] N. Pires, L. Dolan, *Mol. Biol. Evol.* **2010**, *27*, 862.

[3] J. K. Okamuro, B. Caster, R. Villarroel, M. Van Montagu, K. D. Jofuku, *Proc. Natl. Acad. Sci. USA* **1997**, *94*, 7076.

[4] F. Licausi, M. Ohme-Takagi, P. Perata, *New Phytol.* **2013**, *199*, 639.

[5] K.-J. Dietz, M. O. Vogel, A. Viehhauser, *Protoplasma* **2010**, *245*, 3.

[6] T. Nakano, K. Suzuki, T. Fujimura, H. Shinshi, *Plant Physiol.* **2006**, *140*, 411.

[7] A. Safi, A. Medici, W. Szponarski, S. Ruffel, B. Lacombe, G. Krouk, *Curr. Opin. Plant Biol.* **2017**, *39*, 159.

[8] M. Jakoby, B. Weisshaar, W. Dröge-Laser, J. Vicente-Carbajosa, J. Tiedemann, T. Kroj, F. Parcy, *Trends Plant Sci.* **2002**, *7*, 106.

[9] W. Dröge-Laser, B. L. Snoek, B. Snel, C. Weiste, *Curr. Opin. Plant Biol.* **2018**, *45*, 36.

[10] K. Swaminathan, K. Peterson, T. Jack, *Trends Plant Sci.* **2008**, *13*, 647.

[11] Y. Wang, D. Deng, R. Zhang, S. Wang, Y. Bian, Z. Yin, *Mol. Biol. Rep.* **2012**, 39, 6267.

[12] F. Y. Peng, R. J. Weselake, *Theor. Appl. Genet.* **2013**, *126*, 1305.

[13] E. Morgunova, Y. Yin, A. Jolma, K. Dave, B. Schmierer, A. Popov, N. Eremina, L. Nilsson, J. Taipale, *Nat. Commun.* **2015**, *6*, 10050.

[14] R. Rauber, C. Cabreira, L. B. de Freitas, A. C. Turchetto-Zolet, M. Margis-Pinheiro, *Mol. Phylogenet. Evol.* **2016**, *99*, 225.

[15] P. von Koskull-Doring, K. D. Scharf, L. Nover, *Trends Plant Sci.* **2007**, *12*, 452.
